# Chromosome-level genome assembly of *Ophiorrhiza pumila* reveals the evolution of camptothecin biosynthesis

**DOI:** 10.1038/s41467-020-20508-2

**Published:** 2021-01-15

**Authors:** Amit Rai, Hideki Hirakawa, Ryo Nakabayashi, Shinji Kikuchi, Koki Hayashi, Megha Rai, Hiroshi Tsugawa, Taiki Nakaya, Tetsuya Mori, Hideki Nagasaki, Runa Fukushi, Yoko Kusuya, Hiroki Takahashi, Hiroshi Uchiyama, Atsushi Toyoda, Shoko Hikosaka, Eiji Goto, Kazuki Saito, Mami Yamazaki

**Affiliations:** 1 Graduate School of Pharmaceutical Sciences, Chiba University, Chiba, Japan; 2 Plant Molecular Science Center, Chiba University, Chiba, Japan; 3 Kazusa DNA Research Institute, Kisarazu, Chiba Japan; 4 RIKEN Center for Sustainable Resource Science, Yokohama, Japan; 5 Graduate School of Horticulture, Chiba University, Chiba, Japan; 6 RIKEN Center for Integrative Medical Sciences, Yokohama, Japan; 7 Medical Mycology Research Center, Chiba University, Chiba, Japan; 8 College of Bioresource Sciences, Nihon University, Fujisawa, Kanagawa Japan; 9 Advanced Genomics Center, National Institute of Genetics, Mishima, Shizuoka Japan; 10 Present Address: RIKEN Center for Sustainable Resource Science, Yokohama, Japan

**Keywords:** Metabolomics, Plant biotechnology, Plant evolution, Plant genetics, Secondary metabolism

## Abstract

Plant genomes remain highly fragmented and are often characterized by hundreds to thousands of assembly gaps. Here, we report chromosome-level reference and phased genome assembly of *Ophiorrhiza pumila*, a camptothecin-producing medicinal plant, through an ordered multi-scaffolding and experimental validation approach. With 21 assembly gaps and a contig N50 of 18.49 Mb, *Ophiorrhiza* genome is one of the most complete plant genomes assembled to date. We also report 273 nitrogen-containing metabolites, including diverse monoterpene indole alkaloids (MIAs). A comparative genomics approach identifies strictosidine biogenesis as the origin of MIA evolution. The emergence of strictosidine biosynthesis-catalyzing enzymes precede downstream enzymes’ evolution post γ whole-genome triplication, which occurred approximately 110 Mya in *O. pumila*, and before the whole-genome duplication in *Camptotheca acuminata* identified here. Combining comparative genome analysis, multi-omics analysis, and metabolic gene-cluster analysis, we propose a working model for MIA evolution, and a pangenome for MIA biosynthesis, which will help in establishing a sustainable supply of camptothecin.

## Abstract (web summary)

*Ophiorrhiza pumila* is a medicinal plant that can produce the anti-cancer monoterpene indole alkaloid (MIA) camptothecin. Here, the authors report its genome assembly and propose a working model for MIA evolution and biosynthesis through comparative genomics, synteny, and metabolic gene cluster analyses.

## Introduction

Cancer is the leading cause of death worldwide, with 70% of cases occurring in low- and middle-income countries^1^. Among the 30 essential anticancer drugs categorized by the World Health Organization in 2015, several molecules, including topotecan, irinotecan, vincristine, and vinorelbine, are extracted from plants or derived from plant monoterpene indole alkaloids (MIAs), such as camptothecin and catharanthine^2,3^. MIAs are natural products derived from (*S*)-strictosidine, with a monoterpene moiety derived from secologanin, an iridoid class of monoterpenes, and the indole moiety from tryptamine, a decarboxylation product of the amino acid tryptophan (Supplementary Fig. 1). The monoterpenoid moiety of strictosidine then undergoes extensive modifications catalyzed by various enzymes to form diverse MIAs, which represent over 2500 known metabolites^2^. Most of our current understanding of MIA biosynthesis is restricted to the vinca alkaloid synthesis pathway elucidated in *Catharanthus roseus*^4–9^. Camptothecin, another strictosidine-derived molecule and one of the most potent anticancer MIAs, is the precursor for the commercial synthesis of topotecan and irinotecan, and several other camptothecin derivatives are in clinical trials at different stages^10,11^. The camptothecin biosynthesis pathway and the mechanisms regulating its production remain unknown, even though it is one of the most promising plant-derived antitumor drugs (Supplementary Fig. 1)^11^. Difficulties in extraction, the low content per gram dry weight of the producing plant tissues, and lack of sustainable resources have limited the development of camptothecin-derived and other anticancer MIAs, contributing to the unaffordability of cancer treatment for most patients.

*Ophiorrhiza pumila*, a fast-growing herbaceous plant from the Rubiaceae family, has emerged as a model plant for the study of MIA biosynthesis and regulation, and a sustainable source of camptothecin^12–14^. *O. pumila* hairy roots have been shown to accumulate high levels of camptothecin, and this plant has served as an experimental model for the understanding of MIA biosynthesis for over a decade^3,15,16^. Previous studies have found a correlation of camptothecin biosynthesis and accumulation with the conserved mutation of two amino acids in DNA topoisomerase I in camptothecin-producing plants, including species from the *Ophiorrhiza* genus, that allow the plants to survive camptothecin cytotoxicity^14,17^. While the basis of natural selection for plant species with resistance against camptothecin is relatively straightforward, how nature simultaneously evolved all the enzymes needed for camptothecin biosynthesis is not yet clear. Understanding MIAs’ evolution and biosynthesis is also essential for building sustainable alternate production platforms to facilitate access to these lifesaving compounds. With an estimated 20 million new cancer cases globally by 2025 and an economic burden estimated at $1.16 trillion in 2010^18^, meeting the increasing demands for camptothecin and other anticancer MIAs has become a daunting challenge and requires immediate attention. A high-quality reference genome for an anticancer MIA-producing plant is the first step toward achieving this goal.

In this study, we show the advantage of ordered multitiered scaffolding with assembly validation at each stage to achieve a highly contiguous genome assembly. This strategy allows us to derive a near-finished and experimentally validated reference and phased genome assembly of *O. pumila*. Our results show the relevance of experimental validation for next-generation plant genome assemblies. Further, we expand the nitrogen-containing metabolome space of *Ophiorrhiza* by using complete stable isotope labeling and cheminformatics approaches. A combination of comparative genomics approaches suggest the emergence of strictosidine synthase (STR) as a key event in the evolution of strictosidine-derived MIA biosynthesis in plants. Our results suggest that the enzymes involved in the committed step of a specialized metabolite biosynthesis pathway directs evolution and innovation in the plant kingdom. This study, by establishing a high-quality genome and metabolome resource for *O. pumila*, provides a foundation for yield improvement of valuable anticancer metabolites through synthetic biology and biotechnology.

## Results

### Multitiered scaffolding strategy to derive a high-quality plant genome assembly

**Fig. 1:**
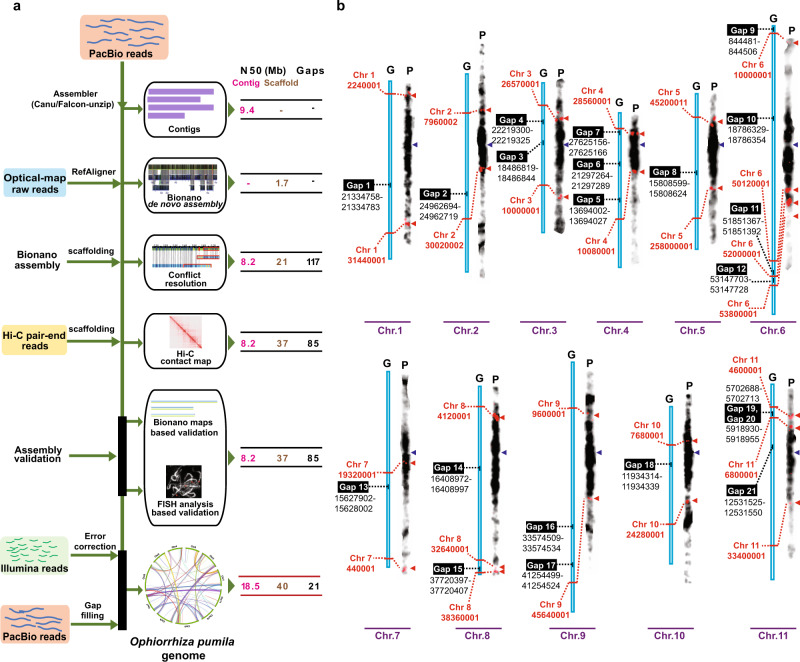
Multistage assembly validation to achieve chromosome-level genome assembly for a high repeat content plant genome. **a** The hierarchical genome assembly and assembly validation pipeline, and the improvement of assembly contiguity in terms of contig N50, scaffold N50, and assembly gaps of *O. pumila* genome. The assembly schema consistently corrects genome assembly from the point of contig-level assembly to the scaffold-level assembly, using orthogonal evidence and experimental validations. **b** Fluorescence in situ hybridization (FISH)-based validation of chromosome arms and orientation of scaffolds at the assembly gaps of *Ophiorrhiza* genome. The red triangle represents the site of FISH probe signal identified for a given chromosome, and the dotted line corresponds to the position at the scaffold. The purple colored triangle represents putative centromere at the pachytene chromosome, representing densely packed genomic segment, and hence darkly stained. The FISH experiment was repeated twice, and for each experiment, at least ten slides for each chromosome were observed and verified for signals as shown in **b**. G assembled chromosome, P pachytene chromosome with a FISH signal.

Assembling a high-quality reference plant genome is challenging due to the inherent heterozygosity, polyploidy, and high repeat content of plant genomes. With the reduced cost of long-read sequencing and advances in scaffolding technologies; however, it has become feasible to achieve plant genome assemblies at the pseudomolecule level^19^. Next-generation sequencing (NGS) technologies, such as Bionano optical maps and Hi-C library sequencing provide valuable orthogonal evidence to validate and improve reference genomes, and to derive chromosome-level genome assemblies^20,21^. Despite this progress, nearly all plant genomes remain highly fragmented, with hundreds to thousands of remaining assembly gaps (Supplementary Fig. 2). In this study, we used a stepwise integration and assembly validation approach using four complementary NGS technologies to derive *O. pumila* de novo genome assembly: PacBio single-molecule reads (~122×), Illumina paired-end reads (~96×), Bionano optical mapping (~250×), and Hi-C library sequencing (~90×; Fig. 1a and Supplementary Data 1). A contig-level genome assembly, optimized for different parameters and raw-read lengths using PacBio reads in the Canu assembler^22^, spanned the entire genome in 243 contigs (Supplementary Data 2). Validation of the contig-level assembly using Bionano optical maps identified 15 assembly conflicts (Supplementary Fig. 3), which were manually examined and subsequently corrected. The contig-level genome assembly was subjected to scaffolding using either Bionano or Hi-C libraries, or sequential scaffolding using all possible combinations, i.e., scaffolding first through Bionano and subsequently through Hi-C, or vice versa. We observed an advantage of sequential scaffolding over a single scaffolding approach, with scaffolding first through Bionano and then by Hi-C as the best combination (Table 1). The final *Ophiorrhiza* genome assembly of 439.90 Mb was achieved within 31 contigs, with contig and scaffold N50 values as 18.49 and 40.06 Mb, respectively (Fig. 1a, Table 1, and Supplementary Fig. 4). Full-length chromosome arms were assembled for half of the *Ophiorrhiza* chromosomes, with only 21 remaining assembly gaps in the entire genome assembly, including the 11 difficult-to-assemble and highly repetitive centromeres (Fig. 1b). We also adopted our sequential scaffolding strategy to achieve phased diploid assembly of *Ophiorrhiza* using preliminary contig-level assembly derived from Falcon-unzip^23^, resulting in 11 chromosomes with scaffold N50 for haplotig1 and haplotig2 as 40.36 and 42.83 Mb, respectively (Supplementary Figs. 4 and 5, and Supplementary Data 3 and 4).

**Table 1:** *O. pumila* reference genome assembly statistics at different stages and combinations of scaffolding.

Assembly	Number of contigs	Number of scaffolds	Number of contigs assigned to scaffolds	Contig N50 (Mb)	Scaffold N50 (Mb)	Number of gaps	Assembly size (Mb)
PacBio^a^ only (Canu assembly)	243	—	—	9.38	—	—	449.00
Bionano de novo Optical Map	—	458	—	—	1.68	—	442.00
PacBio + Optical^b^ Map	108	45	83	8.21	21.05	117	442.00
PacBio + Hi-C^c^	213	34	198	9.39	40.80	96	441.00
PacBio + Hi-C + Optical Map^d^	239	26	208	8.21	24.17	91	441.90
PacBio + Optical Map + Hi-C^e^	108	13	108	8.21	37.11	85	439.00
PacBio + Optical Map + Hi-C + PbJelly (PacBio) + genome polishing (final *O. pumila* reference genome)	31	13 (11 Chromosomes + 1 MT + 1 CP)	31	18.49	40.06	21	439.90

*O. pumila* is a medicinal plant that can produce the anticancer monoterpene indole alkaloid (MIA) camptothecin. Here, the authors report its genome assembly, and propose a working model for MIA evolution and biosynthesis through comparative genomics, synteny, and metabolic gene cluster analyses.

^a^PacBio refers to contig assembly derived using Pacbio reads only and Canu^22^ assembler.

^b^Pacbio + Optical Map refers to Pacbio contig-level assembly scaffolded by Bionano de novo assembly.

^c^PacBio + Hi-C refers to Pacbio contig-level assembly scaffolded by Hi-C library sequencing datasets.

^d^PacBio + Hi-C + Optical Map refers to Pacbio + Hi-C assembly scaffolded by Bionano de novo assembly.

^e^PacBio + Optical Map + Hi-C refers to Pacbio + Optical Map assembly scaffolded by Hi-C library sequencing datasets.

Compared to previously published genome assemblies of anticancer MIA-producing plant species, namely, *C. roseus* v 2.0 (contig N50: 0.076 Mb)^6^, *Rhazya stricta* (contig N50: 0.08 Mb)^24^, *Gelsemium sempervirens* v 3.0 (contig N50: 0.051 Mb)^6^, and *Camptotheca acuminata* (contig N50: 0.1 Mb)^25^, we achieved an improvement of over 180 times from the next best assembly in terms of contig N50 value (Supplementary Fig. 2). Assembly validation, starting from PacBio, followed by Bionano, and finally Hi-C provided a stepwise improvement in assembly contiguity. With few remaining genome gaps and contig-level completeness, the *Ophiorrhiza* reference and phased genomes are, to the best of our knowledge, the most contiguous and complete de novo reference plant genomes to date (Supplementary Fig. 2 and Supplementary Data 5).

### Experimental validation for gaps is the missing piece of the next-generation genome assembly pipeline

**Fig. 2:**
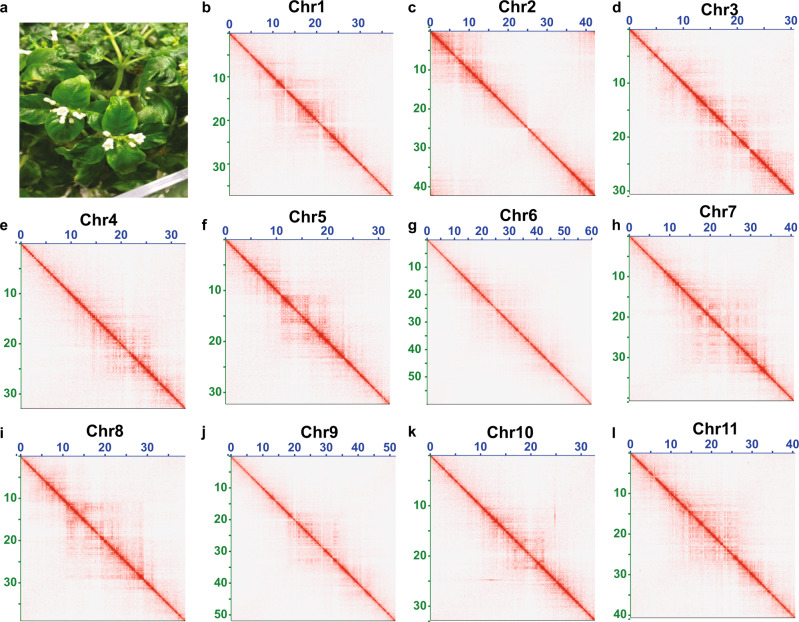
Hi-C contact map of high-quality chromosome-scale genome assembly of *O. pumila*. **a** *O. pumila* plant under controlled growth conditions. **b**–**l** A Hi-C contact matrix visualization for individual chromosomes of *O. pumila* reference genome assembly. The pixel intensity represents the count of Hi-C links at 150-Kb size windows on the chromosomes on a logarithmic scale. Darker red color indicates higher contact probability, while white space represents no or fewer contacts.

While scaffolding approaches align and order contigs, orientation of contigs within an assigned scaffold are prone to errors due to the lack of sequencing data evidence at the assembly gaps. This shortcoming of modern-day genome assemblies is widely acknowledged, yet it has been neglected and is regarded as a technical limitation of the genome assembly pipeline. To achieve an experimentally validated and accurate plant genome assembly, we next performed fluorescence in situ hybridization (FISH) analysis for each scaffold of the *Ophiorrhiza* genome at the assembly gaps (Supplementary Data 6). All chromosomes, except chromosome 2, showed FISH signals at the ends of contiguous scaffold arms separated by assembly gaps, in accordance with our genome assembly (Fig. 1b and Supplementary Fig. 6). For chromosome 2, an orientation misalignment was detected at the gap between two contigs, as the sites that were expected to be adjacent to the assembly gaps were detected at the end of each of the chromosomal arms (Supplementary Fig. 6). We used FISH evidence to correct contig orientation within assigned scaffolds for chromosome 2. The chromosome sizes estimated using FISH analysis were consistent with the corresponding assembled chromosome sizes for the *O. pumila* genome (Supplementary Fig. 7 and Supplementary Table 1). The accuracy of the finalized reference and phased genome assemblies was also supported by Bionano optical map data, Bionano de novo assembly, and Hi-C chromosomal contact matrix (Fig. 2, and Supplementary Figs. 8 and 9).

We compared the *Ophiorrhiza* genome karyotype with the reconstructed ancient eudicot karyotype (AEK)^26^ and *Vitis vinifera* genome^27^, which are regarded to have emerged after the whole-genome triplication of AEK with the least karyotype rearrangement^26,28^. Synteny analysis of the *Ophiorrhiza* genome showed synteny depths of 3:1 and 2:2 with the AEK and *V. vinifera* genomes, respectively (Supplementary Figs. 10 and 11). The results showed a conserved and colinear relationship for the *Ophiorrhiza* genome with AEK and *V. vinifera* with minimal rearrangements, with 1 chromosome fission and 11 chromosome fusions, resulting in the present-day karyotype of the *Ophiorrhiza* genome. Comparison with AEK further supported the accuracy of the karyotypic order of the *Ophiorrhiza* genome and identified the whole-genome triplication (γ) shared among eudicots, with no sign of further genome duplications (Supplementary Fig. 10a–c).

**Fig. 3:**
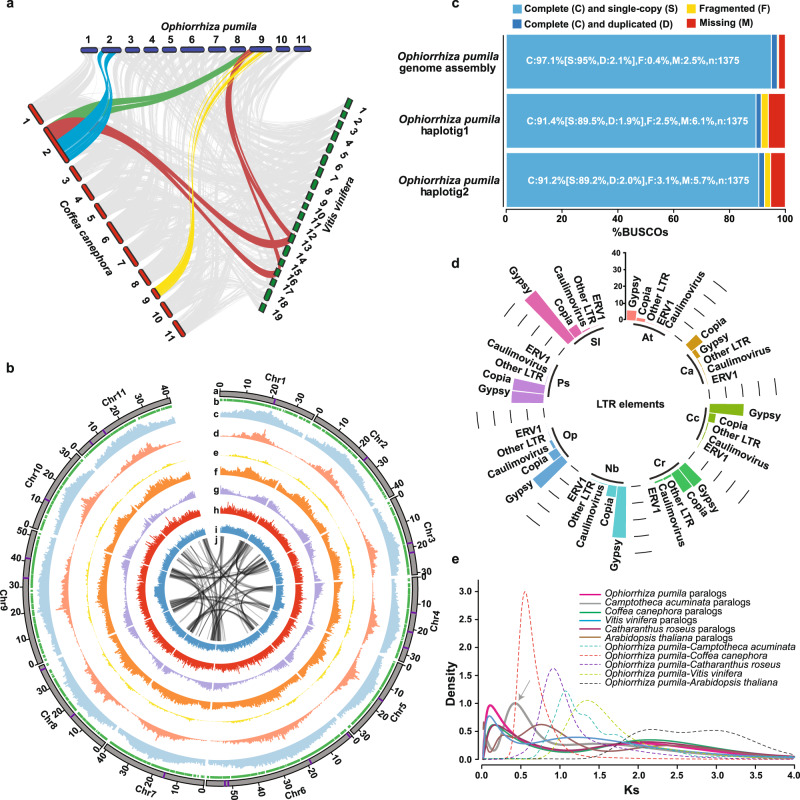
*O. pumila* genomic landscape and convergent evolution of monoterpene indole alkaloid biosynthesis. **a** Synteny blocks between *O. pumila*, *C. canephora*, and *V. vinifera*. Synteny analysis suggested karyotypic rearrangement between chromosomes 2 and 9 of *O. pumila* and *C. canephora* genomes, respectively. **b** Characteristics of the 11 chromosomes of *O. pumila*. Track a–c corresponds to chromosomes (assembly gap is depicted by purple line at each chromosome), phasing blocks, and repetitive sequences, respectively. Track d–i corresponds to the distribution of long terminal repeat (LTR)-Gypsy, distribution of LTR-Copia, GC density, distribution of predicted gene models, SNP density, and indel density, respectively. Track j corresponds to syntenic blocks. The bar, representing chromosomes, are scaled to chromosome lengths. **c** Evaluation of *O. pumila* genome assemblies using Benchmarking Universal Single-copy Orthologs (BUSCO) analysis. **d** Percentage of genome content comprising LTR elements for eight plant species. At *Arabidopsis thaliana*, Ca *Camptotheca acuminata*, Cc *Coffea canephora*, Cr *Catharanthus roseus*, Nb *Nicotiana benthamiana*, Op *O. pumila*, Ps *Papaver somniferum*, Sl *Solanum lycopersicum*. **e** Synonymous substitution rate (Ks) distribution plot for paralogs and orthologs of *O. pumila* with other eudicots as shown through colored continuous and dotted lines, respectively. The arrow highlights the recent whole-genome duplication identified in *C. acuminata* genome. Source data are provided as a Source data file.

We next compared the *Ophiorrhiza* genome with the genome of *Coffea canephora*^29^, also from the Rubiaceae family, which has previously been used as a representative asterid genome for paleogenomic interpretations^28^. Synteny analysis between the *Ophiorrhiza* and *C. canephora* genomes showed a potential karyotype rearrangement, with chromosomes 2 and 9 of *Ophiorrhiza* showing syntenic relationships with chromosome 2 of the coffee genome (Fig. 3a and Supplementary Fig. 10e). The chromosome 2 segment of the coffee genome with a syntenic relationship with chromosome 9 of the *Ophiorrhiza* genome showed associations with chromosome 13 and chromosome 16 of *V. vinifera* (Fig. 3a). Given that the genome of *Ophiorrhiza* is near-complete with only 21 assembly gaps, whereas the coffee genome is only 80% complete and has 7250 assembly gaps, the observed syntenic and potential karyotype rearrangement could very well be a possible misassembly of the coffee genome (Supplementary Fig. 12). Furthermore, the chromosome sizes estimated through FISH analysis supported the *Ophiorrhiza* genome assembly (Supplementary Fig. 7 and Supplementary Table 1). Therefore, we tested the hypothesis of coffee genome misassembly through FISH analysis in *Coffea arabica*, an allotetraploid genome resulting from hybridization between *C. canephora* and *Coffea eugenioides*. FISH analysis using probes designed for the two segments on chromosome 2 of the coffee genome that were in apparent synteny with two chromosomes of the *Ophiorrhiza* genome produced signals on two different chromosomes instead of on the same chromosome. The FISH analysis suggested a possible misassembly in the coffee genome (Supplementary Fig. 13). Plant genome paleohistorical scenarios and gene-cluster-based specialized metabolite analysis assume accurate gene order for the inputted plant genomes. However, this is most likely not the case for most plant genomes, as very few genomes have been verified. *Ophiorrhiza* genome assembly provides a valuable resource to validate and improve other plant genomes, and will serve as a model to understand the evolution of genome structure in asterids.

### Contrasting genomic features indicate convergent evolution of MIA biosynthesis

The *O. pumila* genome comprises 32,389 gene models, including 778 transcription factors (TFs), 2827 noncoding RNAs, 87 microRNAs (miRNAs), and 493 tRNAs (Fig. 3b, Supplementary Figs. 14 and 15, and Supplementary Data 7–10). The distribution of predicted gene models along the respective chromosomes was in a V-shaped valley form, with low gene density near the centromere for all 11 chromosomes, including for the chromosome 2 after correction based on FISH evidence (Fig. 3b). We used repeat analysis to locate the pericentromeric regions, which were confirmed using FISH analysis (Supplementary Fig. 16). The near-complete genome assembly of *Ophiorrhiza* also allowed us to identify telomere regions for all eleven chromosomes (Supplementary Data 11). Benchmarking Universal Single-Copy Ortholog (BUSCO)^30^ analysis using the *Ophiorrhiza* reference and phased genome assemblies showed 97.1% and 91.2–91.4% completeness, respectively (Fig. 3c).

Repeat analysis showed 58.17% *Ophiorrhiza* genome comprised of transposable elements (TEs), the majority being long terminal repeat (LTR) retroelements, ~75% of which were classified as Gypsy-LTRs (Supplementary Data 12). A comparison of TEs across other plant species showed Gypsy-LTR as the dominant repeat class in *O. pumila*, *R. stricta*, *C. roseus*, *C. canephora*, *Nicotiana benthamiana*^31^, and *Solanum lycopersicum*^32^, while the *C. acuminata* genome was dominated by Copia-LTR repeats (Fig. 3d, Supplementary Fig. 17, and Supplementary Data 12). OrthoFinder^33^ analysis-based gene family classification for 33 plant genomes representing broader plant lineages, followed by phylogenetic analysis for single-copy genes using PAML MCMCTREE^34^ software estimated divergence times for *Ophiorrhiza* from coffee, *C. roseus*, and *C. acuminata* at ~47, ~68, and ~120 Mya, respectively (Supplementary Fig. 18). Synonymous substitutions per synonymous site (Ks) for the paralogs of *O. pumila*, *C. roseus*, *C. acuminata*, *Arabidopsis thaliana*, and coffee genome showed a distinct peak at Ks = 2, which represents well-reported and conserved whole-genome triplication across core eudicots^35^ (Fig. 3e and Supplementary Fig. 19). The Ks plot for the paralogs of *O. pumila* did not show signs of any recent whole-genome duplication (WGD), while the Ks plot for the paralogs of *C. acuminata* suggested a previously unreported WGD, occurring after the γ event, at Ks-peak 0.469, which we estimated at ~42.27 Mya (Fig. 3e and Supplementary Figs. 18–20). Using Ks values for synteny blocks and orthologs between coffee and *Ophiorrhiza*, and the estimated divergence time, we determined the Ks per year (*r*) to be 6.54e−9 for the Rubiaceae.

MIA biosynthesis is known to be remarkably restricted to Gentianales, such as in Rubiaceae^24^. The exceptions are MIA quinolone derivatives, e.g., camptothecin, which is synthesized by Rubiaceae members, such as *Ophiorrhiza*, as well as by *C. acuminata* in the Cornales. WGDs and TE are regarded as key mechanisms for evolving novel features in plants^36–39^. The differential repeat profiles across the *O. pumila, C. roseus*, *R. stricta*, and *C. acuminata* genomes and WGD in *C. acuminata* suggest different trajectories of acting evolutionary forces, yet resulting in similar chemotypes across MIA-producing plants from the Gentianales and Cornales orders. These results raise the possibility of either a convergent evolution of MIA biosynthesis in otherwise distant plant species or an ancient origin of MIA biosynthesis, which is subsequently lost repeatedly across plant species, while retained by the producing plants.

### Diverse indole alkaloids consistent with enzyme families evolved in the *Ophiorrhiza* genome

**Fig. 4:**
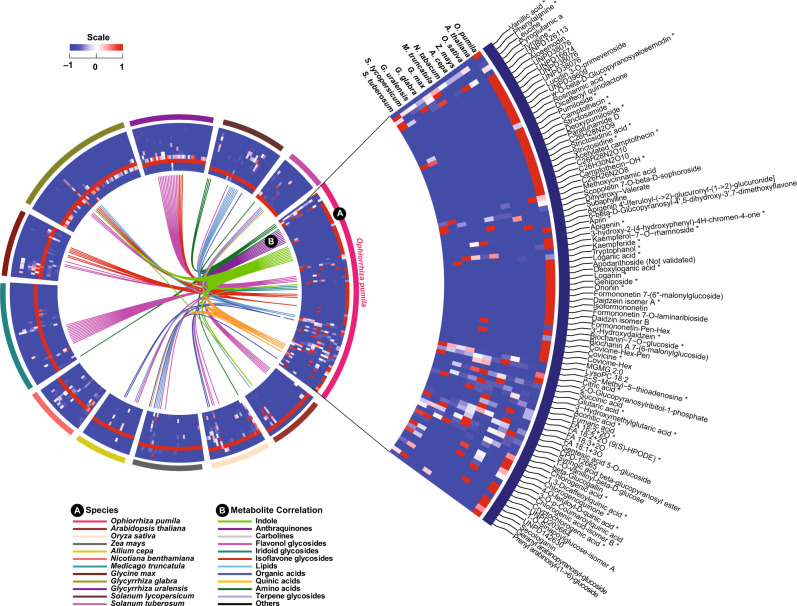
*Ophiorrhiza pumila* metabolome, assigned using ^13^C and ^15^N stable isotope labeling, compared with metabo-space of 11 plant species. Circuit A shows plant species used to compare metabolome space with that of *O. pumila*, and circuit B shows the connections between metabolite features, and are based on metabolite network relationships defined by a correlation coefficient >0.85. Highly accumulated metabolites across 12 plant species and their relationships in the form of metabo-ontology and scaled accumulation levels as a heat map are shown here. Metabolites were filtered (log10 intensity > 3.9) and assigned to the *O. pumila* category. If a metabolite was not detected in *O. pumila*, then it was assigned to the plant category with the highest accumulation compared to the rest of the plant species. *Indicates chemically assigned metabolites based on pure standards or MS/MS analysis using public databases. Source data are provided as a Source data file.

Stable isotope labeling, coupled with high-resolution mass spectrometry, offers a powerful approach to assign atom numbers and chemical information to the detected metabolites. It increases the confidence in molecular formula determination for identified metabolite features by eliminating false positives, while considering elemental compositions^7,40,41^. To expand the known chemodiversity of *O. pumila*, particularly nitrogen-containing specialized metabolites, we used a complete ^15^N-based stable isotope labeling and metabolome analysis approach as previously reported for complete ^13^C-based metabolome labeling for *O. pumila* and 11 other plant species (Supplementary Fig. 21)^7,40^. Complete ^15^N labeling of the *Ophiorrhiza* metabolome and previously acquired ^13^C-labeled metabolome annotation datasets allowed us to chemically assign 273 nitrogen-containing metabolites, mostly annotated as indole alkaloids (IAs), MIAs, and carboline moieties containing metabolites (Fig. 4, Supplementary Fig. 21, and Supplementary Data 13 and 14). The *Ophiorrhiza* metabolome showed distinct and diverse nitrogen-containing metabolites, including MIAs, when compared with previously analyzed plant metabolomes (Fig. 4)^40^. The MIA biosynthetic pathways are well conserved across producing plant species and are derived from strictosidine (or strictosidinic acid, in the case of *C. acuminata*)^42^. Along with derivatives of strictosidine, camptothecin, and known intermediates of the camptothecin biosynthetic pathway (Supplementary Fig. 1), MIAs such as eburnamonine, nothapodytine, vincamenine, and D-glucopyranosyl vincosamide have also been identified in *Ophiorrhiza*. Specialized metabolite classes, including MIAs, IAs, and anthraquinones, accumulated in a tissue-specific manner, with the highest levels in the root and hairy root, and low levels in the leaf tissues (Supplementary Figs. 22 and 23, and Supplementary Data 15). Several of the assigned MIAs were also reported previously in *C. roseus*, *G. sempervirens*, and *C. acuminata*^7^. Consistent with MIA accumulation, gene expression analysis showed tissue-specific expression of secoiridoid biosynthesis-related genes, with high expression in the root and hairy root, and low expression in the leaf and cell suspension culture of *O. pumila* (Supplementary Figs. 1, 24 and 26, and Supplementary Data 16–18). Expression analysis showed that homologs of genes associated with secoiridoid and MIA biosynthesis were highly coexpressed, and were considered strong candidate MIA biosynthesis genes in *O. pumila*. Secoiridoid biosynthesis genes were highly coexpressed with homologs of MIA biosynthesis-associated enzymes, including 10-hydroxycamptothecin *O*-methyltransferase, *O*-acetylstemmadenine oxidase (*ASO/PAS*), polyneuridine-aldehyde esterase (*PNAE*), perakine reductase (*PR*), rankinidine/humantenine-11-hydroxylase 3 (*RH11H*), sarpagan bridge enzyme (*SBE*), strictosidine beta-D-glucosidase (*SGD*), tabersonine-19-hydroxy-*O*-acetyltransferase (*T19AT*), tabersonine 3-oxygenase, and tetrahydroalstonine synthase (*THAS*; Supplementary Figs. 25–27). Integration of metabolome and transcriptome profiling for multiple tissues of *Ophiorrhiza* identified strong candidate MIA biosynthesis genes (Supplementary Fig. 27 and Supplementary Data 19). The expression of genes associated with secoiridoids and MIA biosynthesis showed a strong correlation with MIA accumulation, indicating that the *Ophiorrhiza* root and hairy roots are the sites of active biosynthesis (Supplementary Figs. 23, 26 and 27, and Supplementary Data 19).

**Fig. 5:**
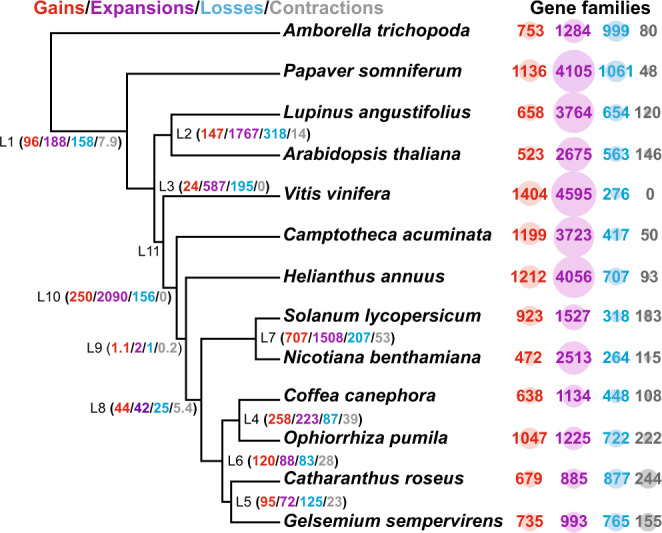
Evolution as gain (red), expansion (purple), loss (blue), or contraction (gray) of the orthogene families in context of phylogenetic profile. Count software was used to calculate posterior probabilities to capture the dynamics of evolution through the reconstruction of ancestral gene content and changes at the key nodes, which correspond to the lineage-specific gene characteristics. The circle radius is scaled based on the number of genes assigned to a specific category. Source data are provided as a Source data file.

To understand the features of conserved gene sets associated with MIA biosynthesis and their evolution, we compared the *Ophiorrhiza* genome with that of 12 plant species, including three MIA-producing plant genomes (*C. acuminata*, *C. roseus*, and *G. sempervirens*). Using OrthoFinder-based gene classification, we identified a total of 15,943 orthogroups shared among the four MIA-producing plants, with 64.8% of orthogroups being common to all four species and 513 orthogroups being specific to the MIA-producing plants (Supplementary Fig. 28 and Supplementary Data 20). Gene ontology (GO) enrichment analysis of the MIA-specific orthogroups showed dioxygenase (OG:0016701) and oxidoreductase activities (OG:0051213), biochemical reactions essential for MIA biosynthesis and diversification, as the significantly enriched GO terms (Supplementary Fig. 28c). The orthogroups were further analyzed to infer the ancestral and lineage-specific gene content along the phylogenetic tree, and to calculate posterior probabilities for gene family evolution and dynamics. Compared to *Ophiorrhiza* and other MIA-producing plants, the *C. acuminata* genome showed massive gene expansion, which is consistent with the WGD identified in this study. Overall, the *Ophiorrhiza* genome showed gain and expansion for 1047 and 1225 orthogroups, respectively (Fig. 5 and Supplementary Data 21), and GO enrichment analysis showed significant enrichment of processes associated with dioxygenase activity, hydrolase activities, hydroxyl-methyl glutaryl-CoA, and oxidoreductase activities. Several genes assigned to secoiridoid and MIA biosynthesis and highly correlated with metabolite accumulation patterns in *Ophiorrhiza* were among the gene families expanded or gained specifically to MIA-producing plants (Supplementary Data 19–21). Unless there exists a completely independent pathway toward the biosynthesis of MIAs across producing plants, an unlikely scenario, the shared chemotype suggests the possible existence of conserved gene families and secondary metabolite gene clusters within these plant genomes, with positive gene selection or gene expansion occurring during the evolution of MIA biosynthesis^2,7,42^.

### Strictosidine biogenesis is the driving force of MIA evolution in plants

**Fig. 6:**
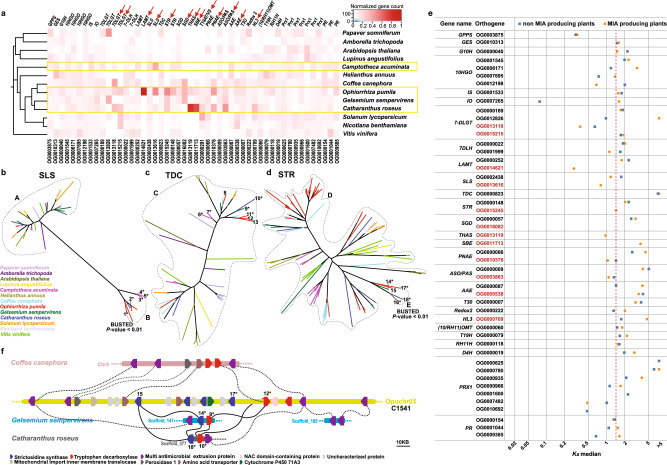
Emergence of strictosidine synthase, the starting point for the evolution of monoterpene indole alkaloid biosynthesis in plants. **a** Normalized gene count data for orthogene families assigned to MIA biosynthesis across 13 plant species. The yellow box indicates MIA-producing plants. Normalization was performed by dividing gene count data for a specific plant species with total number of genes assigned to a given orthogene family. The red arrows at the top of gene names highlight the orthogene families specifically gained or expanded in the MIA-producing plant species. **b**–**d** Maximum likelihood phylogenetic tree based on genes from orthogene OG0002438^(A)^ and OG0013616^(B)^, representing *SLS*; orthogene OG0000823^(C)^, representing *TDC*; and orthogene OG0000148^(D)^ and OG0015245^(E)^, representing *STR* coding genes. BUSTED analysis was performed using genes in the group B as test set and the genes in the group A as the background for SLS, and genes in the group E as test set against genes in the group D as the background for STR. *Functionally characterized genes. 1: Opuchr02_g0013060-1.1; 2: CRO_T109448; 3: Cac_g017137.t1; 4: Cac_g012666.t1; 5: Cac_g012664.t1; 6: Cac_g023139.t1; 7: Cac_g018974.t1; 8: Opuchr05_g0005520-1.1; 9: Gs_TDC; 10: CRO_T125328; 11: Opu_chr01_g0011270-1.1; 12: Opuchr05_g0008370-1.1; 13: Opuchr01_g0009570-1.1; 14: Gs_STR; 15: Opuchr05_g0008180-1.1; 16: AAY81922; 17: Opuchr05_g0008300-1.1; 18: CRO_T125329. **e** Median synonymous substitutions per synonymous sites (Ks) distribution for genes associated with secoiridoid and MIA biosynthesis pathways across MIA-producing and non-MIA-producing plants. Dotted red line refers to Ks median for functional STR in *Ophiorrhiza* genome. Orthogenes highlighted by red color are the orthogenes specifically gained or expanded in MIA-producing plants as shown in **a**. **f** Conserved gene clusters, C1541, essential for the biosynthesis of strictosidine-derived MIAs. Dashed lines show syntenic blocks, while scaffolds for each genome are shown through distinct color. *GPPS* geranyl diphosphate synthase, *GES* geraniol synthase, *G10H* geraniol 10-hydroxylase, *10-HGO* 10-hydroxygeraniol oxidoreductase, *IS* iridoid synthase, *IO* iridoid oxidase, *7-DLGT* 7-deoxyloganetin glucosyltransferase, *7-DLH* 7-deoxyloganic acid 7-hydroxylase, *LAMT* loganic acid *O*-methyltransferase, *SLS* secologanin synthase, *TDC* tryptophan decarboxylase, *STR* strictosidine synthase, *SGD* strictosidine beta-D-glucosidase, *THAS* tetrahydroalstonine synthase, *PNAE* polyneuridine-aldehyde esterase, *ASO/PAS* *O*-acetylstemmadenine oxidase, *AAE* acetylajmaline esterase, *TEX1*tabersonine 6,7-epoxidase 1, *HL3* hydrolase 3, *10OMT* 10-hydroxycamptothecin *O*-methyltransferase, *T19H* tabersonine-19-hydroxy-*O*-acetyltransferase, *RH11H* rankinidine/humantenine-11-hydroxylase 3, *D4H* deacetoxyvindoline 4-hydroxylase, *Prx1* peroxidase 1, *PR* perakine reductase. Source data are provided as a Source data file.

Orthogene families with genes assigned to early secoiridoid biosynthesis pathway (Supplementary Fig. 26a), including geraniol synthase (*GES*), geraniol 10-hydroxylase (*G10H*), 10-hydroxygeraniol oxidoreductase (*10-HGO*), iridoid synthase, iridoid oxidase, 7-deoxyloganetin glucosyltransferase (*7-DLGT*), and 7-deoxyloganic acid 7-hydroxylase (*7-DLH*), were well conserved and uniformly represented in all 13 plant genomes (Fig. 6a). However, gene families corresponding to late secoiridoid pathway genes, including loganic acid *O*-methyltransferase (*LAMT*) and secologanin synthase (*SLS*), were specifically gained and expanded in MIA-producing plants. While orthogroups corresponding to *LAMT* (OG0000252) and *SLS* (OG0002438) were retained in all species, OG0014621 (*LAMT*) was specifically gained in *O. pumila*, and OG0013616 (*SLS*) was specifically gained and expanded in the *C. acuminata*, *C. roseus*, and *Ophiorrhiza* genomes (Fig. 6a).

The generation of strictosidine is the committed step in the biosynthesis of MIAs in Gentianales. Orthogene-based classification of genes showed that STR coding enzymes were divided into two distinct groups. Orthogene family OG0000148 was represented in all plants except *Papaver somniferum* and *Amborella trichopoda*, while OG0015245, which included all known and functionally characterized genes for strictosidine synthesis, was specifically gained and expanded in Gentianales, including those from *O. pumila*, *C. roseus*, and *G. sempervirens* (Fig. 6a and Supplementary Data 21). Compared to *C. roseus* and *G. sempervirens*, which included single-copy genes in the orthogene family OG0015245, the *O. pumila* genome included two genes resulting from tandem duplication, namely, Opuchr05_g0008300 and Opuchr05_g0008180. Gene family analysis of functional *STR* homologs of *O. pumila* across the genomes of 32 plant species showed no representatives from ancient plant genomes, monocots, and AEK (Supplementary Fig. 29). The biosynthesis of MIAs in *C. acuminata* was previously attributed to strictosidinic acid and not strictosidine, which supports the absence of genes in orthogroup OG0015245^42,43^.

An alternate pathway for MIA biosynthesis in *C. acuminata* is through strictosidinic acid, which is synthesized by the condensation of secologanic acid with tryptamine^42^. Recently, Yang et al. functionally characterized Cac_g012666.t1 (CYP72A610) and Cac_g017137.t1 (CYP72A565) as bifunctional SLS-like enzymes, catalyzing the synthesis of loganic acid from 7-deoxyloganic acid and subsequently to secologanic acid^43^. The tryptophan decarboxylase (TDC) enzyme, which synthesizes tryptamine from tryptophan, is represented by the orthogene OG0000823. Unlike orthogenes representing functional *STR* and *SLS*, OG0000823 was present in all plant species that we analyzed, suggesting an essential role of TDC in amino acid metabolism in plants. Phylogenetic analysis for orthogenes representing *SLS* and *STR* showed genes being assigned to two distinct groups, those present specifically in MIA-producing plants and those that are also present in the non-MIA-producing plants (Fig. 6b–d). Similar to functionally characterized STR and SLS enzymes, orthogenes corresponding to enzymes associated with MIA biosynthesis, such as SGD, THAS, SBE, T19AT, PNAE, ASO/PAS, and PR, were also specifically expanded in *Ophiorrhiza* and at least one of the other three MIA-producing plant genomes (Fig. 6a and Supplementary Data 21). Phylogenetic analysis of these specialized enzymes also showed MIA plant-specific gene family formation, which included previously functionally characterized enzymes involved in MIA biosynthesis (Supplementary Figs. 30–40). Phylogenetic analysis and gene gain/expansion analysis showed a positive selection of gene sets specific to MIA-producing plants. Moreover, the emergence of specialized enzymes, including STR and bifunctional SLS, specific to MIA-producing plants and catalyzing the synthesis of strictosidine showed the importance of strictosidine biogenesis in facilitating the evolution of novel enzymes for MIA biosynthesis and diversification.

WGDs and small-scale duplications (SSDs) are the major source of evolutionary novelty, providing gene pools to evolve new or specialized functions, and also play an important role in speciation^44–46^. Theoretical models for the evolutionary trajectories of duplicated genes propose that, in most cases, one copy of the duplicated gene retains the original function, while another copy neutrally evolves without any selective constraints, thus resulting in its inactivation due to the accumulation of deleterious mutations or even deletion^47^. In a small fraction of cases, the duplicate gene undergoes gain-of-function mutations and is retained through positive selection forces^46^. The native genes undergo a rapid rate of mutation and thus should have a lower Ks value than the ancestral genes, resulting in the emergence of a new enzyme with a novel function. However, in contrast to this hypothesis, the median Ks for enzymes associated with secoiridoids and MIA biosynthesis in both MIA-producing and nonproducing plants was 1.586, which corresponds to the Ks median for OG0015245 (STR; Fig. 6e). MIA-producing plants showed a lower Ks median than non-MIA-producing plants for OG000040 (*G10H*), OG0012198 (*10-HGO*), OG0013118 (*7-DLGT*), OG0014621 (*LAMT*), OG0013616 (*SLS*), OG0015245 (*STR*), OG0011713 (*THAS*), OG0010376 (*PNAE*), OG0003863 (*ASO*), and OG0007482 (*PRX1*), suggesting a higher rate of substitution and evolution for these specialized enzymes. The median Ks for TDC, an enzyme essential for tryptophan metabolism across all plant species, showed a similar substitution rate in MIA- and non-MIA-producing plants (Fig. 6e and Supplementary Data 22). For camptothecin-producing plants, the median Ks for genes associated with MIA biosynthesis was significantly smaller in *O. pumila* and *C. acuminata* but higher in the coffee genome, which shares otherwise high genome collinearity and sequence similarity with the *Ophiorrhiza* genome (Supplementary Fig. 41). A higher median Ks for the MIA-associated orthogene families in the coffee genome suggests an ancient origin for the genes that otherwise would have been actively evolving, as suggested by the smaller median Ks value. Phylogenetic analysis by maximum likelihood test and BUSTED analysis^48^ for orthogene families gained or expanded in MIA-producing plant genomes indicated positive selection for *SLS* (OG0013616) and *STR* (OG0015245) specific to the MIA-producing plant species (Fig. 6b, d).

### Evolution of MIA biosynthesis is centered around secondary metabolite gene clusters

To gain insight into whether the physical location plays a role in positive selection and expansion of genes associated with MIA biosynthesis, we performed gene cluster analysis in the *Ophiorrhiza* genome. In total, we identified 358 metabolic gene clusters in the *O. pumila* genome, representing 3551 gene models across 11 chromosomes (Supplementary Data 23). Coexpression analysis for a given gene cluster showed a low Pearson correlation coefficient (PCC) value, with the median PCC value for 3/4^th^ of the gene clusters being <0.3 (Supplementary Data 24). We identified metabolic gene clusters, such as C1394, C1620, C1708, C1709, C1909, C1925, and C1959, which included 7–18 gene members, and were highly coexpressed. Using the presence of at least one orthogene family associated with MIA biosynthesis pathways from *O. pumila* gene models in the identified gene clusters as the selection criteria, we assigned 33 gene clusters as putative MIA gene clusters (Supplementary Data 18, 23 and 24). While MIA biosynthesis-associated genes were highly coexpressed, MIA gene clusters showed low coexpression values among member genes. Among the MIA gene clusters, C1385 and C1749 showed coexpression between member genes. Although our results showed low coexpression coefficients for genes within a gene cluster, we observed 261 gene clusters with at least one pair of genes having PCC values over 0.7. The fact that several of these gene clusters included genes with no expression within the tissues analyzed was one of the reasons for the low PCC scores within individual gene clusters. The behavior of the identified gene clusters and associated coexpression values were similar to previously reported trends in other plant genomes^49,50^, suggesting a lack of widespread coexpression among member genes of associated gene clusters in plants.

**Fig. 7:**
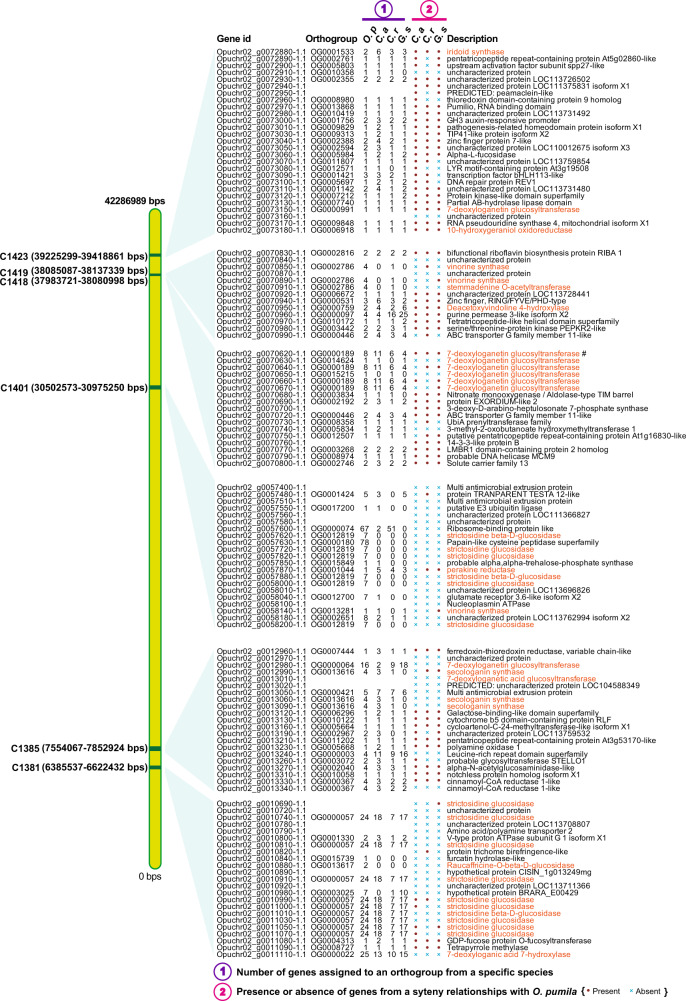
Monoterpene indole alkaloid gene clusters identified in chromosome 2 in the *Ophiorrhiza* genome. In total, we identified 33 MIA gene clusters in *Ophiorrhiza* genome distributed across eight of its chromosomes (Supplementary Figs. 42–48). Conserved synteny at gene clusters across MIA-producing plants suggest role of secondary metabolite clusters toward evolution of specialized metabolites in plants. The position of the gene cluster is scaled based on chromosome size and its physical position on the chromosome. The gene description colored as orange represents genes with functions associated with MIAs biosynthesis. O.p *Ophiorrhiza pumila*, C.a *Camptotheca acuminata*, C.r *Catharanthus roseus*, G.s *Gelsemium sempervirens*.

Out of 358 secondary metabolite gene clusters identified, 91 gene clusters included at least one orthogroup specific to MIA-producing plants (Supplementary Data 20 and 23–26). One of the key gene clusters identified in the *Ophiorrhiza* genome was C1541, which included functionally characterized TDC (Opuchr05_g0008370-1.1) and STR (Opuchr05_g0008300-1.1 and Opuchr05_g0008180-1.1) together with enzymes annotated as amino acid transporters, cytochrome P450 71A3, NAC domain-containing protein, and multi-antimicrobial extrusion (MATE) protein (Fig. 6f). Previously, *C. roseus* and *G. sempervirens* genome analysis have also reported the presence of a STR-TDC-MATE gene cluster^6^. Synteny analysis showed conserved synteny between the *C. roseus* and *G. sempervirens* genomes at C1541, which included both functionally characterized STR, TDC, and MATE proteins. The coffee genome, which shares significant collinearity and sequence similarity with the *Ophiorrhiza* genome, also showed conserved gene cluster collinearity with C1541 (Fig. 6f, and Supplementary Figs. 12 and 15). However, the relatively conserved functional STR orthogenes present across strictosidine-derived MIA-producing plants at gene cluster C1541 were lost in the coffee genome, while other features of the gene cluster, including TDC coding enzymes, were retained (Fig. 6f). Comparative genome analysis between *Ophiorrhiza* and coffee showed high sequence similarity and gene localization along chromosomes, yet loss of functional *STR* within the coffee genome at the gene cluster may have limited the opportunity to direct evolution toward MIA biosynthesis, which also explains the higher median Ks for enzymes associated with MIA biosynthesis (Supplementary Fig. 41). The entire secoiridoid biosynthetic pathway and MIA biosynthesis-associated genes from the *Ophiorrhiza* genome were present in 29 out of 33 MIA gene clusters (Supplementary Fig. 26). The association of coexpressed genes assigned to secoiridoid and MIA biosynthesis pathways with gene clusters was statistically significant based on two-sided Fisher’s exact test with corrected *p* value < 0.05. Furthermore, at least one-third of the member genes of 20 out of the 33 MIA gene clusters showed conserved collinearity in the *C. roseus*, *C. acuminata*, and *G. sempervirens* genomes (Fig. 7, Supplementary Figs. 42–48, and Supplementary Data 26). The gene cluster C1693 exhibited conserved synteny for functionally characterized genes encoding *10-HGO*, *ASO/PAS*, and *THAS3* in *C. roseus*, while an adjacent gene cluster, C1684, showed conserved synteny for the functionally characterized *G10H* in *O. pumila* and *C. roseus* (Supplementary Fig. 46). Synteny between *O. pumila* and *C. roseus* or *G. sempervirens* genomes centered around gene clusters was statistically significant based on Fisher’s exact test (*p* value <0.05), suggesting gene clusters as the critical genomic regions for the evolution and expansion of specialized metabolites (Supplementary Data 26). The median Ks for gene clusters in the *O. pumila* genome in synteny with other MIA-producing plant genomes suggests conserved gene content and gene order (Supplementary Data 25 and 26). Tandem duplications within *O. pumila* MIA gene clusters were also statistically significant, with genes encoding *STR*, *SLS*, *7-DLH*, *7-DLGT*, and other MIA-associated genes being duplicated and gained within identified gene clusters. These gene clusters represent the pangenome for MIA biosynthesis and include several functionally characterized genes, as well as potential genes involved in MIA biosynthesis, which also showed a high correlation with nitrogen-containing metabolites identified in the *Ophiorrhiza* metabolome (Fig. 7 and Supplementary Figs. 42–48).

## Discussion

The evolution of a specialized metabolite biosynthetic pathway involves the emergence of enzymes that catalyze committed steps toward synthesizing core metabolites. These core metabolites are then subsequently catalyzed by native coopting enzymes, resulting in the colossal chemodiversity of the plant metabolome^51,52^. The expanded chemodiversity, upon serving as a positive selection force, activates the evolutionary machinery, including the emergence of novel specialized enzymes through gene expansion/neofunctionalization, thus beginning the process of refining the imperfect secondary metabolite biosynthesis pathways^53^. Among Gentianales, the emergence of *STR* for the synthesis of strictosidine was an important innovation to promote the evolution of MIA biosynthesis, which occurred after the whole-genome triplication of core eudicot genomes (Fig. 6e and Supplementary Fig. 41). While STR-like enzyme homologs were identified and assigned across plant species from different lineages (Supplementary Fig. 29a), functional STRs (OG0015245) were specifically identified in the strictosidine-derived-MIA-producing plants (Fig. 6a and Supplementary Fig. 29b). *C. roseus* and *G. sempervirens* genome, which diverged ~68 Mya from *Ophiorrhiza*, consisted of a single copy of the *STR* gene, while the *Ophiorrhiza* genome contained two *STR* orthogenes, resulting from tandem duplication. One of the exceptions to the otherwise highly restricted MIA biosynthesis in Gentianales is camptothecin, which was first identified in *C. acuminata* of Cornales. Similar to the other plant genomes analyzed in this study, *C. acuminata* lost the functional STR enzyme and did not synthesize strictosidine. Instead, *C. acuminata* synthesizes strictosidinic acid for the biosynthesis of MIAs, including camptothecin^42^. For *C. acuminata*, the emergence of a bifunctional *SLS* (OG0013616) was important for the biosynthesis of strictosidinic acid, which incidentally also showed the fastest rate of substitution among all MIA-producing plants (Fig. 6e and Supplementary Fig. 41)^43^. With the WGD peak for *C. acuminata* detected at peak Ks of 0.469 and median Ks of 0.75 for SLS (OG0013616), our results suggest an earlier emergence of key metabolite intermediates prior to the WGD in *C. acuminata*, which then served as a catalyst that allowed post-WGD expansion and evolution of MIA biosynthesis (Supplementary Fig. 41). Synteny analysis between the coffee and *Ophiorrhiza* genomes showed significant genome collinearity, yet one of the key enzymes lost in the coffee genome included functional *STR* orthogene families. The coffee and *Ophiorrhiza* genomes diverged at ~47 Mya, suggesting that while the STR enzyme evolved through SSDs in *Ophiorrhiza*, the coffee genome instead lost the enzyme required for strictosidine synthesis. Comprehensive metabolite profiling of several species from the *Coffea* genus, including wild coffee species, could not detect strictosidine, suggesting the possibility that STR has been lost across different species from the *Coffea* genus^54^. Our study proposes that the retention of STR after the whole-genome triplication event in core eudicots was the critical event that allowed selected plant species to evolve MIAs and expand their chemodiversity (Fig. 6d–f and Supplementary Fig. 41). *C. acuminata*, the exception, instead uses a promiscuous enzyme for the synthesis of strictosidinic acid, which offered similar opportunities for the evolution of MIA biosynthesis. Despite their similar metabolite intermediates and chemotypes, the two parallel paths to the starting point of MIA biosynthesis in *O. pumila* and *C. acuminata*, an estimated speciation time of 120 Mya and their completely different genome dynamics leading to their present-day genomes lead us to propose the possibility of convergent evolution of camptothecin biosynthesis.

As several functional metabolic gene clusters have been reported in the plant genome, identifying and analyzing gene clusters seems to be a promising means to identify candidate genes involved in the biosynthesis of specialized metabolites^55^. Since the number of functionally characterized metabolic gene clusters is still limited in plants, key features such as the extent of coexpression and the degree to which gene localization corresponds to participation within the same pathways are not yet clear. Wisecaver et al., noting that the physical proximity of genes associated with metabolic pathways is statistically significant in *Arabidopsis*, suggested gene coexpression as a key feature for identifying enzymes associated with known specialized metabolic pathways irrespective of the location of their genes in the genomes^49^. Several studies in the past have also reported the selective nature of coexpression of genes in a predicted metabolic gene cluster^39,55–57^. In the *Ophiorrhiza* genome, we also observed a lack of coexpression trends among member genes of a given gene cluster. The scattered nature of metabolic gene clusters seems to be prevalent across plant genomes, as observed in the case of MIA gene clusters, as well as previously reported secondary metabolic gene clusters in other plant species^55,58^. With the complexities associated with the regulation of gene-expression in plants, it is only rational to suggest that physical proximity may not be sufficient to facilitate coexpression among genes within a gene cluster^57^. On the other hand, gene clusters represent discrete genome segments that serve as the hotspots for retaining and evolving specialized metabolite biosynthesis. Benzylisoquinoline alkaloid biosynthesis is one of the best-known specialized metabolic pathways, with enzymes forming gene clusters within the opium poppy genome. Nevertheless, the nature of gene clustering was reported to be heterogeneous, with the thebaine and noscapine pathways being highly clustered, and the morphine and sanguinarine pathways being scattered^56^. These results suggest the possibility of the active evolution of genome architecture through a combination of natural and artificial selection for specialized metabolite biosynthesis centered at gene clusters. The gene clusters, therefore, could be regarded as blocks of secondary metabolite modules whose novel combinations could produce new chemotypes, which may offer unique phenotypes for positive selection. In the process of evolution, plants could lose some members of these modules or the entire module itself, and thus also lose the ability to further evolve or refine a particular phenotype. On the other hand, plant species that could retain the specific module could continue to iterate a particular phenotype to more perfectly adapt to the ecological challenges presented over time. As key mechanisms of evolution and speciation, genome restructuring and dynamics acting on gene clusters seems to provide an opportunity to evolve diverge chemotypes across plant species. In this study, we identified the C1541 gene cluster precisely playing this role in strictosidine-derived MIA-producing plants. This implies selection pressure favoring the clusters of genes involved in the biosynthesis of specialized metabolites and could be a way to identify genes involved in the biosynthesis of common metabolite classes going forward. One possible explanation for the positive selection of gene clusters is the reduced rate of recombination between genes involved in local adaptation^55,59^. Positive selection of gene clusters does have a possible role in providing chemotypes that may facilitate ecological/local success for a species or cultivar for successful propagation, as was reported for opium poppy^56^. The conserved nature and collinearity of metabolic gene clusters of the *Ophiorrhiza* genome across MIA-producing plant species suggest a potential means to select genes for functional studies. The role that gene clusters could play in the divergence of species is not clear, and more high-quality genomes of plants producing specialized metabolites are needed for comprehensive comparative genome analysis to further understand the evolutionary principles that allow a wide distribution of metabolic gene clusters across diverse plant species.

The genome assembly strategy used in this study showed the importance of assembly validation at each step, which should be ignored. Previously, multistage scaffolding was reported as advantageous in assembling the goat genome^20^, and has also been used for other plant genomes^19,21,60^. However, our results showed that the order of scaffolding plays an important role in improved assembly contiguity. One possible explanation for the relevance of the order is the difference in genome resolution for each of the scaffolding technologies. The genome resolution ranges from 30–50 kb for PacBio reads, 150 kb–10 Mb for Bionano optical maps, ~2 Mb for 10× genomics, and 30–100 Mb for Hi-C^61^. Different plant genomes present different challenges, from polyploidy to genome size to repeat content. While we cannot claim that this order of scaffolding technologies will always offer as significant improvement as we observed for the *Ophiorrhiza* genome, our result certainly showed the importance of assembly validation at each stage of assembly. Stepwise scaffolding and error correction refine the assembly at each stage and therefore assist in achieving high assembly contiguity. While scaffold and contig N50 are widely accepted as genome assembly quality parameters, it is the number of assembly gaps that reflects the real sense of completeness and associated potential misassemblies. Furthermore, although we tested and validated the genome assembly at each stage using multiple orthogonal sequencing technologies, experimental analysis still detected an orientational error in the *Ophiorrhiza* genome, which suggests that relying solely on sequencing technologies is not sufficient in the pursuit of an accurate genome assembly. The next generation of plant genomes will need to go beyond the construction of mere scaffolds or pseudomolecules and should include experimental validation elements. With the emergence of high-fidelity long reads from PacBio, and longer and more accurate reads from Nanopore, assembly contiguity and contig N50 are expected to improve significantly, even for highly heterozygous plant species. Nevertheless, validation steps during and after scaffolding are essential for the accurate interpretation of evolutionary and paleogenomics analysis for all future studies. The status quo of the limited number of near-finished and experimentally validated reference plant genomes needs to change. We believe that this study represents the first step forward in that direction.

## Methods

### Plant material and sequencing

For whole-genome sequencing and assembly, we selected *O. pumila* Champ. ex Benth^62^ (Fig. 3a). *O. pumila* plants and hairy roots, grown under the aseptic conditions, have been used as a model to investigate biosynthesis of MIA and camptothecin^3,12,62–64^. Genomic DNA for whole-genome sequencing was obtained from the young leaf tissues of 60-day-old *O. pumila* plant grown in half-strength Murashige and Skoog culture media containing phytoagar (Wako Pure Chemicals, Japan), maintained at 28 °C in 16-h day condition in the plant growth chamber^65^.

Genomic DNA extraction strategies were specific to the sequencing technologies used in this study. For Illumina sequencing, genomic DNA was extracted using Qiagen DNeasy Plant Mini Kit (Qiagen, Hilden, Germany) following the manufacturer’s instructions. Extracted genomic DNA was fragmented to an average size of 600 bp with the DNA Shearing System M220 (Covaris Inc., USA). A sequencing library was constructed using the TruSeq DNA PCR-Free Library Prep kit (Illumina, San Diego, USA) and was size-selected on an agarose gel using the Zymoclean Large Fragment DNA Recovery Kit (Zymo Research, CA, USA). The libraries were sequenced on the Illumina HiSeq2500 sequencer in a paired-end mode with a read length of 250 bp. High molecular weight genomic DNA for PacBio sequencing was extracted using Qiagen Blood and Cell Culture DNA Mini Kit (Qiagen, Hilden, Germany), following the manufacturer’s instructions. Extracted genomic DNA was processed using Qiagen MagAttract HMW DNA Kit (Qiagen, Hilden, Germany) to enrich DNA over 50 Kb for PacBio sequencing.

For Bionano optical maps sequencing, we used Bionano Prep Plant Tissue DNA Isolation Kit (Bionano genomics, CA, USA), following Bionano Prep Plant Tissue DNA Isolation Base Protocol (part # 30068). Briefly, 5 g of fresh young leaves were collected and fixed by formaldehyde treatment followed by homogenization with TissueRuptor (Qiagen, Hilden, Germany). Nuclear fraction was purified with Bionano Prep Plant Tissue DNA Isolation Kit, and the extracted nuclei were embedded in the low-melting agarose plug. Agarose plug was treated with proteinase K (Qiagen, Hilden, Germany) and RNase A (Qiagen, Hilden, Germany) according to the Bionano protocol, and subsequently melted with GELase (Thermo, MA, USA) and dialyzed with TE buffer. Prepared nuclear DNA was labeled using Nb.BssSI (NEB, MA, USA) as restriction enzyme with Bionano Prep Labeling Kit (Bionano genomics, CA, USA), followed by sequencing through Bionano Irys system using one chip, resulting in 101.9 Gb total data (>20 Kb) in the form of 8,73,588 molecules. Hi-C library was prepared using Proximo Hi-C plant kit (Phase Genomics, WA, USA) following the manufacturer’s protocol with slight modification. In the last step for library preparation, we used a gel extraction-based purification approach to select Hi-C library with a fragment size range of 400–600 bps. Sau3AI was used as a restriction enzyme to prepare Hi-C libraries. Hi-C libraries were quantified using the Qubit 3.0 Fluorometer (Thermo Fisher Scientific, USA) according to the manufacturer’s protocol. We prepared two independent Hi-C libraries and sequenced on the Illumina HiSeq2500 sequencer in the paired-end mode with a read length of 100 bp.

### Karyotyping and genome size estimation

For *O. pumila* karyotyping, we fixed small flower buds (<1 mm) without white petals in 3:1 (v/v) ethanol:acetic acid for 24 h and stored in 70% ethanol at 4 °C. The preparation of mitotic/meiotic chromosome slides was performed, as previously described with some modifications (Supplementary Fig. 4a, b)^66^. Briefly, after washing the fixed buds in the distilled water, two anthers were digested with 50 µl of enzyme solution containing 4% cellulase Onozuka RS (Yakult pharmaceutical, Japan) and 2% pectolyase Y-23 (Kyowa chemicals, Japan) at 37 °C for 30 min. The digested anthers were transferred to 20 µl of 60% acetic acid solution. Subsequent smearing steps were similar to the procedure described previously^66^. Experiments were repeated twice, and for each experiment, we used at least ten or more chromosome slides.

The genomic size of *O. pumila* was estimated using the flow cytometer approach, and *k*-mer analysis was performed using KmerGenie software^67^ (Supplementary Fig. 4c). Flow cytometer-based analysis was performed, as previously described^68^. Briefly, young leaves of *O. pumila* and *A. thaliana* were cut using a razor blade in the ice-cold 2-amino-2-(hydroxymethyl)−1,3 propanediol (TRIS)-MgCl_2_ buffer (0.2 M TRIS-HCl, 4 mM MgCl_2_, 0.5% Triton-X 100, pH 7.5), including propidium iodide (50 μg ml^−1^) and ribonuclease (50 μg ml^−1^), and incubated for 5 min. The relative DNA content of isolated nuclei was analyzed using a flow cytometer, FACSCalibur system (Becton Dickinson, New Jersey, USA), while data were acquired and processed by BD FACS DIVA software (v 7.0). No post-fractions were collected, and preliminary FSC/SSC gates for the starting cell population were not used.

### De novo genome assembly with parameters optimization

To derive de novo genome assembly using PacBio sequencing reads, we used two assemblers, Canu (v.1.7)^22^ and Falcon-unzip (v.1.3)^23^. We tested multiple parameters specific to the individual assemblers as our effort to optimize and derive best contig-level genome assembly (Supplementary Data 2 and 3). Under different parameters tested for Canu, the best assembly (called Canu-assembly from here on) was used to derive the reference genome assembly (Table 1 and Supplementary Data 2), while best Falcon-unzip assembly (called Falcon-unzip-assembly from here on) was used to derive phased genome assembly of *O. pumila* (Supplementary Data 3). We used Falcon-unzip-assembly together with Hi-C libraries as the input for the Falcon-Phase software^69^ to achieve a phased contig-level genome assembly of *O. pumila*. Briefly, we minced primary contigs in the form of haplotigs pair and collapsed the haplotypes followed with mapping of paired-end Hi-C reads to obtain normalized contact matrix. The contact matrix was then used to phase the genome into haplotigs along primary contigs. Default parameters were used for Falcon-Phase software, and the resulting contig-level phased haplotigs of *O. pumila* were used to derive completely phased chromosome-level genome assembly.

### De novo genome assembly using Bionano optical map datasets

Acquired Bionano optical maps sequencing datasets were filtered using length cutoff as 150 Kb. We used Canu-assembly as the reference genome to derive guided de novo genome assembly and generated the “.cmap” file. Parameters used for creating “.cmap” files and genome-guided de novo assembly using Bionano optical maps were in accordance with the recommendation of Bionano Solve v 3.0.1 manual (BioNano genomics). Final de novo assembly using Bionano optical maps include 458 scaffolds with N50, and a cumulative assembly length of 1.68 and 442 Mb, and over 83% of optical maps mapped to the Bionano de novo assembly (Table 1).

### Chromosome-level genome assembly through stepwise scaffolding

We adopted a stepwise scaffolding approach to derive the chromosome-scale genome assembly of *O. pumila*. The strategy to derive a high-quality genome assembly included five stages in the order, as described below.

(i) Canu-assembly (or Falcon-unzip-assembly or genome assemblies scaffolded using Hi-C library) was used together with Bionano de novo assembly to derive hybrid scaffolding, using Bionano Solve v 3.0.1 software (BioNano genomics) with default parameters. Hybrid scaffolding detected chimeric sites with conflicts supported by Bionano optical maps and de novo assembly (Supplementary Fig. 3). We inspected conflicts using IrysView software (BioNano genomics), which were manually verified and were used as evidence to split at the sites of misassemblies.
(ii) Canu-Bionano assembly was next split at the gaps and used as input for scaffolding through Hi-C libraries. Hi-C paired-end reads were aligned to the genome assemblies, using BWA (v 0.7.16)^70^ with strict parameters (-n 0). Read pairs that aligned to different contigs were used for scaffolding.
(iii) After splitting assembly at the gaps of Canu-Bionano-assembly, Hi-C-based scaffolding was derived through the Proximo Hi-C scaffolding pipeline (Phase genomics, CA, USA), as described previously^71^. The proximity-guided assembly performed chromosome clustering and determined contig orientations. Briefly, the Proximo Hi-C scaffolding pipeline is based on an enhanced version of LACHESIS algorithm^72^, which additionally performs scaffold optimization and quality control steps based on interaction probabilities to group and orient contigs. Hi-C interactions binned the contigs into 11 groups (corresponding to the haploid or phased chromosomes) and successfully oriented all contigs. The gap size between the ordered contigs was set to 25 bp. Canu-assembly, scaffolded through Bionano followed by Hi-C, was further checked for the presence of any assembly conflicts and was thoroughly verified, using Hi-C reads-based contact matrix, Bionano raw reads, and Bionano de novo assembly (Fig. 3b–l, and Supplementary Figs. 8 and 9).
(iv) The final genome assembly was subjected to PacBio reads-based gap filling, using PBJelly^73^ from PBSuite v15.8.24 with default parameters. PbJelly closed 64 out of the 85 assembly gaps.
(v) We next performed assembly polishing. Firstly, we performed three rounds of assembly polishing by PacBio reads, using arrow software (https://github.com/PacificBiosciences/GenomicConsensus/tree/develop/GenomicConsensus/arrow). Arrow-based polishing was followed by a final round of error correction, using Illumina reads through Pilon software^74^. Illumina sequencing reads were trimmed based on Phred score using Trimmomatic software^75^, mapped to the genome assembly using Bowtie 2.0^76^, and were subsequently used for Pilon software-based error correction.

For in tandem scaffolding, assemblies scaffolded using Bionano datasets or Hi-C were first disintegrated at the assembly gaps and were subsequently used as input for scaffolding by either by Hi-C or Bionano datasets, respectively (Table 1). The final reference and phased genome assemblies of *O. pumila* were validated based on Bionano optical maps, Bionano de novo assembly using IrysView software, and Hi-C contact map using Juicerbox software^77^ (Fig. 2b–l, and Supplementary Figs. 8 and 9). BioNanoAnalyst^78^ software-based assembly quality assessment showed 100% of the assembly supported by Bionano optical maps for *O. pumila* genome assemblies.

### Phasing genome assemblies into haplotigs

We used contig-level genome assemblies, obtained from analyzing Falcon-unzip-assembly with Hi-C reads using Falcon-Phase software, to derive phased genome assemblies of *O. pumila* (Supplementary Data 3 and 4). Each of the contig-level haplotigs was first scaffolded using Bionano de novo assembly followed by Hi-C-based scaffolding. Subsequently, PbJelly-based gap filling and assembly polishing were performed, as described for *O. pumila* reference genome assembly (Supplementary Data 4). Comparing *O. pumila*-phased genomes with the reference genome assembly showed perfect alignment except an assembly gap in chromosome 1 of the haplotigs (Supplementary Fig. 5), which originates from the difference between contig-level assembly, resulting from Falcon-unzip and Canu, the primary assemblers used.

### Experimental validation of genome assembly

We first identified repeats in *O. pumila* genome and masked repeats using RepeatMasker (http://www.repeatmasker.org/RMDownload.html), and fragmented the genome assemblies along chromosomes into 20 or 40 Kb genome segments. We performed BLASTN-based analysis using each fragment as query against all genome fragments together as the database. The genome segments with ≤2% identity were regarded as nonhomologous genomic regions and the repeat-free nonhomologous regions >7 Kb adjacent to the genome assembly gaps were selected as probes. PCR primers were designed using Primer 3 (http://bioinfo.ut.ee/primer3/) (Supplementary Data 6). Touchdown PCR with KOD Plus Neo (TOYOBO, Japan) was conducted as follows: 94 °C for 2 min, five cycles of 94 °C for 15 s and 74 °C for 6 min, five cycles of 94 °C for 15 s and 72 °C for 6 min, five cycles of 94 °C for 15 s and 70 °C for 6 min, and 30 cycles of 94 °C for 15 s and 68 °C for 6 min. Purified PCR products, showing a single DNA band on electrophoresis, were labeled by DIG-Nick Translation Mix (Sigma-Aldrich, MO, USA). For FISH analysis, 10 µl of hybridization solution (50% formamide, 10% dextran sulfate, 2× SSC, 200 ng of each probe) was applied to each chromosome slide, covered with 22 × 22 mm coverslip and sealed with a paper bond (Kokuyo), and the chromosomal DNA and the probe DNA were denatured for 4 min using a heat block (80 °C). The slide was incubated in a moisture chamber at 37 °C for 2 days. After washing in the distilled water, 125 µl of the antibody cocktail (1 % BSA (Roche), 4× SSC, 0.1 µg anti-digoxigenin-rhodamine Fab fragments (Roche)) was applied and covered with parafilm, and incubated in a moisture chamber for 60 min at 37 °C. The chromosome slide was air-dried after washing it three times in 42 °C distilled water each time for 5 min. Finally, we counterstained chromosomes with 5 µl of VECTASHIELD (Vector Laboratories) containing 5 µg ml^−1^of 4, 6 diamidino-2-phenylindole (Thermo Fisher Scientific). FISH signals were captured with an OLYMPUS BX-53 fluorescence microscope equipped with a CCD camera (Photometrics Cool SNAP MYO), and processed by MetaVue/MetaMorph (v.7.8) and Adobe Photoshop CC. Image J (https://imagej.nih.gov/ij/) was used to straighten pachytene chromosomes. For experimentally validating the orientations of contigs within a scaffold, the position of FISH probes was compared with the expected position along the chromosome arms. We also used repeat analysis to identify the pericentromeric region and centromere repeats annotated as putative OpuCEN (Supplementary Fig. 16). *C. arabica*, an allotetraploid genome resulting from hybridization between *C. canephora* and *C. eugenioides*, was recently sequenced^79,80^. We designed FISH probes corresponding to the site of potential misassembly in the *C. canephora* genome and tested in *C. arabica*. FISH analysis for *C. arabica* was performed as has been described for *O. pumila*, and fresh roots were chosen for the experiment. FISH analysis for all probes were performed as two independent experiments, and for each probe, the signals were confirmed for at least ten or more instances.

### Genome assessment

The *O. pumila* genome assembly and phased genome assemblies were benchmarked using BUSCO (v 3.0.2b)^30^. We identified 1335 out of 1375 (97.1%) complete gene models and six fragmented gene models (0.4%) in *O. pumila* reference genome assembly; 95.0% of these complete gene models were single copy, while only 2.1% had more than one copy (Fig. 3c). BUSCO analysis for *O. pumila*-phased genome assemblies (haplotigs) identified 91.2–91.4% of core gene models. We compared predicted gene models with de novo transcriptome assembly derived using RNA-seq datasets for five tissues and previously published datasets of *O. pumila*^3,15,65^. De novo transcriptome assembly was derived using Trinity software (v 2.6.6)^81^. Mapping these unigenes to the *O. pumila* genome using BLAT software^82^ showed 99.32% of the unigenes could be identified, suggesting a good representation of coding sequences in the genome.

### Gene prediction and functional annotation

Gene models for *O. pumila* genome assembly were predicted as described before^83^. Briefly, evidence-based gene prediction was first performed by BRAKER 2 software^84^, using *O. pumila* de novo transcriptome assembly. The predicted gene models together with gene models of *C. canephora* (v 1.0)^29^, *Nicotiana tabacum*^85^, and *Trifolium pratense* (v 2.0)^86^, were used as training sets for the ab initio gene prediction. Gene model prediction was performed using the MAKER-P pipeline (v 2.31.8)^87^ by incorporating three ab initio gene prediction tools, namely, AUGUSTUS (v 3.3)^88^, SNAP (v 2006-07-28)^89^, and GeneMark_ES (version 4.33)^90^. In parallel, we performed an evidence-based gene model prediction by mapping *O. pumila* transcriptome datasets onto the assembled genome sequences, using TopHat (v 2.1.1) and Cufflink (v 2.2.1) pipeline^91^. The predicted gene models were used to perform InterProScan^92^ against the InterPro database and BLAST search against GyDB 2.0^93^ with an E-value cutoff as 1.0. Gene models annotated as TEs based on GyDB 2.0 or InterProScan annotation were excluded for analysis, and regarded as de novo predicted novel TEs of *O. pumila* genome. The remaining gene models were subjected to homology searches against NCBI-nr database, *A. thaliana* in TAIR11, and SwissProt protein databases using BLASTP with an E-value cutoff of 1E−20. The gene models with homology against searched databases or annotation edit distance (AED) score ≤ 0.9 were selected as high-confidence intrinsic gene models, resulting in 32,389 gene models for the *O. pumila* genome in total. The rest of the predicted gene models with no RNA-seq evidence or annotation were categorized as low-quality gene models and were not used for any downstream analysis. Annotated gene models were functionally mapped and annotated using OmicsBox software (BioBam). Annotation-based GO terms assigned to *O. pumila* gene models showed the top six assigned biological processes related to different metabolic processes, including organic substance metabolic process and nitrogen compound metabolic process (Supplementary Fig. 10f). *O. pumila* gene models were also assigned to TFs families based on PlantTFDB classification^94^. The TF database was obtained from PlantTFDB (http://planttfdb.cbi.pku.edu.cn/download.php) and used for reciprocal best hit (RBH), using blast_rbh.py script (https://github.com/peterjc/galaxy_blast/tree/master/tools/blast_rbh). A total of 778 genes were assigned to 54 TF families, including bHLH, MYB, NAC, C2H2, and ERF based on the number of assigned genes (Supplementary Data 7).

*O. pumila* noncoding RNAs were annotated using multiple databases and software packages. The tRNA genes and their secondary structure were identified by tRNAscan-SE software^95^ with default parameters (Supplementary Data 9). The ribosomal RNAs (rRNAs) were predicted based on BLASTN search against rRNA sequences at an E-value cutoff of 1e−10. For microRNAs and small nuclear RNA (snRNA) coding genes prediction, we used INFERNAL software^96^ against the Rfam database (release 13)^97^. In total, we identified 90 miRNAs and 2032 snRNAs in the *O. pumila* genome (Supplementary Data 8).

### De novo transposable elements and repeat annotation

We used known repetitive sequences in Repbase (http://www.girinst.org/repbase/)^98^ and de novo repeat libraries to annotate *O. pumila* repeat contents. For the de novo repeat prediction, we used RepeatModeler (v 1.0.11) (http://www.repeatmasker.org/RepeatModeler/), LTR_FINDER^99^, and RepeatScout 1.0.5^100^. The repetitive elements in the Repbase and *O. pumila* de novo repeat library were annotated using RepeatMasker (v 4.0.7)^101^. Tandem repeats of the *O. pumila* genome were identified using Tandem Repeats Finder (TRF) software^102^. Using TRF, we identified telomere regions for all eleven chromosomes of *O. pumila* (Supplementary Data 11).

### Whole-genome duplication and intergenomic analysis

To understand *O. pumila* genome evolution, we searched for genome-wide duplications in the assembled *O. pumila* genome. We performed the self-alignment of the genome assembly using LAST (v 963)^103^. Using a cscore filter of 0.7, we filtered LAST-run output to identify significantly matching sequences within the *O. pumila* genome. The analysis suggested a minimal genome duplication within the *O. pumila genome*, with 2917 gene sets showing small stretches of duplication in the form of 200 clusters (Supplementary Fig. 10b). MCSCANX^104^ based synteny analysis of *O. pumila* genome with default parameters detected 132 syntenic blocks representing 3227 genes (9.97% of genomic space), while 1351 gene pairs were identified as tandem repeats. Minimal segmental duplication blocks suggested small-scale background duplications rather than a WGD event in *O. pumila*.

We next compared *O. pumila* genome assembly with 12 other plant genomes, namely, *A. trichopoda*^105^*, A. thaliana*^106^*, C. acuminata*^25^*, C. roseus*^6^*, G. sempervirens*^6^*, Helianthus annuus*^107^*, Lupinus angustifolius*^108^*, N. benthamiana*^31^*, P. somniferum*^109^*, S. lycopersicum*^32^, and *V. vinifera*^27^. We identified paralogs by all_vs_all BLASTP search for each of the plant genomes with E-value cutoff as 1e−10, followed by MCL^110^ clustering with inflation factor 1.5. Identified paralog groups with a maximum of 100 genes were selected to perform pairwise sequence alignment using MUSCLE (v 3.8.31)^111^, with the number of times to perform ML estimation set as 5. The synonymous substitution rate (Ks) for paralogous gene pairs were calculated using codeml program of PAML package^34^. The Ks distribution plot for *O. pumila* paralogs showed a typical gamma (γ) event corresponding to whole-genome triplication, but no peak corresponding to a new WGD event was identified (Fig. 3e and Supplementary Fig. 19). Similar to *O. pumila*, no WGD was detected in *C. roseus*. For *C. acuminata*, Ks distribution plot clearly showed two peaks at Ks value 1.681, representing eudicot whole-genome triplication, and 0.469, representing a recent whole-genome duplication (Supplementary Fig. 19). Using whole-genome triplication time as ~154 Mya and Ks-peak as 1.681, we estimated substitution rate as 5.547 × 10^−9^ mutations per site per year (*r*) for *C. acuminata*. Thus, the whole-genome duplication time for *C. acuminata* was dated as 42.27 ± 0.73 Mya using the formula *T* = Ks/2*r* (where standard deviation was calculated based on Ks standard deviation of *C. acuminata* paralogs centered around the Ks median value; Supplementary Figs. 18 and 19). We next compared the *O. pumila* genome with 12 plant species by first identifying RBH using blast_rbh.py script between *O. pumila* and other plant species, and then performing pairwise sequence alignment using MUSCLE (v 3.8.31)^111^ with the number of times to perform ML estimation set as 5. The Ks values for RBHs of *O. pumila* with other plant species were calculated using the codeml program of the PAML package^34^ (Supplementary Fig. 20).

### Phylogenetic tree reconstruction and divergence time prediction

The assembled genome of *O. pumila* allowed us to understand its evolution and to estimate divergence time within Rubiaceae species. In order to achieve a robust phylogenetic reconstruction with high confidence and concordance, we used gene models for 33 plant species; namely, *A. trichopoda*^105^*, Aquilegia coerulea*^112^*, A. thaliana*^106^*, Brachypodium stacei* (*B. stacei v 1.1 DOE-JGI*, http://phytozome.jgi.doe.gov/)*, Brassica rapa*^113^*, C. acuminata*^25^*, C. roseus*^6^*, Cicer arietinum*^114^*, Citrus clementina*^115^*, C. canephora*^29^*, Cucumis sativus*^116^*, G. sempervirens*^6^*, Glycine max*^117^*, Glycyrrhiza uralensis*^118^*, Gossypium raimondii*^119^*, H. annuus*^107^*, L. angustifolius*^108^*, Malus domestica*^120^*, Medicago truncatula*^121^*, Musa acuminata*^122^*, Nelumbo nucifera*^123^*, N. benthamiana*^31^*, O. pumila* (this study)*, Oryza sativa*^124^*, P. somniferum*^109^*, Populus trichocarpa*^125^*, Prunus persica*^126^*, Selaginella moellendorffii*^127^*, S. lycopersicum*^32^*, Sorghum bicolor*^127^*, Theobroma cacao*^128^*, V. vinifera*^27^, and *Zea mays*^129^; thus covering diverse plant lineages. Using OrthoFinder (v 2.3.1)^33^, we identified 31 single-copy orthologous genes from these selected plant genomes. Single-copy genes for each plant species for a given orthogroup were aligned using MUSCLE (v 3.8.31)^111^, and alignments were concatenated to create a super alignment matrix. The concatenated alignment was subsequently used to construct a maximum likelihood phylogenetic tree using RAxML (v 8.2.11)^130^. The concatenated alignment was subsequently used to construct a maximum likelihood phylogenetic tree using RAxML (v 8.2.11).

The derived phylogenetic tree was used to infer divergence time using the MCMCtree program^34,131^ implemented in the phylogenetic analysis by maximum likelihood, using *S. moellendorffii* as an outgroup. The MCMCtree analysis was executed using the following parameters: burn-in-10,000, sample-frequency-2, sample number-100,000. For the divergence time estimation, we calibrated the model using divergence time between *O. sativa* and *A. thaliana* (148–173 Mya), *B. stacei* and *O. sativa* (42–52 Mya), *A. thaliana* and *V. vinifera* (105–115 Mya), and *S. moellendorffii* and *A. trichopoda* (410–468 Mya), obtained from the TimeTree database^132^. The time of speciation between *C. canephora* and *O. pumila* was estimated as 47 Mya (Supplementary Fig. 18). Using the divergence time and Ks-peak between *C. canephora* and *O. pumila*, we estimated synonymous substitutions per site per year (*r*) for Rubiaceae as 6.54e−9 (*T* = Ks/2*r*).

### Expansion and contraction of gene families

Protein sequences of *O. pumila* and 12 other plant species, namely, *A. trichopoda, A. thaliana, C. acuminata, C. roseus, C. canephora, G. sempervirens, H. annuus, L. angustifolius, N. benthamiana, P. somniferum, S. lycopersicum*, and *V. vinifera* were used for the gene family construction. We filtered sequences of length <30 amino acids and performed CD-HIT-EST^133^-based protein clustering to select the longest sequences from the cluster of highly similar sequences for a species. Protein sequences for all the plant species were used as input and were grouped in orthogenes families using Orthofinder (v 2.3.1)^33^ using the following parameters: -S blast -t 70 –M msa –A muscle –T raxml-ng –I 1.5. In total, 18,226 orthogroups were assigned across 13 plant species, including 675 single-copy genes, with 23,229 genes of *O. pumila* being assigned to the orthogroups (Supplementary Data 20). We identified 95 orthogroups representing 632 genes specific to *O. pumila*, while 514 orthogene families were specific to MIAs producing plant species (*O. pumila*, *C. acuminata, G. sempervirens*, and *C. roseus*) with 1,078 genes from *O. pumila* genome and 2,885 genes across the MIA-producing plant species.

We estimated orthogene family gain, expansion, loss, and contraction by comparing the cluster size between species, using COUNT software (Fig. 5)^134^. We first derived the maximum likelihood phylogenetic tree using 675 single-copy orthologs across 13 plant species used for the gene family construction, as described above. The definition of orthogene family evolution in terms of gains, expansions, losses, or contractions are described, using a posterior probability in the COUNT software user’s guide (http://www.iro.umontreal.ca/~csuros/gene_content/count-usage.pdf). COUNT software reconstructs ancestral state using orthogenes family count data and phylogeny relationships and compares the closest outgroup. It uses phylogenetic birth-and-death models for the probabilistic inferences, where rates are optimized using the selected orthogenes family size. For the rate optimization, we used the gain-loss-duplication model type with Poisson distribution for the family size at the root of the phylogenetic tree, and the same gain-loss and duplication-loss ratio was selected as a lineage-specific variation. The maximum number of optimizations rounds for COUNT software was set as 10,000, and the convergence threshold of the likelihood was set as 0.1. Optimized rates for the evolution of orthogenes families were used to calculate family history by posterior probabilities, which provided the corresponding *p* values in each lineage (Supplementary Data 21). A *p* value of 0.05 was used to identify gene families gained/expanded/lost/contracted at a specific lineage or species. Orthogroups specifically gained or expanded in plants producing MIAs were further investigated centered around *O. pumila*.

### Isotope labeling-based approach to identify nitrogen-containing metabolites

Using a ten-step cheminformatics workflow, we performed nitrogen number determination, chemical formula prediction, and structure elucidation for unknown metabolites based on complete nitrogen labeling and liquid chromatography–tandem mass spectrometry (LC–MS/MS) approach, as previously described^7,40^. We performed complete stable isotope nitrogen labeling (^15^N) for *O. pumila* metabolome pool using hairy roots to capture diverse nitrogen-containing specialized metabolites (i.e., MIAs). *O. pumila* hairy roots were maintained as previously described^65^. For complete nitrogen labeling, we replaced KNO_3_ and (NH_4_)_2_SO_4_ with complete nitrogen-labeled substitutes (^15^N) in the growth media, while control samples were maintained in non-labeled nitrogen sources (Supplementary Data 13). Two generations of isotope labeling were used to dilute any carryover from the starting material and to achieve a high degree of ^15^N labeling. Hairy root, post isotope labeling, was used for metabolites extraction and profiling using LC–Q-TOF/MS (LC, Waters Acquity UPLC System; MS, Waters Xevo G2 Q-TOF). The method used for metabolite extraction, purification, and metabolite profiling, including UHPLC conditions, the column used, and MS and MS/MS conditions, have been previously described^40^. Metabolite profiling datasets were analyzed to evaluate the extent of nitrogen labeling of the metabolome space. Principal component analysis clustered samples as two clear groups based on control or nitrogen labeling (Supplementary Fig. 21). We next selected metabolite features distinguishing isotope-labeled vs non-isotope-labeled samples using loadings plot and S-plot. For these selected metabolite features, chemical structures and the number of nitrogen atoms were identified using cheminformatics approach previously described^40,135^. Identified metabolites were manually validated based on mass shift expected due to stable isotope labeling of ^15^N, as well as to exclude any metabolites that were assigned structures due to contamination through in-source fragmentation. Identified metabolites were next compared to previously reported metabolome data for 11 other plant species (Fig. 4)^40^. We also performed metabolite profiling for the same five tissues and *O. pumila* hairy roots that were used to perform transcriptome profiling (Supplementary Figs. 22 and 23, and Supplementary Data 15). Metabolite profiling for isotope-labeled hairy roots, and tissues of *O. pumila* was performed using five biological replicates, and a newly established metabolome database for *O. pumila* was used to assign chemical identity for metabolites and to perform differential metabolome analysis.

### RNA preparation and expression analysis

To facilitate gene model prediction and to capture diverse genes expression, we extracted RNA from five tissues of *O. pumila*, namely, root, stem, shoot-apex, internode, and leaf. Total RNAs from these tissues were extracted using RNeasy Plant Mini Kit (Qiagen, USA) following the manufacturer’s protocol. The RNA quality was assessed using Agilent Bioanalyzer 2100 (Agilent Technology, USA), and RNA samples with RNA integrity number (RIN) >8.0 were used for cDNA library preparation. Illumina libraries for RNA sequencing were prepared, as previously described^136^. cDNA libraries for Illumina sequencing were prepared using the SureSelect Strand-specific RNA library kit (Agilent Technology, USA) according to the manufacturer’s instructions, and sequenced using Illumina HiSeq 2000 sequencer (Illumina, San Diego, USA) to obtain paired-end reads with an average length of 101 bp and a total of 10 Gb sequencing reads for each of the five tissues. For gene model prediction, we used RNA-seq datasets for five tissues generated in this study, and datasets previously acquired from our laboratory deposited under DDBJ accession nos.—DRA000930, DRA000931, and SRA492327. Transcriptome profiling datasets for hairy roots and cell suspension culture of *O. pumila* were obtained from DDBJ (accession no. DRA000930). RNA-seq raw reads were processed to remove adapters and poor-quality reads (base quality score >30) using Trimommatic software (v 0.38)^75^. The processed reads were used as an input for Trinity software (v 2.6.6)^137^ to derive de novo transcriptome assembly, which was then used as EST evidence to validate *O. pumila* gene model prediction. For expression analysis, we used Tophat2 software (v 2.1.1)^91^ to map clean RNA-seq reads to *O. pumila* reference genome with the following parameters: –max-intron-length 500000, –read-gap-length 10, –read-edit-dis 15, –max-insertion-length 5; –max-deletion length 5. The expression level for *O. pumila* genes (RPKM, TPM, and expression count data) was obtained using HTSeq software (v 0.11.1)^138^.

We adopted a targeted approach to identify high-confidence candidate genes associated with MIAs biosynthetic pathway. We first manually curated genes that have been functionally characterized to be associated with MIAs biosynthesis pathways, accounting for 94 genes in total (Supplementary Data 16). Protein sequences for these genes were used as a database, and *O. pumila* gene models were annotated through this database using BLASTP with E-value cutoff as 1E−20 and an alignment length over 100 amino acids. In total, 1226 *O. pumila* genes were annotated as putative genes sharing high sequence similarity with functionally characterized genes (Supplementary Data 17). We next performed CD-HIT-EST-based protein sequence clustering using all *O. pumila* gene models and 94 genes from MIAs protein database. Protein clusters that included *O. pumila* genes together with functionally characterized MIAs genes were selected. CD-HIT-EST-based protein clustering and BLASTP-based annotation were used to select high-confidence genes associated with MIAs biosynthesis in *O. pumila*. We selected 216 *O. pumila* genes representing 40 known enzymes involved in the biosynthesis of MIAs (Supplementary Data 18). These genes were used as criteria to identify MIAs gene clusters.

We next performed coexpression analysis using genes assigned to the secoiridoid biosynthesis branch of MIAs biosynthesis pathways. Coexpression analysis, using Spearman’s correlation followed by hierarchical clustering, identified a highly coexpressed gene cluster representing the complete secoiridoid biosynthetic pathway, including all four functionally characterized genes of *O. pumila* associated with MIA biosynthesis (Supplementary Fig. 24). These genes were used to perform coexpression analysis with other *O. pumila* genes assigned to MIAs biosynthetic pathway, and a highly coexpressed gene cluster was selected (Supplementary Figs. 1 and 25). Heat maps to visualize the expression of assigned MIAs biosynthesis genes were drawn using a heatmap2.0 package^139^ in R (v 3.5.3), while coexpression analysis and hierarchical clustering was performed using in-built functions in R. For integrative omics analysis and gene-metabolite correlation network, we used normalized expression dataset (in the form of transcript per million) for genes assigned to secoiridoids and MIA biosynthesis pathways with metabolome datasets (normalized using internal standard), and performed pairwise Pearson’s correlation analysis using in-built psych package (https://cran.r-project.org/web/packages/psych/index.html) in R. Edges between genes and metabolites are drawn using Cytoscape v 3.6.1, when the correlation coefficient between genes and metabolites are >0.7 with a corrected *p* value < 0.05.

### Metabolic gene cluster prediction

For gene cluster analysis, we used PlantClusterFinder software (v 1.3) pipeline^140^. We used *O. pumila* gene models to assign four-part EC numbers and MetaCyc reaction identifiers based on protein sequence data, and classification according to the predicted catalytic functions by E2P2 software (v 3.1)^141^. E2P2-based enzyme annotation assigned 9584 *O. pumila* genes with an EC number, which were then converted into the corresponding MetaCyc (v 22.5)^142^ reaction identifiers. It was used for pathway inference and pathway database construction using the PathoLogic software (v 22.5)^143^ (Pathway Tool software). The derived pathways database was then manually curated and validated, using SAVI software (v 3.0.2)^140^ to remove any false positive and redundant pathways, such as non-plant pathway variants, and pathways already included as part of a larger pathway. The pathway database for *O. pumila* with assigned metabolic reactions and enzymes were then used as input together with *O. pumila* genome annotation structure for the PlantClusterFinder software, as instructed by the tool developers.

Using the PlantClusterFinder pipeline, we identified 358 gene clusters representing 3387 genes of the *O. pumila* genome (Supplementary Data 23 and 24). To assign gene clusters to MIA biosynthesis, we mapped *O. pumila* genome annotation using MIAs protein database and considered a gene cluster as an MIA gene cluster if it included one or more of the 216 high-confidence MIAs biosynthesis genes. We identified 33 MIA gene clusters across 8 out of the 11 chromosomes (Fig. 7 and Supplementary Figs. 42–48). The obtained gene clusters were mapped with synteny data to compare the *O. pumila* genome with three other MIAs producing plants, namely, *C. acuminata, C. roseus*, and *G. sempervirens* (Fig. 7, Supplementary Figs. 42–48, and Supplementary Data 23–26). Despite the fragmented genome assemblies for other MIA-producing plants, synteny analysis showed conserved gene order centered at *O. pumila* MIAs gene clusters to be statistically significant based on one-sided Fisher exact test.

### Divergence time estimation of orthogene families and syntenic metabolic gene clusters

The phylogenomic analysis was performed to estimate divergence time for syntenic genes that were part of secondary metabolite gene clusters. We first selected syntenic gene pairs between *O. pumila* and other three MIAs producing plants, namely, *C. acuminata*, *C. roseus*, and *G. sempervirens*, as well as *C. canephora*, another plant from Rubiaceae family. Syntenic gene pairs, including a member of one of the 358 metabolic gene clusters identified in the *O. pumila* genome, were selected for further analysis. For each pair, which is also a member of a metabolic gene cluster in the *O. pumila* genome, protein sequence alignment for corresponding syntenic genes from the selected plant genomes was performed using MAFFT^144^, and Ks values were calculated as described above (Supplementary Data 25 and 26). Median Ks value for a gene cluster block was estimated, and was considered as Ks value for that specific gene cluster with respect to the plant species that were used for comparison. We then used one-sided Fisher’s exact test to calculate statistical significance for divergence time of MIA gene clusters, using syntenic analysis data of the whole genome of *O. pumila* as a reference set. For Ks analysis of genes assigned to a given orthogene family, we performed pairwise alignment for paralogs of a given plant species for an orthogene ID, and Ks values for pairwise alignment were estimated, as described above (Fig. 6e, Supplementary Fig. 41, and Supplementary Data 22). For the calculation of Ks median, we discarded values with alignment length <300 bp and alignment coverage <0.2.

We used tools from the ete3 pipeline^145^ to generate maximum likelihood phylogenetic trees for orthogene families using MUSCLE as aligner, trimal_gappyout as alignment cleaner, pmodeltest_soft_slow as model tester, and raxml:default_bootstrap as tree builder (Supplementary Figs. 30–40). We also performed BUSTED analysis^48^ to ascertain if the STR or SLS clade, including orthogenes, gained only in MIA-producing species, as well as conserved across other plant species, has experienced positive selection for at least one site and at least one branch, with *p* level cutoff set as 0.05 for significance (Fig. 6b, d). For BUSTED analysis, we used orthogroups specific to MIA-producing plants as a query, and rest of the genes in the phylogenetic tree were used as the background.

### Reporting summary

Further information on research design is available in the Nature Research Reporting Summary linked to this article.

## Supplementary information

Supplementary Information

Peer Review File

Reporting Summary

Description of Additional Supplementary Files

Supplementary Data 1-26

## Data Availability

The datasets and plant materials generated and analyzed during the current study are available from the corresponding author upon request, and subjected to the material transfer agreement. All raw genome sequencing datasets have been deposited at the DDBJ database (Experiment: DRX185163–DRX185191; Run: DRR194711–DRR194739) under the accession id—DRP006713; with BioProject id—PRJDB8685, submission id—DRA009076. Assembled *O. pumila* genome assembly has been deposited at the DDBJ database (accession ids—BLIW01000001–BLIW01000013). All sequence datasets, assembled genome sequences, predicted gene models, transcriptome datasets, genome browser, annotation, and KEGG mapping results are available to download and analyze through a dedicated server to perform comparative genome analysis (http://pumila.kazusa.or.jp/). RNA-seq datasets have been deposited under submission accession id DRA011108 (https://www.ncbi.nlm.nih.gov//sra/?term=DRP006713/rna)under the experiment accessions DRX245380–DRX245384, and sample accessions DRS163600–DRS163604. For gene model prediction, we also used RNA-seq datasets previously acquired from our laboratory and deposited under DDBJ accession nos.—DRA000930, DRA000931, and SRA492327. Transcriptome profiling datasets for hairy roots and cell suspension culture of *O. pumila* were obtained from DDBJ (accession no. DRA000930). Data supporting the findings of this work are available within the paper and its Supplementary Information files. All results and generated datasets, including comparative genome analysis, transcriptome analysis, metabolome analysis, and integrative omics analysis are available in the form of supplementary datasets. A reporting summary for this article is available as a Supplementary Information file. Source data are provided with this paper.

## Source data

Source Data

## Code availability

Codes for hybrid genome assembly, and the RScript used to create Fig. 4 together with the comparative metabolome dataset and associated metabo-ontologies are available through GitHub (10.5281/zenodo.4279079)^146^. Parameters used for genome assembly, annotation, comparative genome analysis, and metabolome analysis have been provided in detailed Supplementary Methods and Supplementary Tables in the relevant sections.
