# Supplementary material for: Chromosome-level genome assembly of Ophiorrhiza pumila reveals the evolution of camptothecin biosynthesis
Source: Nat Commun. 2021 Jan 15;12:405. doi: 10.1038/s41467-020-20508-2 (PMC7810986; doi:10.1038/s41467-020-20508-2)
Supplement: Supplementary file 1 — Supplementary Information [file 41467_2020_20508_MOESM1_ESM.pdf]

Supplementary Materials for

Chromosome-level genome assembly of *Ophiorrhiza pumila* reveals the evolution of camptothecin biosynthesis

Authors: Amit Rai[1,2*$], Hideki Hirakawa[3], Ryo Nakabayashi[4], Shinji Kikuchi[2,5], Koki Hayashi[1], Megha Rai[1], Hiroshi Tsugawa[4,6], Taiki Nakaya[1], Tetsuya Mori[4], Hideki Nagasaki[3], Runa Fukushi[5], Yoko Kusuya[7], Hiroki Takahashi[2,7], Hiroshi Uchiyama[8], Atsushi Toyoda[9], Shoko Hikosaka[2,5], Eiji Goto[2,5], Kazuki Saito[1,2,4] and Mami Yamazaki[1,2*]

*Correspondence to: mamiy@faculty.chiba-u.jp (M.Y); amit.rai@chiba-u.jp (A.R)

Supplementary Table 1. Karyotype measurements for *Ophiorrhiza pumila* chromosomes.

Chromosome	Short arm	Long arm	Total length	Relative length of chromosome (%)	Total length (Mb)	Relative length of sequence (%)
1	08.4 ± 1.2	13.8 ± 4.8	22.2 ± 5.1	8.4	37.1	8.4
2	10.6 ± 1.5	13.7 ± 3.8	24.3 ± 5.2	9.1	42.3	9.6
3	09.6 ± 4.2	13.9 ± 2.1	23.5 ± 6.3	8.8	30.5	6.9
4	05.7 ± 1.5	14.9 ± 0.9	20.5 ± 2.1	7.7	32.8	7.5
5	07.7 ± 1.3	12.5 ± 1.6	20.2 ± 2.3	7.6	32.3	7.3
6	15.2 ± 1.4	17.3 ± 1.9	32.5 ± 3.0	12.2	60	13.6
7	10.5 ± 0.5	13.3 ± 1.5	23.8 ± 1.5	9	40.7	9.3
8	12.2 ± 2.7	14.2 ± 2.5	26.4 ± 4.9	9.9	39	8.9
9	12.3 ± 0.8	13.0 ± 1.7	25.3 ± 2.5	9.5	51.8	11.8
10	08.5 ± 2.9	16.1 ± 4.4	24.6 ± 6.9	9.2	32.7	7.4
11	09.1 ± 2.7	13.6 ± 2.4	22.7 ± 4.9	8.5	40.6	9.2

Three pachytenes were used for measurements.
The relative length of chromosome (%) is in the percentage of the karyotype length.
Total length (Mb) is the length of assembled sequences for each chromosome.
The relative length of the sequence (%) is calculated using the assembled genome size.

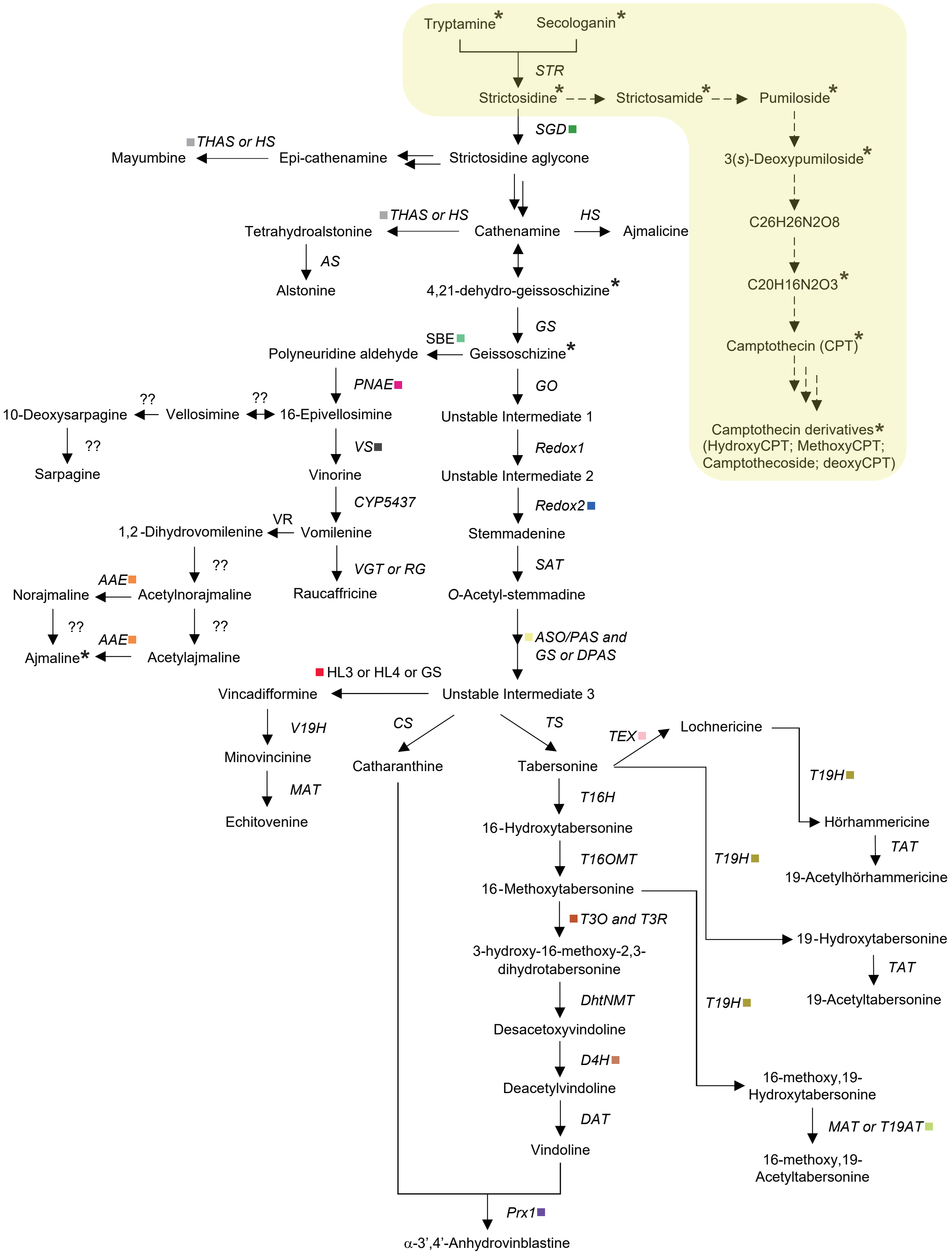

Tryptamine*
Secologanin*
STR
Strictosidine*
Strictosamide*
Pumiloside*
3(s)-Deoxypumiloside*
C26H26N2O8
C20H16N2O3*
Camptothecin (CPT)*
Camptothecin derivatives*
(HydroxyCPT; MethoxyCPT; Camptothecoside; deoxyCPT)
SGD
THAS or HS
Mayumbine
Epi-cathenamine
Strictosidine aglycone
THAS or HS
Tetrahydroalstonine
Cathenamine
HS
Ajmalicine
AS
Alstonine
4,21-dehydro-geissoschizine*
GS
SBE
Polyneuridine aldehyde
Geissoschizine*
PNAE
GO
??
??
10-Deoxysarpagine
Vellosimine
16-Epivellosimine
Unstable Intermediate 1
??
VS
Redox1
Sarpagine
Vinorine
Unstable Intermediate 2
CYP5437
Redox2
VR
1,2-Dihydrovomilenine
Vomilenine
Stemmadenine
??
VGT or RG
SAT
AAE
Norajmaline
Acetylnorajmaline
Raucaffricine
O-Acetyl-stemmadine
??
??
AAE
ASO/PAS and GS or DPAS
Ajmaline*
Acetylajmaline
HL3 or HL4 or GS
Vincadifformine
Unstable Intermediate 3
V19H
CS
TS
TEX
Lochnericine
Minovincinine
Catharanthine
Tabersonine
T19H
MAT
T16H
Echitovenine
Hörhammericine
16-Hydroxytabersonine
TAT
T16OMT
T19H
19-Acetylhörhammericine
16-Methoxytabersonine
T3O and T3R
19-Hydroxytabersonine
3-hydroxy-16-methoxy-2,3-dihydrotabersonine
TAT
DhtNMT
T19H
19-Acetyltabersonine
Desacetoxyvindoline
D4H
16-methoxy,19-Hydroxytabersonine
Deacetylvindoline
DAT
MAT or T19AT
Vindoline
16-methoxy,19-Acetyltabersonine
Prx1
α-3',4'-Anhydrovinblastine

Supplementary Fig. 1. Strictosidine derived monoterpene indole alkaloid (MIA) biosynthetic pathways. Color codes represent specific enzymes assigned to *Ophiorrhiza pumila* genome. Metabolite names with * were chemically assigned and identified in metabolite profiling datasets of *O. pumila* in this study. The yellow shaded branch pathway represents camptothecin biosynthesis pathway. Abbreviations- *STR*: strictosidine synthase; *SGD*: strictosidine-β-D-glucosidase; *THAS*: tetrahydroalstonine synthase; *HS*: heteroyohimbine synthase; *AS*: alstonine synthase; *GS*: geissoschizine synthase; *SBE*: sarpagan bridge enzyme; *GO*: geissoschizine oxidase; *PNAE*: polyneuridine-aldehyde esterase; *VS*: vinorine synthase; *CYP5437*: vinorine hydroxylase; *VR*: vomilenine reductase; *SAT*: stemmadenine *O*-acetyltransferase; *RG*: raucaffricine-β-D-glucosidase; *AAE*: acetylajmaline esterase; *ASO/PAS*: *O*-acetylstemmadenine oxidase; *DPAS*: dihydroprecondylocarpine synthase; *HL3*: hydrolase 3; *HL4*: hydrolase 4; *CS*: catharanthine synthase; *TS*: tabersonine synthase; *V19H*: vincadifformine-19-hydroxylase; *MAT*: minovincinine-*O*-acetyltransferase; *TEX*: tabersonine-6,7-epoxidase; *T16H*: tabersonine-16-hydroxylase; *T19H*: tabersonine-19-hydroxylase; *T16OMT*: tabersonine-16-*O*-methyltransferase; *T3O*: tabersonine-3-oxygenase; *T3R*: tabersonine-3-reductase; *TAT*: 19-hydroxytabersonine,19-*O*-acetyltransferase; *DhtNMT*: 3-hydroxy-16-methoxy-2,3-dihydrotabersonine-N-methyltransferase; *D4H*: deacetoxyvindoline-4-hydroxylase; *DAT*: deacetylvindoline-*O*-acetyltransferase; *T19AT*: tabersonine-19-hydroxy-*O*-acetyltransferase; *Prx1*: peroxidase 1

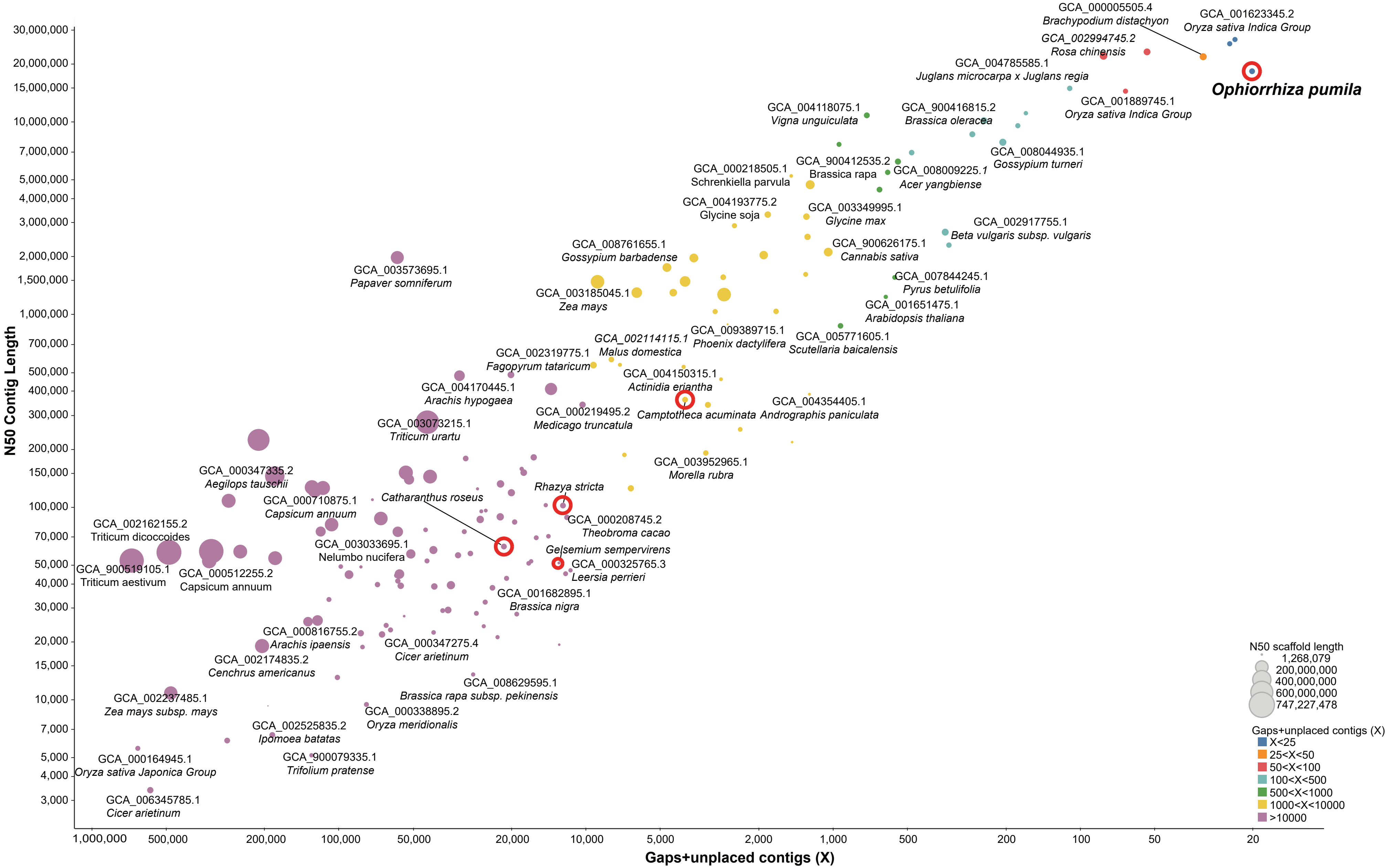
N50 Contig Length
Gaps+unplaced contigs (X)
30,000,000
20,000,000
15,000,000
10,000,000
7,000,000
5,000,000
4,000,000
3,000,000
2,000,000
1,500,000
1,000,000
700,000
500,000
400,000
300,000
200,000
150,000
100,000
70,000
50,000
40,000
30,000
20,000
15,000
10,000
7,000
5,000
4,000
3,000
1,000,000
500,000
200,000
100,000
50,000
20,000
10,000
5,000
2,000
1,000
500
200
100
50
20
GCA_000005505.4
Brachypodium distachyon
GCA_001623345.2
Oryza sativa Indica Group
GCA_002994745.2
Rosa chinensis
GCA_004785585.1
Juglans microcarpa x Juglans regia
Ophiorrhiza pumila
GCA_001889745.1
Oryza sativa Indica Group
GCA_004118075.1
Vigna unguiculata
GCA_900416815.2
Brassica oleracea
GCA_008044935.1
Gossypium turneri
GCA_000218505.1
Schrenkiella parvula
GCA_900412535.2
Brassica rapa
GCA_008009225.1
Acer yangbiense
GCA_004193775.2
Glycine soja
GCA_003349995.1
Glycine max
GCA_002917755.1
Beta vulgaris subsp. vulgaris
GCA_008761655.1
Gossypium barbadense
GCA_900626175.1
Cannabis sativa
GCA_003573695.1
Papaver somniferum
GCA_007844245.1
Pyrus betulifolia
GCA_003185045.1
Zea mays
GCA_001651475.1
Arabidopsis thaliana
GCA_002114115.1
Malus domestica
GCA_009389715.1
Phoenix dactylifera
GCA_005771605.1
Scutellaria baicalensis
GCA_002319775.1
Fagopyrum tataricum
GCA_004150315.1
Actinidia eriantha
GCA_004170445.1
Arachis hypogaea
GCA_000219495.2
Medicago truncatula
Camptotheca acuminata
GCA_004354405.1
Andrographis paniculata
GCA_003073215.1
Triticum urartu
GCA_003952965.1
Morella rubra
GCA_000347335.2
Aegilops tauschii
Rhazya stricta
Catharanthus roseus
GCA_000710875.1
Capsicum annuum
GCA_000208745.2
Theobroma cacao
GCA_002162155.2
Triticum dicoccoides
GCA_003033695.1
Nelumbo nucifera
Gelsemium sempervirens
GCA_000325765.3
Leersia perrieri
GCA_900519105.1
Triticum aestivum
GCA_000512255.2
Capsicum annuum
GCA_001682895.1
Brassica nigra
GCA_000816755.2
Arachis ipaensis
GCA_000347275.4
Cicer arietinum
GCA_002174835.2
Cenchrus americanus
GCA_008629595.1
Brassica rapa subsp. pekinensis
GCA_002237485.1
Zea mays subsp. mays
GCA_000338895.2
Oryza meridionalis
GCA_002525835.2
Ipomoea batatas
GCA_000164945.1
Oryza sativa Japonica Group
GCA_900079335.1
Trifolium pratense
GCA_006345785.1
Cicer arietinum
N50 scaffold length
1,268,079
200,000,000
400,000,000
600,000,000
747,227,478
Gaps+unplaced contigs (X)
X<25
25<X<50
50<X<100
100<X<500
500<X<1000
1000<X<10000
>10000

Supplementary Fig. 2. Assembly statistics for chromosome-scale plant genome assemblies deposited at the NCBI genome database. Chromosome-scale plant genome assemblies being published and deposited in NCBI genome database are included here, while *Ophiorrhiza pumila* genome was assembled in this study. All publicly available genome stats of monoterpene indole alkaloids producing plants were highlighted through red circle.

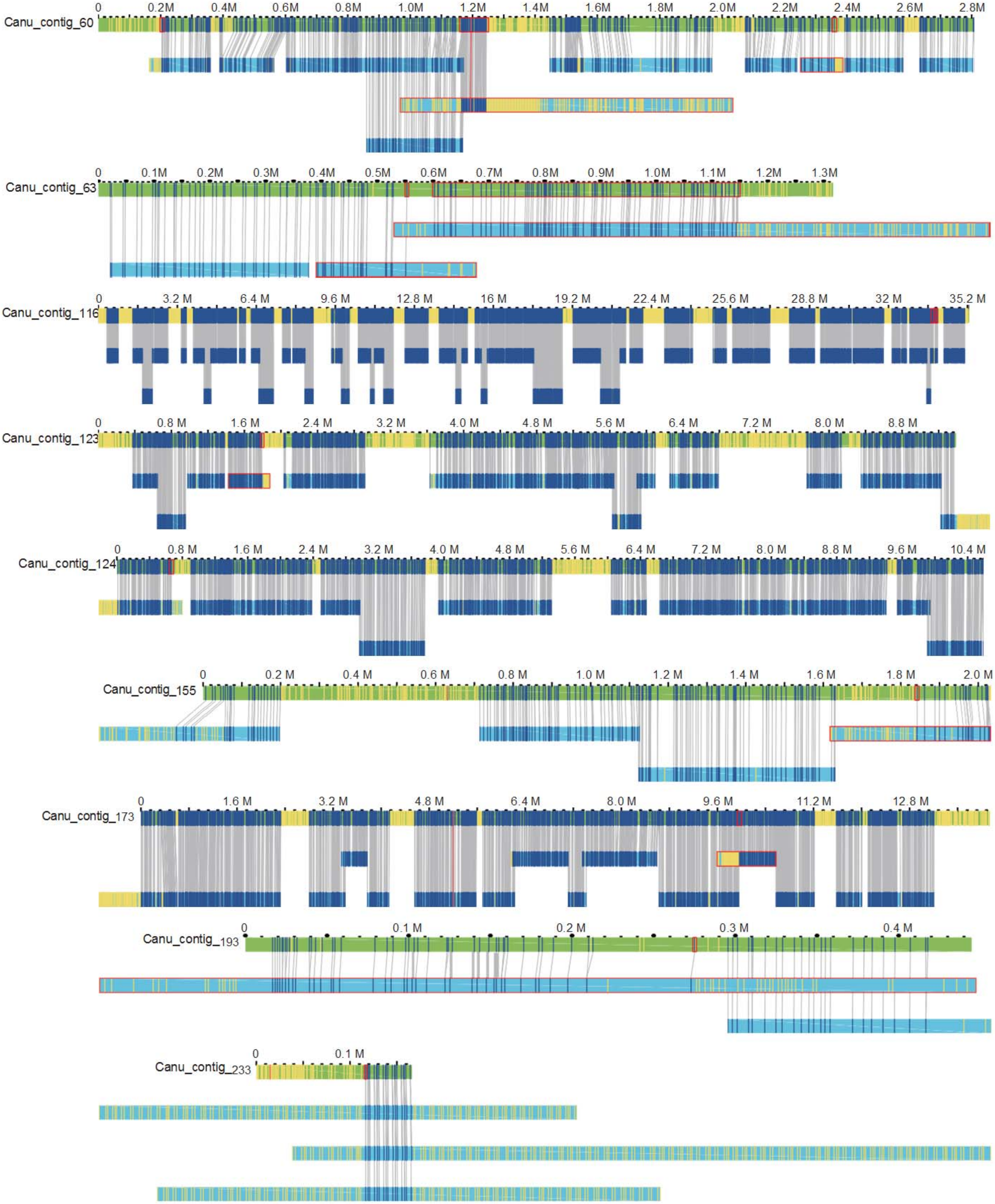

Supplementary Fig. 3. Contig-level genome assembly validation using Bionano de novo genome assembly for *Ophiorrhiza* genome. The Canu PacBio contigs are shown as green bar, Bionano de novo contigs are shown as light blue (turquoise) bar. The regions, on both of the NGS and Bionano assemblies, with consensus are shown as dark blue shades on the bar connected through grey lines, the regions with no support either for NGS assembly or Bionano assembly are shown through yellow lines, and the regions which has conflicts based on NGS assembly and Bionano alignments are shown through red box.

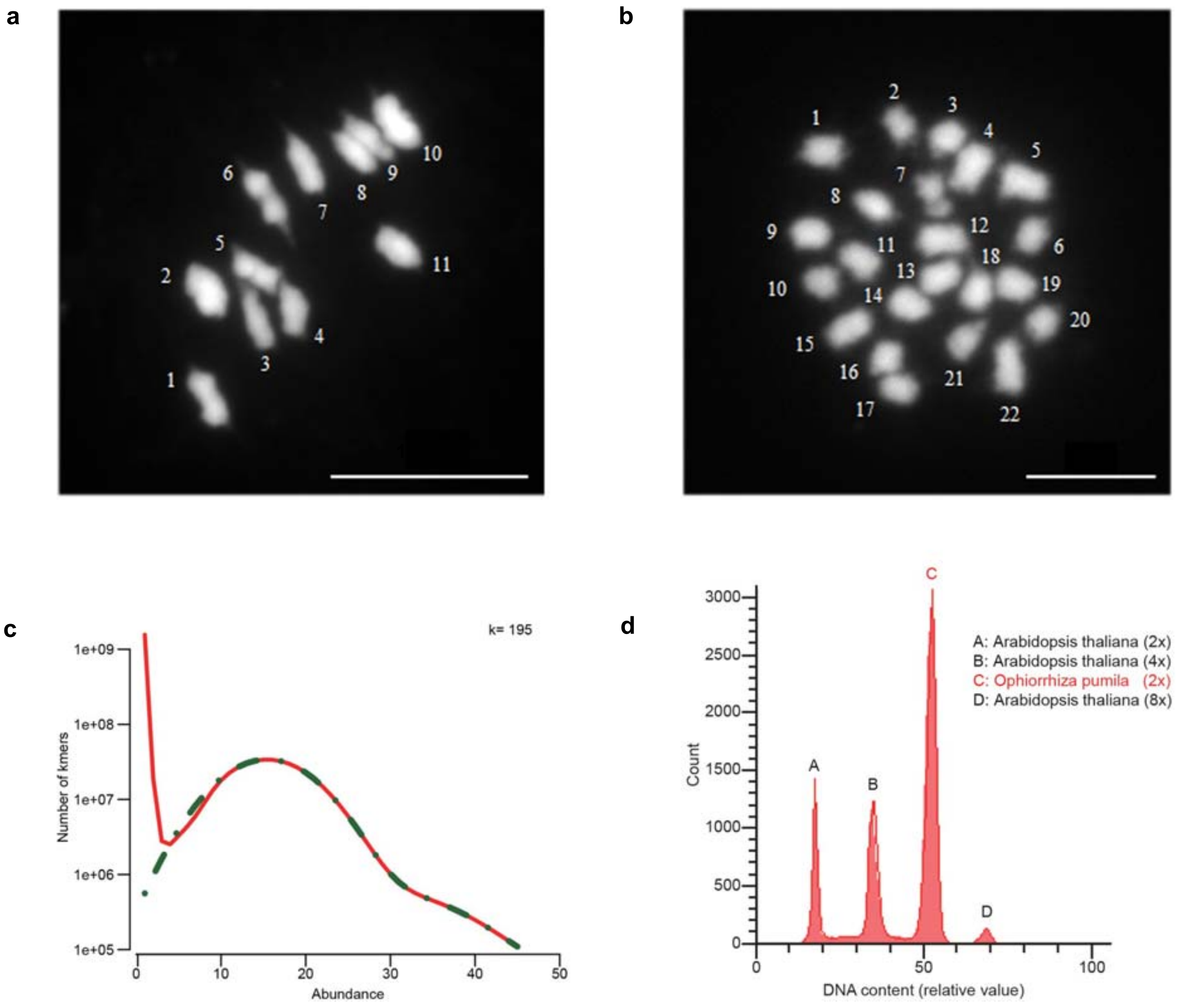

Supplementary Fig. 4. *Ophiorrhiza pumila* karyotype and genome size estimation. (a) Meiotic metaphase I chromosomes of *O. pumila*. The chromosomes form 11 bivalents. The scale bar corresponds to 10 µm. **(b)** Mitotic chromosomes of *O. pumila*, representing a diploid genome with 2n = 22 chromosomes. The scale bar corresponds to 5 µm. The karyotyping was performed twice, and for each experiment, at least 10 metaphase plates were analyzed. **(c)** Genome size estimation of *O. pumila* genome using KmerGenie software. Genome size at kmer-195 was estimated as 440 Mb. **(d)** Flow cytometer-based genome estimation, with genome size predicted as 420 Mb. Here count refers to the number of nuclei.

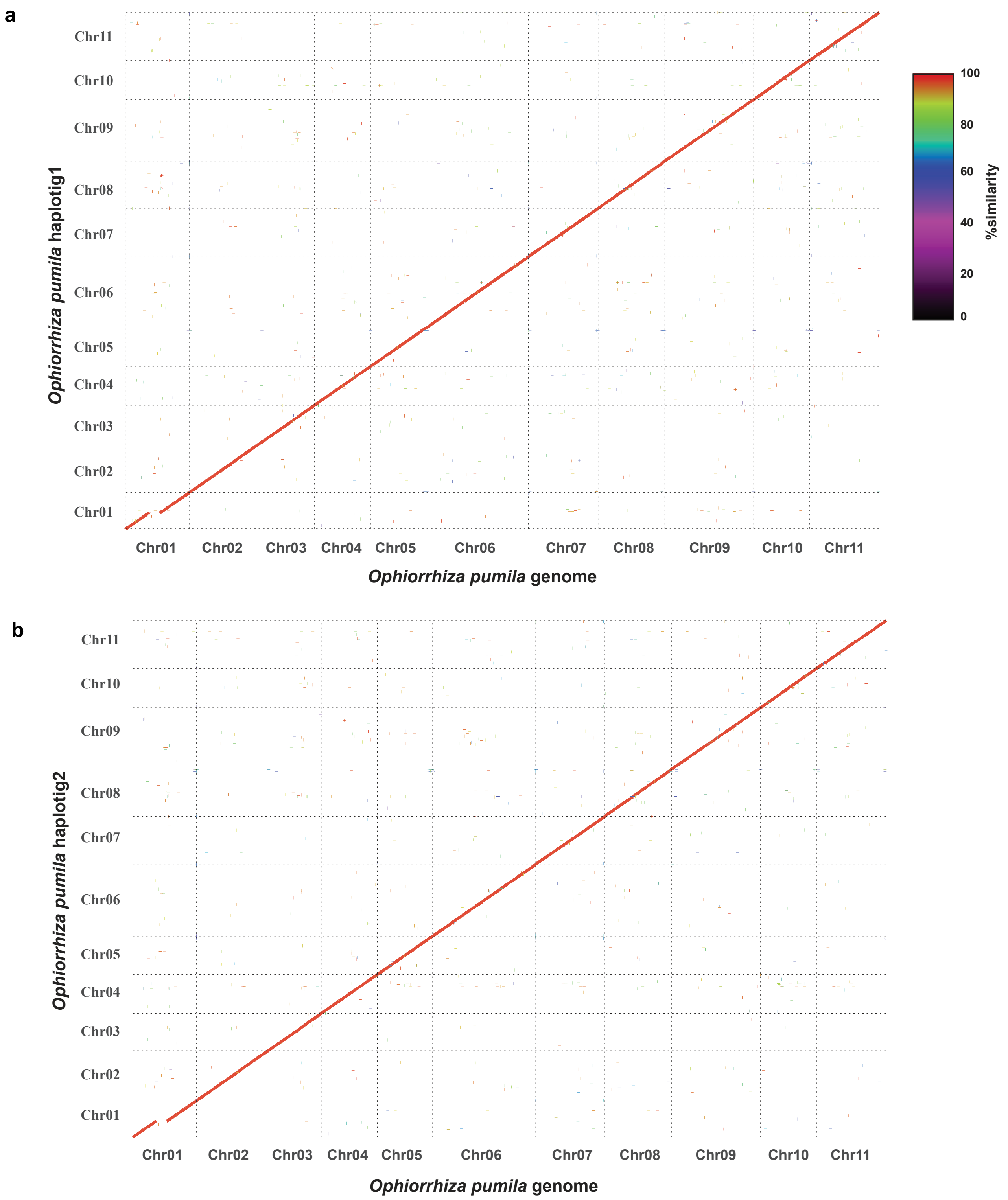

Supplementary Fig. 5. Alignment of phased and reference genome assemblies of *Ophiorrhiza pumila*. Phased genome assemblies of *O. pumila*, haplotig1 **(a)** and haplotig2 **(b)**, were aligned to the reference genome using PROmer from MUMmer package, and the alignments are represented by dot-plots.

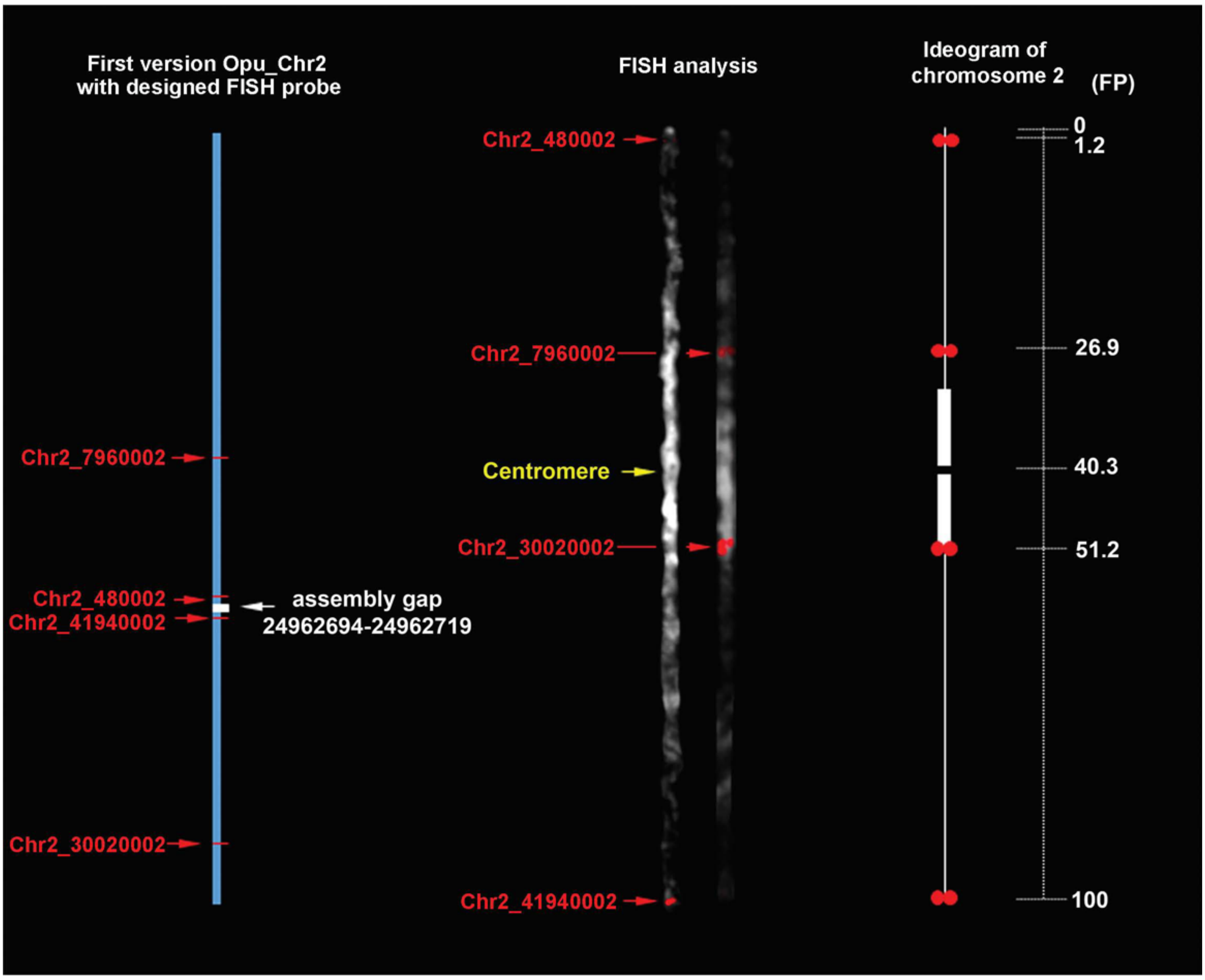

Supplementary Fig. 6. Fluorescence *in situ* hybridization (FISH) analysis for chromosome 2 of *Ophiorrhiza pumila*. The four probes (red text) correspond to the four different sites on chromosome 2 of *O. pumila*. While probes on the same chromosome arms were consistent with the expected assigned scaffolds, their positions at the assembly gap provided evidence of misalignment. The scaffolds at the assembly gap of chromosome 2 were re-oriented in the final reference genome of *O. pumila*. The experiment was performed twice, and for each experiment, we validated chromosomes for at least 10 slides.

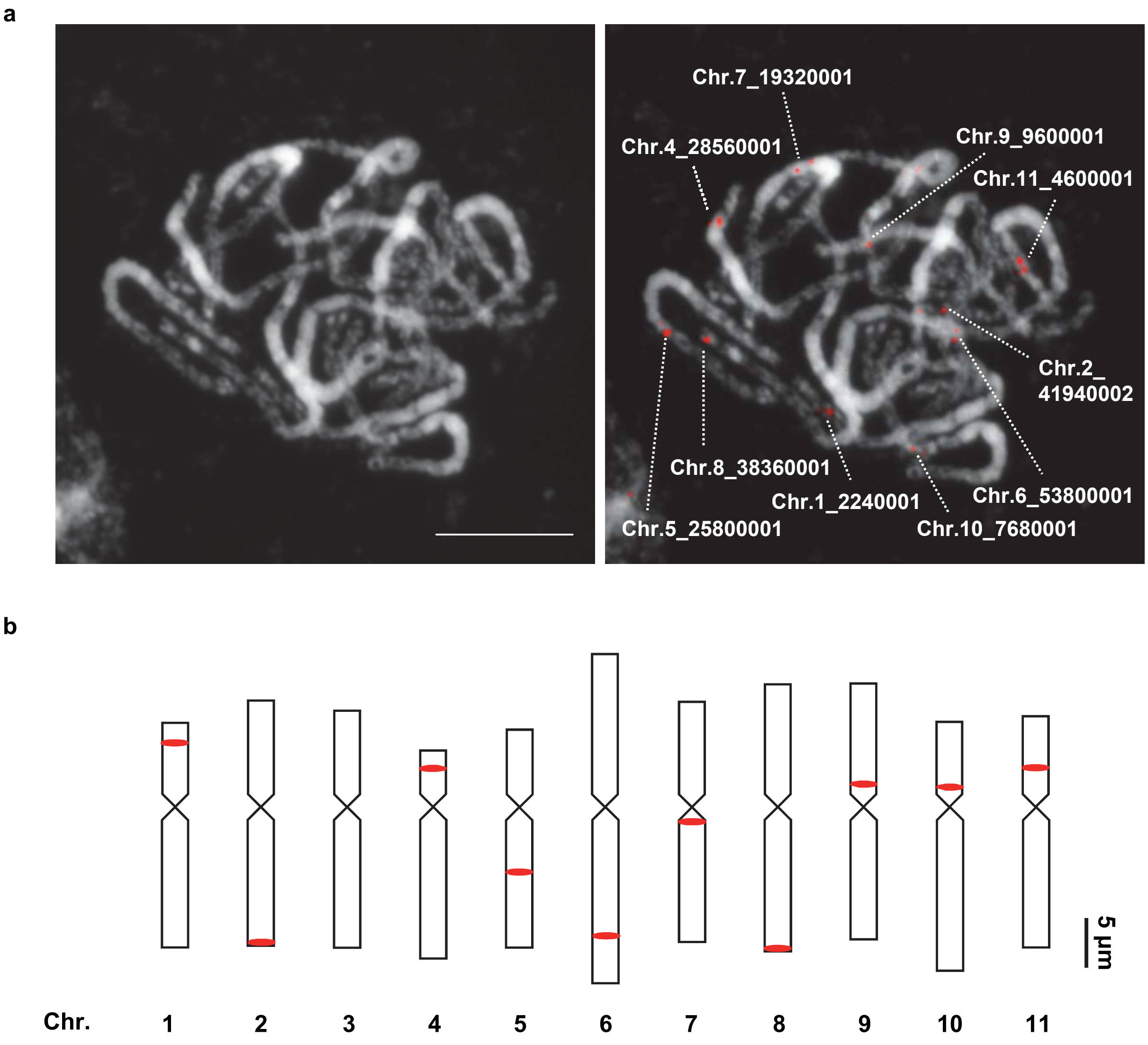

Supplementary Fig. 7. Identification of *Ophiorrhiza pumila* chromosomes and its karyotype. (a) DAPI stained *O. pumila* pachytene chromosomes and Fluorescence *in situ* hybridization (FISH) analysis-based identifications of the chromosomes. The scale bar corresponds to 5µm. **(b)** Ideogram of the *O. pumila* chromosome, are drawn based on Supplementary Table 1 results. Red bars indicate the position of FISH signals used in the identification of respective chromosomes, as shown in **(a)**. The experiment was repeated twice, and for each experiment, at least 10 chromosome slides were analyzed.

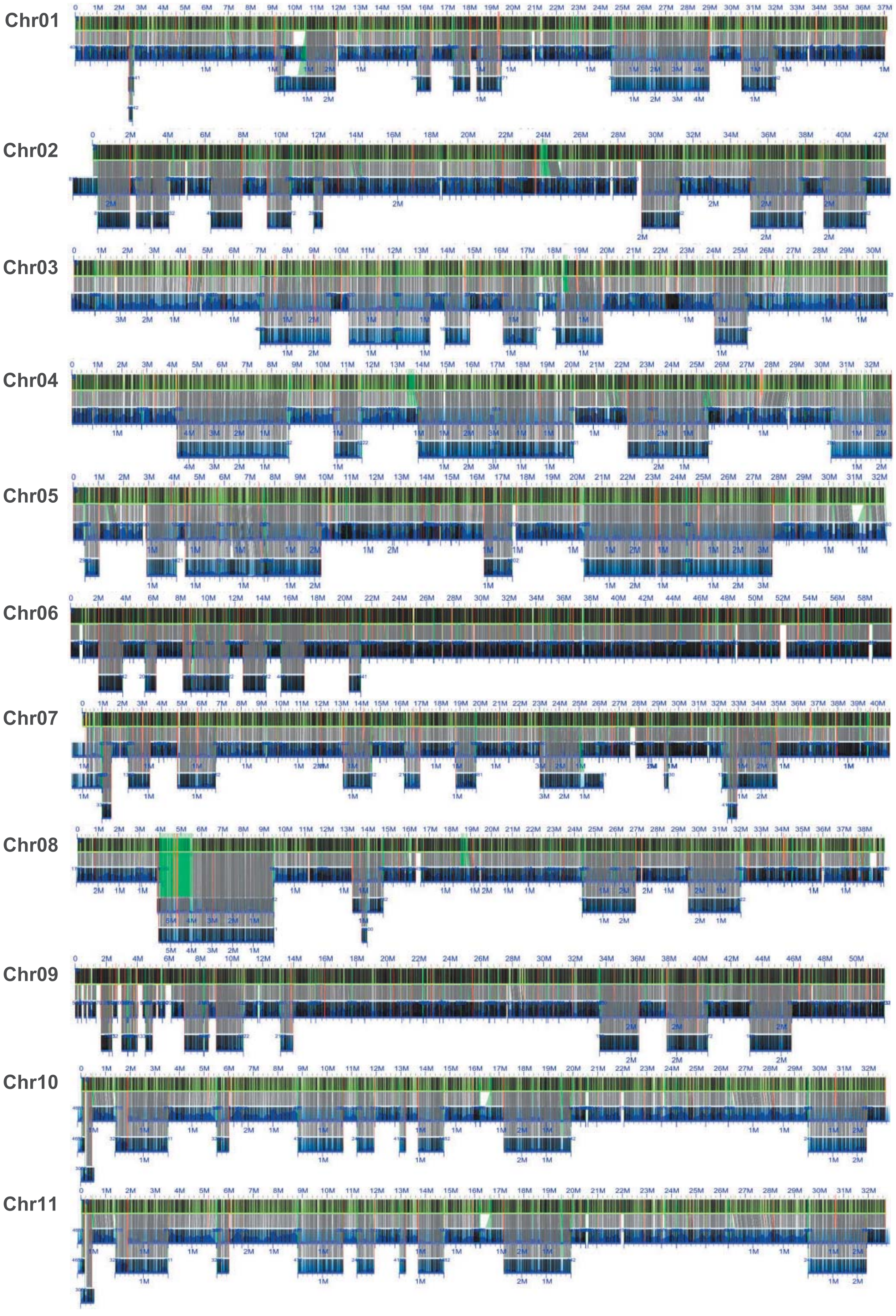

Supplementary Fig. 8. The *Ophiorrhiza pumila* reference genome assembly validation using Bionano de novo genome assembly as orthogonal evidence. All 11 chromosomes were supported by Bionano de novo assembly (blue color bars), with green and red color shades between these two assembly alignments representing regions of insertions or deletions, respectively.

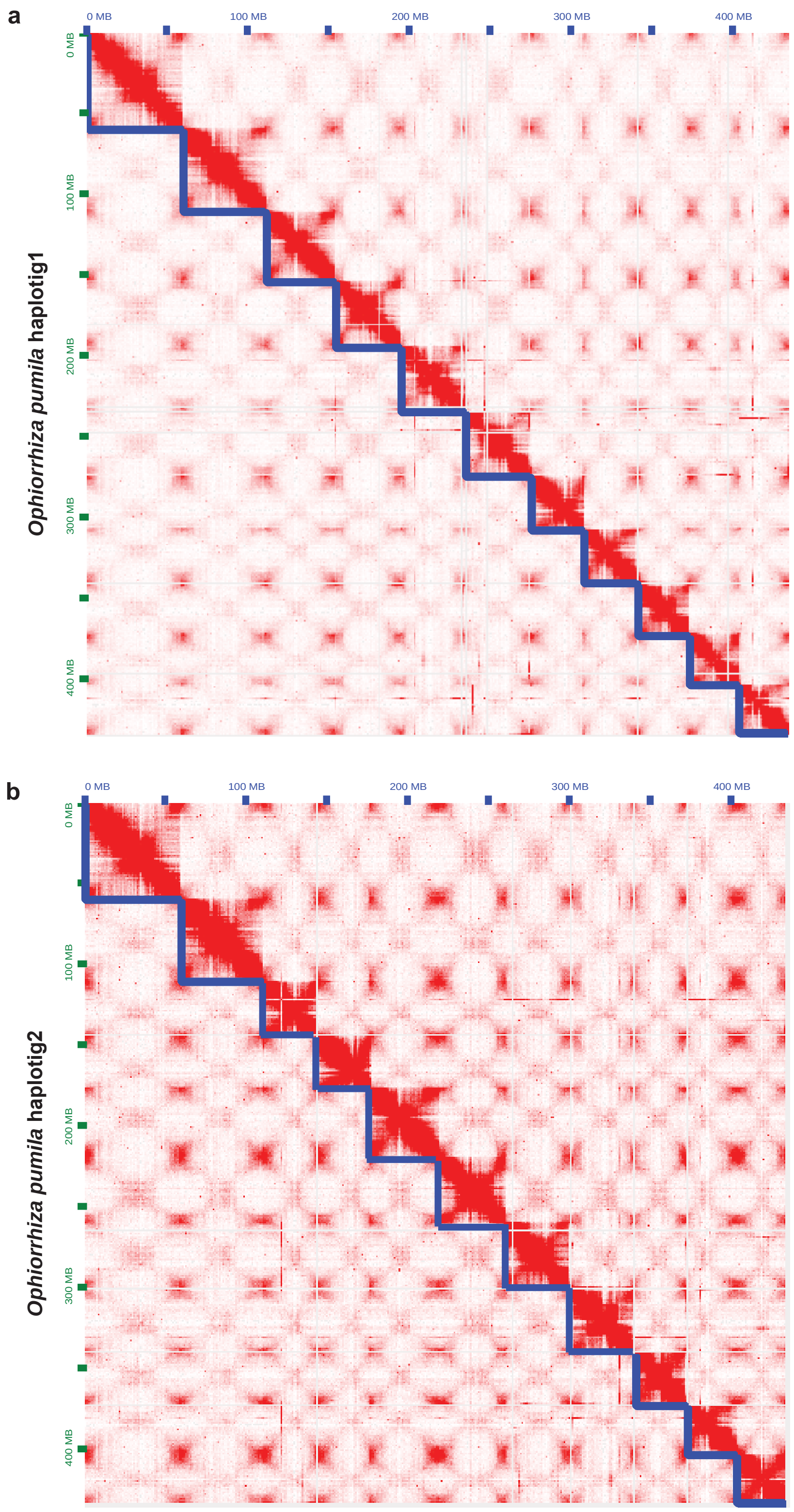

Supplementary Fig. 9. Hi-C contact map representing chromosome-scale phased genomes of *Ophiorrhiza pumila*. Hi-C contact matrix visualization for the *O. pumila* phased genomes, haplotig1 **(a)** and haplotig2 **(b)**. The pixel intensity represents the count of Hi-C links at 150 Kb size-windows on the chromosomes on a logarithmic scale. Darker red color indicates higher contact probability, while white space represents no or fewer contacts. The blue lines separate chromosomes for each phased genome.

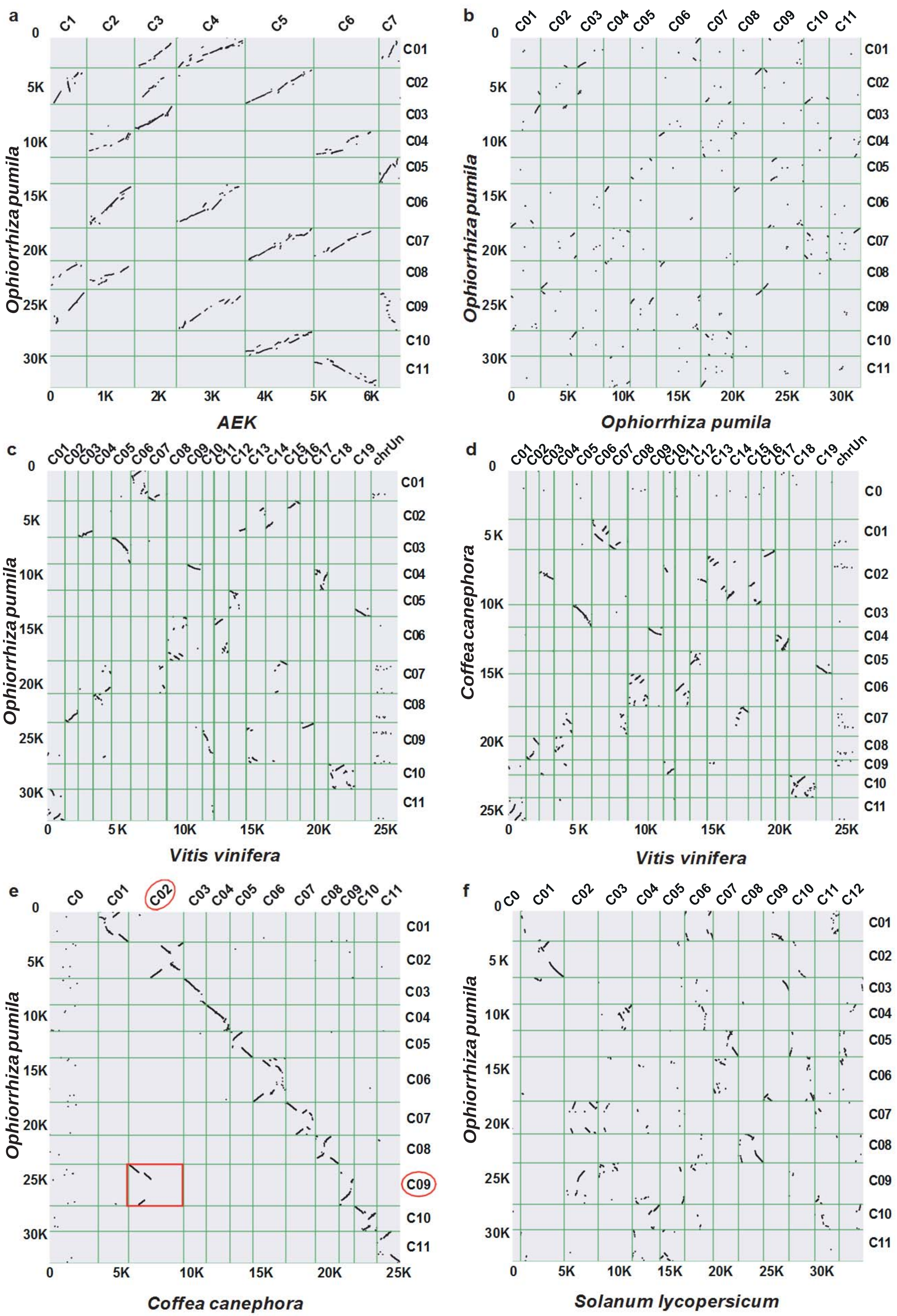

a
C1 C2 C3 C4 C5 C6 C7
C01 C02 C03 C04 C05 C06 C07 C08 C09 C10 C11
0 5K 10K 15K 20K 25K 30K
0 1K 2K 3K 4K 5K 6K
Ophiorrhiza pumila
AEK
b
C01 C02 C03 C04 C05 C06 C07 C08 C09 C10 C11
0 5K 10K 15K 20K 25K 30K
Ophiorrhiza pumila
c
C01 C02 C03 C04 C05 C06 C07 C08 C09 C10 C11 C12 C13 C14 C15 C16 C17 C18 C19 chrUn
0 5K 10K 15K 20K 25K 30K
0 5K 10K 15K 20K 25K
Ophiorrhiza pumila
Vitis vinifera
d
C0 C01 C02 C03 C04 C05 C06 C07 C08 C09 C10 C11
0 5K 10K 15K 20K 25K
Coffea canephora
Vitis vinifera
e
C0 C01 C02 C03 C04 C05 C06 C07 C08 C09 C10 C11
0 5K 10K 15K 20K 25K 30K
0 5K 10K 15K 20K 25K
Ophiorrhiza pumila
Coffea canephora
f
C0 C01 C02 C03 C04 C05 C06 C07 C08 C09 C10 C11 C12
0 5K 10K 15K 20K 25K 30K
Ophiorrhiza pumila
Solanum lycopersicum

Supplementary Fig. 10. Synteny dot-plot for *Ophiorrhiza pumila* genome within, and with selected plant species including ancestral eudicot karyotype (AEK). (a) Dot-plot based synteny visualization showed a near-perfect 1:3 synteny relationship between AEK and *O. pumila* genome. Synteny dot-plot between **(b)** *O. pumila* - *O. pumila*, **(c)** *O. pumila* - *Vitis vinifera*, **(d)** *Coffea canephora* - *V. vinifera*, **(e)** *O. pumila* - *C. canephora*, and **(f)** *O. pumila* - *Solanum lycopersicum* showed conserved gene blocks within these species. Synteny dot-plot between *O. pumila* and *C. canephora* showed a 1:1 synteny depth with an interesting interaction between chromosome 9 of the *Ophiorrhiza* genome and chromosome 2 of coffee genome, as shown in the red box.

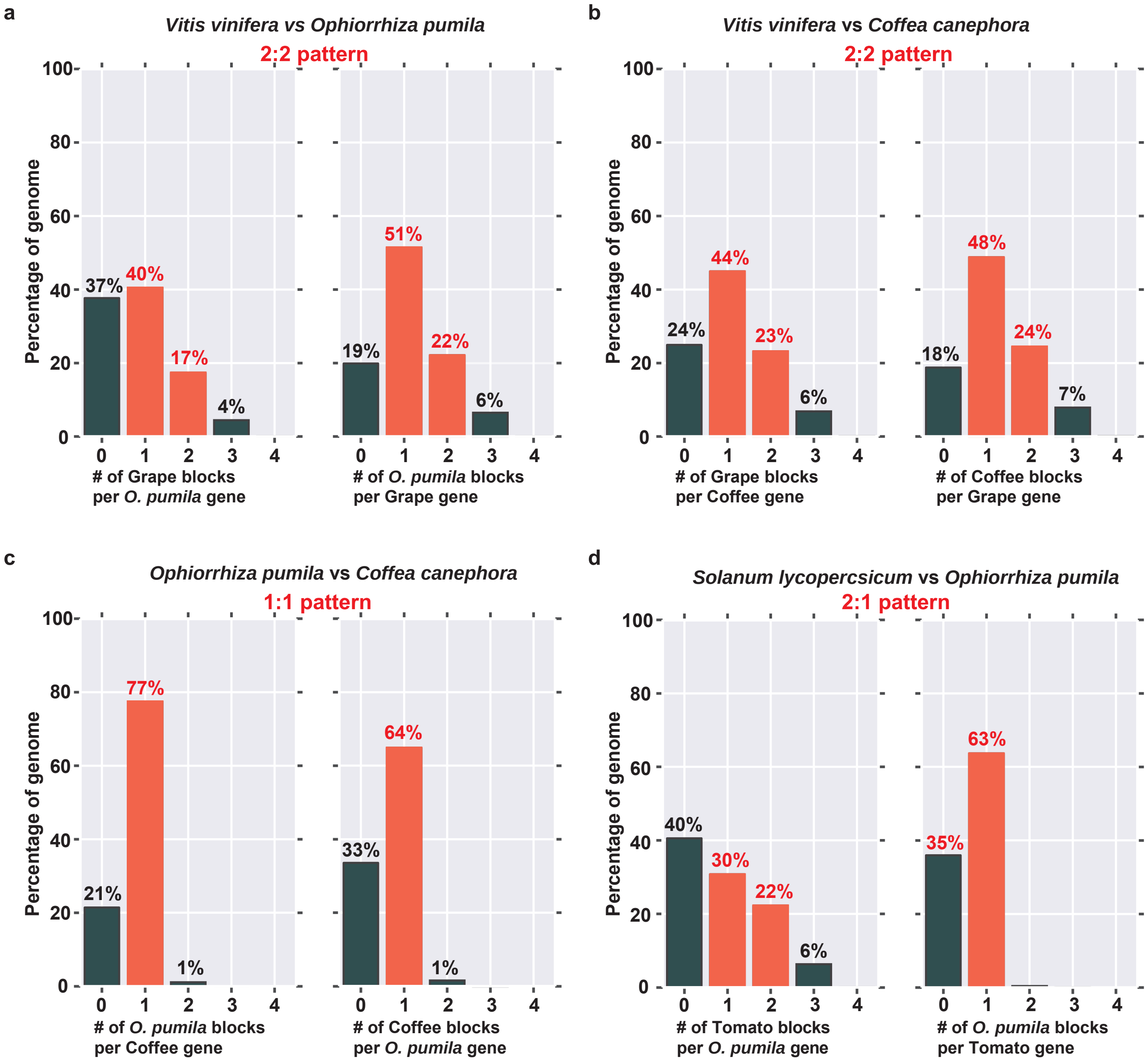

Supplementary Fig. 11. Estimated synteny depths between selected plant species. Synteny depths for *Vitis vinifera* with *Ophiorrhiza pumila* **(a)** and *Coffea canephora* **(b)** was determined as 2:2, while synteny depths for *O. pumila* with *C. canephora* **(c)** and *Solanum lycopersicum* **(d)** as 1:1 and 2:1, respectively.

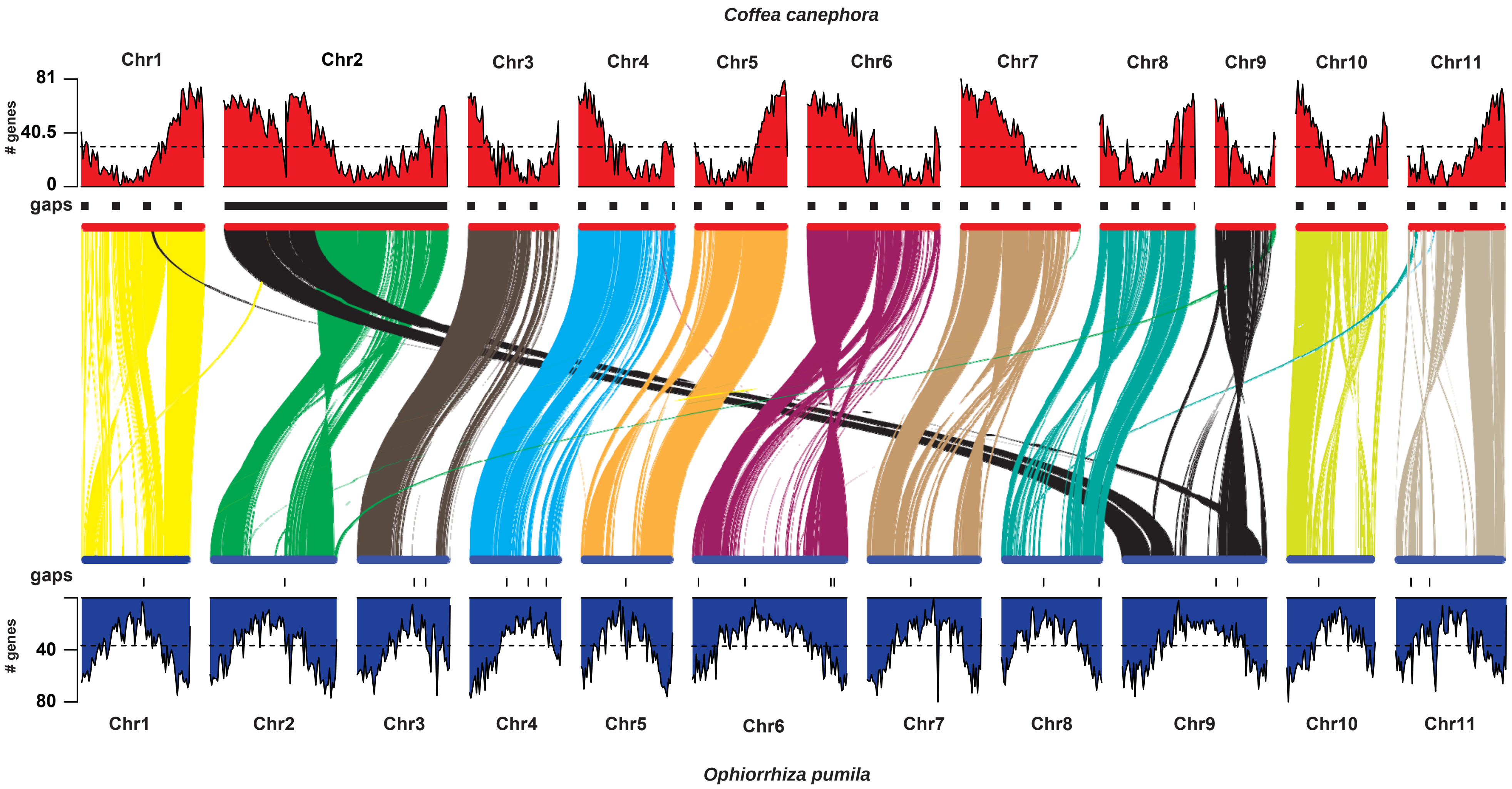

Supplementary Fig. 12. Macrosynteny analysis for *Ophiorrhiza pumila* and *Coffea canephora* genome assemblies. Karyotypes with synteny associations, gene density distribution, and assembly gaps for *Ophiorrhiza* and coffee genome are represented. The color of synteny relationships are based on associations with *Ophiorrhiza* chromosomes.

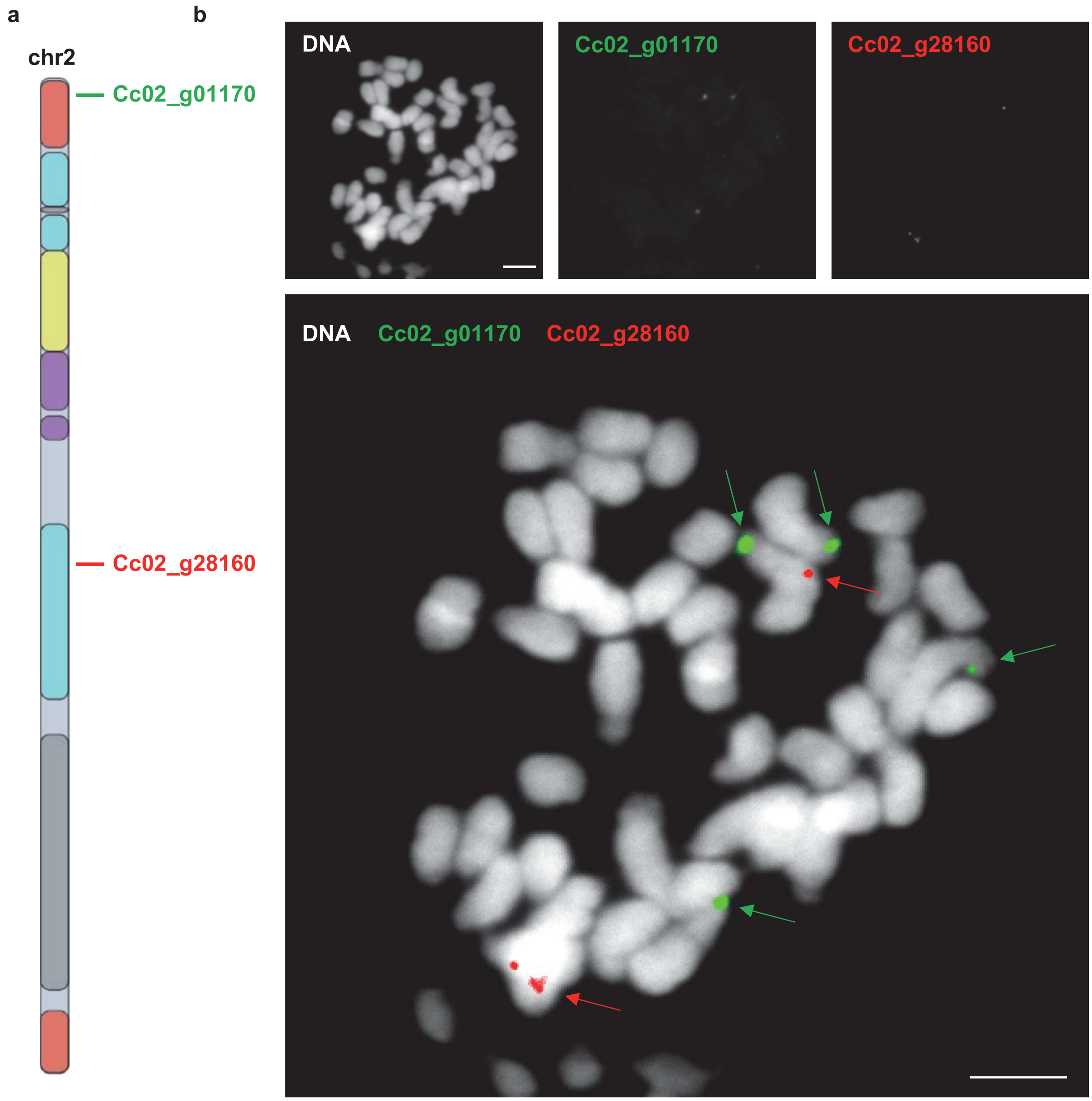

Supplementary Fig. 13. Fluorescence *in situ* hybridization (FISH) analysis to test potential mis-assembly in the chromosome 2 of *Coffea arabica.* **(a)** Sequence positions for two FISH probes in *Coffea canephora* chromosome 2 representing two regions that showed syntenic relationships with chromosome 2 and 9 of *Ophiorrhiza pumila* as shown in Supplementary Fig. 12. The chromosome 2 map of *C. canephora* with assigned ancestral blocks was obtained from Coffee Genome hub (http://coffee-genome.org/coffeacanephora). The probe names are corresponding to the gene ID in the *C. canephora* genome. **(b)** FISH mapping using two FISH probes on *C. arabica* chromosomes (2n = 4x = 44). The Cc02_g01170 probe shows four FISH signals, and the Cc02_g28160 probe shows two signals on 44 chromosomes of the *C. arabica*. These six signals (green and red arrows) were not detected on the same chromosome. The experiment was repeated twice, and for each experiment, we verified at least six slides. The scale bar corresponds to 5 µm.

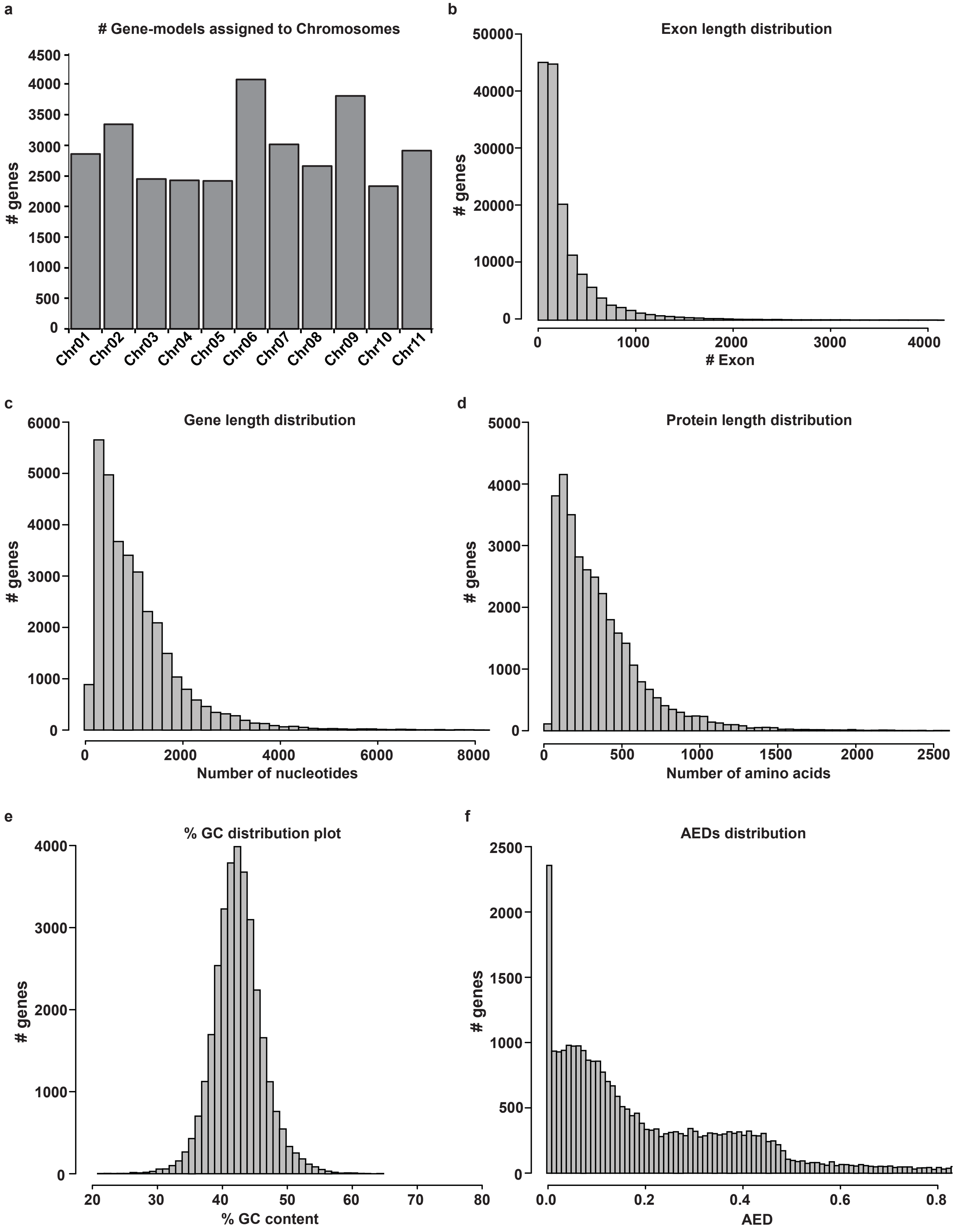

Supplementary Fig. 14. Characteristics of *Ophiorrhiza pumila* predicted gene models. (a) Gene models predicted across 11 chromosomes of *O. pumila*. **(b)** Exon length distribution plot. **(c)** Gene length distribution plot. **(d)** Protein length distribution plot. **(e)** % GC distribution plot for predicted gene models. **(f)** Distribution of annotation edit distance (AED).

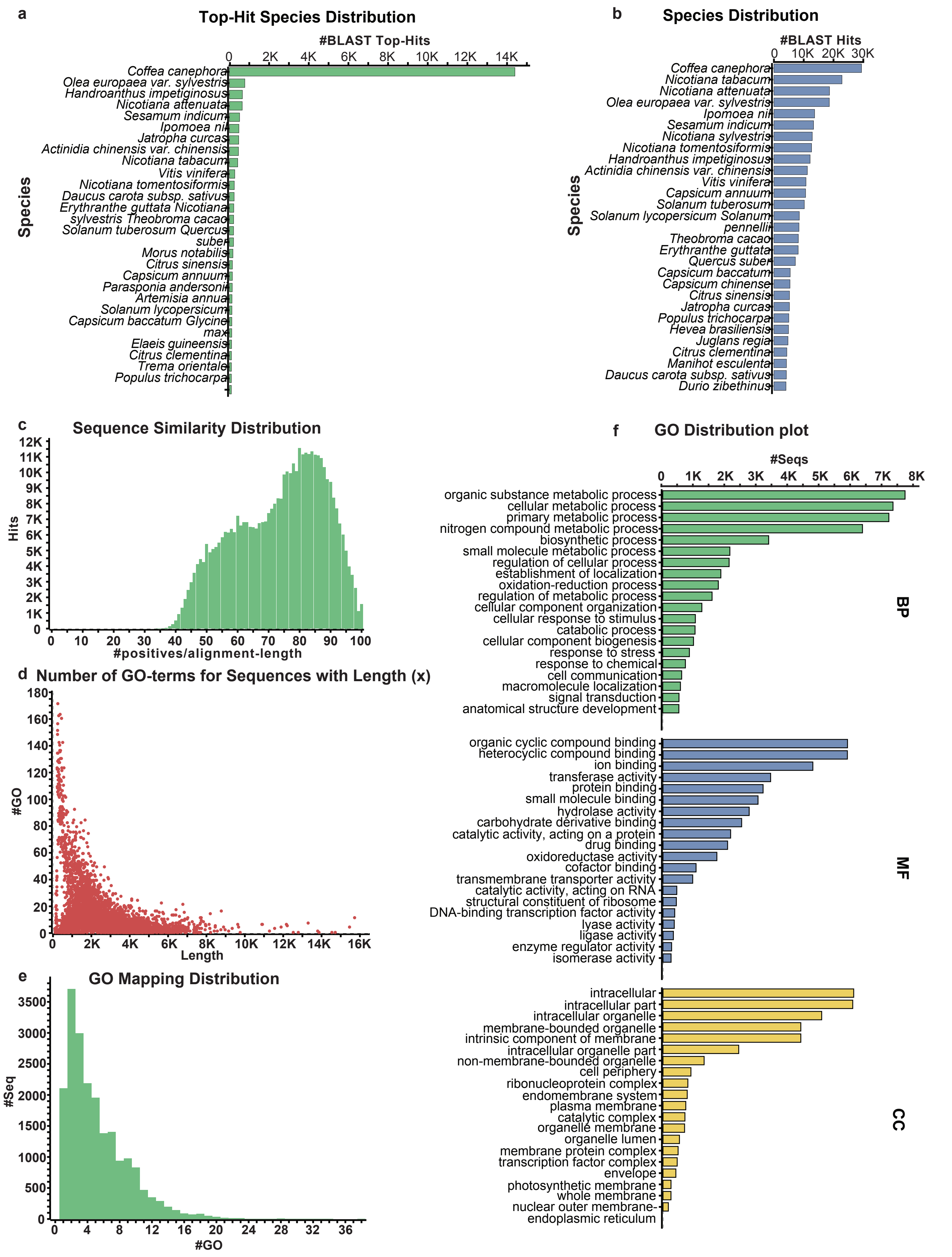

Supplementary Fig. 15. Functional classification of *Ophiorrhiza pumila* gene models. (a) Top-hit species distribution plot. **(b)** Species distribution plot based on database-based annotation. **(c)** Sequence similarity distribution plot. **(d)** Gene length distribution plot with number of assigned gene ontologies (GO). **(e)** GO mapping distribution plot. **(f)** GO distribution as biological process (BP), Molecular function (MF), and Cellular component (CC) categories. Annotation based functional classification was performed using Omics box software. Source data are provided as a Source Data File.

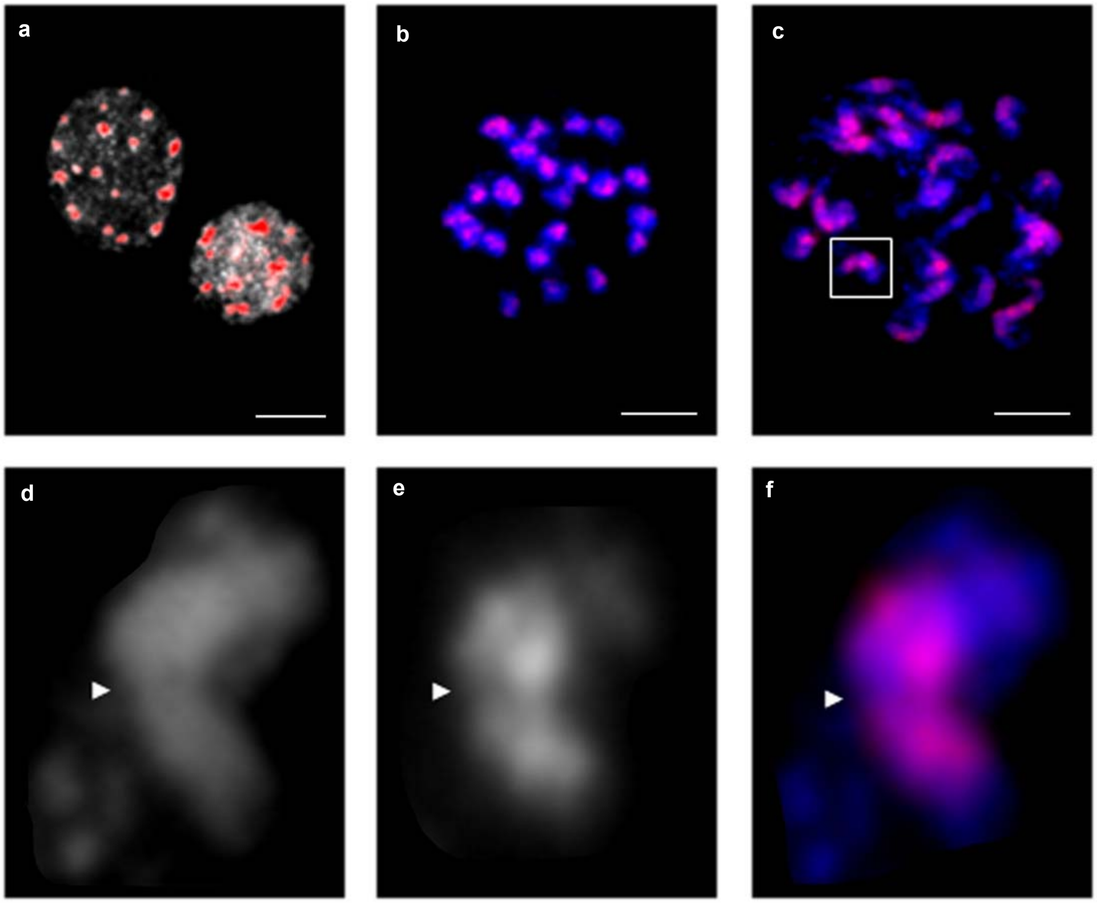

Supplementary Fig. 16. Chromosome primary constriction and centromere localization in *Ophiorrhiza pumila* chromosomes using Fluorescence *in situ* hybridization (FISH) analysis. (a) Detection of OpCEN (red) at the heterochromatic chromocenters in the nuclei (white). **(b)** Centromeric detection of OpCEN (red) in somatic metaphase chromosomes (blue). **(c)** Detection of OpCEN (red) at centromeric regions of prometaphase chromosomes (blue). **(d)-(f)** Cut-off image for chromosome selected in the white color box in **(c)**. **(d)** DAPI-stained chromosome. **(e)** Detection of OpCEN signal. **(f)** Merged image. Arrowheads indicated primary constriction. Repeat analysis using *O. pumila* reference genome identified pericentromeric repeat sequence, conserved across all chromosomes, and positioned sat putative centromere region, named OpCEN. Probes designed for OpCEN labeled putative centromere of *O. pumila*. The scale bar corresponds to 5µm. The FISH experiment was performed twice, and for each experiment, at least 10 slides were analyzed.

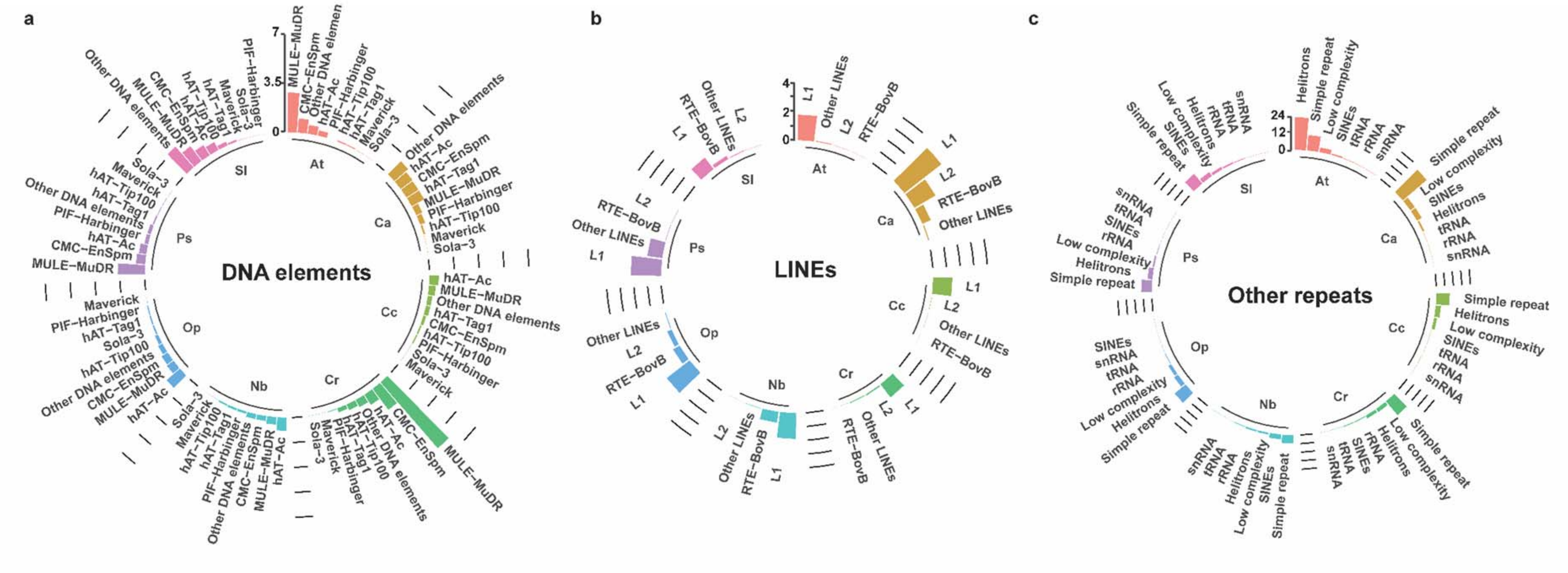

Supplementary Figure. 17. Distribution of repeat elements across eight plant species. Percentage of genome content consisting of **(a)** DNA element repeats. **(b)** LINEs repeat; and **(c)** Other repeats. At: *Arabidopsis thaliana*, Ca: *Camptotheca acuminata*, Cc: *Coffea canephora*, Cr: *Catharanthus roseus*, Nb: *Nicotiana benthamiana*, Op: *Ophiorrhiza pumila*, Ps: *Papaver somniferum*, Sl: *Solanum lycopersicum*. Source data are provided as a Source Data file.

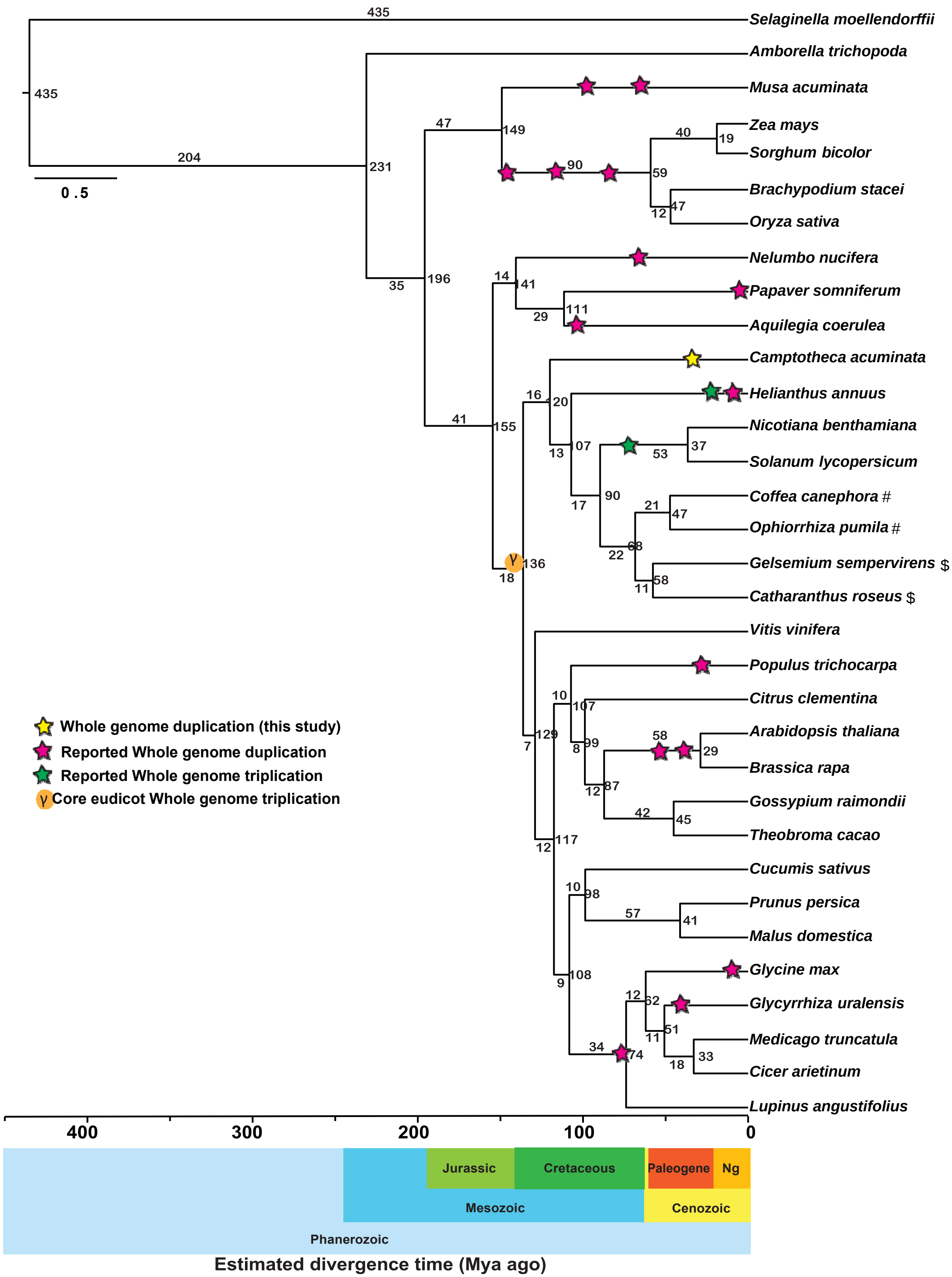

Supplementary Fig. 18. Estimated divergence and whole genome duplication for monoterpene indole alkaloids producing species. The lineage divergence time as branch length is indicated at each of the branch points in Million years ago (Mya). Divergence time for *Ophiorrhiza pumila* with coffee and *Camptotheca acuminata* was estimated at 47 Mya and 120 Mya, respectively. The whole-genome duplication for *C. acuminata* was identified and the duplication time was estimated. "#" and "$" depicts species from Rubiaceae and Apocynaceae families, respectively.

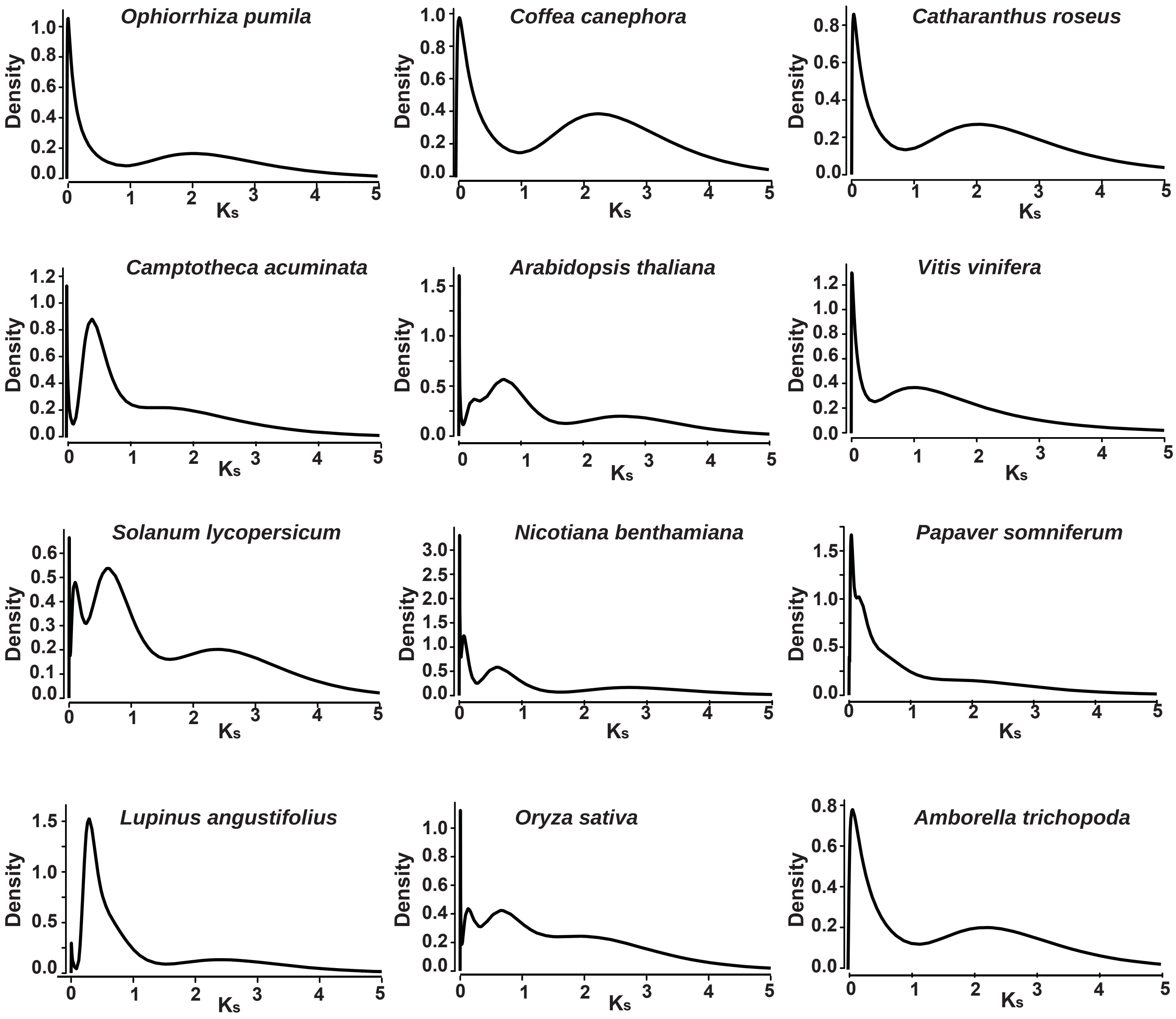

Supplementary Fig. 19. Density plot depicting *Ks* (synonymous substitution rate) distribution of paralogous gene pairs for selected eudicot plant genome assemblies.

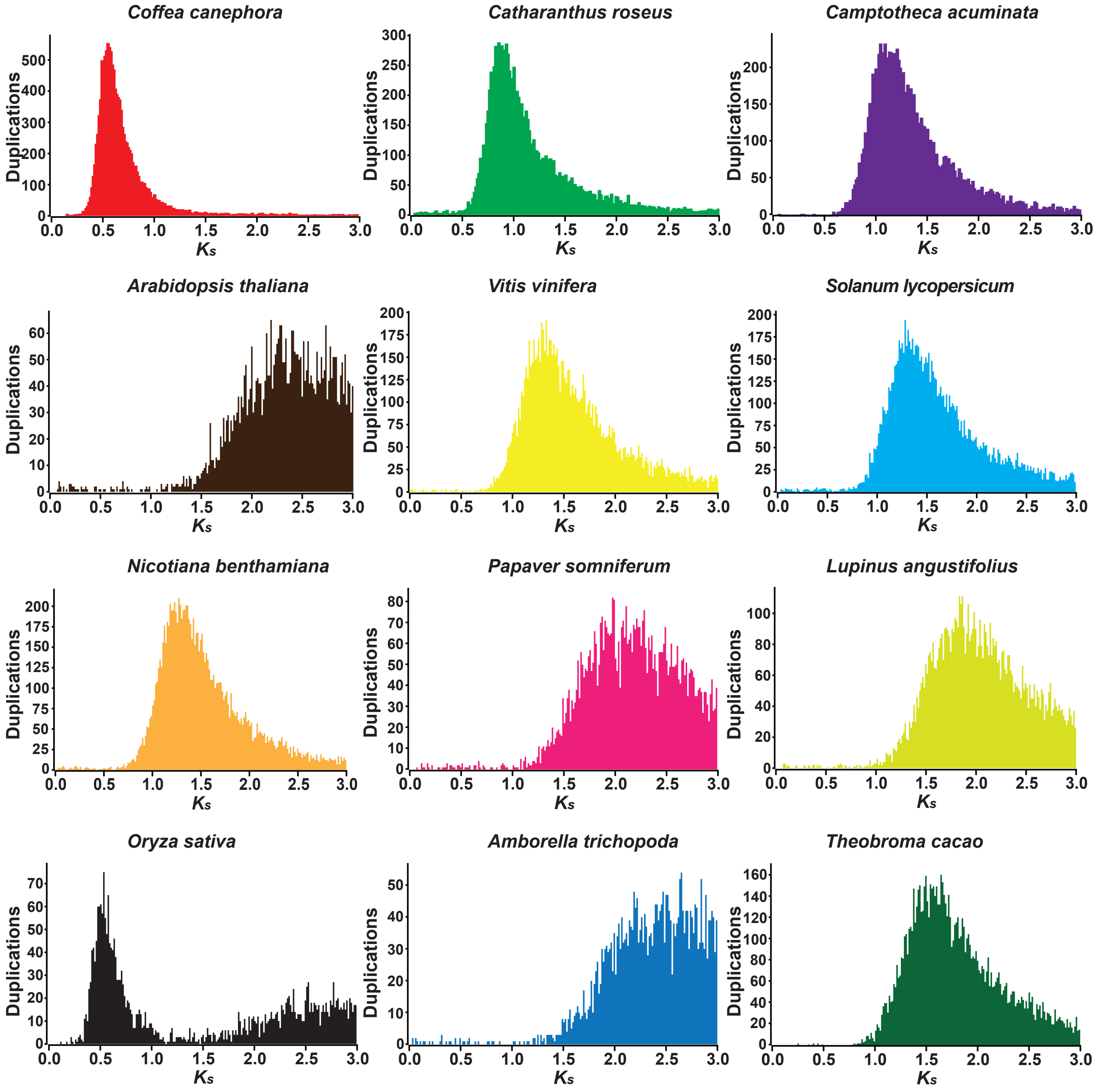

Supplementary Fig. 20. Density plot depicting *Ks* (synonymous substitute rate) distribution for orthologous gene pairs identified between *Ophiorrhiza pumila* and 12 other eudicot plant genome assemblies.

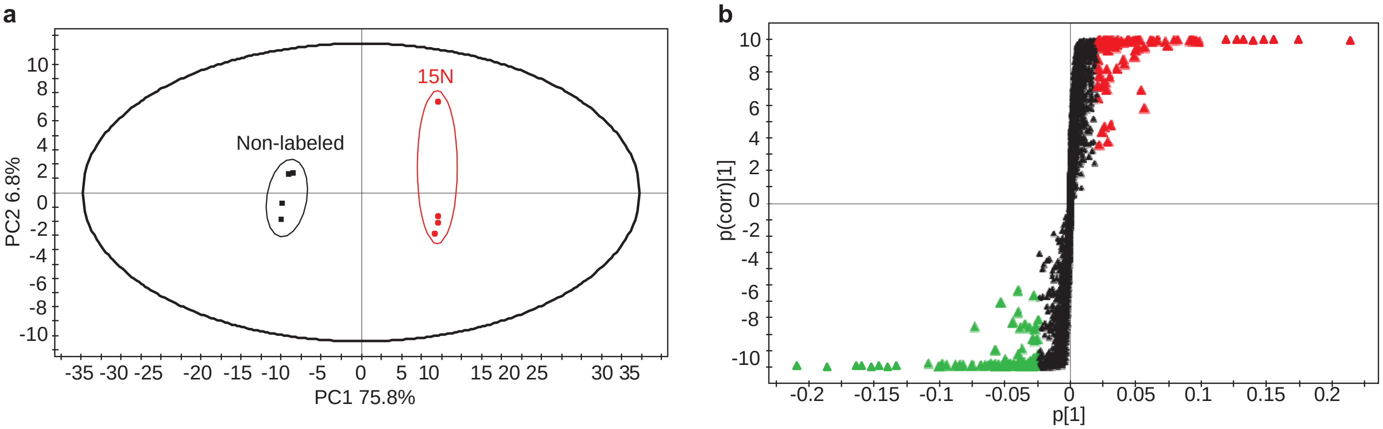

Supplementary Fig. 21. Metabolite profiling for complete nitrogen labeled (^{15}N) or non-labeled hairy roots to identify nitrogen-containing metabolites in *Ophiorrhiza pumila*. (a) Principle component analysis for labeled and non-labeled hairy root metabolite profiling datasets. Five biological replicates for each condition were used for metabolite profiling. **(b)** S-plot based multivariate analysis approach to select characteristic metabolite features of labeled and non-labeled hairy root samples. Metabolite features colored red and green represents stable isotope labeled and non-labeled metabolites, respectively.

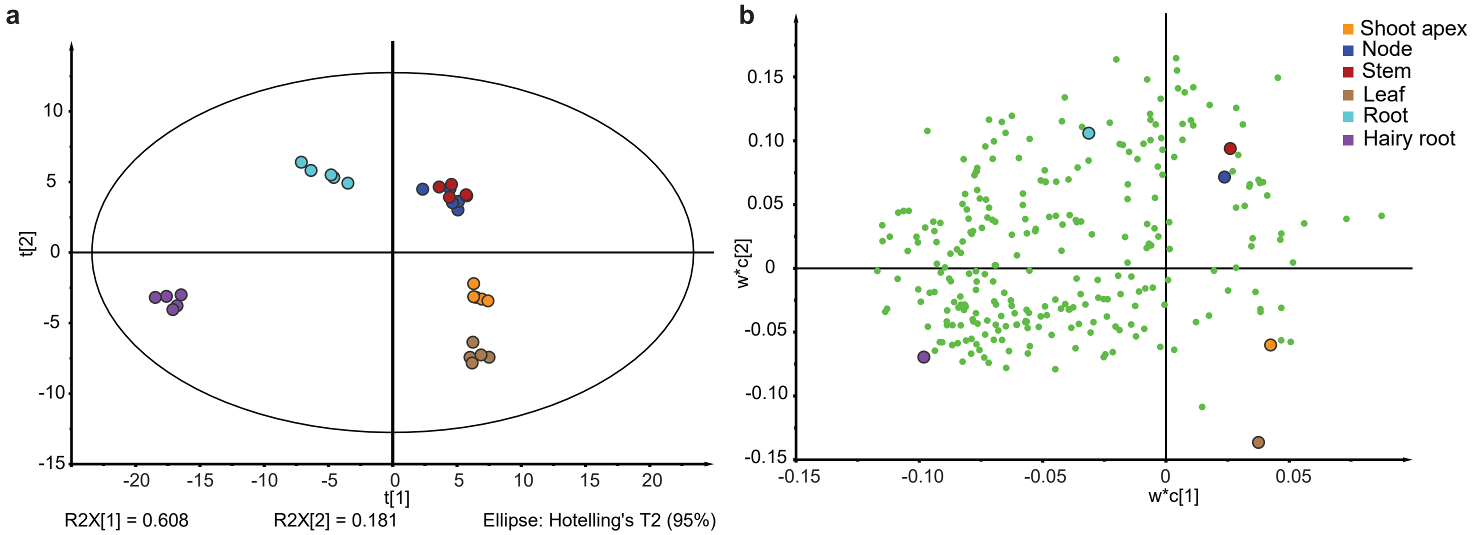

Supplementary Fig. 22. Metabolite profiling for six tissues of *Ophiorrhiza pumila*. *O. pumila* metabolome database was established by assigning chemical identity to metabolite features using previously reported complete carbon labeling approach and complete nitrogen labeling approach used in this study. **(a)** Principle component analysis. **(b)** Loadings plot for metabolite profiling datasets of six tissues. Five biological replicates for each tissue were used for metabolite profiling.

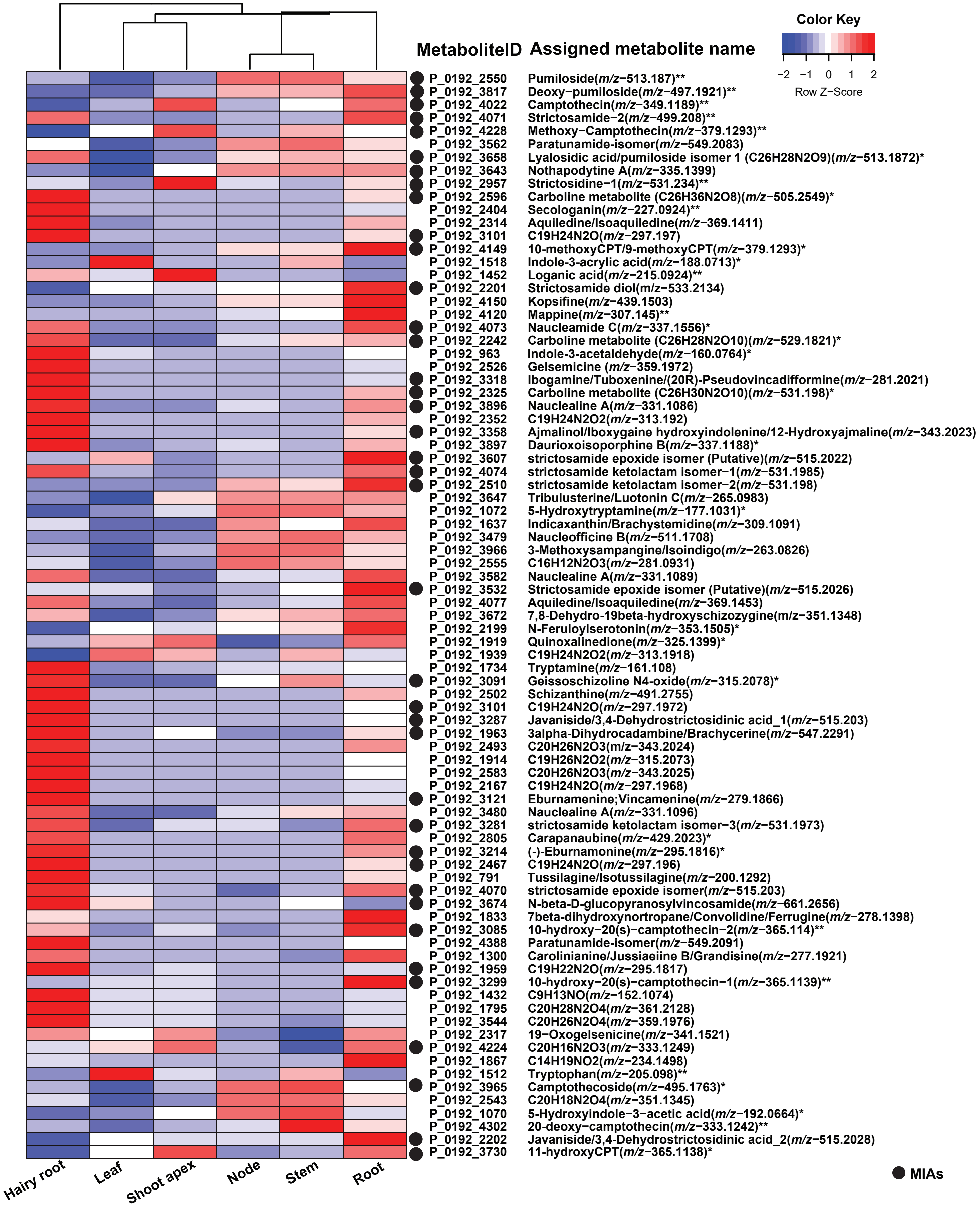

Supplementary Fig. 23. Accumulation of specialized metabolites in six tissues of *Ophiorrhiza pumila*. * and ** represent chemically assigned metabolites based on MS/MS analysis using the public database and pure standards, respectively; *m/z*-mass by charge ratio. The color intensity refers to the scaled median intensity of metabolites across tissues of *O. pumila* using five biological replicates for each tissue.

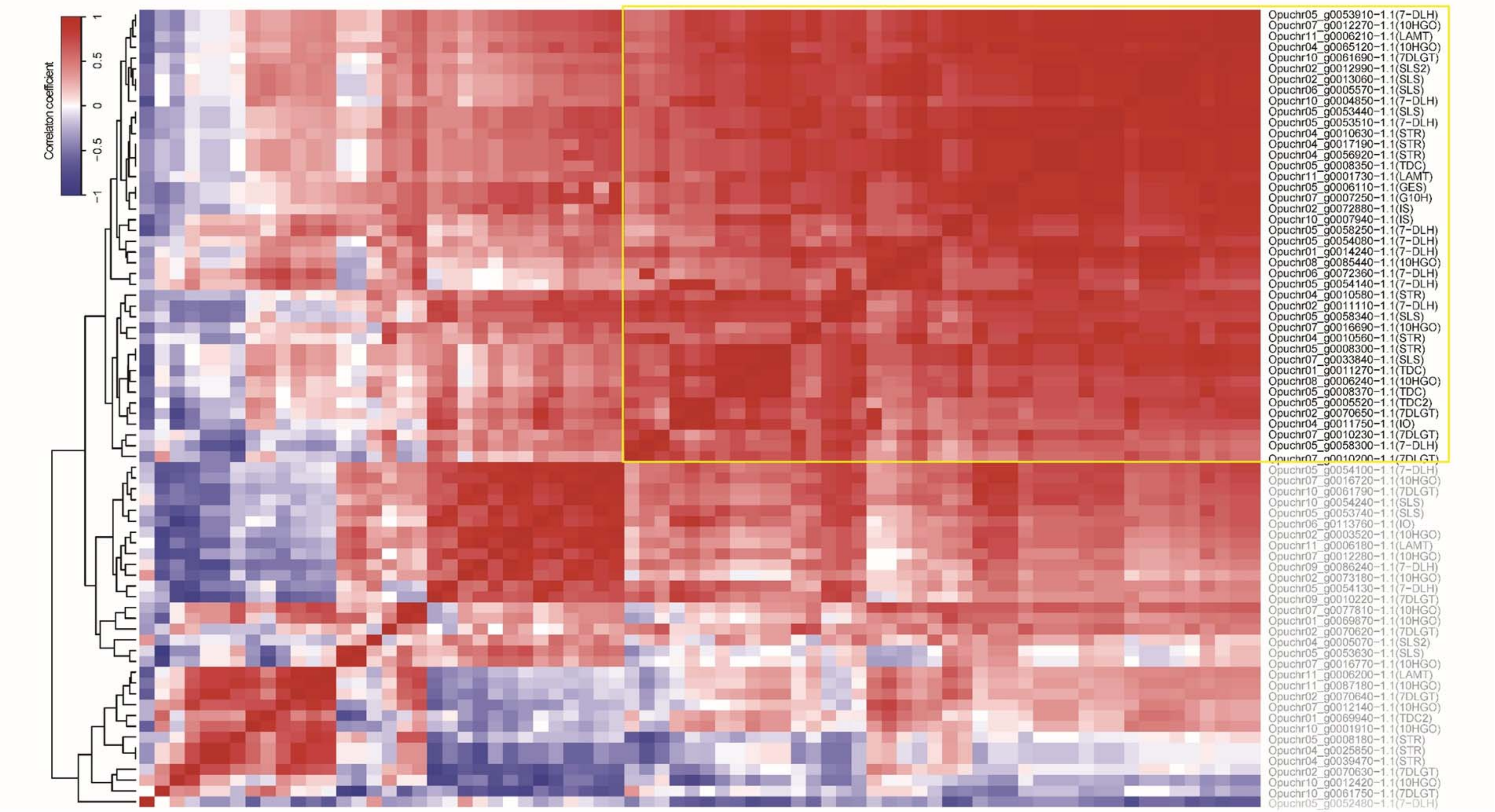

Supplementary Fig. 24. Coexpression based hierarchical clustering for genes assigned to enzymes from the secoiridoid biosynthesis pathway in *Ophiorrhiza pumila*. Spearman's correlation coefficients were calculated using transcript expression data for seven tissues of *O. pumila*. Hierarchical clustering identified complete secoiridoid biosynthesis pathway as a gene cluster at the second order of the dendrogram (selected within a yellow-colored box). Source data are provided as a Source Data file.

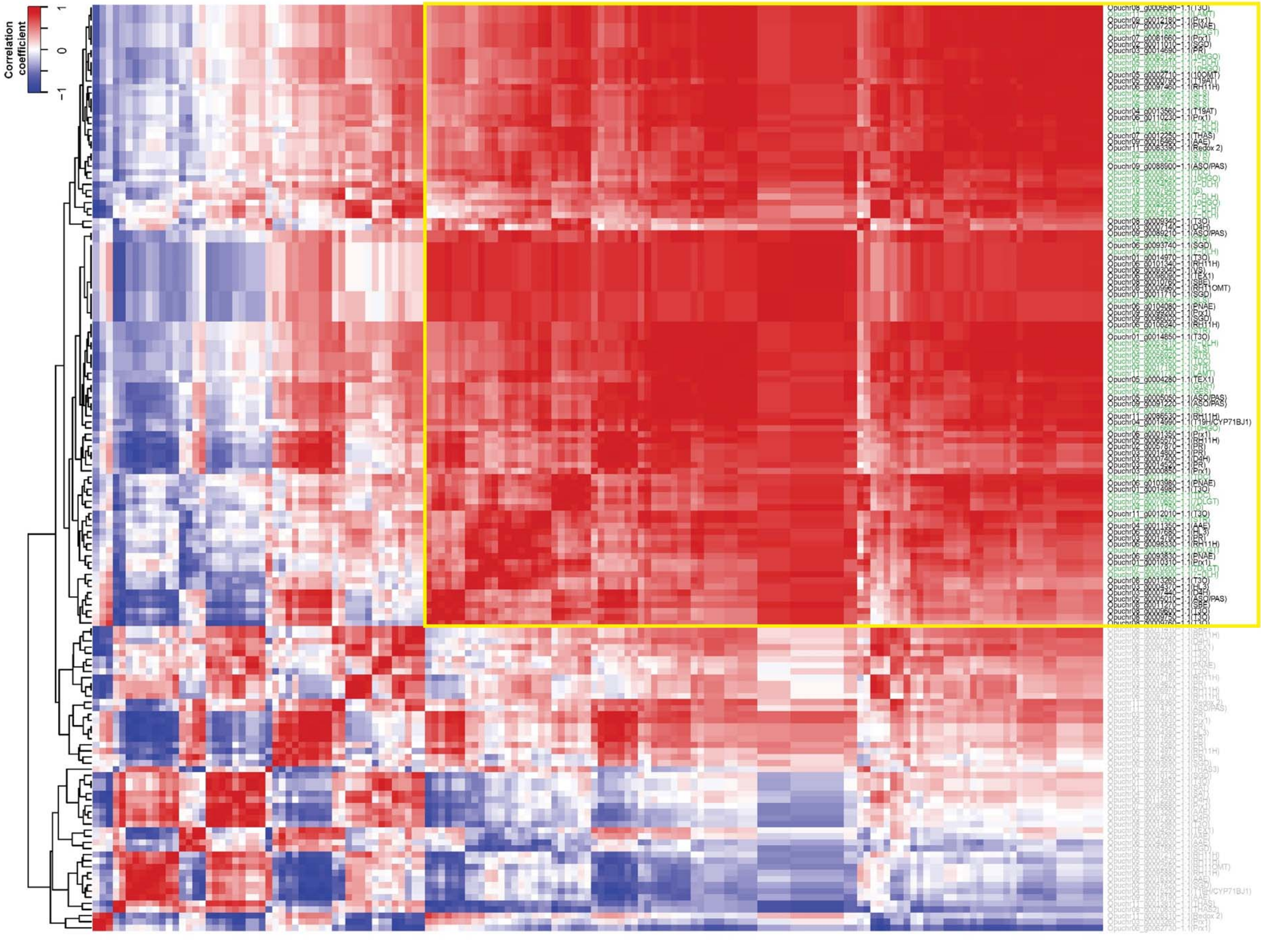
Correlation coefficient
1
0
−1

Supplementary Fig. 25. Coexpression based hierarchical clustering for genes assigned to enzymes from monoterpene indole alkaloid (MIA) biosynthesis in *Ophiorrhiza pumila*. Spearman's correlation coefficients were calculated using transcripts expression data for seven tissues of *O. pumila*. Genes associated with MIA biosynthesis and membered within a coexpressed gene-cluster (selected within a yellow-colored box) including genes assigned to secoiridoid biosynthesis pathway were selected. Genes colored as green are genes assigned to secoiridoid biosynthesis pathways. Source data are provided as a source Data file.

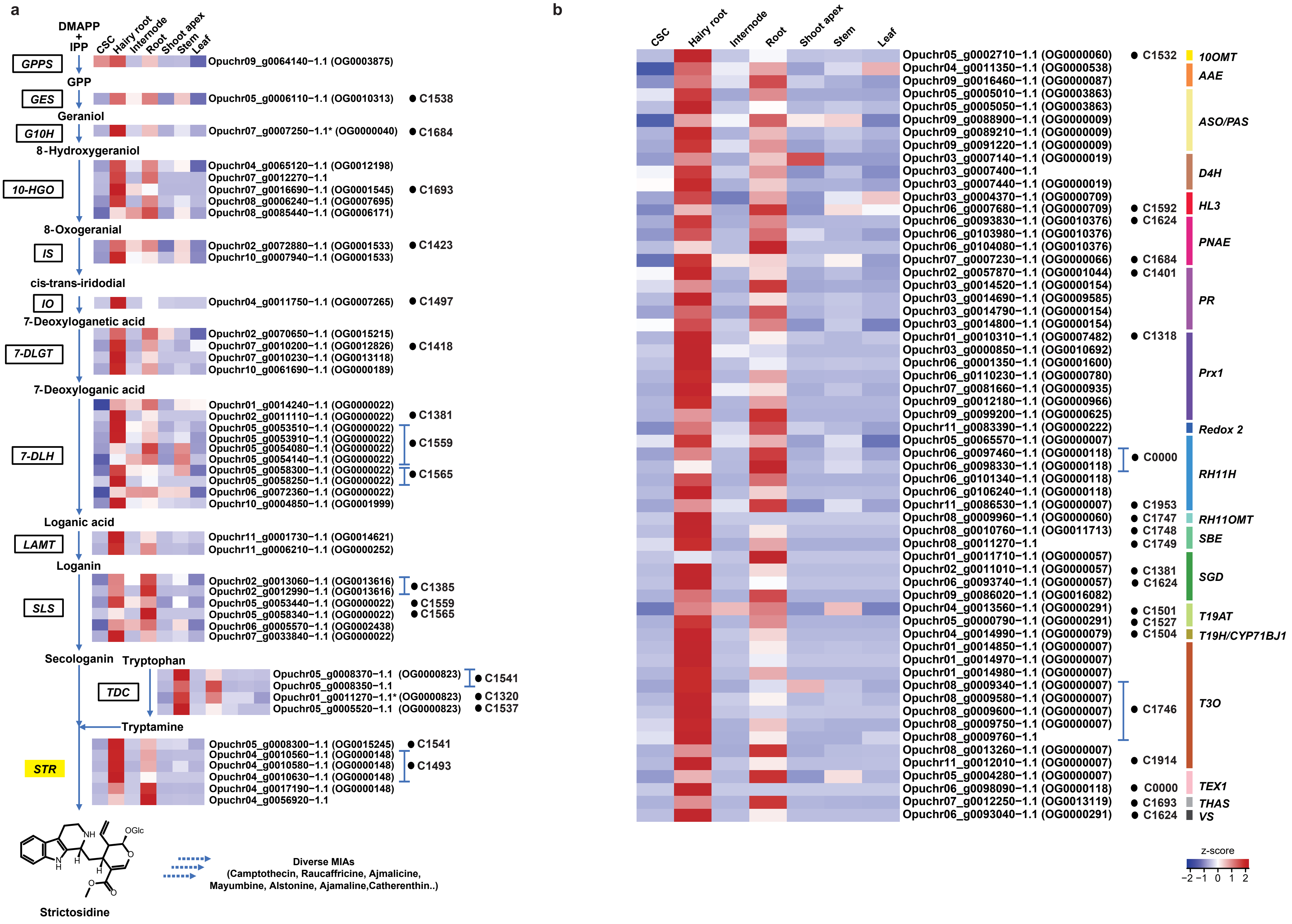
a
DMAPP
+
IPP
CSC
Hairy root
Internode
Root
Shoot apex
Stem
Leaf
GPPS
Opuchr09_g0064140−1.1 (OG0003875)
GPP
GES
Opuchr05_g0006110−1.1 (OG0010313)
● C1538
Geraniol
G10H
Opuchr07_g0007250−1.1* (OG0000040)
● C1684
8-Hydroxygeraniol
10-HGO
Opuchr04_g0065120−1.1 (OG0012198)
Opuchr07_g0012270−1.1
Opuchr07_g0016690−1.1 (OG0001545)
● C1693
Opuchr08_g0006240−1.1 (OG0007695)
Opuchr08_g0085440−1.1 (OG0006171)
8-Oxogeranial
IS
Opuchr02_g0072880−1.1 (OG0001533)
● C1423
Opuchr10_g0007940−1.1 (OG0001533)
cis-trans-iridodial
IO
Opuchr04_g0011750−1.1 (OG0007265)
● C1497
7-Deoxyloganetic acid
7-DLGT
Opuchr02_g0070650−1.1 (OG0015215)
Opuchr07_g0010200−1.1 (OG0012826)
● C1418
Opuchr07_g0010230−1.1 (OG0013118)
Opuchr10_g0061690−1.1 (OG0000189)
7-Deoxyloganic acid
7-DLH
Opuchr01_g0014240−1.1 (OG0000022)
Opuchr02_g0011110−1.1 (OG0000022)
● C1381
Opuchr05_g0053510−1.1 (OG0000022)
Opuchr05_g0053910−1.1 (OG0000022)
● C1559
Opuchr05_g0054080−1.1 (OG0000022)
Opuchr05_g0054140−1.1 (OG0000022)
Opuchr05_g0058300−1.1 (OG0000022)
● C1565
Opuchr05_g0058250−1.1 (OG0000022)
Opuchr06_g0072360−1.1 (OG0000022)
Opuchr10_g0004850−1.1 (OG0001999)
Loganic acid
LAMT
Opuchr11_g0001730−1.1 (OG0014621)
Opuchr11_g0006210−1.1 (OG0000252)
Loganin
SLS
Opuchr02_g0013060−1.1 (OG0013616)
● C1385
Opuchr02_g0012990−1.1 (OG0013616)
Opuchr05_g0053440−1.1 (OG0000022)
● C1559
Opuchr05_g0058340−1.1 (OG0000022)
● C1565
Opuchr06_g0005570−1.1 (OG0002438)
Opuchr07_g0033840−1.1 (OG0000022)
Secologanin
Tryptophan
TDC
Opuchr05_g0008370−1.1 (OG0000823)
● C1541
Opuchr05_g0008350−1.1
Opuchr01_g0011270−1.1* (OG0000823)
● C1320
Opuchr05_g0005520−1.1 (OG0000823)
● C1537
Tryptamine
STR
Opuchr05_g0008300−1.1 (OG0015245)
● C1541
Opuchr04_g0010560−1.1 (OG0000148)
Opuchr04_g0010580−1.1 (OG0000148)
● C1493
Opuchr04_g0010630−1.1 (OG0000148)
Opuchr04_g0017190−1.1 (OG0000148)
Opuchr04_g0056920−1.1
NH
OGlc
O
N
H
H
H
O
O
Strictosidine
Diverse MIAs
(Camptothecin, Raucaffricine, Ajmalicine,
Mayumbine, Alstonine, Ajamaline,Catherenthin..)
b
CSC
Hairy root
Internode
Root
Shoot apex
Stem
Leaf
Opuchr05_g0002710−1.1 (OG0000060) ● C1532 10OMT
Opuchr04_g0011350−1.1 (OG0000538) AAE
Opuchr09_g0016460−1.1 (OG0000087)
Opuchr05_g0005010−1.1 (OG0003863)
Opuchr05_g0005050−1.1 (OG0003863)
Opuchr09_g0088900−1.1 (OG0000009) ASO/PAS
Opuchr09_g0089210−1.1 (OG0000009)
Opuchr09_g0091220−1.1 (OG0000009)
Opuchr03_g0007140−1.1 (OG0000019)
Opuchr03_g0007400−1.1 D4H
Opuchr03_g0007440−1.1 (OG0000019)
Opuchr03_g0004370−1.1 (OG0000709)
Opuchr06_g0007680−1.1 (OG0000709) ● C1592 HL3
Opuchr06_g0093830−1.1 (OG0010376) ● C1624
Opuchr06_g0103980−1.1 (OG0010376) PNAE
Opuchr06_g0104080−1.1 (OG0010376)
Opuchr07_g0007230−1.1 (OG0000066) ● C1684
Opuchr02_g0057870−1.1 (OG0001044) ● C1401
Opuchr03_g0014520−1.1 (OG0000154)
Opuchr03_g0014690−1.1 (OG0009585) PR
Opuchr03_g0014790−1.1 (OG0000154)
Opuchr03_g0014800−1.1 (OG0000154)
Opuchr01_g0010310−1.1 (OG0007482) ● C1318
Opuchr03_g0000850−1.1 (OG0010692)
Opuchr06_g0001350−1.1 (OG0001600)
Opuchr06_g0110230−1.1 (OG0000780) Prx1
Opuchr07_g0081660−1.1 (OG0000935)
Opuchr09_g0012180−1.1 (OG0000966)
Opuchr09_g0099200−1.1 (OG0000625)
Opuchr11_g0083390−1.1 (OG0000222) Redox 2
Opuchr05_g0065570−1.1 (OG0000007)
Opuchr06_g0097460−1.1 (OG0000118) ● C0000
Opuchr06_g0098330−1.1 (OG0000118)
Opuchr06_g0101340−1.1 (OG0000118) RH11H
Opuchr06_g0106240−1.1 (OG0000118)
Opuchr11_g0086530−1.1 (OG0000007) ● C1953
Opuchr08_g0009960−1.1 (OG0000060) ● C1747 RH11OMT
Opuchr08_g0010760−1.1 (OG0011713) ● C1748 SBE
Opuchr08_g0011270−1.1 ● C1749
Opuchr01_g0011710−1.1 (OG0000057)
Opuchr02_g0011010−1.1 (OG0000057) ● C1381 SGD
Opuchr06_g0093740−1.1 (OG0000057) ● C1624
Opuchr09_g0086020−1.1 (OG0016082)
Opuchr04_g0013560−1.1 (OG0000291) ● C1501 T19AT
Opuchr05_g0000790−1.1 (OG0000291) ● C1527
Opuchr04_g0014990−1.1 (OG0000079) ● C1504 T19H/CYP71BJ1
Opuchr01_g0014850−1.1 (OG0000007)
Opuchr01_g0014970−1.1 (OG0000007)
Opuchr01_g0014980−1.1 (OG0000007)
Opuchr08_g0009340−1.1 (OG0000007) T3O
Opuchr08_g0009580−1.1 (OG0000007) ● C1746
Opuchr08_g0009600−1.1 (OG0000007)
Opuchr08_g0009750−1.1 (OG0000007)
Opuchr08_g0009760−1.1
Opuchr08_g0013260−1.1 (OG0000007)
Opuchr11_g0012010−1.1 (OG0000007) ● C1914
Opuchr05_g0004280−1.1 (OG0000007)
Opuchr06_g0098090−1.1 (OG0000118) ● C0000 TEX1
Opuchr07_g0012250−1.1 (OG0013119) ● C1693 THAS
Opuchr06_g0093040−1.1 (OG0000291) ● C1624 VS
z-score
−2 −1 0 1 2

Supplementary Fig. 26. Expression of genes assigned to secoiridoid and monoterpene indole alkaloid (MIA) biosynthesis pathway across seven tissues of *Ophiorrhiza pumila*. Expression of genes assigned to secoiridoid biosynthesis pathway **(a)** and MIA biosynthesis **(b)** across seven tissues of *O. pumila*. The color code used for individual genes corresponds to their position in MIA biosynthesis pathway in Supplementary Fig. 1. Genes were assigned to respective biosynthesis pathways based on co-expression and sequence homology against previously characterized genes. Cell suspension culture (CSC) and hairy root tissues are known to accumulate no and high MIA, respectively. Gene expression analysis showed tissue specific expression of genes, which were correlated with accumulation of MIA in *Ophiorrhiza* tissues. Associated orthogene families (OGs) and gene-cluster IDs are provided together with the gene IDs. Abbreviations- *GPPS*: geranyl diphosphate synthase; *GES*: geraniol synthase; *G10H*: geraniol 10-hydroxylase; *10-HGO*: 10- hydroxygeraniol oxidoreductase; *IS*: iridoid synthase; *IO*: iridoid oxidase; *7-DLGT*: 7-deoxyloganetin glucosyltransferase, *7-DLH*: 7-deoxyloganic acid 7-hydroxylase, *LAMT*: loganic acid O-methyltransferase; *SLS*: secologanin synthase; *TDC*: tryptophan decarboxylase; *STR*: strictosidine synthase; *SGD*: strictosidine beta-D-glucosidase; *THAS*: tetrahydroalstonine synthase; *PNAE*: polyneuridine- aldehyde esterase; *ASO/PAS*: O-acetylstemmadenine oxidase; *AAE*: acetylajmaline esterase; *TEX1*: tabersonine 6,7-epoxidase 1; *HL3*: hydrolase 3; *10OMT*: 10- hydroxycamptothecin O-methyltransferase; *T19H*: tabersonine-19-hydroxy-O-acetyltransferase; *RH11H*: rankinidine/ humantenine-11-hydroxylase 3; *D4H*: deacetoxyvindoline 4-hydroxylase; *Prx1*: peroxidase 1; *PR*: perakine reductase. Source data are provided as Source Data file.

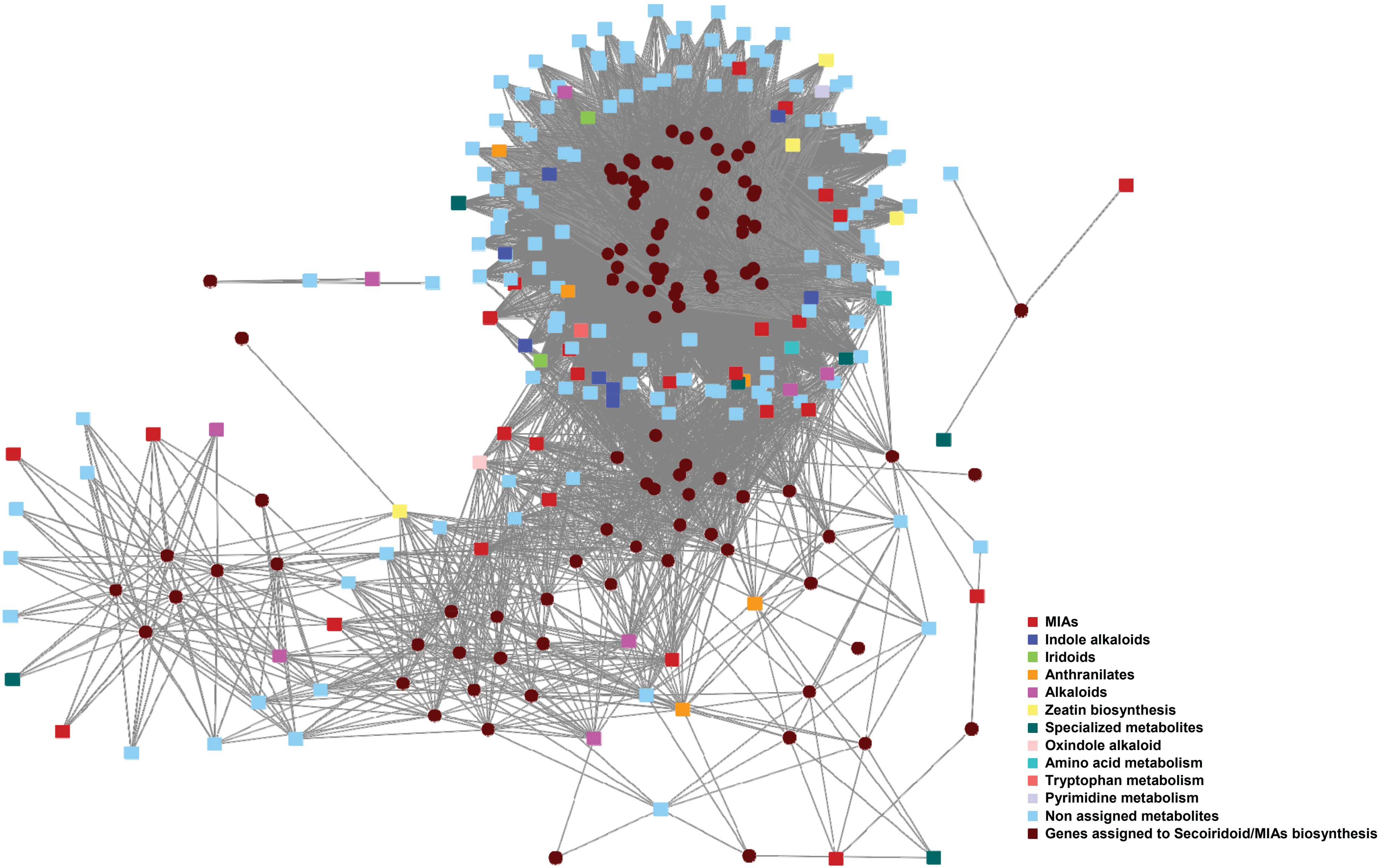

Supplementary Fig. 27. Gene-to-metabolite relationship network associated with MIA biosynthesis. The Pearson's correlation matrix was calculated using the psych package in R and drawn using Cytoscape (v3.6.0). The circle and square nodes represent genes assigned to secoiridoid and MIA biosynthesis pathways (shown in the Supplementary Fig. 26), and the metabolites, respectively. Edges represent gene-metabolite pairs with paired Pearson Correlation coefficient scores over 0.7 and corrected *p*-value < 0.05. Source data are provided as a Source Data file.

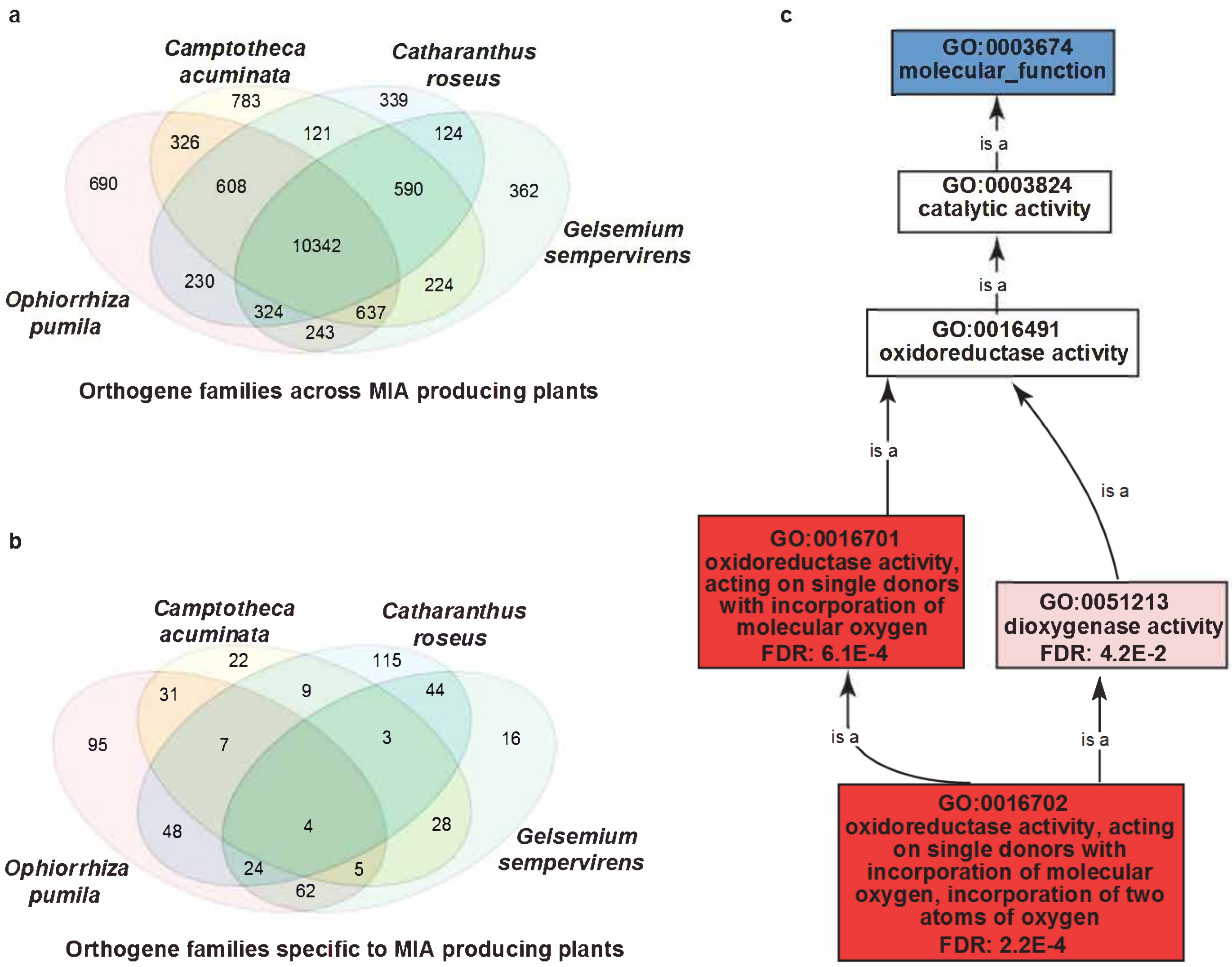

Supplementary Fig. 28. Orthogene families across monoterpene indole alkaloid (MIA) producing plant species. OrthoFinder based gene family classification was performed for 13 plant species, and orthogenes assigned to MIA producing plants were used for comparison. **(a)** Orthogenes shared among four MIA producing plant species. **(b)** Orthogenes specific to MIA producing plants. **(c)** Gene ontology enrichment analysis for *Ophiorrhiza pumila* genes assigned to MIA specific orthogene families as test set and *O. pumila* genome as reference set. Statistical significance was estimated using Fisher's Exact test (two-sided) with the corrected *p*-value cutoff set as 0.05. GO enrichment graph for statistically significant GO terms were drawn using Omics box software.

a **Orthogene family OG0000148**

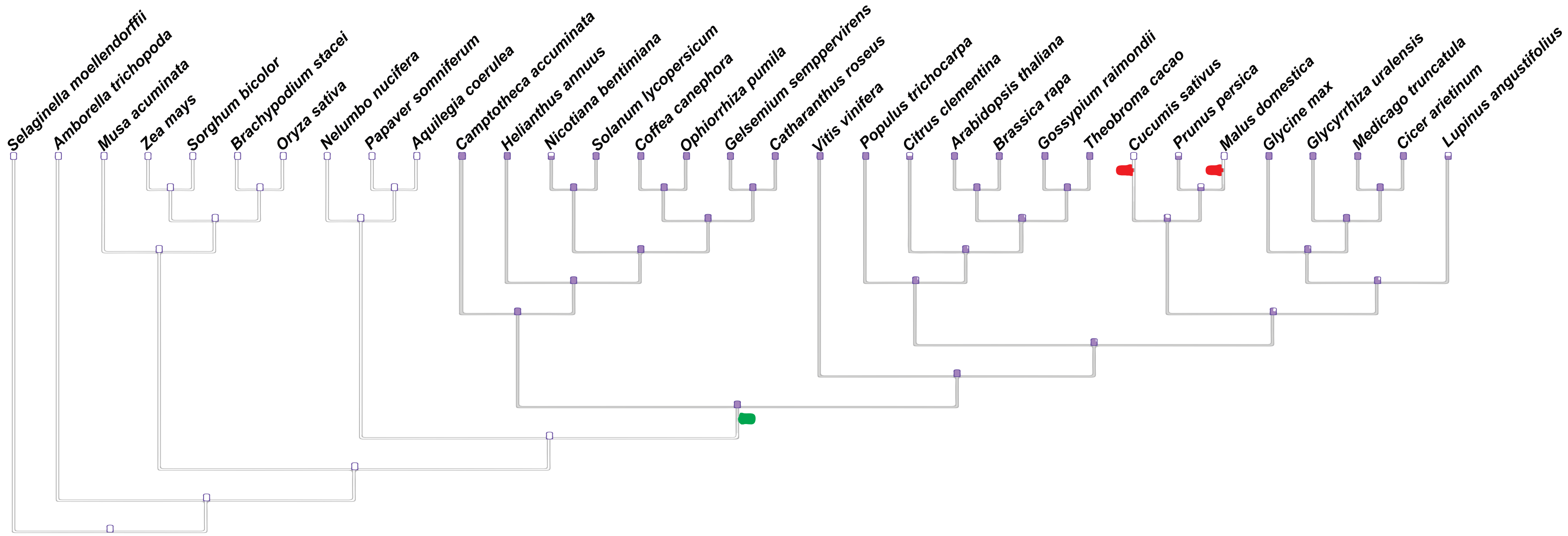

b **Orthogene family OG0015245**

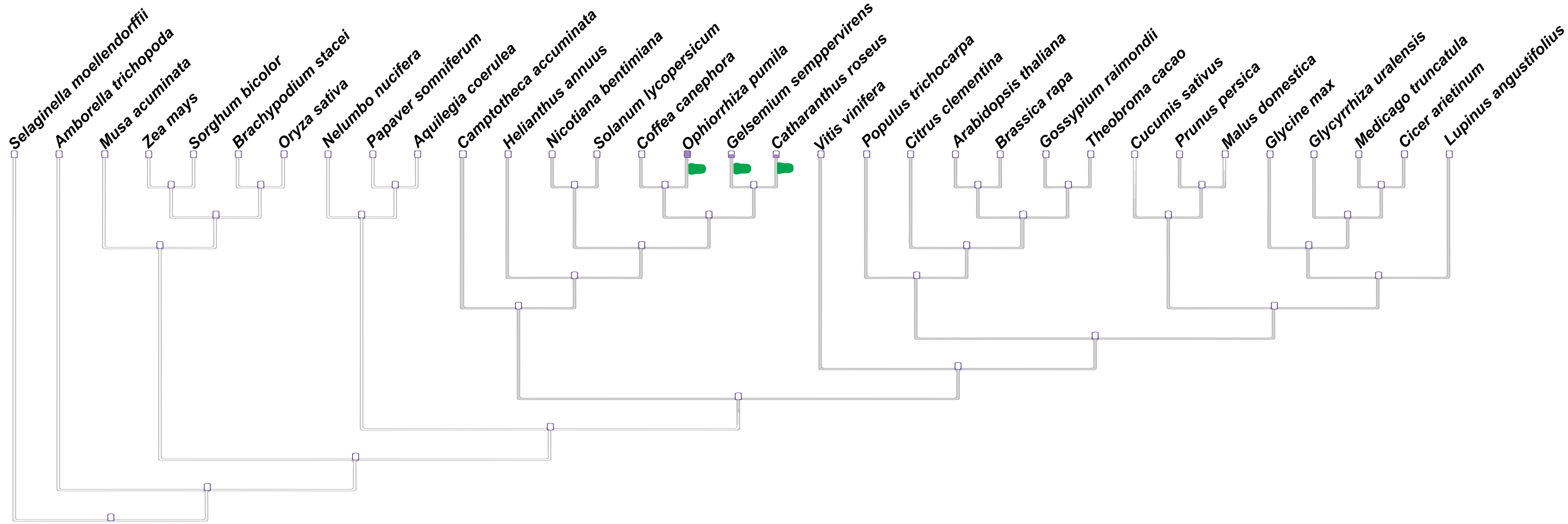

Supplementary Fig. 29. Gain, loss, expansion, or contraction for gene families annotated as strictosidine synthase (STR) across 33 plant species. (a) Orthogene family OG0000148 representing genes annotated as STR and consisting of member genes across multiple plant species. **(b)** Orthogene family OG0015245 consisting of functionally characterized STRs and specifically gained in the strictosidine derived monoterpene indole alkaloids producing plant species. Evolutionary analysis of gene content was performed using COUNT software. Phylogenetic tree and orthogene classifications were performed as described in the material and method section. Empty rectangles at the nodes denote the absence of gene from an ancestral node, shaded node denotes presence. The green bar represents gene gain or expansion, while the red bar represents gene loss or contraction at the node.

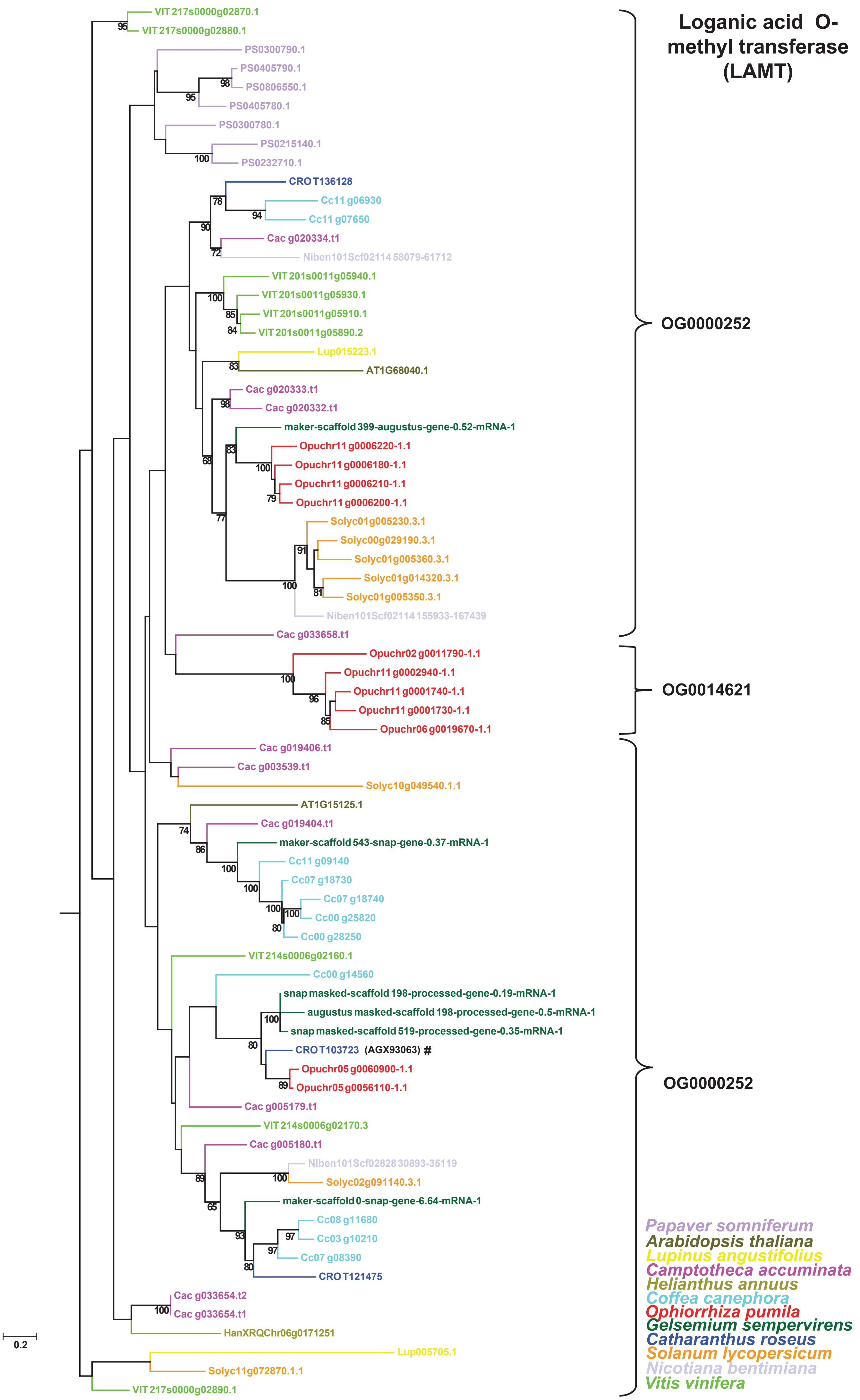
Loganic acid O-methyl transferase (LAMT)
OG0000252
OG0014621
OG0000252
VIT217s0000g02870.1
VIT217s0000g02880.1
PS0300790.1
PS0405790.1
PS0806550.1
PS0405780.1
PS0300780.1
PS0215140.1
PS0232710.1
CROT136128
Cc11g06930
Cc11g07650
Cacg020334.t1
Niben101Scf02114 58079-61712
VIT201s0011g05940.1
VIT201s0011g05930.1
VIT201s0011g05910.1
VIT201s0011g05890.2
Lup015223.1
AT1G68040.1
Cacg020333.t1
Cacg020332.t1
maker-scaffold399-augustus-gene-0.52-mRNA-1
Opuchr11g0006220-1.1
Opuchr11g0006180-1.1
Opuchr11g0006210-1.1
Opuchr11g0006200-1.1
Solyc01g005230.3.1
Solyc00g029190.3.1
Solyc01g005360.3.1
Solyc01g014320.3.1
Solyc01g005350.3.1
Niben101Scf02114 155933-167439
Cacg033658.t1
Opuchr02g0011790-1.1
Opuchr11g0002940-1.1
Opuchr11g0001740-1.1
Opuchr11g0001730-1.1
Opuchr06g0019670-1.1
Cacg019406.t1
Cacg003539.t1
Solyc10g049540.1.1
AT1G15125.1
Cacg019404.t1
maker-scaffold543-snap-gene-0.37-mRNA-1
Cc11g09140
Cc07g18730
Cc07g18740
Cc00g25820
Cc00g28250
VIT214s0006g02160.1
Cc00g14560
snapmasked-scaffold198-processed-gene-0.19-mRNA-1
augustusmasked-scaffold198-processed-gene-0.5-mRNA-1
snapmasked-scaffold519-processed-gene-0.35-mRNA-1
CROT103723 (AGX93063) #
Opuchr05g0060900-1.1
Opuchr05g0056110-1.1
Cacg005179.t1
VIT214s0006g02170.3
Cacg005180.t1
Niben101Scf02828 30893-35119
Solyc02g091140.3.1
maker-scaffold0-snap-gene-6.64-mRNA-1
Cc08g11680
Cc03g10210
Cc07g08390
CROT121475
Cacg033654.t2
Cacg033654.t1
HanXRQChr06g0171251
Lup005705.1
Solyc11g072870.1.1
VIT217s0000g02890.1
0.2
Papaver somniferum
Arabidopsis thaliana
Lupinus angustifolius
Camptotheca accuminata
Helianthus annuus
Coffea canephora
Ophiorrhiza pumila
Gelsemium sempervirens
Catharanthus roseus
Solanum lycopersicum
Nicotiana bentimiana
Vitis vinifera

Supplementary Fig. 30. Phylogenetic analysis of loganic acid O-methyltransferase (LAMT) assigned to the orthogene families, OG0000252 and OG0014621. Gene family classification was performed based on 13 plant genomes, and the genes are colored based on the associated plant species. Functionally characterized genes are represented by "#" with the GenBank accession IDs. Strongly supported nodes with bootstrap value above 60% are shown.

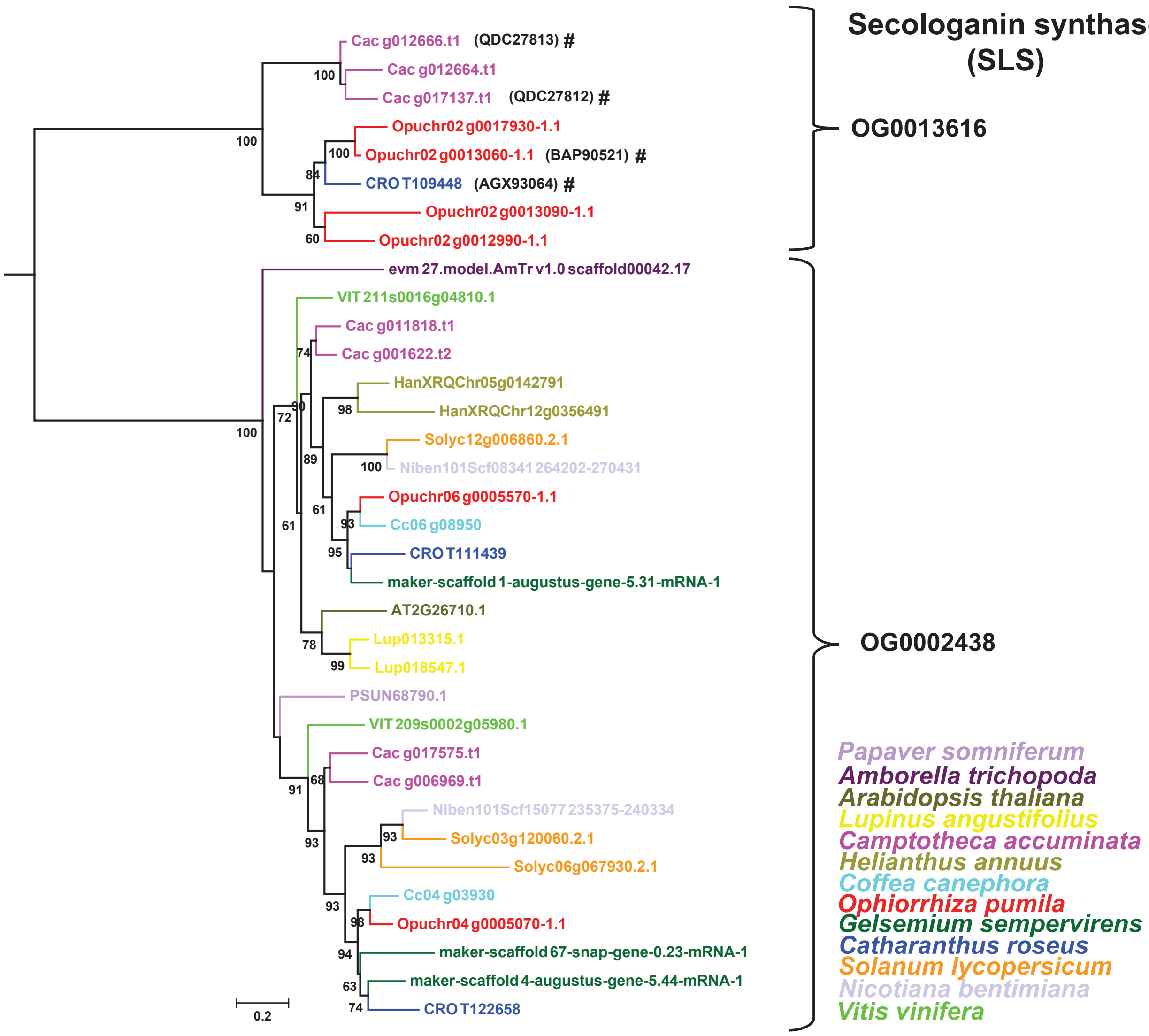

Supplementary Fig. 31. Phylogenetic analysis of secologanin synthase (SLS) assigned to the orthogene families, OG0002438 and OG0013616. Gene family classification was performed based on 13 plant genomes, and the genes are colored based on the associated plant species. Functionally characterized genes are represented by "#" with the GenBank accession IDs. Strongly supported nodes with bootstrap value above 60% are shown.

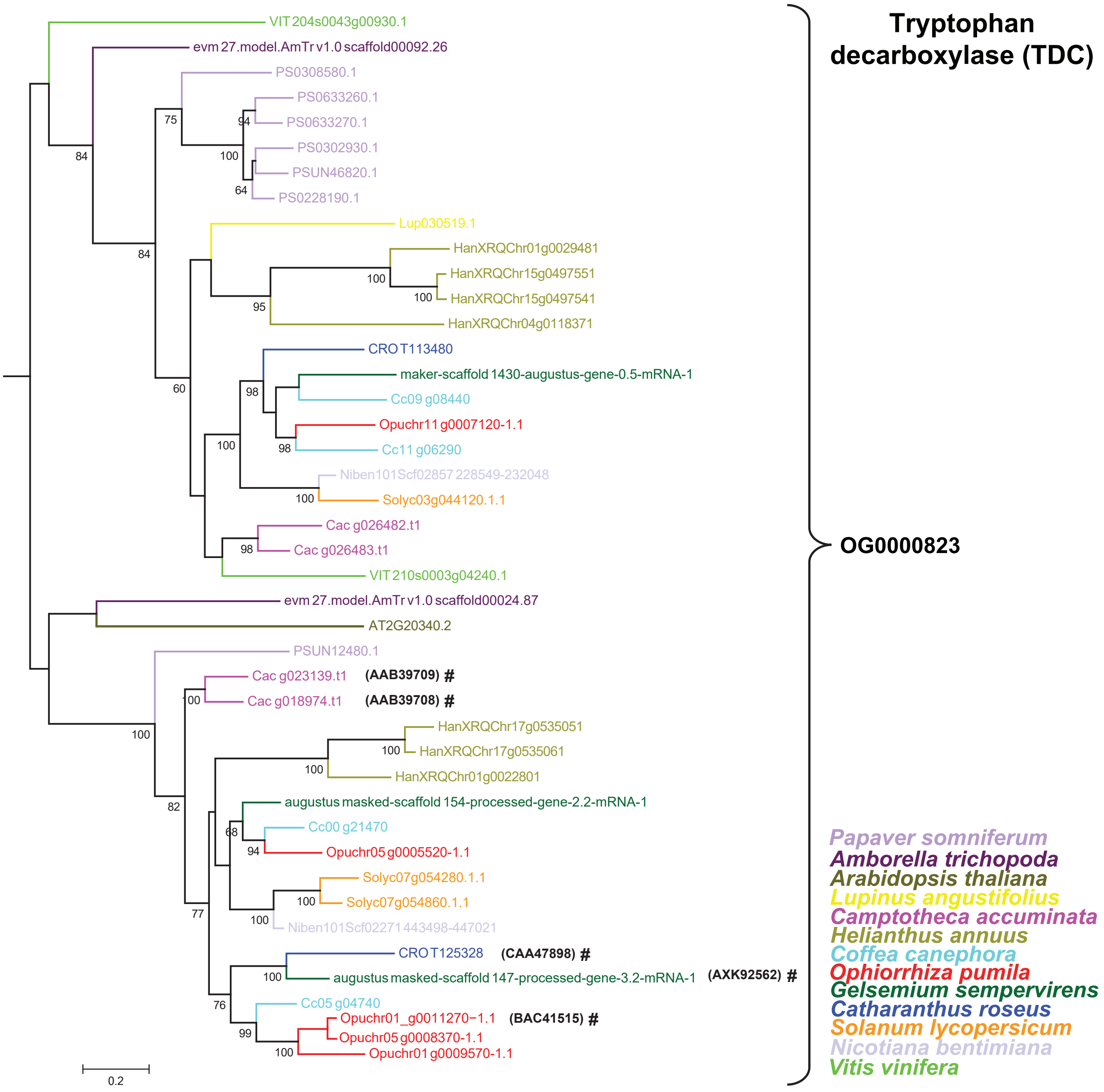

Supplementary Fig. 32. Phylogenetic analysis of tryptophan decarboxylase (TDC) assigned to the orthogene family OG0000823. Gene family classification was performed based on 13 plant genomes, and the genes are colored based on the associated plant species. Functionally characterized genes are represented by "#" with the GenBank accession IDs. Strongly supported nodes with bootstrap value above 60% are shown.

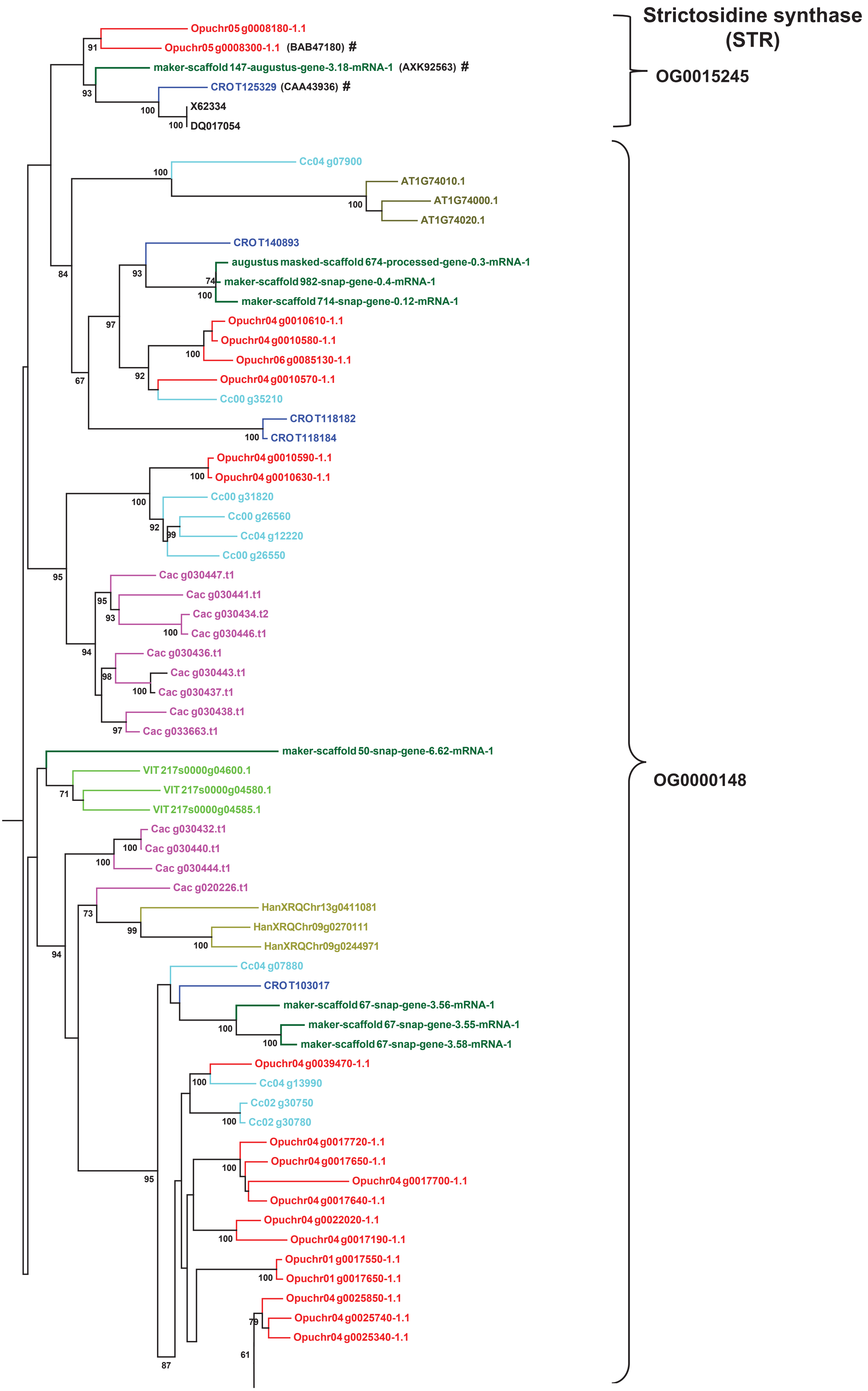
Strictosidine synthase (STR)
OG0015245
OG0000148
Opuchr05 g0008180-1.1
Opuchr05 g0008300-1.1 (BAB47180) #
maker-scaffold 147-augustus-gene-3.18-mRNA-1 (AXK92563) #
CRO T125329 (CAA43936) #
X62334
DQ017054
Cc04 g07900
AT1G74010.1
AT1G74000.1
AT1G74020.1
CRO T140893
augustus masked-scaffold 674-processed-gene-0.3-mRNA-1
maker-scaffold 982-snap-gene-0.4-mRNA-1
maker-scaffold 714-snap-gene-0.12-mRNA-1
Opuchr04 g0010610-1.1
Opuchr04 g0010580-1.1
Opuchr06 g0085130-1.1
Opuchr04 g0010570-1.1
Cc00 g35210
CRO T118182
CRO T118184
Opuchr04 g0010590-1.1
Opuchr04 g0010630-1.1
Cc00 g31820
Cc00 g26560
Cc04 g12220
Cc00 g26550
Cac g030447.t1
Cac g030441.t1
Cac g030434.t2
Cac g030446.t1
Cac g030436.t1
Cac g030443.t1
Cac g030437.t1
Cac g030438.t1
Cac g033663.t1
maker-scaffold 50-snap-gene-6.62-mRNA-1
VIT 217s0000g04600.1
VIT 217s0000g04580.1
VIT 217s0000g04585.1
Cac g030432.t1
Cac g030440.t1
Cac g030444.t1
Cac g020226.t1
HanXRQChr13g0411081
HanXRQChr09g0270111
HanXRQChr09g0244971
Cc04 g07880
CRO T103017
maker-scaffold 67-snap-gene-3.56-mRNA-1
maker-scaffold 67-snap-gene-3.55-mRNA-1
maker-scaffold 67-snap-gene-3.58-mRNA-1
Opuchr04 g0039470-1.1
Cc04 g13990
Cc02 g30750
Cc02 g30780
Opuchr04 g0017720-1.1
Opuchr04 g0017650-1.1
Opuchr04 g0017700-1.1
Opuchr04 g0017640-1.1
Opuchr04 g0022020-1.1
Opuchr04 g0017190-1.1
Opuchr01 g0017550-1.1
Opuchr01 g0017650-1.1
Opuchr04 g0025850-1.1
Opuchr04 g0025740-1.1
Opuchr04 g0025340-1.1

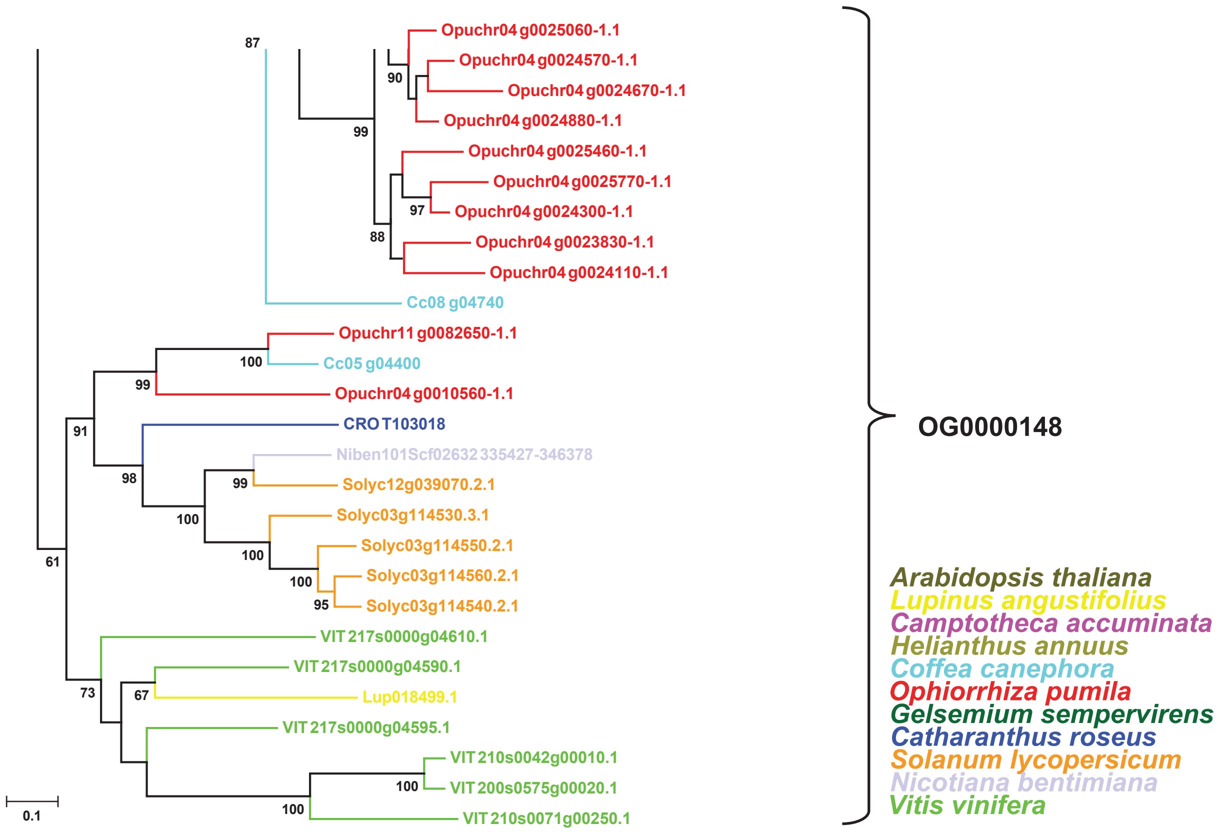

Supplementary Fig. 33. Phylogenetic analysis of strictosidine synthase (STR) assigned to the orthogene families, OG0000148 and OG0015245. Gene family classification was performed based on 13 plant genomes, and the genes are colored based on the associated plant species. Functionally characterized genes are represented by "#" with the GenBank accession IDs. Strongly supported nodes with bootstrap value above 60% are shown.

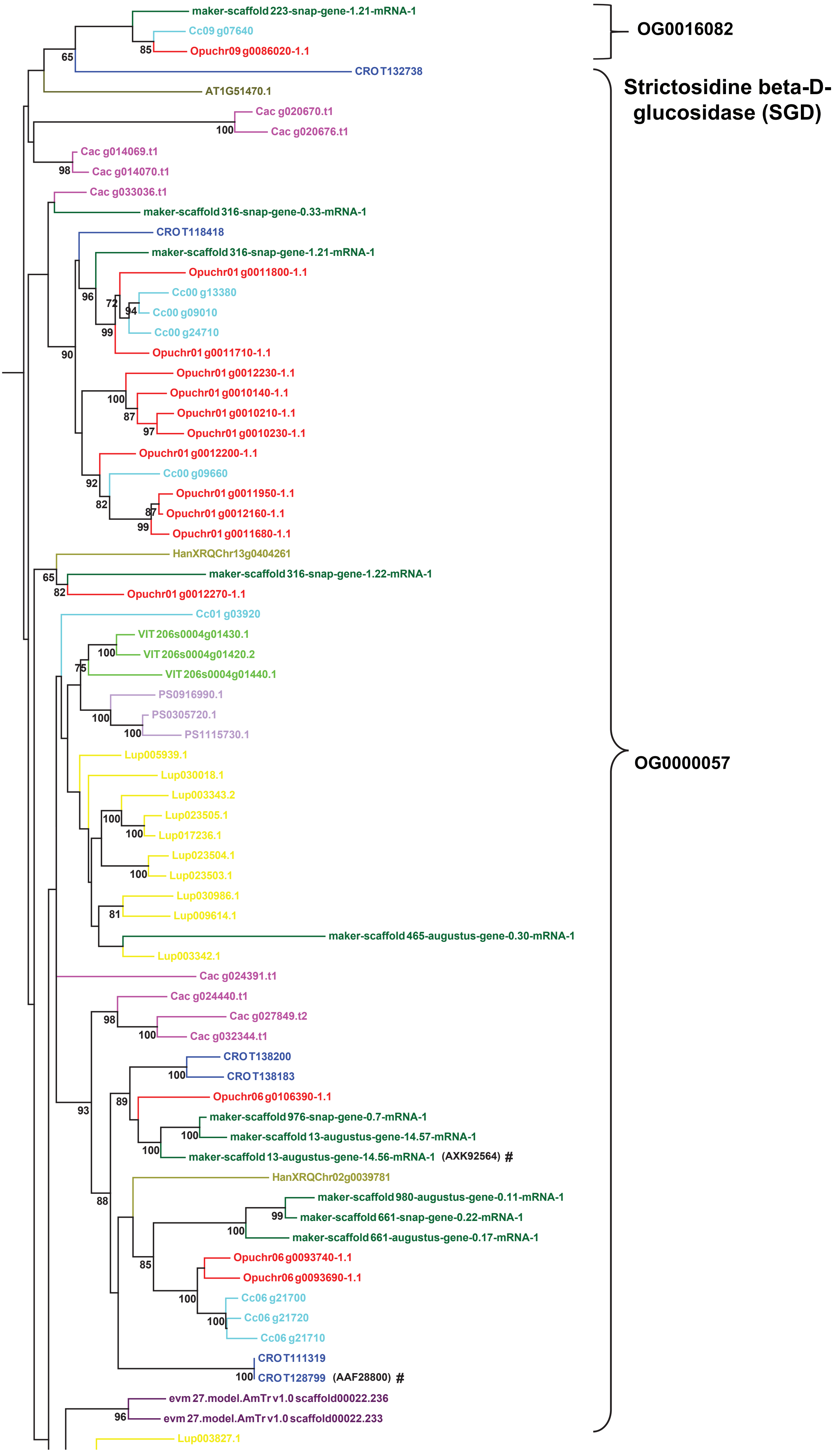

OG0016082
Strictosidine beta-D-glucosidase (SGD)
OG0000057
maker-scaffold223-snap-gene-1.21-mRNA-1
Cc09g07640
Opuchr09g0086020-1.1
CROT132738
AT1G51470.1
Cacg020670.t1
Cacg020676.t1
Cacg014069.t1
Cacg014070.t1
Cacg033036.t1
maker-scaffold316-snap-gene-0.33-mRNA-1
CROT118418
maker-scaffold316-snap-gene-1.21-mRNA-1
Opuchr01g0011800-1.1
Cc00g13380
Cc00g09010
Cc00g24710
Opuchr01g0011710-1.1
Opuchr01g0012230-1.1
Opuchr01g0010140-1.1
Opuchr01g0010210-1.1
Opuchr01g0010230-1.1
Opuchr01g0012200-1.1
Cc00g09660
Opuchr01g0011950-1.1
Opuchr01g0012160-1.1
Opuchr01g0011680-1.1
HanXRQChr13g0404261
maker-scaffold316-snap-gene-1.22-mRNA-1
Opuchr01g0012270-1.1
Cc01g03920
VIT206s0004g01430.1
VIT206s0004g01420.2
VIT206s0004g01440.1
PS0916990.1
PS0305720.1
PS1115730.1
Lup005939.1
Lup030018.1
Lup003343.2
Lup023505.1
Lup017236.1
Lup023504.1
Lup023503.1
Lup030986.1
Lup009614.1
maker-scaffold465-augustus-gene-0.30-mRNA-1
Lup003342.1
Cacg024391.t1
Cacg024440.t1
Cacg027849.t2
Cacg032344.t1
CROT138200
CROT138183
Opuchr06g0106390-1.1
maker-scaffold976-snap-gene-0.7-mRNA-1
maker-scaffold13-augustus-gene-14.57-mRNA-1
maker-scaffold13-augustus-gene-14.56-mRNA-1 (AXK92564) #
HanXRQChr02g0039781
maker-scaffold980-augustus-gene-0.11-mRNA-1
maker-scaffold661-snap-gene-0.22-mRNA-1
maker-scaffold661-augustus-gene-0.17-mRNA-1
Opuchr06g0093740-1.1
Opuchr06g0093690-1.1
Cc06g21700
Cc06g21720
Cc06g21710
CROT111319
CROT128799 (AAF28800) #
evm27.model.AmTrv1.0scaffold00022.236
evm27.model.AmTrv1.0scaffold00022.233
Lup003827.1
65
85
100
98
96
72
94
99
90
100
87
97
92
82
87
99
65
82
100
75
100
100
100
100
100
81
98
100
100
93
89
100
100
88
99
100
85
100
100
100
96

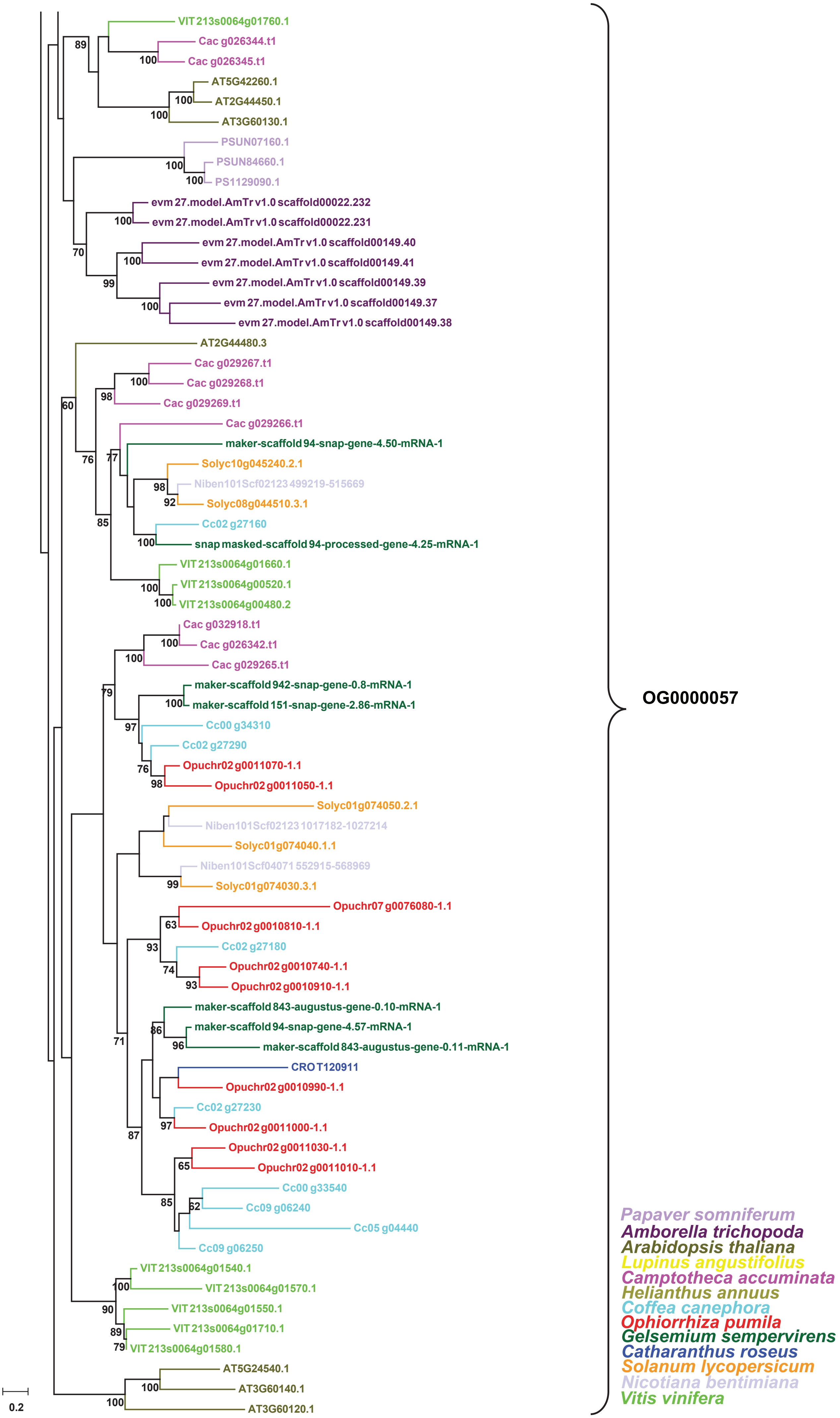
VIT213s0064g01760.1
Cac g026344.t1
Cac g026345.t1
AT5G42260.1
AT2G44450.1
AT3G60130.1
PSUN07160.1
PSUN84660.1
PS1129090.1
evm27.model.AmTrv1.0scaffold00022.232
evm27.model.AmTrv1.0scaffold00022.231
evm27.model.AmTrv1.0scaffold00149.40
evm27.model.AmTrv1.0scaffold00149.41
evm27.model.AmTrv1.0scaffold00149.39
evm27.model.AmTrv1.0scaffold00149.37
evm27.model.AmTrv1.0scaffold00149.38
AT2G44480.3
Cac g029267.t1
Cac g029268.t1
Cac g029269.t1
Cac g029266.t1
maker-scaffold94-snap-gene-4.50-mRNA-1
Solyc10g045240.2.1
Niben101Scf02123 499219-515669
Solyc08g044510.3.1
Cc02 g27160
snap masked-scaffold94-processed-gene-4.25-mRNA-1
VIT213s0064g01660.1
VIT213s0064g00520.1
VIT213s0064g00480.2
Cac g032918.t1
Cac g026342.t1
Cac g029265.t1
maker-scaffold942-snap-gene-0.8-mRNA-1
maker-scaffold151-snap-gene-2.86-mRNA-1
Cc00 g34310
Cc02 g27290
Opuchr02 g0011070-1.1
Opuchr02 g0011050-1.1
Solyc01g074050.2.1
Niben101Scf02123 1017182-1027214
Solyc01g074040.1.1
Niben101Scf04071 552915-568969
Solyc01g074030.3.1
Opuchr07 g0076080-1.1
Opuchr02 g0010810-1.1
Cc02 g27180
Opuchr02 g0010740-1.1
Opuchr02 g0010910-1.1
maker-scaffold843-augustus-gene-0.10-mRNA-1
maker-scaffold94-snap-gene-4.57-mRNA-1
maker-scaffold843-augustus-gene-0.11-mRNA-1
CROT120911
Opuchr02 g0010990-1.1
Cc02 g27230
Opuchr02 g0011000-1.1
Opuchr02 g0011030-1.1
Opuchr02 g0011010-1.1
Cc00 g33540
Cc09 g06240
Cc05 g04440
Cc09 g06250
VIT213s0064g01540.1
VIT213s0064g01570.1
VIT213s0064g01550.1
VIT213s0064g01710.1
VIT213s0064g01580.1
AT5G24540.1
AT3G60140.1
AT3G60120.1
0.2
OG0000057
Papaver somniferum
Amborella trichopoda
Arabidopsis thaliana
Lupinus angustifolius
Camptotheca accuminata
Helianthus annuus
Coffea canephora
Ophiorrhiza pumila
Gelsemium sempervirens
Catharanthus roseus
Solanum lycopersicum
Nicotiana bentimiana
Vitis vinifera

Supplementary Fig. 34. Phylogenetic analysis of strictosidine beta-D-glucosidase (SGD) assigned to the orthogene families, OG0000057 and OG0016082. Gene family classification was performed based on 13 plant genomes, and the genes are colored based on the associated plant species. Functionally characterized genes are represented by "#" with the GenBank accession IDs. Strongly supported nodes with bootstrap value above 60% are shown.

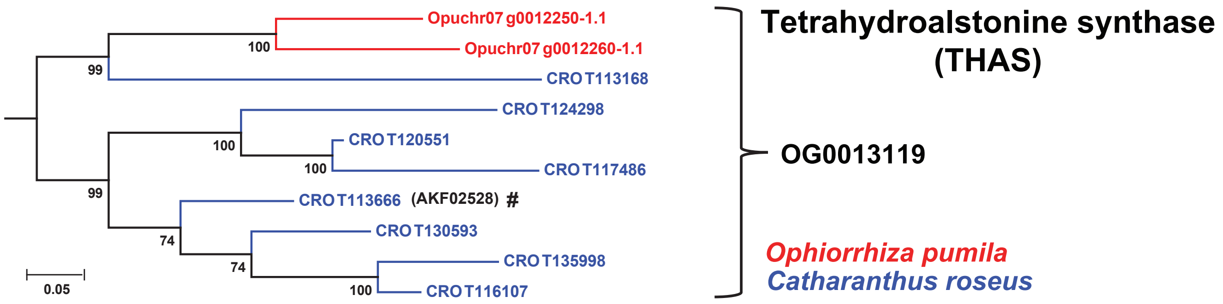

Supplementary Fig. 35. Phylogenetic analysis of tetrahydroalstonine synthase (THAS) assigned to the orthogene family, OG0013119. Gene family classification was performed based on 13 plant genomes, and the genes are colored based on the associated plant species. Functionally characterized genes are represented by "#" with the GenBank accession IDs. Strongly supported nodes with bootstrap value above 60% are shown.

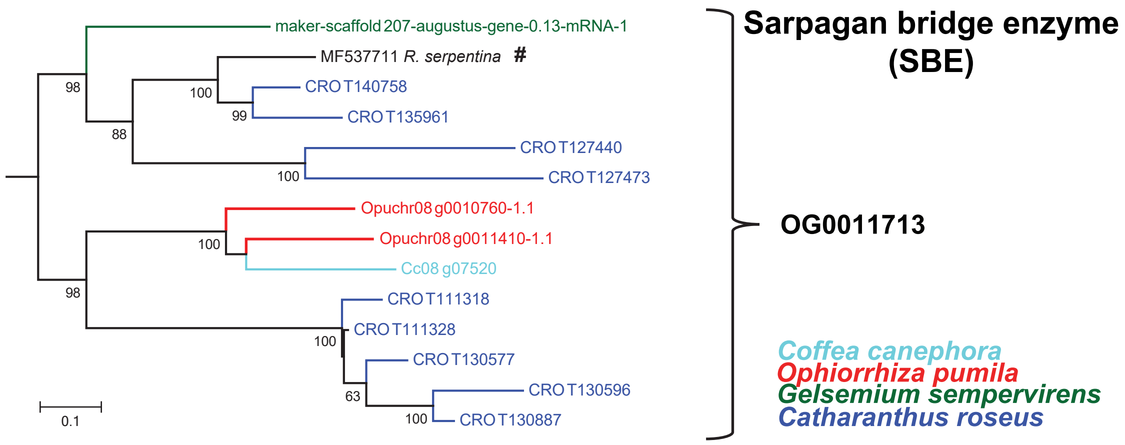

Supplementary Fig. 36. Phylogenetic analysis of sarpagan bridge enzyme (SBE) assigned to the orthogene family, OG0011713. Gene family classification was performed based on 13 plant genomes, and the genes are colored based on the associated plant species. Functionally characterized genes are represented by "#" with the GenBank accession IDs. Strongly supported nodes with bootstrap value above 60% are shown.

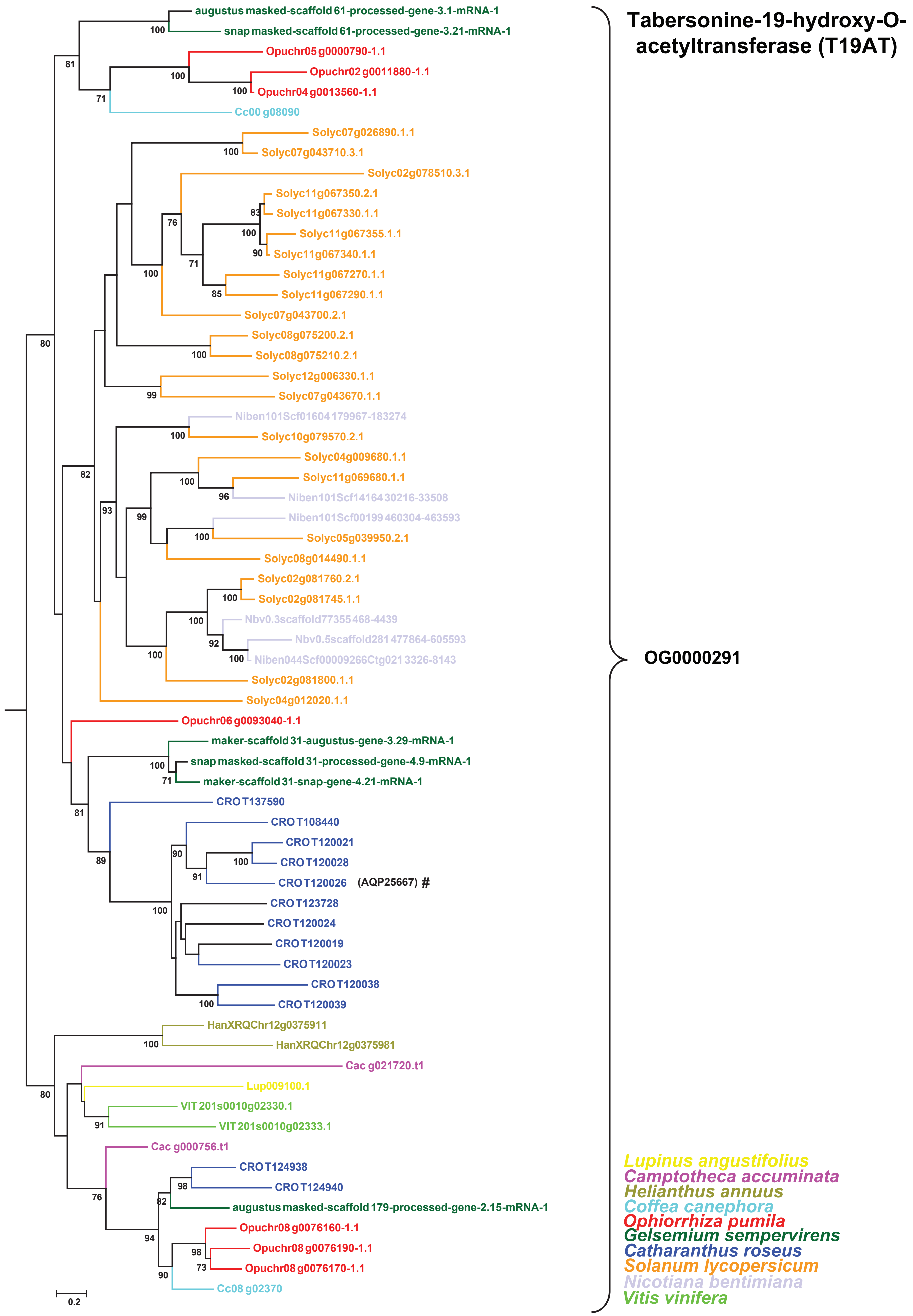
Tabersonine-19-hydroxy-O-acetyltransferase (T19AT)
OG0000291
augustus masked-scaffold 61-processed-gene-3.1-mRNA-1
snap masked-scaffold 61-processed-gene-3.21-mRNA-1
Opuchr05 g0000790-1.1
Opuchr02 g0011880-1.1
Opuchr04 g0013560-1.1
Cc00 g08090
Solyc07g026890.1.1
Solyc07g043710.3.1
Solyc02g078510.3.1
Solyc11g067350.2.1
Solyc11g067330.1.1
Solyc11g067355.1.1
Solyc11g067340.1.1
Solyc11g067270.1.1
Solyc11g067290.1.1
Solyc07g043700.2.1
Solyc08g075200.2.1
Solyc08g075210.2.1
Solyc12g006330.1.1
Solyc07g043670.1.1
Niben101Scf01604 179967-183274
Solyc10g079570.2.1
Solyc04g009680.1.1
Solyc11g069680.1.1
Niben101Scf14164 30216-33508
Niben101Scf00199 460304-463593
Solyc05g039950.2.1
Solyc08g014490.1.1
Solyc02g081760.2.1
Solyc02g081745.1.1
Nbv0.3scaffold77355 468-4439
Nbv0.5scaffold281 477864-605593
Niben044Scf00009266Ctg021 3326-8143
Solyc02g081800.1.1
Solyc04g012020.1.1
Opuchr06 g0093040-1.1
maker-scaffold 31-augustus-gene-3.29-mRNA-1
snap masked-scaffold 31-processed-gene-4.9-mRNA-1
maker-scaffold 31-snap-gene-4.21-mRNA-1
CRO T137590
CRO T108440
CRO T120021
CRO T120028
CRO T120026 (AQP25667) #
CRO T123728
CRO T120024
CRO T120019
CRO T120023
CRO T120038
CRO T120039
HanXRQChr12g0375911
HanXRQChr12g0375981
Cac g021720.t1
Lup009100.1
VIT 201s0010g02330.1
VIT 201s0010g02333.1
Cac g000756.t1
CRO T124938
CRO T124940
augustus masked-scaffold 179-processed-gene-2.15-mRNA-1
Opuchr08 g0076160-1.1
Opuchr08 g0076190-1.1
Opuchr08 g0076170-1.1
Cc08 g02370
0.2
Lupinus angustifolius
Camptotheca accuminata
Helianthus annuus
Coffea canephora
Ophiorrhiza pumila
Gelsemium sempervirens
Catharanthus roseus
Solanum lycopersicum
Nicotiana bentimiana
Vitis vinifera

Supplementary Fig. 37. Phylogenetic analysis of tabersonine-19-hydroxy-O-acetyltransferase (T19AT) assigned to the orthogene family, OG0000291. Gene family classification was performed based on 13 plant genomes, and the genes are colored based on the associated plant species. Functionally characterized genes are represented by "#" with the GenBank accession IDs. Strongly supported nodes with bootstrap value above 60% are shown.

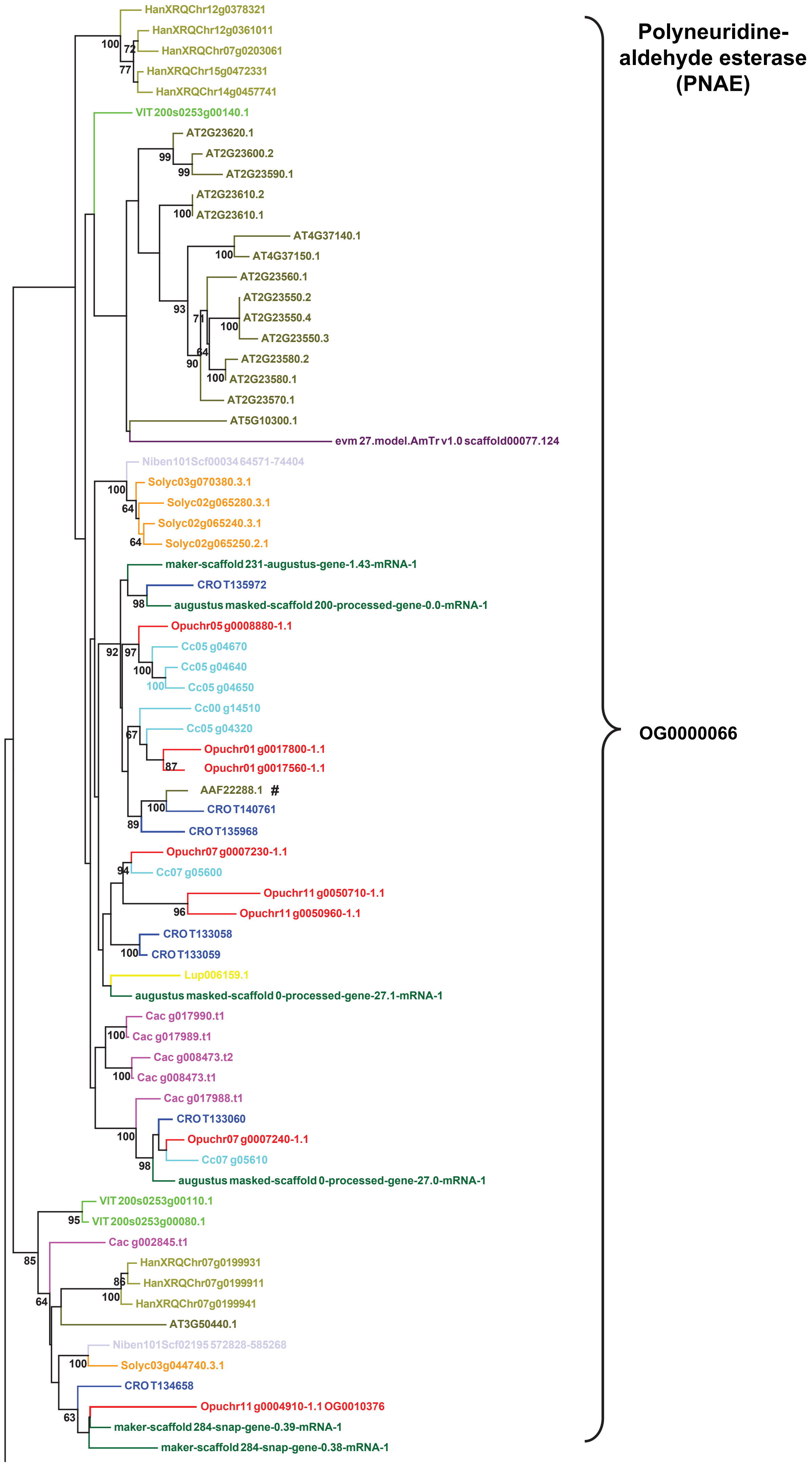

HanXRQChr12g0378321
HanXRQChr12g0361011
HanXRQChr07g0203061
HanXRQChr15g0472331
HanXRQChr14g0457741
VIT 200s0253g00140.1
AT2G23620.1
AT2G23600.2
AT2G23590.1
AT2G23610.2
AT2G23610.1
AT4G37140.1
AT4G37150.1
AT2G23560.1
AT2G23550.2
AT2G23550.4
AT2G23550.3
AT2G23580.2
AT2G23580.1
AT2G23570.1
AT5G10300.1
evm 27.model.AmTr v1.0 scaffold00077.124
Niben101Scf00034 64571-74404
Solyc03g070380.3.1
Solyc02g065280.3.1
Solyc02g065240.3.1
Solyc02g065250.2.1
maker-scaffold 231-augustus-gene-1.43-mRNA-1
CRO T135972
augustus masked-scaffold 200-processed-gene-0.0-mRNA-1
Opuchr05 g0008880-1.1
Cc05 g04670
Cc05 g04640
Cc05 g04650
Cc00 g14510
Cc05 g04320
Opuchr01 g0017800-1.1
Opuchr01 g0017560-1.1
AAF22288.1 #
CRO T140761
CRO T135968
Opuchr07 g0007230-1.1
Cc07 g05600
Opuchr11 g0050710-1.1
Opuchr11 g0050960-1.1
CRO T133058
CRO T133059
Lup006159.1
augustus masked-scaffold 0-processed-gene-27.1-mRNA-1
Cac g017990.t1
Cac g017989.t1
Cac g008473.t2
Cac g008473.t1
Cac g017988.t1
CRO T133060
Opuchr07 g0007240-1.1
Cc07 g05610
augustus masked-scaffold 0-processed-gene-27.0-mRNA-1
VIT 200s0253g00110.1
VIT 200s0253g00080.1
Cac g002845.t1
HanXRQChr07g0199931
HanXRQChr07g0199911
HanXRQChr07g0199941
AT3G50440.1
Niben101Scf02195 572828-585268
Solyc03g044740.3.1
CRO T134658
Opuchr11 g0004910-1.1 OG0010376
maker-scaffold 284-snap-gene-0.39-mRNA-1
maker-scaffold 284-snap-gene-0.38-mRNA-1
100
72
77
99
99
100
100
93
71
100
64
90
100
100
64
64
98
92
97
100
100
67
87
100
89
94
96
100
100
100
100
98
95
85
86
100
64
100
63
Polyneuridine-aldehyde esterase (PNAE)
OG0000066

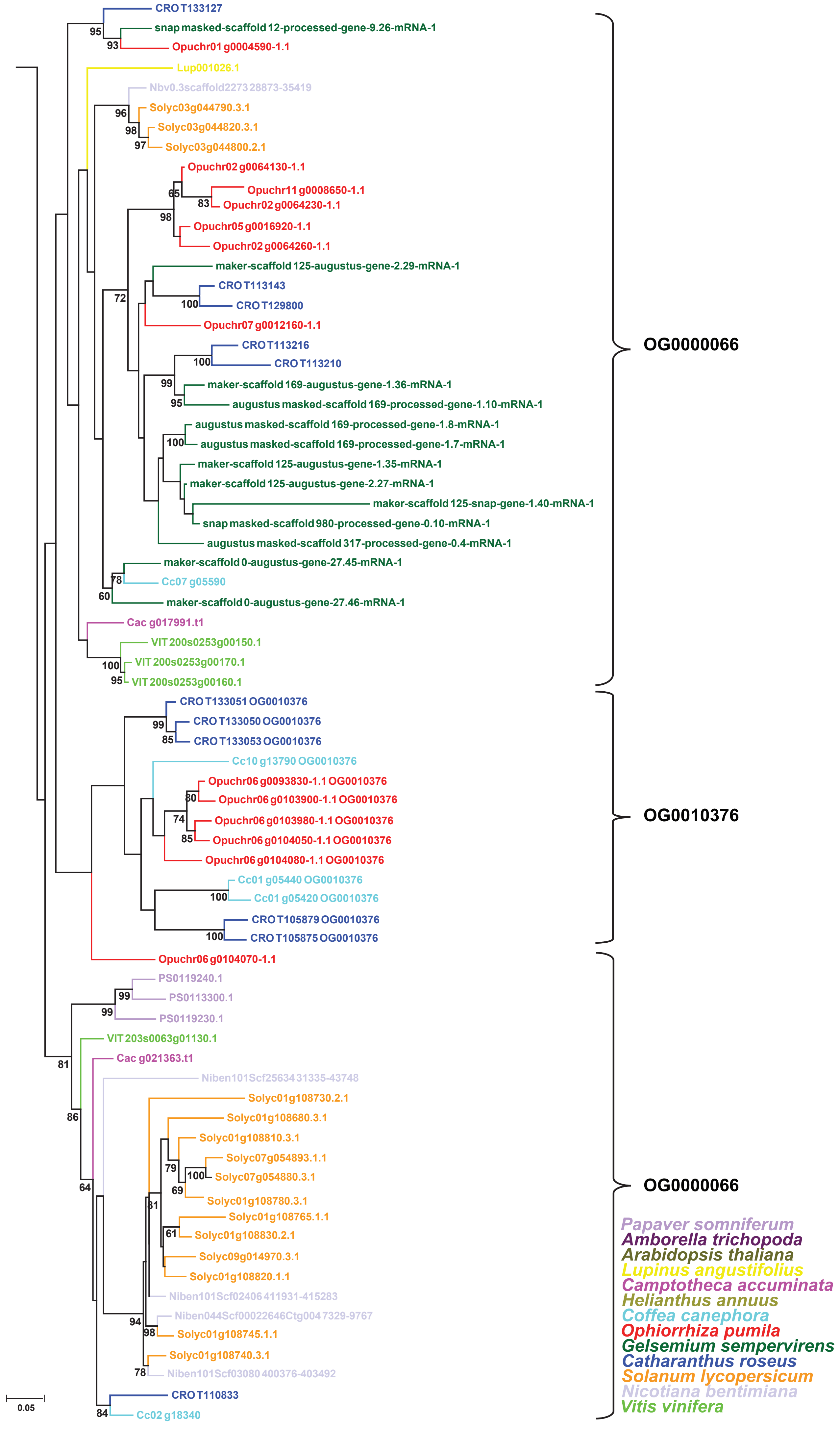
CROT133127
snap masked-scaffold 12-processed-gene-9.26-mRNA-1
Opuchr01 g0004590-1.1
Lup001026.1
Nbv0.3scaffold2273 28873-35419
Solyc03g044790.3.1
Solyc03g044820.3.1
Solyc03g044800.2.1
Opuchr02 g0064130-1.1
Opuchr11 g0008650-1.1
Opuchr02 g0064230-1.1
Opuchr05 g0016920-1.1
Opuchr02 g0064260-1.1
maker-scaffold 125-augustus-gene-2.29-mRNA-1
CROT113143
CROT129800
Opuchr07 g0012160-1.1
CROT113216
CROT113210
maker-scaffold 169-augustus-gene-1.36-mRNA-1
augustus masked-scaffold 169-processed-gene-1.10-mRNA-1
augustus masked-scaffold 169-processed-gene-1.8-mRNA-1
augustus masked-scaffold 169-processed-gene-1.7-mRNA-1
maker-scaffold 125-augustus-gene-1.35-mRNA-1
maker-scaffold 125-augustus-gene-2.27-mRNA-1
maker-scaffold 125-snap-gene-1.40-mRNA-1
snap masked-scaffold 980-processed-gene-0.10-mRNA-1
augustus masked-scaffold 317-processed-gene-0.4-mRNA-1
maker-scaffold 0-augustus-gene-27.45-mRNA-1
Cc07 g05590
maker-scaffold 0-augustus-gene-27.46-mRNA-1
Cac g017991.t1
VIT 200s0253g00150.1
VIT 200s0253g00170.1
VIT 200s0253g00160.1
OG0000066
CROT133051 OG0010376
CROT133050 OG0010376
CROT133053 OG0010376
Cc10 g13790 OG0010376
Opuchr06 g0093830-1.1 OG0010376
Opuchr06 g0103900-1.1 OG0010376
Opuchr06 g0103980-1.1 OG0010376
Opuchr06 g0104050-1.1 OG0010376
Opuchr06 g0104080-1.1 OG0010376
Cc01 g05440 OG0010376
Cc01 g05420 OG0010376
CROT105879 OG0010376
CROT105875 OG0010376
OG0010376
Opuchr06 g0104070-1.1
PS0119240.1
PS0113300.1
PS0119230.1
VIT 203s0063g01130.1
Cac g021363.t1
Niben101Scf25634 31335-43748
Solyc01g108730.2.1
Solyc01g108680.3.1
Solyc01g108810.3.1
Solyc07g054893.1.1
Solyc07g054880.3.1
Solyc01g108780.3.1
Solyc01g108765.1.1
Solyc01g108830.2.1
Solyc09g014970.3.1
Solyc01g108820.1.1
Niben101Scf02406 411931-415283
Niben044Scf00022646Ctg004 7329-9767
Solyc01g108745.1.1
Solyc01g108740.3.1
Niben101Scf03080 400376-403492
CROT110833
Cc02 g18340
OG0000066
0.05
Papaver somniferum
Amborella trichopoda
Arabidopsis thaliana
Lupinus angustifolius
Camptotheca accuminata
Helianthus annuus
Coffea canephora
Ophiorrhiza pumila
Gelsemium sempervirens
Catharanthus roseus
Solanum lycopersicum
Nicotiana bentimiana
Vitis vinifera

Supplementary Fig. 38. Phylogenetic analysis of polyneuridine-aldehyde esterase (PNAE) assigned to the orthogene families, OG0000066 and OG0010376. Gene family classification was performed based on 13 plant genomes, and the genes are colored based on the associated plant species. Functionally characterized genes are represented by "#" with the GenBank accession IDs. Strongly supported nodes with bootstrap value above 60% are shown.

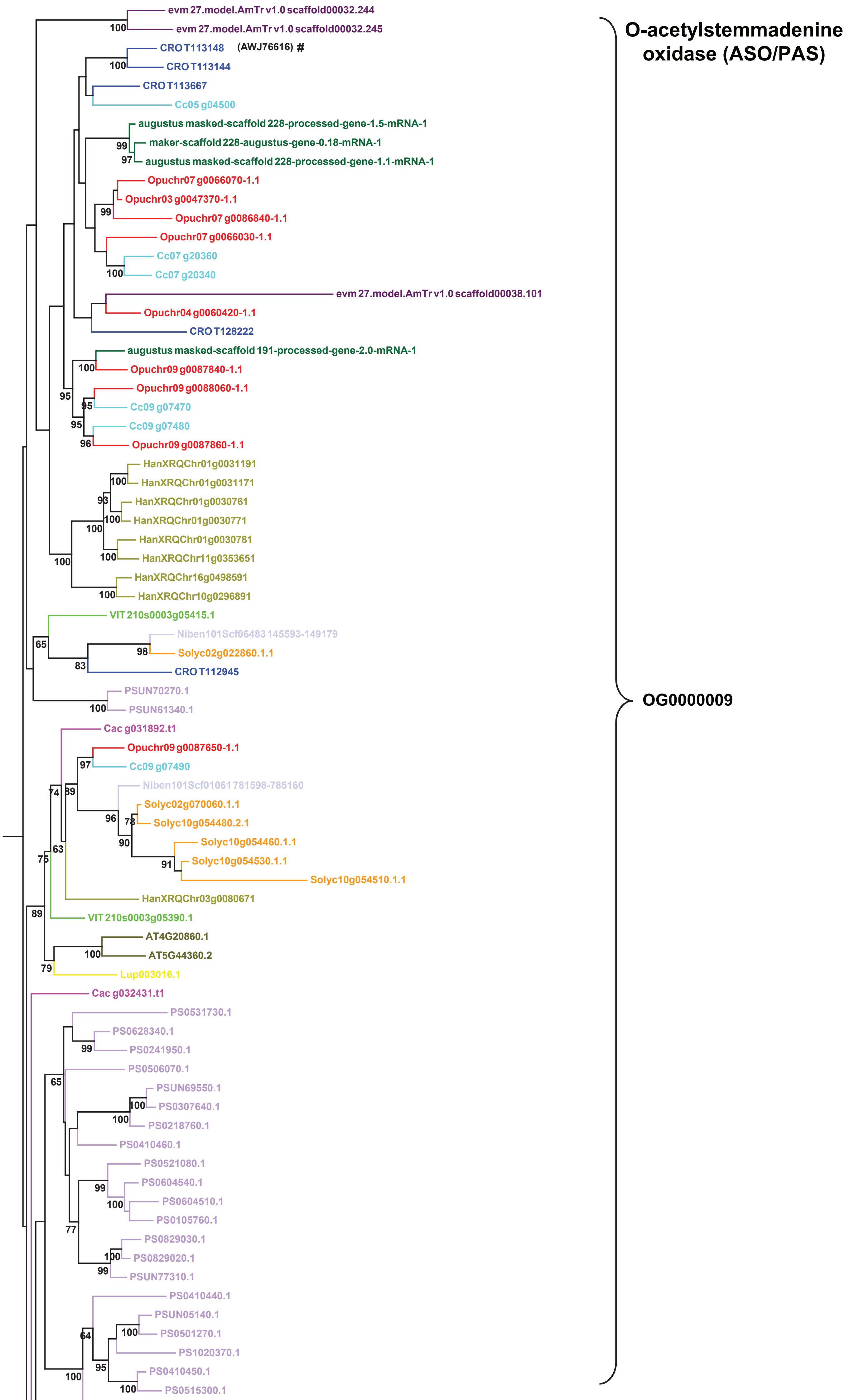

O-acetylstemmadenine oxidase (ASO/PAS)
OG0000009
evm 27.model.AmTr v1.0 scaffold00032.244
evm 27.model.AmTr v1.0 scaffold00032.245
CRO T113148
(AWJ76616) #
CRO T113144
CRO T113667
Cc05 g04500
augustus masked-scaffold 228-processed-gene-1.5-mRNA-1
maker-scaffold 228-augustus-gene-0.18-mRNA-1
augustus masked-scaffold 228-processed-gene-1.1-mRNA-1
Opuchr07 g0066070-1.1
Opuchr03 g0047370-1.1
Opuchr07 g0086840-1.1
Opuchr07 g0066030-1.1
Cc07 g20360
Cc07 g20340
evm 27.model.AmTr v1.0 scaffold00038.101
Opuchr04 g0060420-1.1
CRO T128222
augustus masked-scaffold 191-processed-gene-2.0-mRNA-1
Opuchr09 g0087840-1.1
Opuchr09 g0088060-1.1
Cc09 g07470
Cc09 g07480
Opuchr09 g0087860-1.1
HanXRQChr01g0031191
HanXRQChr01g0031171
HanXRQChr01g0030761
HanXRQChr01g0030771
HanXRQChr01g0030781
HanXRQChr11g0353651
HanXRQChr16g0498591
HanXRQChr10g0296891
VIT 210s0003g05415.1
Niben101Scf06483 145593-149179
Solyc02g022860.1.1
CRO T112945
PSUN70270.1
PSUN61340.1
Cac g031892.t1
Opuchr09 g0087650-1.1
Cc09 g07490
Niben101Scf01061 781598-785160
Solyc02g070060.1.1
Solyc10g054480.2.1
Solyc10g054460.1.1
Solyc10g054530.1.1
Solyc10g054510.1.1
HanXRQChr03g0080671
VIT 210s0003g05390.1
AT4G20860.1
AT5G44360.2
Lup003016.1
Cac g032431.t1
PS0531730.1
PS0628340.1
PS0241950.1
PS0506070.1
PSUN69550.1
PS0307640.1
PS0218760.1
PS0410460.1
PS0521080.1
PS0604540.1
PS0604510.1
PS0105760.1
PS0829030.1
PS0829020.1
PSUN77310.1
PS0410440.1
PSUN05140.1
PS0501270.1
PS1020370.1
PS0410450.1
PS0515300.1

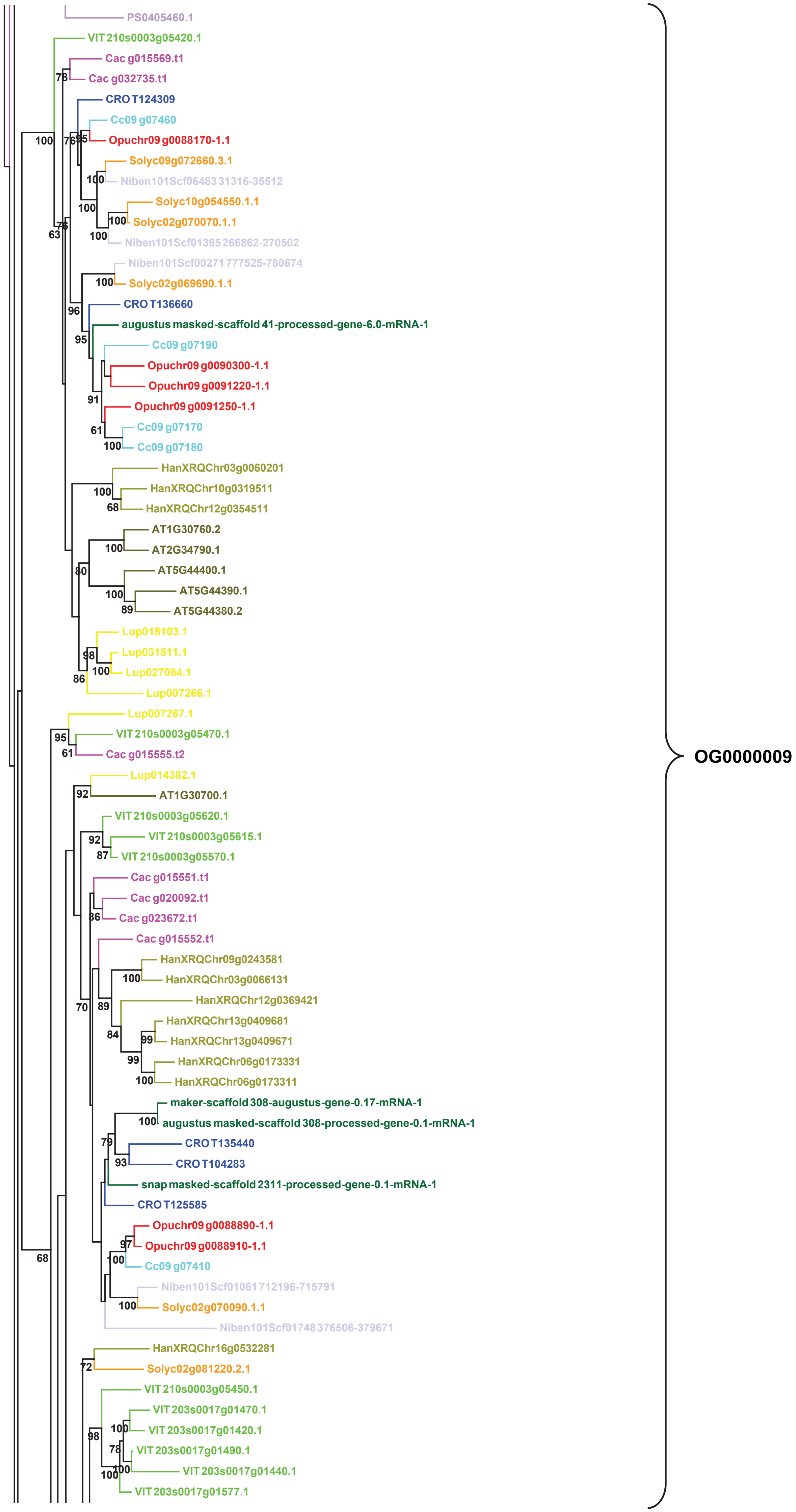
PS0405460.1
VIT 210s0003g05420.1
Cac g015569.t1
Cac g032735.t1
CRO T124309
Cc09 g07460
Opuchr09 g0088170-1.1
Solyc09g072660.3.1
Niben101Scf06483 31316-35512
Solyc10g054550.1.1
Solyc02g070070.1.1
Niben101Scf01395 266862-270502
Niben101Scf00271 777525-780674
Solyc02g069690.1.1
CRO T136660
augustus masked-scaffold 41-processed-gene-6.0-mRNA-1
Cc09 g07190
Opuchr09 g0090300-1.1
Opuchr09 g0091220-1.1
Opuchr09 g0091250-1.1
Cc09 g07170
Cc09 g07180
HanXRQChr03g0060201
HanXRQChr10g0319511
HanXRQChr12g0354511
AT1G30760.2
AT2G34790.1
AT5G44400.1
AT5G44390.1
AT5G44380.2
Lup018103.1
Lup031811.1
Lup027084.1
Lup007266.1
Lup007267.1
VIT 210s0003g05470.1
Cac g015555.t2
Lup014382.1
AT1G30700.1
VIT 210s0003g05620.1
VIT 210s0003g05615.1
VIT 210s0003g05570.1
Cac g015551.t1
Cac g020092.t1
Cac g023672.t1
Cac g015552.t1
HanXRQChr09g0243581
HanXRQChr03g0066131
HanXRQChr12g0369421
HanXRQChr13g0409681
HanXRQChr13g0409671
HanXRQChr06g0173331
HanXRQChr06g0173311
maker-scaffold 308-augustus-gene-0.17-mRNA-1
augustus masked-scaffold 308-processed-gene-0.1-mRNA-1
CRO T135440
CRO T104283
snap masked-scaffold 2311-processed-gene-0.1-mRNA-1
CRO T125585
Opuchr09 g0088890-1.1
Opuchr09 g0088910-1.1
Cc09 g07410
Niben101Scf01061 712196-715791
Solyc02g070090.1.1
Niben101Scf01748 376506-379671
HanXRQChr16g0532281
Solyc02g081220.2.1
VIT 210s0003g05450.1
VIT 203s0017g01470.1
VIT 203s0017g01420.1
VIT 203s0017g01490.1
VIT 203s0017g01440.1
VIT 203s0017g01577.1
OG0000009

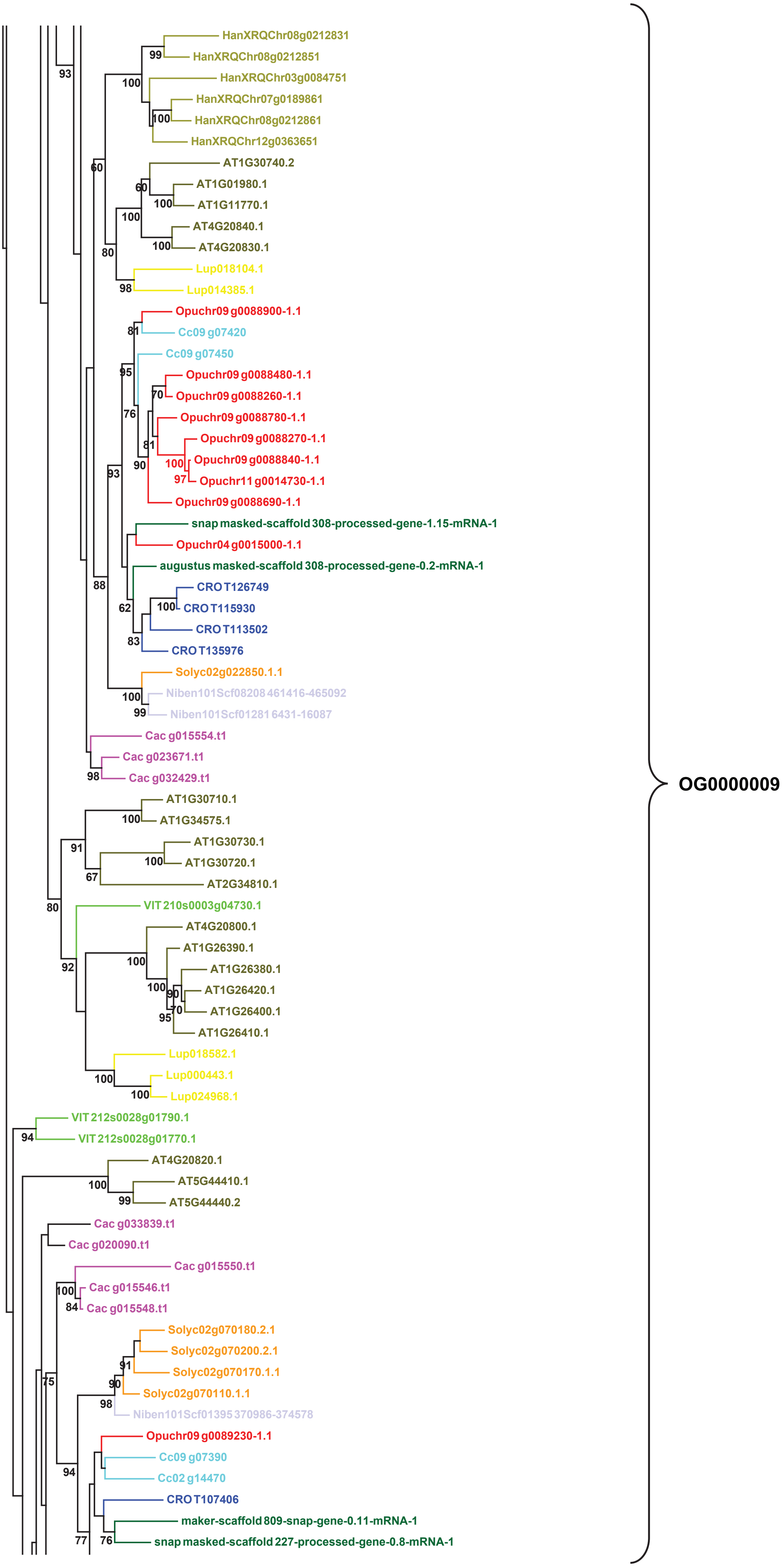

HanXRQChr08g0212831
HanXRQChr08g0212851
HanXRQChr03g0084751
HanXRQChr07g0189861
HanXRQChr08g0212861
HanXRQChr12g0363651
AT1G30740.2
AT1G01980.1
AT1G11770.1
AT4G20840.1
AT4G20830.1
Lup018104.1
Lup014385.1
Opuchr09 g0088900-1.1
Cc09 g07420
Cc09 g07450
Opuchr09 g0088480-1.1
Opuchr09 g0088260-1.1
Opuchr09 g0088780-1.1
Opuchr09 g0088270-1.1
Opuchr09 g0088840-1.1
Opuchr11 g0014730-1.1
Opuchr09 g0088690-1.1
snap masked-scaffold 308-processed-gene-1.15-mRNA-1
Opuchr04 g0015000-1.1
augustus masked-scaffold 308-processed-gene-0.2-mRNA-1
CRO T126749
CRO T115930
CRO T113502
CRO T135976
Solyc02g022850.1.1
Niben101Scf08208 461416-465092
Niben101Scf01281 6431-16087
Cac g015554.t1
Cac g023671.t1
Cac g032429.t1
AT1G30710.1
AT1G34575.1
AT1G30730.1
AT1G30720.1
AT2G34810.1
VIT 210s0003g04730.1
AT4G20800.1
AT1G26390.1
AT1G26380.1
AT1G26420.1
AT1G26400.1
AT1G26410.1
Lup018582.1
Lup000443.1
Lup024968.1
VIT 212s0028g01790.1
VIT 212s0028g01770.1
AT4G20820.1
AT5G44410.1
AT5G44440.2
Cac g033839.t1
Cac g020090.t1
Cac g015550.t1
Cac g015546.t1
Cac g015548.t1
Solyc02g070180.2.1
Solyc02g070200.2.1
Solyc02g070170.1.1
Solyc02g070110.1.1
Niben101Scf01395 370986-374578
Opuchr09 g0089230-1.1
Cc09 g07390
Cc02 g14470
CRO T107406
maker-scaffold 809-snap-gene-0.11-mRNA-1
snap masked-scaffold 227-processed-gene-0.8-mRNA-1
OG0000009

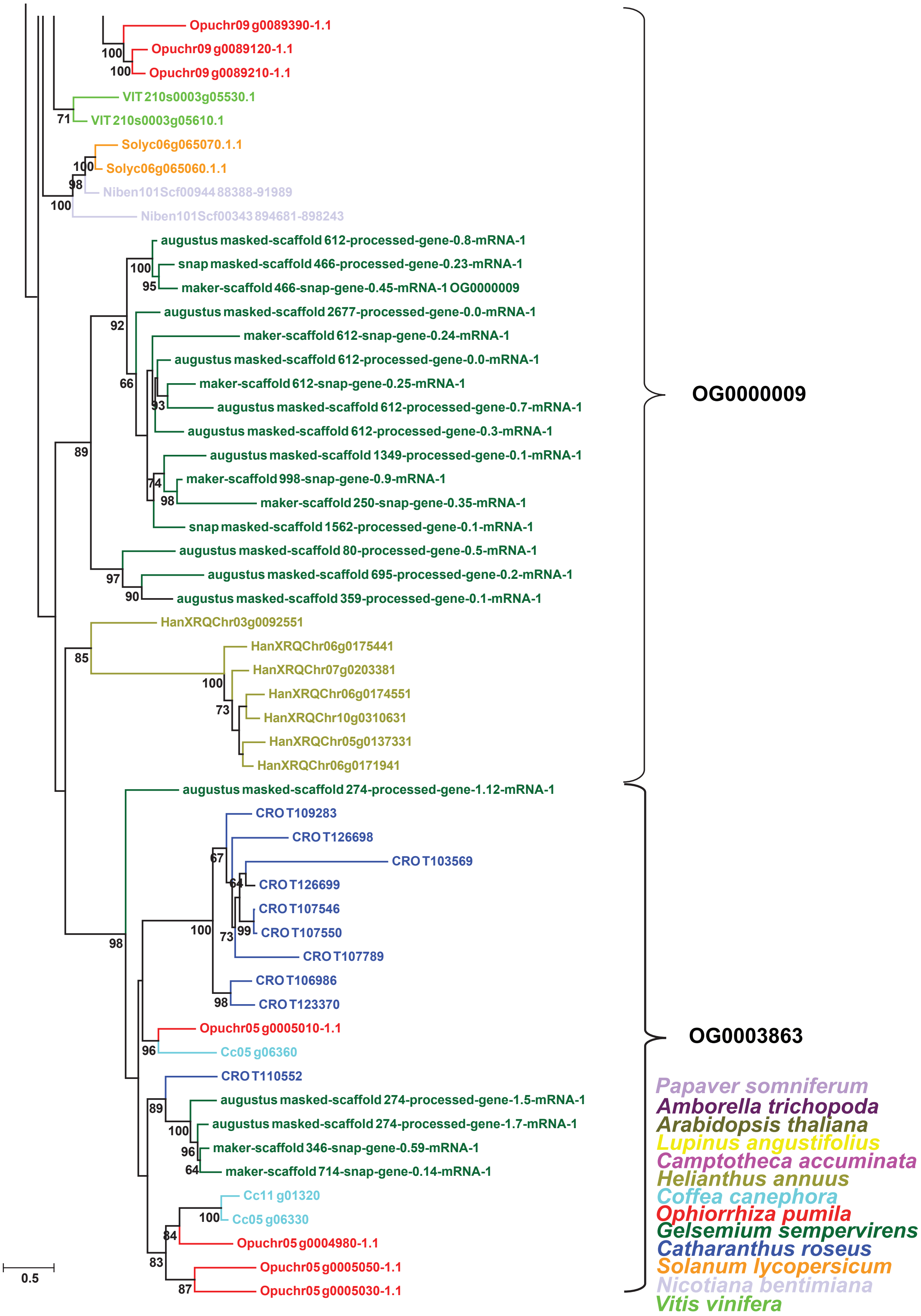

Supplementary Fig. 39. Phylogenetic analysis of O-acetylstemmadenine oxidase (ASO/PAS) assigned to the orthogene families, OG0000009 and OG0003863. Gene family classification was performed based on 13 plant genomes, and the genes are colored based on the associated plant species. Functionally characterized genes are represented by "#" with the GenBank accession IDs. Strongly supported nodes with bootstrap value above 60% are shown.

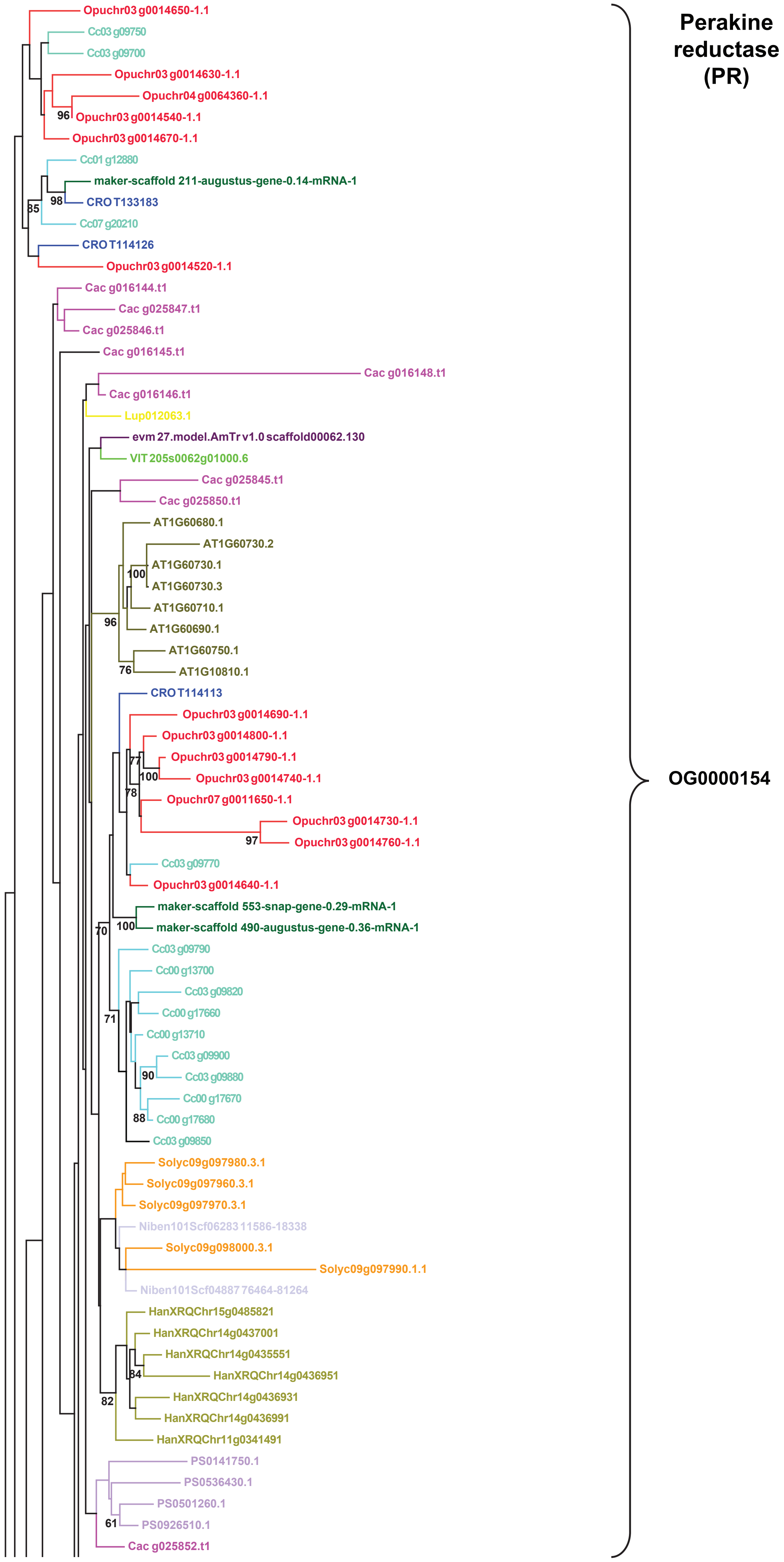

Perakine reductase (PR)
OG0000154
Opuchr03g0014650-1.1
Cc03g09750
Cc03g09700
Opuchr03g0014630-1.1
Opuchr04g0064360-1.1
96
Opuchr03g0014540-1.1
Opuchr03g0014670-1.1
Cc01g12880
maker-scaffold 211-augustus-gene-0.14-mRNA-1
98
CROT133183
85
Cc07g20210
CROT114126
Opuchr03g0014520-1.1
Cacg016144.t1
Cacg025847.t1
Cacg025846.t1
Cacg016145.t1
Cacg016148.t1
Cacg016146.t1
Lup012063.1
evm27.model.AmTrv1.0scaffold00062.130
VIT205s0062g01000.6
Cacg025845.t1
Cacg025850.t1
AT1G60680.1
AT1G60730.2
AT1G60730.1
100
AT1G60730.3
AT1G60710.1
96
AT1G60690.1
AT1G60750.1
76
AT1G10810.1
CROT114113
Opuchr03g0014690-1.1
Opuchr03g0014800-1.1
77
Opuchr03g0014790-1.1
100
Opuchr03g0014740-1.1
78
Opuchr07g0011650-1.1
Opuchr03g0014730-1.1
97
Opuchr03g0014760-1.1
Cc03g09770
Opuchr03g0014640-1.1
maker-scaffold 553-snap-gene-0.29-mRNA-1
70
100
maker-scaffold 490-augustus-gene-0.36-mRNA-1
Cc03g09790
Cc00g13700
Cc03g09820
71
Cc00g17660
Cc00g13710
Cc03g09900
90
Cc03g09880
Cc00g17670
88
Cc00g17680
Cc03g09850
Solyc09g097980.3.1
Solyc09g097960.3.1
Solyc09g097970.3.1
Niben101Scf06283 11586-18338
Solyc09g098000.3.1
Solyc09g097990.1.1
Niben101Scf04887 76464-81264
HanXRQChr15g0485821
HanXRQChr14g0437001
HanXRQChr14g0435551
84
HanXRQChr14g0436951
HanXRQChr14g0436931
82
HanXRQChr14g0436991
HanXRQChr11g0341491
PS0141750.1
PS0536430.1
PS0501260.1
61
PS0926510.1
Cacg025852.t1

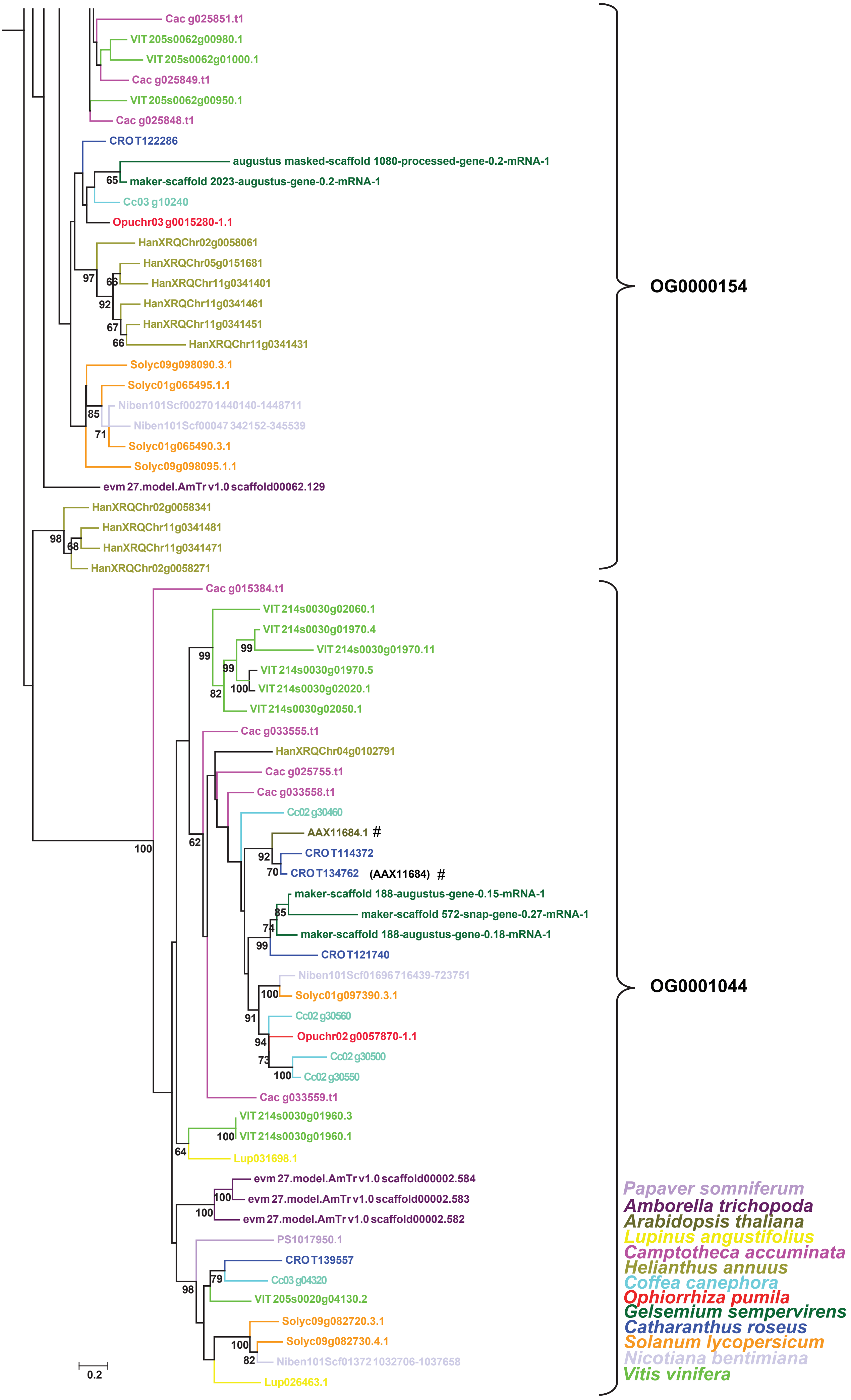

Cac g025851.t1
VIT 205s0062g00980.1
VIT 205s0062g01000.1
Cac g025849.t1
VIT 205s0062g00950.1
Cac g025848.t1
CRO T122286
augustus masked-scaffold 1080-processed-gene-0.2-mRNA-1
65
maker-scaffold 2023-augustus-gene-0.2-mRNA-1
Cc03 g10240
Opuchr03 g0015280-1.1
HanXRQChr02g0058061
HanXRQChr05g0151681
97
66
HanXRQChr11g0341401
92
HanXRQChr11g0341461
67
HanXRQChr11g0341451
66
HanXRQChr11g0341431
Solyc09g098090.3.1
Solyc01g065495.1.1
85
Niben101Scf00270 1440140-1448711
Niben101Scf00047 342152-345539
71
Solyc01g065490.3.1
Solyc09g098095.1.1
evm 27.model.AmTr v1.0 scaffold00062.129
HanXRQChr02g0058341
HanXRQChr11g0341481
98
68
HanXRQChr11g0341471
HanXRQChr02g0058271
OG0000154
Cac g015384.t1
VIT 214s0030g02060.1
VIT 214s0030g01970.4
99
VIT 214s0030g01970.11
99
99
VIT 214s0030g01970.5
100
VIT 214s0030g02020.1
82
VIT 214s0030g02050.1
Cac g033555.t1
HanXRQChr04g0102791
Cac g025755.t1
Cac g033558.t1
Cc02 g30460
AAX11684.1 #
100
62
92
CRO T114372
70
CRO T134762 (AAX11684) #
maker-scaffold 188-augustus-gene-0.15-mRNA-1
85
maker-scaffold 572-snap-gene-0.27-mRNA-1
74
maker-scaffold 188-augustus-gene-0.18-mRNA-1
99
CRO T121740
Niben101Scf01696 716439-723751
100
Solyc01g097390.3.1
91
Cc02 g30560
94
Opuchr02 g0057870-1.1
73
Cc02 g30500
100
Cc02 g30550
Cac g033559.t1
VIT 214s0030g01960.3
100
VIT 214s0030g01960.1
64
Lup031698.1
evm 27.model.AmTr v1.0 scaffold00002.584
100
evm 27.model.AmTr v1.0 scaffold00002.583
100
evm 27.model.AmTr v1.0 scaffold00002.582
PS1017950.1
CRO T139557
79
Cc03 g04320
98
VIT 205s0020g04130.2
Solyc09g082720.3.1
100
Solyc09g082730.4.1
82
Niben101Scf01372 1032706-1037658
Lup026463.1
0.2
OG0001044
Papaver somniferum
Amborella trichopoda
Arabidopsis thaliana
Lupinus angustifolius
Camptotheca accuminata
Helianthus annuus
Coffea canephora
Ophiorrhiza pumila
Gelsemium sempervirens
Catharanthus roseus
Solanum lycopersicum
Nicotiana bentimiana
Vitis vinifera

Supplementary Fig. 40. Phylogenetic analysis of polyneuridine-aldehyde esterase (PR) assigned to the orthogene families, OG0000154, and OG0001044. Gene family classification was performed based on 13 plant genomes, and the genes are colored based on the associated plant species. Functionally characterized genes are represented by "#" with the GenBank accession IDs. Strongly supported nodes with bootstrap value above 60% are shown.

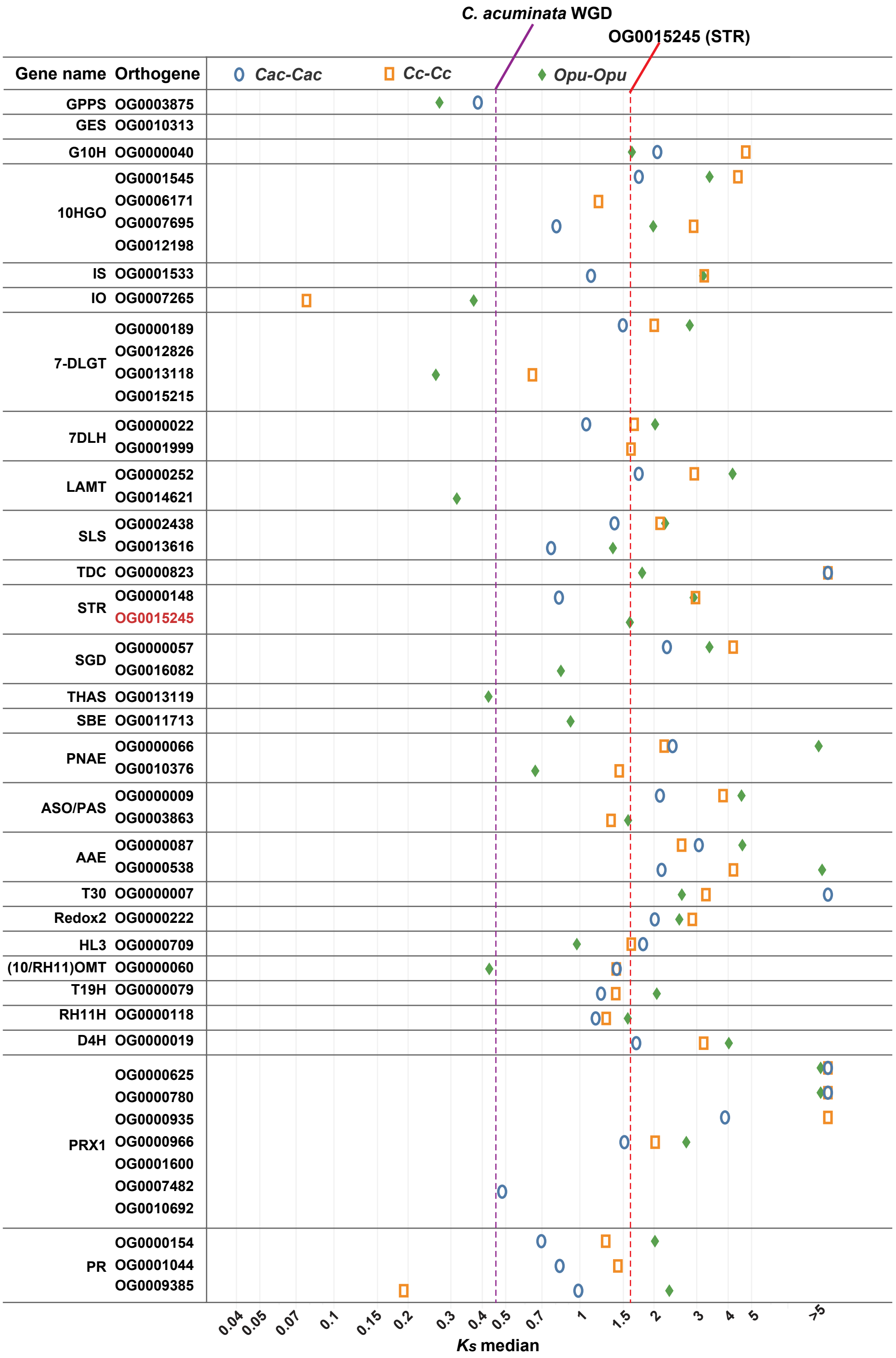

Supplementary Fig. 41. Evolution of secoiridoid and MIA biosynthesis associated orthogene families in camptothecin producing plants and coffee genome. Median for synonymous substitutions per synonymous sites (*Ks*) were calculated using genes assigned to a given orthogene family for a plant species as described in method section. Small *Ks*-median values for several key enzymes associated with MIA biosynthesis in *O. pumila* and *C. acuminata*, while high *Ks*-median values for genes from coffee genome suggests faster and active evolution of the functional genes in the camptothecin producing plants post established STR enzymes. Results suggested evolution of STR as key event that preceded with evolution of genes associated with MIA biosynthesis, a factor that was missing for coffee genome. Source data are provided as a Source Data file.

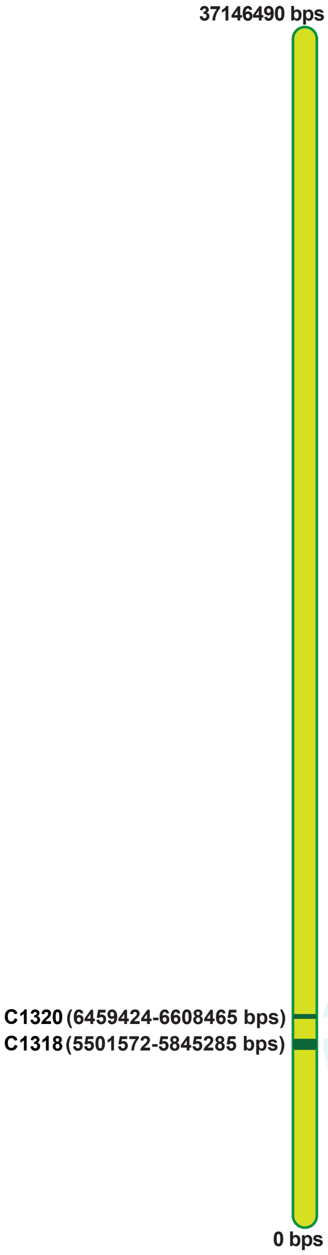

Gene id	Orthogroup	① O.p	① C.a	① C.r	① G.s	② C.a	② C.r	② G.s	Description
Opuchr01_g0011180-1.1	OG0013007	6	0	0	0	×	×	×	strictosidine glucosidase
Opuchr01_g0011190-1.1	OG0013509	1	0	0	0	×	×	•	Protein kinase domain
Opuchr01_g0011200-1.1	OG0013270	2	0	0	2	×	×	×	putative strictosidine beta-D-glucosidase
Opuchr01_g0011210-1.1	OG0013007	6	0	0	0	×	×	×	strictosidine glucosidase
Opuchr01_g0011270-1.1	OG0000823	4	4	2	3	×	×	×	tryptophan decarboxylase #
Opuchr01_g0011280-1.1	OG0013007	6	0	0	0	×	×	×	strictosidine glucosidase
Opuchr01_g0011300-1.1	OG0003025	7	0	1	10	×	×	×	putative strictosidine beta-D-glucosidase
Opuchr01_g0011310-1.1						×	×	×	strictosidine glucosidase
Opuchr01_g0011320-1.1	OG0003025	7	0	1	10	×	×	×	putative strictosidine beta-D-glucosidase
Opuchr01_g0011330-1.1	OG0013007	6	0	0	0	×	×	×	strictosidine glucosidase
Opuchr01_g0011340-1.1	OG0015956	1	0	0	0	×	×	×	UDP-glycosyltransferase 92A1-like
Opuchr01_g0011350-1.1						×	×	×	uncharacterized protein
Opuchr01_g0011360-1.1						×	×	×	unnamed protein product
Opuchr01_g0011370-1.1						×	×	•	strictosidine glucosidase
Opuchr01_g0011440-1.1	OG0013007	6	0	0	0	×	×	×	strictosidine glucosidase
Opuchr01_g0011450-1.1	OG0003025	7	0	1	10	×	×	×	putative strictosidine beta-D-glucosidase

Gene id	Orthogroup	① O.p	① C.a	① C.r	① G.s	② C.a	② C.r	② G.s	Description
Opuchr01_g0009890-1.1	OG0001702	8	3	2	1	×	•	×	Deacetoxyvindoline 4-hydroxylase
Opuchr01_g0009930-1.1	OG0001702	8	3	2	1	×	×	×	Deacetoxyvindoline 4-hydroxylase
Opuchr01_g0009950-1.1						×	×	×	Deacetoxyvindoline 4-hydroxylase
Opuchr01_g0009970-1.1						×	•	•	taxadien-5-alpha-ol O-acetyltransferase-like
Opuchr01_g0010030-1.1	OG0001702	8	3	2	1	×	×	×	Deacetoxyvindoline 4-hydroxylase
Opuchr01_g0010040-1.1						×	×	×	Phosphatidate cytidylyltransferase
Opuchr01_g0010050-1.1	OG0010906	1	1	1	1	×	×	×	vinorine synthase
Opuchr01_g0010060-1.1	OG0001272	3	2	2	3	×	•	•	Deacetoxyvindoline 4-hydroxylase
Opuchr01_g0010100-1.1	OG0003923	2	1	2	1	×	×	×	Alanine-glyoxylate aminotransferase AGT2
Opuchr01_g0010140-1.1	OG0000057	24	18	7	17	×	×	×	strictosidine beta-D-glucosidase
Opuchr01_g0010150-1.1						×	×	×	uncharacterized protein LOC113729443
Opuchr01_g0010180-1.1						×	•	×	APO domain
Opuchr01_g0010190-1.1	OG0005658	1	1	1	2	×	•	×	7-deoxyloganetin glucosyltransferase
Opuchr01_g0010200-1.1	OG0000046	12	8	1	6	×	×	×	putative disease resistance protein RGA3
Opuchr01_g0010210-1.1	OG0000057	24	18	7	17	×	×	×	strictosidine glucosidase
Opuchr01_g0010220-1.1	OG0013500	2	0	1	0	×	•	×	strictosidine glucosidase
Opuchr01_g0010230-1.1	OG0000057	24	18	7	17	×	×	×	strictosidine beta-D-glucosidase
Opuchr01_g0010240-1.1	OG0001272	3	2	2	3	×	×	×	Deacetoxyvindoline 4-hydroxylase
Opuchr01_g0010250-1.1	OG0000967	4	3	3	2	×	•	×	vesicle transport protein GOT1-like
Opuchr01_g0010260-1.1	OG0005244	1	1	1	1	×	×	•	SET domain
Opuchr01_g0010280-1.1	OG0009219	1	1	1	1	×	•	•	Argininosuccinate synthase
Opuchr01_g0010300-1.1	OG0008238	1	1	1	1	•	×	•	formate--tetrahydrofolate ligase
Opuchr01_g0010310-1.1	OG0007482	1	2	1	1	•	•	•	peroxidase 1
Opuchr01_g0010320-1.1	OG0007999	1	1	1	1	×	×	×	transcription factor like protein
Opuchr01_g0010330-1.1	OG0011040	1	1	1	1	•	•	•	probable plastid-lipid-associated protein 12, chloroplastic isoform X2
Opuchr01_g0010340-1.1	OG0013060	1	1	1	1	×	×	×	uncharacterized protein LOC113760095
Opuchr01_g0010350-1.1	OG0008594	1	1	1	1	×	•	•	probable ribose-5-phosphate isomerase 3, chloroplastic

① **Number of genes assigned to an orthogroup from a specific species**

② **Presence or absence of genes from a syteny relationships with *O. pumila*** { • Present × Absent }

Supplementary Fig. 42. Monoterpene indole alkaloid (MIA) gene-clusters identified in chromosome 1 of the *Ophiorrhiza* genome assembly. The position of the gene-cluster is scaled based on chromosome size and its physical position on the chromosome. The gene description, colored as orange, represents genes with functions associated with MIA biosynthesis. O.p: *Ophiorrhiza pumila;* C.a: *Camptotheca acuminata;* C.r: *Catharanthus roseus;* G.s: *Gelsemium sempervirens.*

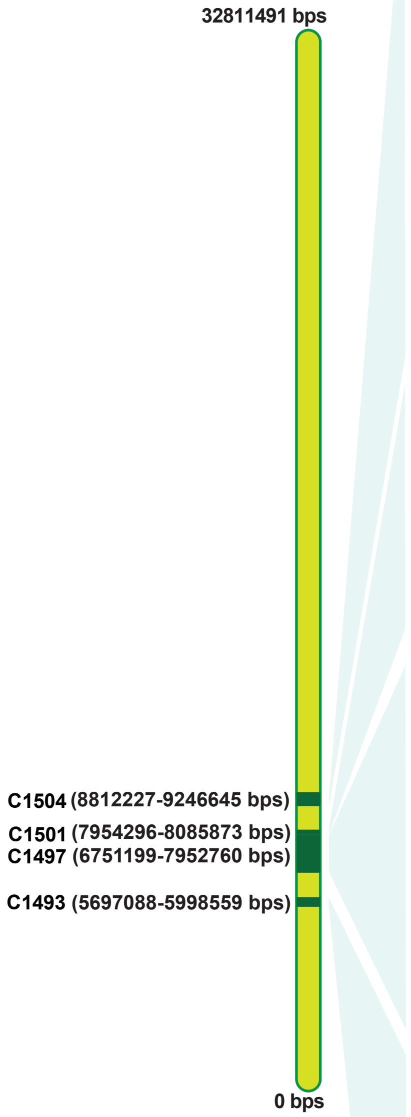

Gene id	Orthogroup	① O.p	① C.a	① C.r	① G.s	② C.a	② C.r	② G.s	Description
Opuchr04_g0014720-1.1	OG0005946	1	1	1	1	×	•	•	Indole-3-glycerol phosphate synthase, conserved site
Opuchr04_g0014730-1.1						×	×	×	uncharacterized protein
Opuchr04_g0014760-1.1	OG0013021	1	1	0	1	•	×	•	Glyoxal oxidase, N-terminal
Opuchr04_g0014790-1.1	OG0012283	1	0	0	0	×	×	×	endoglucanase 11-like
Opuchr04_g0014800-1.1	OG0015239	4	0	0	0	×	×	×	uncharacterized protein LOC113761003 isoform X2
Opuchr04_g0014810-1.1						×	×	×	UDP-3-O-[3-hydroxymyristoyl] glucosamine N-acyltransferase
Opuchr04_g0014840-1.1	OG0005182	15	1	1	0	×	×	×	Endonuclease/exonuclease/phosphatase superfamily
Opuchr04_g0014850-1.1	OG0005775	12	0	0	0	×	×	×	uncharacterized protein
Opuchr04_g0014880-1.1	OG0000232	3	6	4	5	•	•	•	E3 ubiquitin-protein ligase RGLG2-like
Opuchr04_g0014900-1.1	OG0005213	1	1	1	1	×	×	×	NADH dehydrogenase
Opuchr04_g0014920-1.1	OG0009626	1	1	1	1	×	•	•	Nucleoside phosphorylase/phosphoribosyltransferase catalytic domain superfamily
Opuchr04_g0014930-1.1	OG0006840	1	2	1	1	×	•	•	Aminoacyl-tRNA synthetase, class I, conserved site
Opuchr04_g0014940-1.1	OG0000079	13	4	11	7	×	×	×	tabersonine/lochnericine 19-hydroxylase
Opuchr04_g0014950-1.1						×	×	×	cytochrome P450 71A3-like
Opuchr04_g0014960-1.1	OG0010699	1	1	1	1	•	•	•	rhodanese-like domain-containing protein 11, chloroplastic
Opuchr04_g0014970-1.1	OG0000007	40	13	26	24	•	×	•	rankinidine/humantenine-11-hydroxylase 3
Opuchr04_g0014990-1.1	OG0000079	13	4	11	7	×	×	×	Tabersonine 19-hydroxylase
Opuchr04_g0015000-1.1	OG0000009	28	17	15	28	×	×	×	O-acetylstemmadenine oxidase
Opuchr04_g0015020-1.1	OG0000027	72	6	15	7	×	×	×	uncharacterized protein LOC113737449
Opuchr04_g0015120-1.1						×	×	×	hypothetical protein L484_006967
Opuchr04_g0015160-1.1	OG0000079	13	4	11	7	×	•	×	tabersonine/lochnericine 19-hydroxylase
Opuchr04_g0015170-1.1	OG0000079	13	4	11	7	•	•	×	tabersonine/lochnericine 19-hydroxylase
Opuchr04_g0015190-1.1	OG0009082	2	1	1	1	×	×	×	multiprotein-bridging factor 1a
Opuchr04_g0015220-1.1	OG0000079	13	4	11	7	•	×	•	Tabersonine 19-hydroxylase
Opuchr04_g0015230-1.1	OG0000079	13	4	11	7	×	×	×	Tabersonine 19-hydroxylase
Opuchr04_g0015240-1.1						×	×	×	Ulp1 protease family, C-terminal catalytic domain
Opuchr04_g0015280-1.1	OG0000079	13	4	11	7	×	•	•	tabersonine/lochnericine 19-hydroxylase
Opuchr04_g0015290-1.1	OG0000079	13	4	11	7	•	×	×	tabersonine/lochnericine 19-hydroxylase
Opuchr04_g0015320-1.1	OG0001242	3	3	1	3	×	•	•	Protein kinase domain
Opuchr04_g0015330-1.1	OG0006608	2	2	2	1	•	•	×	protein LYK5-like
Opuchr04_g0015350-1.1	OG0010268	1	2	1	1	•	•	•	quinolinate synthase chloroplastic
Opuchr04_g0015360-1.1	OG0002105	2	2	5	2	•	•	•	high-affinity nitrate transporter 3.1-like
Opuchr04_g0015370-1.1	OG0002105	2	2	5	2	×	×	×	high-affinity nitrate transporter 3.1-like
Opuchr04_g0015380-1.1						×	×	×	uncharacterized protein LOC113755505
Opuchr04_g0015390-1.1	OG0012335	1	1	1	1	•	•	•	DHH phosphoesterase superfamily
Opuchr04_g0013420-1.1	OG0000082	13	12	15	20	•	×	×	stemmadenine O-acetyltransferase
Opuchr04_g0013430-1.1	OG0000082	13	12	15	20	×	×	•	stemmadenine O-acetyltransferase
Opuchr04_g0013440-1.1	OG0000082	13	12	15	20	×	×	×	stemmadenine O-acetyltransferase
Opuchr04_g0013450-1.1	OG0003283	8	0	5	0	×	×	×	Zinc finger, BED-type
Opuchr04_g0013480-1.1	OG0000342	4	4	3	3	×	×	×	vinorine synthase
Opuchr04_g0013530-1.1						×	×	×	Cytochrome P450, E-class, group I
Opuchr04_g0013540-1.1						×	×	×	hypothetical protein MIMGU_mgv1a0268911mg, partial
Opuchr04_g0013560-1.1	OG0000291	7	2	13	6	×	×	×	tabersonine-19-hydroxy-O-acetyltransferase
Opuchr04_g0013580-1.1	OG0000099	21	0	14	4	×	×	×	NAD(P)H-quinone oxidoreductase subunit 2 A, chloroplastic
Opuchr04_g0013590-1.1						×	×	×	PREDICTED: NAD(P)H-quinone oxidoreductase subunit 2 B, chloroplastic, partial
Opuchr04_g0013620-1.1	OG0000082	13	12	15	20	•	×	×	stemmadenine O-acetyltransferase
Opuchr04_g0013630-1.1	OG0001432	13	2	2	1	×	×	×	hypothetical protein CUMW_134320
Opuchr04_g0013640-1.1	OG0000082	13	12	15	20	•	•	•	stemmadenine O-acetyltransferase
Opuchr04_g0013670-1.1	OG0009879	1	1	1	1	•	×	•	uncharacterized protein LOC113738824
Opuchr04_g0013680-1.1	OG0006463	1	2	1	1	•	×	•	nucleoside diphosphate kinase 2, chloroplastic
Opuchr04_g0013690-1.1	OG0005755	2	2	1	2	•	•	•	DELLA protein RGL1-like
Opuchr04_g0013700-1.1	OG0005981	1	1	1	1	•	•	•	Protein kinase domain
Opuchr04_g0011750-1.1	OG0007265	2	1	2	3	×	×	×	7-deoxyloganetic acid synthase
Opuchr04_g0011760-1.1	OG0010940	1	0	1	0	×	×	×	unnamed protein product
Opuchr04_g0011770-1.1	OG0000905	2	3	2	3	•	×	•	NAC domain
Opuchr04_g0011800-1.1	OG0002886	1	3	2	1	•	•	•	Homeobox domain
Opuchr04_g0011820-1.1	OG0003862	17	0	2	0	×	×	×	putative F-box/LRR-repeat protein At3g28410
Opuchr04_g0011830-1.1						×	×	×	uncharacterized protein
Opuchr04_g0011840-1.1	OG0007971	1	1	1	2	×	×	×	probable tRNA N6-adenosine threonylcarbamoyltransferase
Opuchr04_g0011860-1.1	OG0003774	1	2	1	4	×	×	•	probable cyclic nucleotide-gated ion channel 20, chloroplastic
Opuchr04_g0011880-1.1	OG0003418	2	2	1	1	×	×	×	elongation factor 1-alpha-like
Opuchr04_g0011890-1.1	OG0001236	3	3	3	2	×	•	•	Mitochondrial import inner membrane translocase subunit TIM14-3, partial
Opuchr04_g0011900-1.1	OG0007172	1	2	2	1	×	•	•	putative D-cysteine desulfhydrase 1, mitochondrial
Opuchr04_g0011910-1.1	OG0000674	3	5	3	3	•	•	•	glutamate decarboxylase-like
Opuchr04_g0011920-1.1	OG0007489	1	1	1	2	×	•	•	DNA polymerase alpha/epsilon, subunit B
Opuchr04_g0011970-1.1	OG0011305	1	1	1	1	×	•	•	protein NRDE2 homolog
Opuchr04_g0011980-1.1	OG0001326	1	3	2	2	×	×	×	protein LURP-one-related 8-like
Opuchr04_g0011990-1.1	OG0003159	1	2	1	1	×	•	•	uncharacterized protein LOC113741941
Opuchr04_g0012000-1.1	OG0011237	1	1	1	1	×	•	•	probable arabinose 5-phosphate isomerase
Opuchr04_g0012010-1.1	OG0009158	1	1	1	0	×	•	×	thylakoid lumenal 17.9 kDa protein, chloroplastic-like
Opuchr04_g0012020-1.1	OG0006104	2	1	1	1	×	•	•	uncharacterized protein LOC113742114 isoform X5
Opuchr04_g0012030-1.1						×	×	×	uncharacterized protein LOC112177048 isoform X2
Opuchr04_g0012040-1.1	OG0000222	5	9	4	5	×	•	•	Redox 2 #
Opuchr04_g0012050-1.1	OG0000222	5	9	4	5	×	×	×	redox 2
Opuchr04_g0012060-1.1	OG0000693	9	2	2	1	×	×	×	peroxidase 1
Opuchr04_g0012080-1.1	OG0000222	5	9	4	5	×	×	×	redox 2
Opuchr04_g0013410-1.1						•	×	•	vinorine synthase-like
Opuchr04_g0010120-1.1	OG0003212	1	1	4	2	×	×	•	strictosidine beta-D-glucosidase
Opuchr04_g0010130-1.1	OG0001686	1	4	1	1	•	•	•	uncharacterized protein LOC113740142
Opuchr04_g0010140-1.1	OG0009434	1	1	1	1	•	•	•	oleosin 1-like
Opuchr04_g0010150-1.1	OG0014255	1	1	1	1	•	•	•	protein CHUP1, chloroplastic-like
Opuchr04_g0010160-1.1	OG0002233	2	3	2	3	•	•	•	translation initiation factor eIF-2B subunit delta-like isoform X3
Opuchr04_g0010180-1.1	OG0006148	1	1	1	1	•	•	•	UspA (IPR006016) / Rossmann-like alpha/beta/alpha sandwich fold
Opuchr04_g0010190-1.1	OG0012821	1	1	1	1	•	•	•	protein decapping 5-like
Opuchr04_g0010210-1.1	OG0011233	1	1	1	1	•	•	•	single-stranded DNA-binding protein, mitochondrial
Opuchr04_g0010220-1.1	OG0008273	1	1	1	1	•	•	•	Zinc finger, C3HC-like
Opuchr04_g0010240-1.1	OG0002690	2	2	2	2	•	•	•	piriformospora indica-insensitive protein 2-like isoform X1
Opuchr04_g0010250-1.1	OG0008363	1	1	1	1	×	•	•	cytochrome c-type biogenesis CcmH-like mitochondrial protein
Opuchr04_g0010260-1.1	OG0002334	3	2	2	1	•	•	×	mavicyanin-like
Opuchr04_g0010270-1.1	OG0005002	1	1	2	1	•	•	•	DEAD-box ATP-dependent RNA helicase 510
Opuchr04_g0010280-1.1	OG0007032	1	2	1	1	•	•	•	kinesin-like protein KIN-8A isoform X1
Opuchr04_g0010290-1.1	OG0005061	1	1	1	1	•	•	•	protein MIZU-KUSSEI 1-like
Opuchr04_g0010310-1.1	OG0006540	1	2	1	2	•	•	•	G patch domain-containing protein TGH-like
Opuchr04_g0010330-1.1	OG0000692	3	6	3	3	•	•	•	putative phospholipid-transporting ATPase 9 isoform X10
Opuchr04_g0010350-1.1	OG0011983	1	1	1	1	•	•	•	uncharacterized protein LOC113741806 isoform X2
Opuchr04_g0010360-1.1	OG0011638	1	1	1	1	•	•	•	ethylene-responsive transcription factor WIN1-like
Opuchr04_g0010370-1.1						×	×	×	F-box/kelch-repeat protein At3g23880-like
Opuchr04_g0010390-1.1	OG0010273	1	1	1	1	•	•	•	calcium sensing receptor, chloroplastic isoform X1
Opuchr04_g0010400-1.1	OG0005691	1	2	1	1	•	•	•	AMP-binding domain-containing protein
Opuchr04_g0010410-1.1	OG0007057	1	2	1	1	•	•	•	unnamed protein product
Opuchr04_g0010420-1.1	OG0001509	5	2	2	2	×	•	•	protein trichome birefringence-like 43
Opuchr04_g0010430-1.1	OG0009628	1	1	1	1	•	•	•	enolase 1, chloroplastic
Opuchr04_g0010440-1.1	OG0011058	1	1	1	1	•	•	•	plant cysteine oxidase 3
Opuchr04_g0010450-1.1	OG0006521	1	1	1	1	•	•	•	protein MIS12 homolog
Opuchr04_g0010460-1.1						×	×	•	strictosidine synthase
Opuchr04_g0010470-1.1						×	×	×	STRICTOSIDINE SYNTHASE-LIKE 12-like
Opuchr04_g0010480-1.1	OG0000178	53	0	0	0	×	×	×	uncharacterized protein LOC113729443
Opuchr04_g0010540-1.1	OG0013627	3	0	0	4	×	×	×	strictosidine synthase
Opuchr04_g0010550-1.1						×	×	×	Six-bladed beta-propeller
Opuchr04_g0010560-1.1	OG0000148	29	13	5	7	•	•	•	Strictosidine synthase
Opuchr04_g0010570-1.1	OG0000148	29	13	5	7	×	×	×	strictosidine synthase
Opuchr04_g0010580-1.1	OG0000148	29	13	5	7	×	×	×	Strictosidine synthase
Opuchr04_g0010590-1.1	OG0000148	29	13	5	7	×	×	×	strictosidine synthase
Opuchr04_g0010600-1.1						×	×	×	protein STRICTOSIDINE SYNTHASE-LIKE 11-like
Opuchr04_g0010610-1.1	OG0000148	29	13	5	7	×	×	×	strictosidine synthase
Opuchr04_g0010630-1.1	OG0000148	29	13	5	7	•	×	×	Strictosidine synthase
Opuchr04_g0010640-1.1	OG0010255	1	1	1	1	•	•	•	H/ACA ribonucleoprotein complex subunit 2-like protein
Opuchr04_g0010650-1.1	OG0002672	2	2	2	2	•	•	•	inorganic pyrophosphatase TTM1-like
Opuchr04_g0010660-1.1	OG0010162	1	1	1	1	•	•	•	unnamed protein product

① Number of genes assigned to an orthogroup from a specific species

② Presence or absence of genes from a syteny relationships with *O. pumila* { • Present × Absent }

Supplementary Fig. 43. Monoterpene indole alkaloid (MIA) gene-clusters identified in chromosome 4 of the *Ophiorrhiza* genome assembly. The position of the gene-cluster is scaled based on chromosome size and its physical position on the chromosome. The gene description, colored as orange, represents genes with functions associated with MIA biosynthesis. O.p: *Ophiorrhiza pumila*; C.a: *Camptotheca acuminata*; C.r: *Catharanthus roseus*; G.s: *Gelsemium sempervirens*.

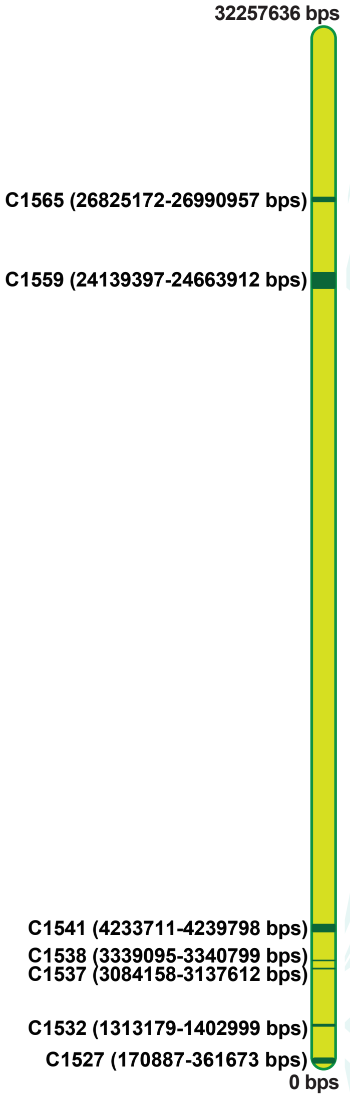

Gene id	Orthogroup	O. p (1)	C. a (1)	C. r (1)	G. s (1)	C. a (2)	C. r (2)	G. s (2)	Description
Opuchr05_g0058170-1.1	OG0012090	1	0	0	0	•	•	•	putative callose synthase 8 isoform X1
Opuchr05_g0058180-1.1	OG0011264	1	1	1	1	•	•	•	Tetratricopeptide-like helical domain superfamily
Opuchr05_g0058190-1.1	OG0003740	1	2	1	1	•	•	•	C2 domain-containing protein At1g53590-like
Opuchr05_g0058200-1.1	OG0006180	1	1	1	1	•	•	•	persulfide dioxygenase ETHE1 homolog, mitochondrial
Opuchr05_g0058210-1.1	OG0002937	2	2	1	1	•	•	•	60S ribosomal protein L18a-like protein
Opuchr05_g0058250-1.1	OG0000022	25	13	10	15	•	•	•	7-deoxyloganic acid 7-hydroxylase #
Opuchr05_g0058270-1.1	OG0000180	78	0	0	0	×	×	×	hypothetical protein VIGAN_05281600, partial
Opuchr05_g0058300-1.1	OG0000022	25	13	10	15	•	×	×	7-deoxyloganic acid 7-hydroxylase #
Opuchr05_g0058340-1.1	OG0000022	25	13	10	15	•	•	•	secologanin synthase
Opuchr05_g0058380-1.1	OG0000022	25	13	10	15	×	×	×	secologanin synthase
Opuchr05_g0058390-1.1	OG0002307	2	2	1	1	•	•	•	mitochondrial inner membrane protease subunit 1
Opuchr05_g0058410-1.1	OG0006200	1	2	1	1	•	×	×	probable solanesyl-diphosphate synthase 3, chloroplastic
Opuchr05_g0058420-1.1	OG0000636	1	2	2	3	•	•	•	PREDICTED: major pollen allergen Ole e 10-like
Opuchr05_g0058430-1.1	OG0007175	1	2	1	1	•	•	•	fatty-acid-binding protein 3, chloroplastic
Opuchr05_g0053440-1.1	OG0000022	25	13	10	15	×	•	×	secologanin synthase
Opuchr05_g0053510-1.1	OG0000022	25	13	10	15	×	×	×	7-deoxyloganic acid 7-hydroxylase
Opuchr05_g0053540-1.1						×	×	×	farnesyl diphosphate synthase
Opuchr05_g0053550-1.1	OG0014197	5	0	0	0	×	×	×	AAA-ATPase
Opuchr05_g0053590-1.1	OG0000022	25	13	10	15	×	×	×	secologanin synthase
Opuchr05_g0053600-1.1	OG0000022	25	13	10	15	×	×	×	secologanin synthase
Opuchr05_g0053610-1.1	OG0014261	1	0	0	1	×	×	×	unnamed protein product
Opuchr05_g0053620-1.1						×	×	×	uncharacterized protein LOC113755505
Opuchr05_g0053630-1.1	OG0000022	25	13	10	15	×	×	×	secologanin synthase
Opuchr05_g0053640-1.1						×	×	×	unnamed protein product
Opuchr05_g0053690-1.1	OG0001291	3	1	1	1	×	×	×	secologanin synthase
Opuchr05_g0053720-1.1						×	×	×	Protein BOBBER like
Opuchr05_g0053740-1.1	OG0000022	25	13	10	15	×	×	×	secologanin synthase
Opuchr05_g0053750-1.1						×	×	×	uncharacterized protein LOC113706943
Opuchr05_g0053810-1.1	OG0000022	25	13	10	15	×	×	×	secologanin synthase
Opuchr05_g0053850-1.1						×	×	×	hypothetical protein L484_003434
Opuchr05_g0053880-1.1						×	×	×	WD40-repeat-containing domain superfamily
Opuchr05_g0053910-1.1	OG0000022	25	13	10	15	×	×	×	7-deoxyloganic acid 7-hydroxylase
Opuchr05_g0053920-1.1	OG0002045	10	4	1	2	×	×	×	Inner centromere protein, ARK-binding domain
Opuchr05_g0053930-1.1	OG0000022	25	13	10	15	×	×	×	secologanin synthase
Opuchr05_g0053940-1.1						×	×	×	glutamate receptor 3.6-like isoform X1
Opuchr05_g0053950-1.1	OG0014838	1	0	0	0	×	×	×	uncharacterized protein
Opuchr05_g0053990-1.1	OG0001291	3	1	1	1	×	×	×	secologanin synthase
Opuchr05_g0054000-1.1	OG0014246	2	1	2	0	×	×	×	uncharacterized protein
Opuchr05_g0054020-1.1	OG0000022	25	13	10	15	×	×	×	secologanin synthase
Opuchr05_g0054040-1.1						×	×	×	Nuclear/nucleolar GTPase 2
Opuchr05_g0054050-1.1	OG0013935	1	0	2	3	×	•	•	secologanin synthase
Opuchr05_g0054070-1.1	OG0010077	1	1	1	1	×	×	×	Component of the U4/U6.U5 snRNP/mitosis protein DIM1
Opuchr05_g0054080-1.1	OG0000022	25	13	10	15	×	×	•	7-deoxyloganic acid 7-hydroxylase
Opuchr05_g0054090-1.1	OG0002398	4	0	2	2	×	×	×	homeobox-leucine zipper protein ANTHOCYANINLESS 2-like isoform X4
Opuchr05_g0054100-1.1						×	×	•	7-deoxyloganic acid 7-hydroxylase
Opuchr05_g0054130-1.1	OG0000022	25	13	10	15	×	×	×	7-deoxyloganic acid 7-hydroxylase
Opuchr05_g0054140-1.1	OG0000022	25	13	10	15	×	•	•	7-deoxyloganic acid 7-hydroxylase
Opuchr05_g0054200-1.1	OG0017253	1	0	0	1	×	×	×	cytochrome P450 CYP72A219-like
Opuchr05_g0054210-1.1						×	×	•	secologanin synthase
Opuchr05_g0054220-1.1						×	×	×	secologanin synthase
Opuchr05_g0008060-1.1	OG0000421	5	7	7	6	×	×	•	Multi antimicrobial extrusion protein
Opuchr05_g0008080-1.1	OG0000421	5	7	7	6	•	×	×	Multi antimicrobial extrusion protein
Opuchr05_g0008090-1.1	OG0004051	2	2	1	1	×	×	×	peroxidase 1
Opuchr05_g0008110-1.1						×	×	×	mitochondrial import inner membrane translocase subunit TIM23-2-like
Opuchr05_g0008130-1.1	OG0000191	47	0	0	33	×	×	×	uncharacterized protein LOC113711366
Opuchr05_g0008140-1.1						×	×	×	uncharacterized protein LOC113759893
Opuchr05_g0008160-1.1						×	×	×	amino acid transporter AVT1J
Opuchr05_g0008180-1.1	OG0015245	2	0	1	1	×	•	•	Strictosidine synthase #
Opuchr05_g0008210-1.1						×	×	×	uncharacterized protein
Opuchr05_g0008240-1.1	OG0000536	3	3	2	2	×	×	×	Amino acid transporter, transmembrane domain
Opuchr05_g0008250-1.1						×	×	×	strictosidine synthase
Opuchr05_g0008260-1.1						×	×	×	hypothetical protein CR513_15997, partial
Opuchr05_g0008270-1.1						×	×	×	strictosidine synthase
Opuchr05_g0008280-1.1						×	×	×	cytochrome P450 71A3-like
Opuchr05_g0008290-1.1	OG0000180	78	0	0	0	×	×	×	uncharacterized protein LOC111240713
Opuchr05_g0008300-1.1	OG0015245	2	0	1	1	×	×	×	Strictosidine synthase *
Opuchr05_g0008350-1.1						×	•	•	tryptophan decarboxylase #
Opuchr05_g0008360-1.1						×	×	×	putative NAC domain-containing protein 61, partial
Opuchr05_g0008370-1.1	OG0000823	4	4	2	3	×	×	×	tryptophan decarboxylase *
Opuchr05_g0008390-1.1	OG0000421	5	7	7	6	•	•	•	multi-antimicrobial extrusion-like protein
Opuchr05_g0008410-1.1	OG0000421	5	7	7	6	•	×	•	Multi antimicrobial extrusion protein
Opuchr05_g0006090-1.1	OG0000067	4	13	3	2	×	×	×	vinorine hydroxylase
Opuchr05_g0006100-1.1	OG0006048	1	2	1	1	•	•	•	xyloglucan endotransglycosylase/hydrolase 2
Opuchr05_g0006110-1.1	OG0010313	1	2	1	1	×	•	•	geraniol synthase #
Opuchr05_g0006120-1.1	OG0000047	13	11	4	7	•	×	×	geraniol synthase
Opuchr05_g0005520-1.1	OG0000823	4	4	2	3	•	×	•	tryptophan decarboxylase 2
Opuchr05_g0005530-1.1	OG0008024	1	1	1	1	•	•	•	Rieske iron-sulphur protein, C-terminal
Opuchr05_g0005540-1.1	OG0002466	1	3	1	2	•	•	•	putative cyclin-D6-1
Opuchr05_g0005550-1.1	OG0001045	2	4	2	2	•	•	•	SANT/Myb domain
Opuchr05_g0005560-1.1	OG0004222	3	0	1	3	×	×	•	UDP-glucose iridoid glucosyltransferase
Opuchr05_g0005580-1.1						×	×	×	7-deoxyloganetic acid glucosyltransferase
Opuchr05_g0005590-1.1	OG0004222	3	0	1	3	×	×	×	7-deoxyloganetic acid glucosyltransferase
Opuchr05_g0002460-1.1						×	×	×	O-methyltransferase
Opuchr05_g0002470-1.1	OG0000060	6	8	17	6	×	×	×	Tabersonine 16-O-methyltransferase
Opuchr05_g0002480-1.1						×	×	×	cytochrome P450 81D11-like
Opuchr05_g0002490-1.1						×	×	×	uncharacterized protein
Opuchr05_g0002520-1.1						×	×	×	vinorine hydroxylase
Opuchr05_g0002590-1.1	OG0000060	6	8	17	6	×	×	×	rankinidine/humantenine-11-O-methyltransferase
Opuchr05_g0002600-1.1	OG0013566	1	2	0	2	×	×	×	cytochrome P450 family 71 subfamily CR polypeptide 1
Opuchr05_g0002650-1.1	OG0004229	21	0	0	0	×	×	×	uncharacterized protein LOC113699335 isoform X2
Opuchr05_g0002670-1.1	OG0004583	2	4	1	0	×	×	×	vinorine hydroxylase
Opuchr05_g0002680-1.1	OG0008242	1	1	1	1	•	•	×	Acyl-CoA oxidase, C-terminal
Opuchr05_g0002690-1.1						×	×	×	uncharacterized protein
Opuchr05_g0002700-1.1	OG0001252	2	2	2	2	•	•	•	OPA3-like protein
Opuchr05_g0002710-1.1	OG0000060	6	8	17	6	•	•	•	10-hydroxycamptothecin O-methyltransferase
Opuchr05_g0002720-1.1	OG0000060	6	8	17	6	×	•	×	Tabersonine 16-O-methyltransferase
Opuchr05_g0000510-1.1						×	×	×	ATP sulfurylase 2
Opuchr05_g0000520-1.1	OG0002845	2	1	1	2	×	×	•	late embryogenesis abundant protein EMB564-like
Opuchr05_g0000540-1.1	OG0002212	1	1	0	2	×	×	•	cysteine-rich and transmembrane domain-containing protein B-like
Opuchr05_g0000550-1.1	OG0012112	1	2	1	1	×	×	•	7-deoxyloganetin glucosyltransferase
Opuchr05_g0000560-1.1	OG0002350	2	3	2	2	•	×	•	OTU domain-containing protein DDB_G0284757
Opuchr05_g0000570-1.1	OG0001246	2	3	2	2	×	•	•	protein PAT1 homolog 1
Opuchr05_g0000610-1.1	OG0002880	2	1	2	1	×	•	•	UDP-arabinose 4-epimerase 1-like
Opuchr05_g0000620-1.1	OG0006944	1	2	1	1	×	•	×	heat shock 70 kDa protein 17
Opuchr05_g0000630-1.1	OG0000114	7	6	6	3	×	×	×	glutamate receptor 2.8-like
Opuchr05_g0000640-1.1	OG0008182	2	1	1	1	×	•	×	perakine reductase
Opuchr05_g0000650-1.1	OG0012412	1	1	1	0	×	•	×	transcription factor PHYTOCHROME INTERACTING FACTOR-LIKE 13-like
Opuchr05_g0000670-1.1	OG0006802	1	1	1	1	×	•	×	FAD/NAD(P)-binding domain superfamily (IPR036188)
Opuchr05_g0000680-1.1	OG0011218	1	1	1	1	×	•	•	pentatricopeptide repeat-containing protein At3g59040-like
Opuchr05_g0000690-1.1	OG0001742	6	1	1	7	×	×	×	geissoschizine oxidase
Opuchr05_g0000700-1.1	OG0001742	6	1	1	7	×	×	×	Cytochrome P450 87A3 like
Opuchr05_g0000710-1.1	OG0012560	1	2	0	0	•	×	•	Multi antimicrobial extrusion protein
Opuchr05_g0000730-1.1	OG0003167	1	3	2	2	•	•	×	auxin response factor 6-like
Opuchr05_g0000740-1.1	OG0012431	1	1	1	1	×	•	•	pentatricopeptide repeat-containing protein At2g35030, mitochondrial-like
Opuchr05_g0000750-1.1	OG0002940	2	2	2	2	•	•	•	N-acetyl-D-glucosamine kinase-like
Opuchr05_g0000780-1.1	OG0004974	1	2	1	1	•	•	•	DEAD-box ATP-dependent RNA helicase 53, mitochondrial-like
Opuchr05_g0000790-1.1	OG0000291	7	2	13	6	×	×	×	tabersonine-19-hydroxy-O-acetyltransferase
Opuchr05_g0000810-1.1	OG0000424	4	4	2	3	•	×	•	Zinc finger, RING/FYVE/PHD-type
Opuchr05_g0000820-1.1	OG0005626	1	1	1	2	×	×	•	endoplasmic reticulum-Golgi intermediate compartment protein 3
Opuchr05_g0000830-1.1	OG0007923	1	1	2	1	×	×	•	membrane-associated progesterone-binding protein 4-like isoform X1
Opuchr05_g0000850-1.1	OG0016714	1	0	0	0	×	×	×	Squalene/phytoene synthase
Opuchr05_g0000860-1.1	OG0010773	1	1	0	1	×	×	×	RNA polymerases N / 8 kDa subunit
Opuchr05_g0000870-1.1	OG0011835	1	1	1	1	×	•	•	glucuronoxylan 4-O-methyltransferase 3-like
Opuchr05_g0000880-1.1	OG0005151	2	2	2	2	×	•	•	Pyridoxal phosphate-dependent transferase, major domain

(1) Number of genes assigned to an orthogroup from a specific species

(2) Presence or absence of genes from a syteny relationships with *O. pumila* { • Present × Absent }

Supplementary Fig. 44. Monoterpene indole alkaloid (MIA) gene-clusters identified in chromosome 5 of the *Ophiorrhiza* genome assembly. The position of the gene-cluster is scaled based on chromosome size and its physical position on the chromosome. The gene description, colored as orange, represents genes with functions associated with MIA biosynthesis. O.p: *Ophiorrhiza pumila*; C.a: *Camptotheca acuminata*; C.r: *Catharanthus roseus*; G.s: *Gelsemium sempervirens*.

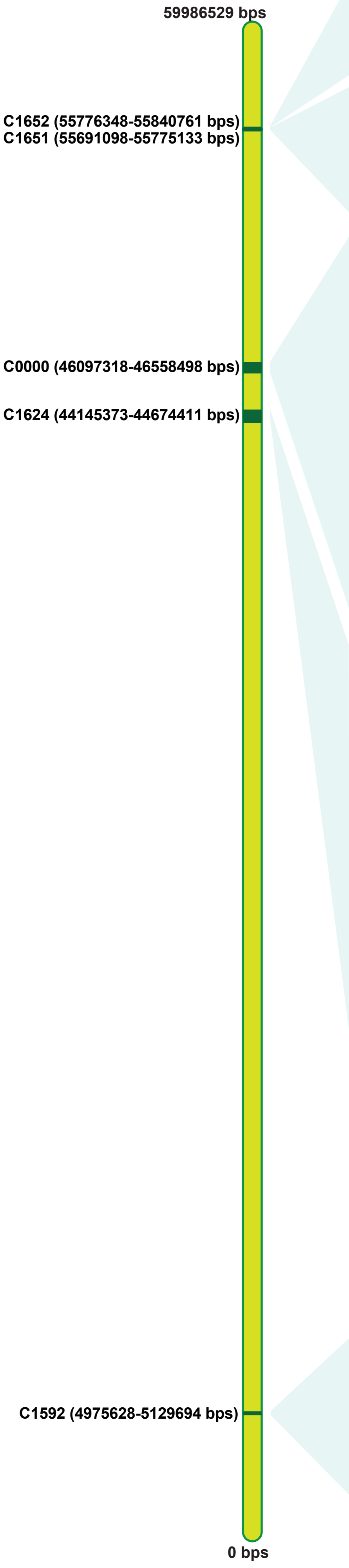

Gene id	Orthogroup	① O. p	① C. a	① C. r	① G. s	② C. a	② C. r	② G. s	Description
Opuchr06_g0113730-1.1	OG0002046	2	6	1	1	•	•	•	7-deoxyloganetic acid synthase #
Opuchr06_g0113760-1.1	OG0002046	2	6	1	1	×	×	•	7-deoxyloganetic acid synthase
Opuchr06_g0113770-1.1	OG0007265	2	1	2	3	•	×	•	7-deoxyloganetic acid synthase
Opuchr06_g0113780-1.1	OG0007545	1	2	1	1	•	•	•	probable 3-deoxy-D-manno-octulosonic acid transferase
Opuchr06_g0113790-1.1	OG0006798	1	2	1	1	•	•	•	leucine-rich repeat receptor-like protein kinase PXC1
Opuchr06_g0113800-1.1	OG0003811	2	4	1	1	•	•	•	uncharacterized protein LOC113776178 isoform X2
Opuchr06_g0113810-1.1	OG0002234	2	2	2	2	•	•	•	Pyruvate kinase
Opuchr06_g0113820-1.1	OG0003796	2	2	1	1	•	•	•	uncharacterized protein LOC113694709 isoform X1

Gene id	Orthogroup	① O. p	① C. a	① C. r	① G. s	② C. a	② C. r	② G. s	Description
Opuchr06_g0113530-1.1	OG0008470	1	1	1	1	•	•	•	delta(14)-sterol reductase
Opuchr06_g0113540-1.1	OG0000633	3	5	1	1	•	•	•	histone deacetylase HDT1-like isoform X1
Opuchr06_g0113550-1.1	OG0000807	2	3	2	3	•	•	•	splicing factor U2af small subunit B-like
Opuchr06_g0113570-1.1	OG0004674	1	1	1	1	•	•	•	enolase
Opuchr06_g0113580-1.1	OG0006303	2	1	1	1	•	•	•	peroxiredoxin-2E-2, chloroplastic-like
Opuchr06_g0113660-1.1	OG0000598	3	7	3	3	•	•	•	glucomannan 4-beta-mannosyltransferase 9
Opuchr06_g0113670-1.1	OG0004591	5	2	0	1	×	×	×	uncharacterized protein At2g27730, mitochondrial-like isoform X3
Opuchr06_g0113680-1.1	OG0004536	1	1	2	2	×	×	×	uncharacterized protein LOC113695442
Opuchr06_g0113700-1.1	OG0000273	3	4	2	3	•	×	×	uncharacterized protein LOC113697438 isoform X2
Opuchr06_g0113710-1.1	OG0006603	1	2	1	1	•	•	•	AT-hook motif nuclear-localized protein 7-like
Opuchr06_g0113720-1.1	OG0012375	1	2	1	1	•	•	•	unnamed protein product

Gene id	Orthogroup	① O. p	① C. a	① C. r	① G. s	② C. a	② C. r	② G. s	Description
Opuchr06_g0097330-1.1	OG0000118	17	8	1	3	×	×	×	rankinidine/humantenine-11-hydroxylase 3
Opuchr06_g0097420-1.1						×	×	×	uncharacterized protein LOC113708247
Opuchr06_g0097430-1.1						×	×	×	uncharacterized protein
Opuchr06_g0097440-1.1						×	×	×	uncharacterized protein
Opuchr06_g0097450-1.1						×	×	×	uncharacterized protein LOC113723305
Opuchr06_g0097460-1.1	OG0000118	17	8	1	3	×	×	×	rankinidine/humantenine-11-hydroxylase 3
Opuchr06_g0097490-1.1						×	×	×	uncharacterized protein LOC108660713
Opuchr06_g0097500-1.1	OG0004775	18	0	0	0	×	×	×	hypothetical protein TorRG33x02_048750
Opuchr06_g0097600-1.1	OG0000473	8	2	3	2	×	×	×	ABC transporter-like
Opuchr06_g0097670-1.1						×	×	×	putative U-box domain-containing protein 50 isoform X2
Opuchr06_g0097680-1.1	OG0010047	3	2	1	1	×	×	×	putative U-box domain-containing protein 50 isoform X2
Opuchr06_g0097690-1.1						×	×	×	probable F-box protein At4g22030
Opuchr06_g0097700-1.1	OG0000118	17	8	1	3	×	×	×	rankinidine/humantenine-11-hydroxylase 3
Opuchr06_g0097710-1.1	OG0014658	3	0	0	0	×	×	×	hypothetical protein CRG98_035434, partial
Opuchr06_g0097720-1.1						×	×	×	actin-related protein 5 isoform X2
Opuchr06_g0097730-1.1	OG0003605	5	2	1	1	×	×	×	Calcineurin-like phosphoesterase domain, ApaH type
Opuchr06_g0097770-1.1						×	×	×	uncharacterized protein LOC108220559
Opuchr06_g0097780-1.1	OG0000872	7	0	0	1	×	×	×	Defensin-like protein 1
Opuchr06_g0097810-1.1						×	×	×	rankinidine/humantenine-11-hydroxylase 3
Opuchr06_g0097820-1.1	OG0001738	29	0	0	0	×	×	×	uncharacterized protein
Opuchr06_g0097880-1.1						×	×	×	Endonuclease/exonuclease/phosphatase superfamily (IPR036691)
Opuchr06_g0097900-1.1	OG0006632	17	0	0	0	×	×	×	uncharacterized protein LOC113696826
Opuchr06_g0097960-1.1						×	×	×	hypothetical protein PanWU01x14_251180, partial
Opuchr06_g0097990-1.1						×	×	×	hypothetical protein CDL12_03455
Opuchr06_g0098090-1.1	OG0000118	17	8	1	3	×	×	×	tabersonine 6,7-epoxidase 1
Opuchr06_g0098150-1.1	OG0013268	2	0	0	0	×	×	×	ABC transporter G family member 29-like
Opuchr06_g0098160-1.1						×	×	×	ACT domain
Opuchr06_g0098190-1.1						×	×	×	hypothetical protein TanjilG_11455
Opuchr06_g0098200-1.1	OG0014129	1	0	0	0	×	×	×	Ribosomal protein L7A/L8
Opuchr06_g0098210-1.1						×	×	×	Protein kinase domain
Opuchr06_g0098250-1.1	OG0000118	17	8	1	3	×	×	×	rankinidine/humantenine-11-hydroxylase 3
Opuchr06_g0098260-1.1	OG0000626	43	1	0	1	×	×	×	uncharacterized protein LOC113719845, partial
Opuchr06_g0098330-1.1	OG0000118	17	8	1	3	×	×	×	rankinidine/humantenine-11-hydroxylase 3

Gene id	Orthogroup	① O. p	① C. a	① C. r	① G. s	② C. a	② C. r	② G. s	Description
Opuchr06_g0093040-1.1	OG0000291	7	2	13	6	×	×	×	vinorine synthase
Opuchr06_g0093060-1.1						×	×	×	uncharacterized protein
Opuchr06_g0093080-1.1	OG0005113	3	1	1	3	×	•	×	pentatricopeptide repeat-containing protein At5g57250, mitochondrial
Opuchr06_g0093110-1.1	OG0012825	3	1	1	0	×	×	×	B3 domain-containing protein REM5-like
Opuchr06_g0093160-1.1	OG0003150	2	2	1	4	×	×	×	60S ribosomal protein L37-3
Opuchr06_g0093230-1.1						×	×	×	uncharacterized protein LOC113739276
Opuchr06_g0093270-1.1	OG0000180	78	0	0	0	×	×	×	Papain-like cysteine peptidase superfamily
Opuchr06_g0093330-1.1						×	×	×	uncharacterized protein
Opuchr06_g0093340-1.1						×	×	×	uncharacterized protein LOC113689119
Opuchr06_g0093350-1.1						×	×	×	uncharacterized protein
Opuchr06_g0093370-1.1						×	×	×	uncharacterized protein
Opuchr06_g0093430-1.1						×	×	×	uncharacterized protein
Opuchr06_g0093470-1.1						×	×	×	uncharacterized protein LOC112092850
Opuchr06_g0093530-1.1						×	×	×	uncharacterized protein
Opuchr06_g0093540-1.1	OG0011699	13	0	0	0	×	×	×	uncharacterized protein LOC109183381
Opuchr06_g0093610-1.1						×	×	×	dihydropyrimidine dehydrogenase (NADP(+)), chloroplastic
Opuchr06_g0093620-1.1	OG0000748	2	3	1	1	×	×	×	EF-Hand 1, calcium-binding site
Opuchr06_g0093630-1.1	OG0000080	6	18	1	0	×	×	×	Protein kinase domain
Opuchr06_g0093650-1.1	OG0000080	6	18	1	0	×	×	×	Malectin-like carbohydrate-binding domain
Opuchr06_g0093660-1.1						×	×	×	uncharacterized protein At2g27730, mitochondrial-like isoform X3
Opuchr06_g0093670-1.1	OG0004168	2	1	1	1	×	×	×	DDT domain-containing protein DDB_G0282237-like
Opuchr06_g0093690-1.1	OG0000057	24	18	7	17	×	×	×	strictosidine beta-D-glucosidase
Opuchr06_g0093710-1.1	OG0000180	78	0	0	0	×	×	×	hypothetical protein glysoja_018320
Opuchr06_g0093720-1.1	OG0013271	4	0	0	1	×	×	×	serine/threonine-protein kinase ATM
Opuchr06_g0093730-1.1	OG0017289	1	0	0	1	×	×	×	uncharacterized protein
Opuchr06_g0093740-1.1	OG0000057	24	18	7	17	×	×	×	strictosidine beta-D-glucosidase
Opuchr06_g0093750-1.1						×	×	×	SKP1-like protein 1A
Opuchr06_g0093780-1.1	OG0006038	3	1	0	1	×	×	×	hypothetical protein Csa_2G382530
Opuchr06_g0093800-1.1						×	×	×	uncharacterized protein LOC105641421
Opuchr06_g0093810-1.1	OG0000797	5	5	2	1	×	×	×	uncharacterized protein LOC113754190
Opuchr06_g0093820-1.1						×	×	×	Protein misato like
Opuchr06_g0093830-1.1	OG0010376	6	0	5	0	×	×	×	polyneuridine-aldehyde esterase
Opuchr06_g0093840-1.1	OG0002829	3	3	1	1	×	×	×	cadmium/zinc-transporting ATPase HMA3-like
Opuchr06_g0093870-1.1	OG0000914	36	0	0	0	×	×	×	Endonuclease/exonuclease/phosphatase superfamily
Opuchr06_g0093980-1.1	OG0002829	3	3	1	1	×	×	×	cadmium/zinc-transporting ATPase HMA3-like

Gene id	Orthogroup	① O. p	① C. a	① C. r	① G. s	② C. a	② C. r	② G. s	Description
Opuchr06_g0007620-1.1	OG0008895	1	1	1	1	•	•	•	ABC transporter-like
Opuchr06_g0007630-1.1	OG0011761	1	1	1	0	•	•	×	tRNA A64-2'-O-ribosylphosphate transferase-like isoform X1
Opuchr06_g0007640-1.1	OG0003990	1	2	1	1	•	•	•	Senescence/spartin-associated
Opuchr06_g0007660-1.1	OG0005220	1	3	1	1	•	•	•	uncharacterized protein LOC113691393
Opuchr06_g0007670-1.1	OG0008946	1	1	1	1	•	•	•	rRNA methyltransferase 1, mitochondrial-like
Opuchr06_g0007680-1.1	OG0000709	4	2	13	4	×	×	×	hydrolase 3
Opuchr06_g0007690-1.1	OG0010609	1	1	1	1	•	•	•	probable 3-hydroxyisobutyrate dehydrogenase-like 1, mitochondrial
Opuchr06_g0007720-1.1	OG0000357	2	4	1	4	•	•	•	receptor-like protein kinase HSL1
Opuchr06_g0007730-1.1	OG0002253	2	2	2	2	•	•	•	hexokinase-2-like
Opuchr06_g0007740-1.1	OG0013030	1	1	1	1	•	•	•	unnamed protein product
Opuchr06_g0007760-1.1	OG0006924	1	2	1	1	•	•	•	Multi antimicrobial extrusion protein
Opuchr06_g0007780-1.1	OG0001474	2	3	2	2	•	•	•	protein IQ-DOMAIN 14-like isoform X2
Opuchr06_g0007820-1.1	OG0010062	1	1	1	1	•	•	•	probable pectinesterase 53
Opuchr06_g0007830-1.1	OG0006040	1	1	1	1	•	•	•	probable glutamate carboxypeptidase LAMP1

① Number of genes assigned to an orthogroup from a specific species

② Presence or absence of genes from a syteny relationships with *O. pumila* { • Present × Absent }

Supplementary Fig. 45. Monoterpene indole alkaloid (MIA) gene-clusters identified in chromosome 6 of the *Ophiorrhiza* genome assembly. The position of the gene-cluster is scaled based on chromosome size and its physical position on the chromosome. The gene description, colored as orange, represents genes with functions associated with MIA biosynthesis. O.p: *Ophiorrhiza pumila*; C.a: *Camptotheca acuminata*; C.r: *Catharanthus roseus*; G.s: *Gelsemium sempervirens*.

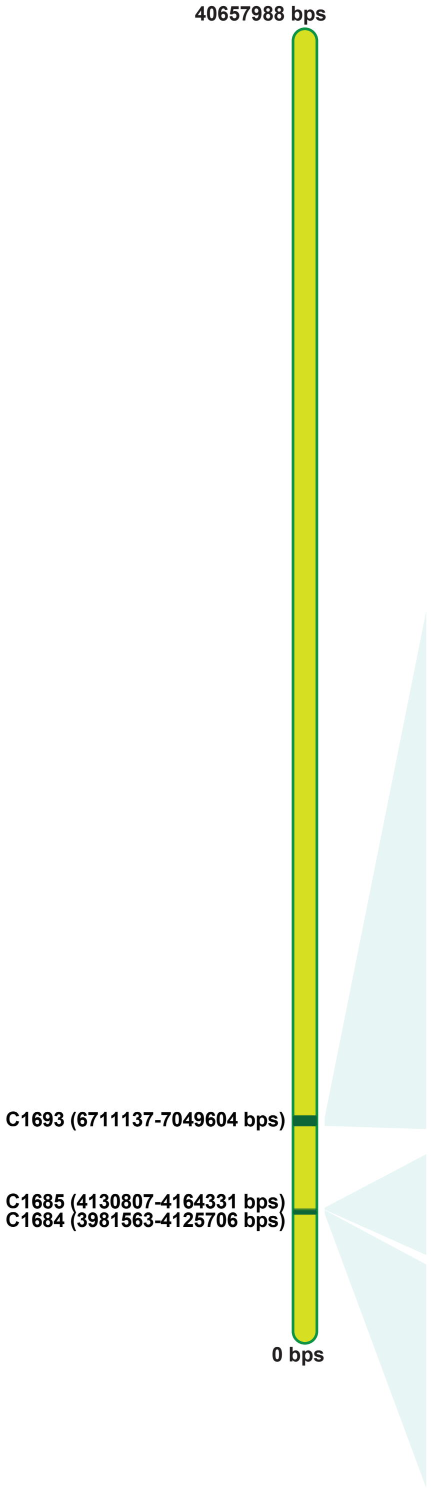

Gene id	Orthogroup	① O.p	① C.a	① C.r	① G.s	② C.a	② C.r	② G.s	Description
Opuchr07_g0011910-1.1	OG0000111	8	10	6	9	•	•	•	UDP-glucose iridoid glucosyltransferase
Opuchr07_g0011920-1.1						×	×	×	Mitochondrial glycoprotein
Opuchr07_g0011930-1.1	OG0000111	8	10	6	9	×	•	•	7-deoxyloganetin glucosyltransferase
Opuchr07_g0011950-1.1	OG0000111	8	10	6	9	×	•	×	UDP-glucose iridoid glucosyltransferase
Opuchr07_g0011960-1.1	OG0000111	8	10	6	9	×	×	•	7-deoxyloganetin glucosyltransferase
Opuchr07_g0011970-1.1	OG0014269	1	1	0	1	×	×	×	PREDICTED: UDP-glycosyltransferase 91D1-like
Opuchr07_g0011980-1.1	OG0000111	8	10	6	9	•	×	×	UDP-glucose iridoid glucosyltransferase
Opuchr07_g0011990-1.1	OG0000111	8	10	6	9	•	•	×	7-deoxyloganetin glucosyltransferase
Opuchr07_g0012000-1.1						•	×	×	7-deoxyloganetin glucosyltransferase
Opuchr07_g0012010-1.1	OG0001390	2	3	1	2	•	•	•	K Homology domain
Opuchr07_g0012020-1.1	OG0003534	2	3	2	2	•	•	×	DXS1
Opuchr07_g0012040-1.1	OG0015273	1	0	1	0	×	•	×	metal transporter Nramp5-like isoform X1
Opuchr07_g0012050-1.1	OG0006045	1	2	1	1	•	•	•	Exostosin-like
Opuchr07_g0012060-1.1	OG0001423	7	3	4	2	•	•	•	methylmalonate-semialdehyde dehydrogenase
Opuchr07_g0012070-1.1	OG0004687	1	2	1	1	•	•	•	Kinesin motor domain
Opuchr07_g0012080-1.1	OG0005067	1	2	1	1	•	•	•	Zinc finger, Dof-type
Opuchr07_g0012090-1.1	OG0014934	1	0	1	0	•	×	•	PREDICTED: 8-hydroxygeraniol dehydrogenase-like
Opuchr07_g0012100-1.1	OG0008433	1	1	1	1	×	•	•	elongator complex protein 4
Opuchr07_g0012120-1.1	OG0015226	4	0	0	0	×	×	×	uncharacterized protein LOC113757157
Opuchr07_g0012130-1.1						×	×	×	PREDICTED: mavicyanin-like
Opuchr07_g0012140-1.1	OG0000163	5	16	9	6	•	•	×	10-hydroxygeraniol oxidoreductase
Opuchr07_g0012150-1.1	OG0013584	2	0	0	1	×	×	×	Redox 1
Opuchr07_g0012160-1.1	OG0000066	15	8	13	19	×	•	•	polyneuridine aldehyde esterase
Opuchr07_g0012180-1.1	OG0002003	7	1	0	1	×	×	×	ABC transporter-like
Opuchr07_g0012210-1.1	OG0014643	3	0	0	2	×	•	×	O-acetylstemmadenine oxidase #
Opuchr07_g0012230-1.1	OG0015274	3	0	1	0	×	•	×	Tetrahydroalstonine synthase 3 #
Opuchr07_g0012240-1.1	OG0013584	2	0	0	1	×	•	×	10-hydroxygeraniol oxidoreductase
Opuchr07_g0012250-1.1	OG0013119	2	0	8	0	×	×	×	Tetrahydroalstonine synthase
Opuchr07_g0012260-1.1	OG0013119	2	0	8	0	×	×	•	Tetrahydroalstonine synthase
Opuchr07_g0012270-1.1						•	•	•	10-hydroxygeraniol oxidoreductase #
Opuchr07_g0012280-1.1	OG0000163	5	16	9	6	×	×	×	10-hydroxygeraniol oxidoreductase
Opuchr07_g0012300-1.1	OG0000124	3	7	7	3	•	•	•	probable rhamnogalacturonate lyase B
Opuchr07_g0012310-1.1	OG0000124	3	7	7	3	×	×	×	probable rhamnogalacturonate lyase B
Opuchr07_g0012320-1.1	OG0002804	2	2	1	2	•	×	•	DELLA protein GAI-like
Opuchr07_g0012330-1.1	OG0012628	1	0	1	1	•	•	•	glutathione S-transferase DHAR3, chloroplastic-like
Opuchr07_g0012340-1.1	OG0013343	1	1	1	1	•	•	•	geranylgeranyl pyrophosphate synthase 7, chloroplastic-like
Opuchr07_g0007500-1.1	OG0003153	2	3	1	2	•	•	•	peroxidase 1
Opuchr07_g0007510-1.1	OG0002827	2	2	2	2	•	•	•	peptidyl-prolyl cis-trans isomerase CYP40-like isoform X1
Opuchr07_g0007530-1.1	OG0003860	2	3	0	1	•	•	•	Patatin-like phospholipase domain
Opuchr07_g0007540-1.1	OG0003860	2	3	0	1	•	×	•	patatin-like protein 2
Opuchr07_g0007550-1.1	OG0003798	1	2	1	1	•	•	•	Phosphoglycerate/bisphosphoglycerate mutase
Opuchr07_g0007570-1.1						•	•	•	CDPK-related protein kinase
Opuchr07_g0007580-1.1	OG0005633	1	2	1	1	•	•	•	3-ketodihydrosphingosine reductase isoform X2
Opuchr07_g0007230-1.1	OG0000066	15	8	13	19	•	•	•	polyneuridine-aldehyde esterase
Opuchr07_g0007240-1.1	OG0000066	15	8	13	19	×	•	•	polyneuridine aldehyde esterase
Opuchr07_g0007250-1.1	OG0000040	22	6	3	13	•	•	•	geraniol 10-hydroxylase * #
Opuchr07_g0007260-1.1	OG0007560	1	1	1	1	•	•	•	autophagy-related protein 101-like isoform X2
Opuchr07_g0007340-1.1						×	×	×	uncharacterized protein LOC113755505
Opuchr07_g0007360-1.1	OG0000088	35	4	67	0	×	×	×	uncharacterized protein LOC113755505
Opuchr07_g0007380-1.1	OG0010342	1	2	1	1	•	•	•	dof zinc finger protein DOF5.7-like
Opuchr07_g0007390-1.1	OG0013117	1	1	2	2	•	•	•	Multicopper oxidase, type 1
Opuchr07_g0007400-1.1	OG0011736	1	1	1	1	•	•	•	reticulon-like protein B13
Opuchr07_g0007410-1.1	OG0001529	2	3	2	2	•	•	•	Protein kinase domain
Opuchr07_g0007420-1.1	OG0003435	2	1	2	1	•	•	•	upstream activation factor subunit spp27-like
Opuchr07_g0007440-1.1	OG0001953	3	3	2	2	•	•	•	transcription factor HHO5-like
Opuchr07_g0007470-1.1	OG0000231	5	7	4	4	•	•	•	Lateral organ boundaries
Opuchr07_g0007480-1.1	OG0008824	2	1	1	1	•	•	•	Ribosomal protein L25/Gln-tRNA synthetase
Opuchr07_g0007490-1.1	OG0001487	2	3	1	1	•	•	•	mitogen-activated protein kinase kinase kinase 5

① **Number of genes assigned to an orthogroup from a specific species**

② **Presence or absence of genes from a syteny relationships with *O. pumila*** { • Present × Absent }

Supplementary Fig. 46. Monoterpene indole alkaloid (MIA) gene-clusters identified in chromosome 7 of the *Ophiorrhiza* genome assembly. The position of the gene-cluster is scaled based on chromosome size and its physical position on the chromosome. The gene description, colored as orange, represents genes with functions associated with MIA biosynthesis. O.p: *Ophiorrhiza pumila;* C.a: *Camptotheca acuminata;* C.r: *Catharanthus roseus;* G.s: *Gelsemium sempervirens.*

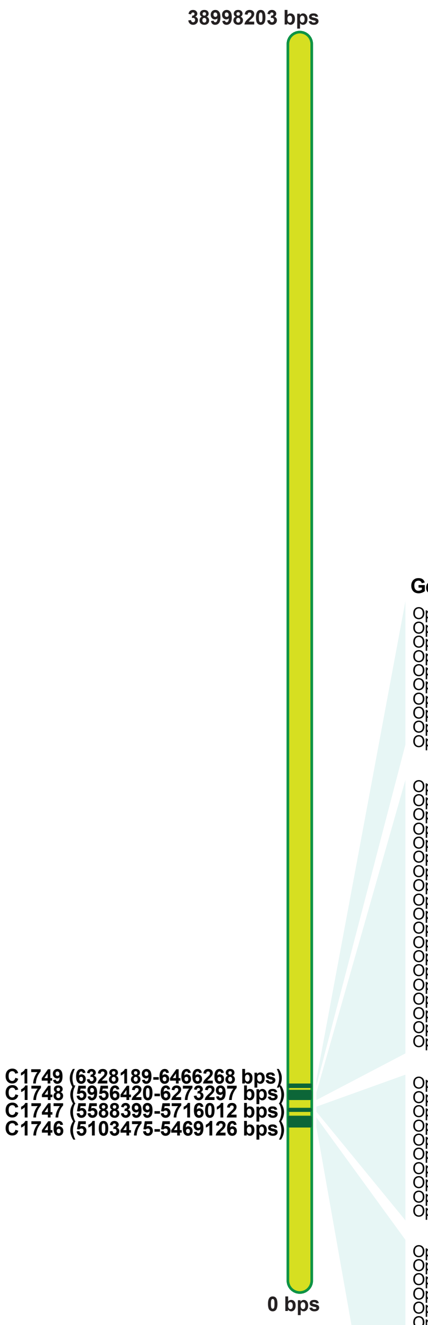

		1				2			
Gene id	Orthogroup	O.p	C.a	C.r	G.s	C.a	C.r	G.s	Description
Opuchr08_g0011230-1.1						×	×	×	Sarpagan Bridge Enzyme
Opuchr08_g0011240-1.1	OG0007700	16	0	0	0	×	×	×	uncharacterized protein
Opuchr08_g0011270-1.1						×	×	×	Sarpagan Bridge Enzyme
Opuchr08_g0011280-1.1	OG0002007	4	1	1	3	×	×	×	geraniol synthase
Opuchr08_g0011290-1.1						×	×	×	uncharacterized protein LOC113690632
Opuchr08_g0011340-1.1	OG0014617	5	0	0	0	×	×	×	hypothetical protein CDL12_26059
Opuchr08_g0011350-1.1	OG0010319	1	1	2	3	×	×	×	Terpenoid cyclases/protein prenyltransferase alpha-alpha toroid
Opuchr08_g0011410-1.1						×	×	•	Cytochrome P450
Opuchr08_g0011430-1.1	OG0000007	40	13	26	24	×	×	×	Tabersonine 3-oxygenase
Opuchr08_g0011440-1.1	OG0014672	2	0	0	2	×	×	×	geraniol synthase
Opuchr08_g0010560-1.1	OG0000043	9	11	11	10	×	×	×	geraniol synthase
Opuchr08_g0010570-1.1						×	×	×	uncharacterized protein
Opuchr08_g0010580-1.1						×	×	×	ALA-interacting subunit 3-like
Opuchr08_g0010650-1.1	OG0000007	40	13	26	24	×	×	×	tabersonine 3-oxygenase
Opuchr08_g0010700-1.1	OG0000178	53	0	0	0	×	×	×	uncharacterized protein LOC113704124
Opuchr08_g0010710-1.1						×	×	×	Tabersonine 3-oxygenase
Opuchr08_g0010720-1.1						×	×	×	uncharacterized protein
Opuchr08_g0010750-1.1						×	×	×	Tabersonine 3-oxygenase
Opuchr08_g0010760-1.1	OG0011713	2	0	9	1	×	×	×	Sarpagan Bridge Enzyme
Opuchr08_g0010940-1.1	OG0004775	18	0	0	0	×	×	×	hypothetical protein L484_012563
Opuchr08_g0010950-1.1	OG0014672	2	0	0	2	×	×	×	geraniol synthase
Opuchr08_g0011020-1.1	OG0004231	21	0	0	0	×	×	×	uncharacterized protein LOC113693287
Opuchr08_g0011030-1.1	OG0005775	12	0	0	0	×	×	×	uncharacterized protein LOC113728951
Opuchr08_g0011040-1.1						×	×	×	geraniol synthase
Opuchr08_g0011060-1.1	OG0002889	1	4	1	2	•	•	•	probable folate-biopterin transporter 2
Opuchr08_g0011080-1.1	OG0006449	1	3	1	1	×	×	•	mediator of RNA polymerase II transcription subunit 30-like
Opuchr08_g0011090-1.1	OG0004145	2	2	2	2	•	•	•	ubiquitin carboxyl-terminal hydrolase 22
Opuchr08_g0011110-1.1						×	×	×	Rhodanese-like domain-containing protein 14, chloroplastic, partial
Opuchr08_g0011140-1.1						×	×	×	hypothetical protein EUGRSUZ_C00375, partial
Opuchr08_g0009960-1.1	OG0000060	6	8	17	6	×	×	×	rankinidine/humantenine-11-O-methyltransferase
Opuchr08_g0009970-1.1	OG0000060	6	8	17	6	×	×	×	10-hydroxycamptothecin O-methyltransferase
Opuchr08_g0010000-1.1						×	×	×	hypothetical protein CDL12_09769
Opuchr08_g0010010-1.1	OG0013612	8	0	0	0	×	×	×	hypothetical protein CFOL_v3_27647
Opuchr08_g0010040-1.1	OG0000043	9	11	11	10	×	×	×	geraniol synthase
Opuchr08_g0010050-1.1	OG0001817	2	3	2	1	•	•	•	ClpP/crotonase-like domain superfamily
Opuchr08_g0010070-1.1	OG0009184	1	1	1	1	•	•	•	BTB/POZ domain-containing protein NPY1
Opuchr08_g0010100-1.1	OG0000428	7	3	4	11	×	×	•	Geraniol 10-hydroxylase
Opuchr08_g0010110-1.1	OG0000350	5	6	3	3	×	×	×	patatin-like protein 1
Opuchr08_g0010120-1.1	OG0000350	5	6	3	3	×	×	×	PREDICTED: patatin-like protein 2
Opuchr08_g0009230-1.1	OG0008683	1	1	1	1	•	×	×	chorismate mutase 2-like
Opuchr08_g0009240-1.1						×	×	×	Cyclophilin-like domain superfamily
Opuchr08_g0009250-1.1	OG0011898	1	1	1	0	×	×	×	Polar localization during asymmetric division and redistribution
Opuchr08_g0009280-1.1	OG0000178	53	0	0	0	×	×	×	uncharacterized protein LOC113729443
Opuchr08_g0009340-1.1	OG0000007	40	13	26	24	×	×	×	tabersonine 3-oxygenase
Opuchr08_g0009430-1.1	OG0011699	13	0	0	0	×	×	×	geraniol synthase
Opuchr08_g0009440-1.1	OG0000043	9	11	11	10	×	×	×	viridiflorene synthase-like
Opuchr08_g0009450-1.1						×	×	×	uncharacterized protein
Opuchr08_g0009460-1.1	OG0015287	1	0	0	2	×	×	×	tabersonine 6,7-epoxidase isoform 1
Opuchr08_g0009480-1.1	OG0002395	5	0	22	0	×	×	×	uncharacterized protein LOC113718472
Opuchr08_g0009540-1.1	OG0000923	8	0	2	0	×	×	×	NdhH (chloroplast)
Opuchr08_g0009550-1.1						•	×	×	geraniol synthase
Opuchr08_g0009580-1.1	OG0000007	40	13	26	24	×	×	×	tabersonine 3-oxygenase
Opuchr08_g0009590-1.1						×	×	×	unnamed protein product
Opuchr08_g0009600-1.1	OG0000007	40	13	26	24	×	×	×	tabersonine 3-oxygenase
Opuchr08_g0009650-1.1	OG0015288	1	2	1	0	×	×	×	11S globulin subunit beta-like isoform X1
Opuchr08_g0009660-1.1	OG0000040	22	6	3	13	×	×	×	geraniol 10-hydroxylase
Opuchr08_g0009690-1.1						×	×	×	uncharacterized protein LOC100798851
Opuchr08_g0009700-1.1	OG0000043	9	11	11	10	×	×	×	geraniol synthase
Opuchr08_g0009730-1.1	OG0002045	10	4	1	2	×	×	×	uncharacterized protein LOC113689764
Opuchr08_g0009740-1.1	OG0000043	9	11	11	10	×	×	×	geraniol synthase
Opuchr08_g0009750-1.1	OG0000007	40	13	26	24	×	×	×	tabersonine 3-oxygenase
Opuchr08_g0009760-1.1						×	×	×	tabersonine 3-oxygenase
Opuchr08_g0009790-1.1	OG0000007	40	13	26	24	×	×	×	tabersonine 3-oxygenase
Opuchr08_g0009840-1.1	OG0017320	1	0	0	0	×	×	×	unnamed protein product
Opuchr08_g0009850-1.1	OG0000007	40	13	26	24	×	×	×	Tabersonine 3-oxygenase
Opuchr08_g0009860-1.1	OG0000007	40	13	26	24	×	×	×	tabersonine 3-oxygenase

1 **Number of genes assigned to an orthogroup from a specific species**

2 **Presence or absence of genes from a syteny relationships with *O. pumila*** { • Present × Absent }

Supplementary Fig. 47. Monoterpene indole alkaloid (MIA) gene-clusters identified in chromosome 8 of the *Ophiorrhiza* genome assembly. The position of the gene-cluster is scaled based on chromosome size and its physical position on the chromosome. The gene description, colored as orange, represents genes with functions associated with MIA biosynthesis. O.p: *Ophiorrhiza pumila*; C.a: *Camptotheca acuminata*; C.r: *Catharanthus roseus*; G.s: *Gelsemium sempervirens*.

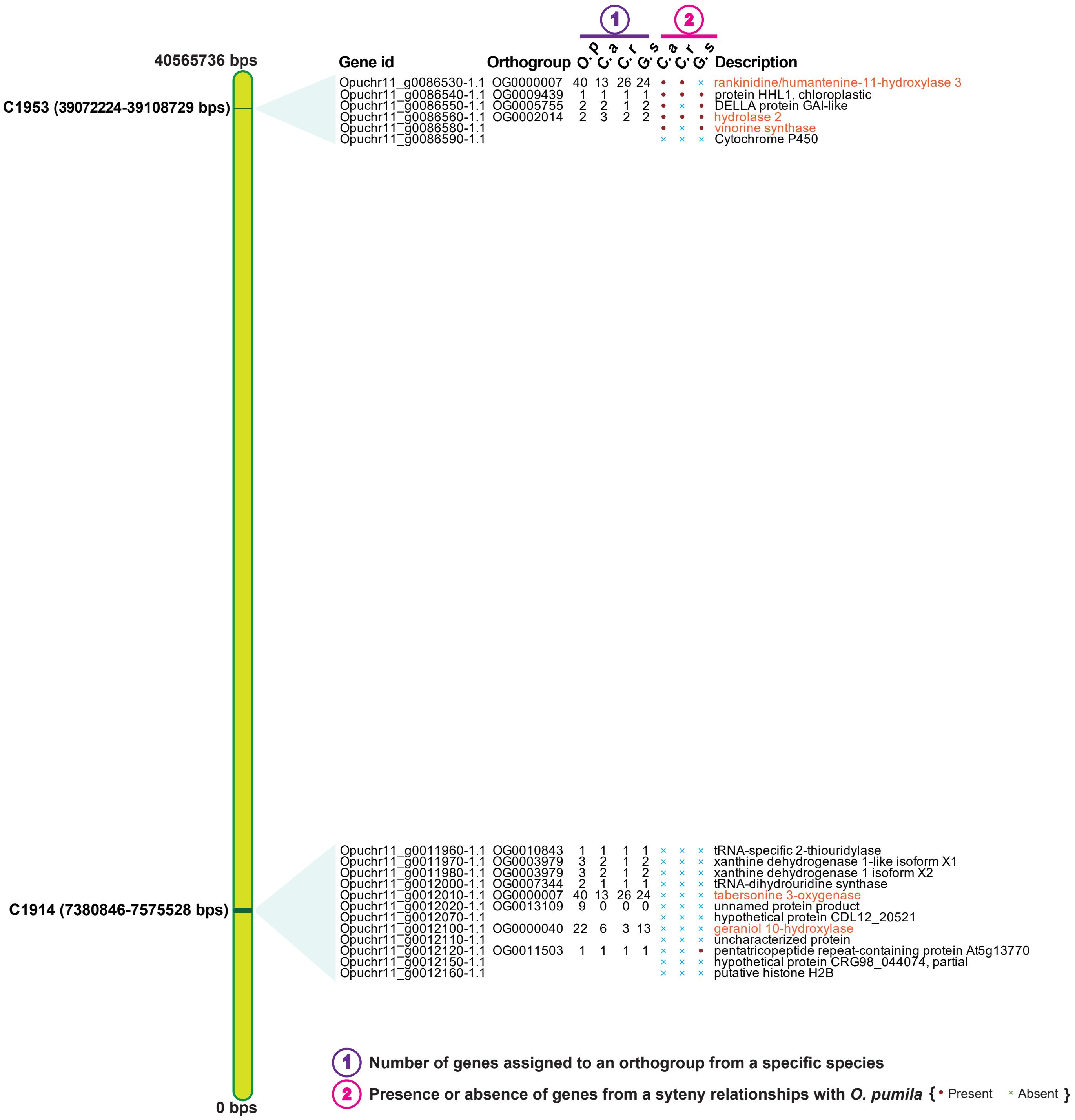

Supplementary Fig. 48. Monoterpene indole alkaloid (MIA) gene-clusters identified in chromosome 11 of the *Ophiorrhiza* genome assembly. The position of the gene-cluster is scaled based on chromosome size and its physical position on the chromosome. The gene description colored as orange represents genes with functions associated with MIA biosynthesis. O.p: *Ophiorrhiza pumila*; C.a: *Camptotheca acuminata*; C.r: *Catharanthus roseus*; G.s: *Gelsemium sempervirens*.
