# Supplementary material for: Chromosome-level genome assembly of Ophiorrhiza pumila reveals the evolution of camptothecin biosynthesis
Source: Nat Commun. 2021 Jan 15;12:405. doi: 10.1038/s41467-020-20508-2 (PMC7810986; doi:10.1038/s41467-020-20508-2)
Supplement: Supplementary file 3 — Reporting Summary [file 41467_2020_20508_MOESM3_ESM.pdf]

nature research

Corresponding author(s): Mami Yamazaki, Amit Rai

Last updated by author(s): Nov 25, 2020

Reporting Summary

Nature Research wishes to improve the reproducibility of the work that we publish. This form provides structure for consistency and transparency in reporting. For further information on Nature Research policies, see Authors & Referees and the Editorial Policy Checklist .

Statistics

For all statistical analyses, confirm that the following items are present in the figure legend, table legend, main text, or Methods section.

n/a	Confirmed	
☐	☒	The exact sample size (*n*) for each experimental group/condition, given as a discrete number and unit of measurement
☐	☒	A statement on whether measurements were taken from distinct samples or whether the same sample was measured repeatedly
☐	☒	The statistical test(s) used AND whether they are one- or two-sided<br>*Only common tests should be described solely by name; describe more complex techniques in the Methods section.*
☒	☐	A description of all covariates tested
☐	☒	A description of any assumptions or corrections, such as tests of normality and adjustment for multiple comparisons
☐	☒	A full description of the statistical parameters including central tendency (e.g. means) or other basic estimates (e.g. regression coefficient) AND variation (e.g. standard deviation) or associated estimates of uncertainty (e.g. confidence intervals)
☐	☒	For null hypothesis testing, the test statistic (e.g. *F*, *t*, *r*) with confidence intervals, effect sizes, degrees of freedom and *P* value noted<br>*Give P values as exact values whenever suitable.*
☐	☒	For Bayesian analysis, information on the choice of priors and Markov chain Monte Carlo settings
☒	☐	For hierarchical and complex designs, identification of the appropriate level for tests and full reporting of outcomes
☐	☒	Estimates of effect sizes (e.g. Cohen's *d*, Pearson's *r*), indicating how they were calculated

Our web collection on statistics for biologists contains articles on many of the points above.

Software and code

Policy information about availability of computer code

Data collection

Please see methods for a full description of analysis tools used. Most are open source standard bio-informatics tools, with custom R scripts used for data analysis. For data acquisition, the software provided by the specific instruments were used. Softwares for metabolite-profiling data processing and analysis, sequencing data processing and analysis, and other tools are mentioned in the method section.

Data analysis

Tools used in this article (further details about command lines are found in Methods and/or Supplementary Information):
- Canu v 1.7
- FALCON-unzip v 1.3
- FALCON-phase software (DOI: 10.1101/327064)
- BioNano Solve v 3.0.1
- PBJelly from PBSuite v 15.8.24
- BWA (v 0.7.16)
- Arrow software (https://github.com/PacificBiosciences/GenomicConsensus/tree/develop/GenomicConsensus/arrow)
- Pilon software (version 1.18) (DOI: 10.1371/journal.pone.0112963)
- IrysView software (BioNano genomics)
- Juicerbox software (DOI: 10.1016/j.cels.2016.07.002)
- BioNanoAnalyst (DOI: 10.1186/s12859-017-1735-4)
- KmerGenie software (DOI: 10.1093/bioinformatics/btt310)
- BioNano Solve software (v 3.0.1)
- Trinity (v 2.6.6)
- OmicsBox software v 1.3.10 (BioBam)
- HTSeq software (v 0.11.1)
- PlantClusterFinder (v 1.3)

- PathoLogic software (v 22.5)
- SAVI software (v 3.0.2)
- MAKER-P pipeline (v 2.31.8)
- Augustus (v3.3)
- SNAP (v2006-07-28)
- GeneMark_ES (v4.33)
- TopHat (v2.1.1)
- Cufflink (v2.2.1)
- GyDB 2.0
- BLASTp (v 2.7.1+)
- Tandem Repeats Finder (DOI: 10.1093/nar/27.2.573)
- INFERNAL Software (DOI: 10.1093/bioinformatics/btt509)
- LAST (v963)
- MCScanx software (DOI:10.1093/nar/gkr1293)
- PAML package (DOI: 10.1093/molbev/msm088)
- MUSCLE (v 3.8.31)
- OrthoFinder (v 2.3.1)
- COUNT software (DOI: 10.1093/bioinformatics/btq315)
- BLAT (DOI: 10.1101/gr.229202)
- BUSCO (DOI: 10.1093/molbev/msx319)
- R package
- BLASTn (v 2.7.1+)
- RepeatMasker (http://www.repeatmasker.org/RMDownload.html)
- RepeatModeler v1.0.11
- BRAKER v2
- InterProScan (V4.8)(DOI: 10.1093/bioinformatics/btu031)
- tRNAscan-SE software (DOI: 10.1007/978-1-4939-9173-0_1)
- Trimmomatic v0.36
- BowTie 2.0
- CD-HIT-EST (DOI: 10.1093/bioinformatics/bts565)
- MAFFT (DOI: 10.1093/bioinformatics/bty121)
- RaxML (v 8.2.11)
- E2P2 software (v 3.1)
- MS-FINDER (v3.0) (http://prime.psc.riken.jp/compms/msfinder/main.html)
- MS-DIAL (v 3.0) (http://prime.psc.riken.jp/compms/msdial/main.html)
- MCMCtree program (http://abacus.gene.ucl.ac.uk/software/paml.html),
- heatmap 2.0 from gplots v3.1.0 (https://www.rdocumentation.org/packages/gplots/versions/3.1.0/topics/heatmap.2)
- Cytoscape software (version 3.6.1)
- BUSCO (v3.0.3)
- SAMtools (version 1.3)
- htslib (version 1.3)
- InterPro (V32.0)
- Pfam (v27.0)
- Juicebox Assembly Tool (v1.9.1)
- Kmergenie (v 1.7048)
- BD FACS DIVA software (v 7.0).

For manuscripts utilizing custom algorithms or software that are central to the research but not yet described in published literature, software must be made available to editors/reviewers. We strongly encourage code deposition in a community repository (e.g. GitHub). See the Nature Research guidelines for submitting code & software for further information.

Data

Policy information about availability of data

All manuscripts must include a data availability statement. This statement should provide the following information, where applicable:

- Accession codes, unique identifiers, or web links for publicly available datasets
- A list of figures that have associated raw data
- A description of any restrictions on data availability

Accessions for genome assemblies used in Supplementary Fig. 2 to compare assembly stats with Ophiorrhiza pumila is provided in Supplementary Table 5. The metabolome datasets for 12 plant species using carbon labeling approach were obtained from http://prime.psc.riken.jp/. The DOI in Metabolomics Workbench is https://doi.org/10.21228/M8XM40. For transcriptome analysis, apart from newly generated datasets, we also used previously published RNA-seq datasets from DDBJ Sequence Read Archive (accession No. DRA000930.). Source data for creating Fig 4 is available as Source Data file, as well as by using this link-https://github.com/amit4mchiba/Codes-and-script-for-Ophiorrhiza-pumila-genome-manuscript/tree/main/Circos-plot-for-12-plant-metabolome-analysis

All raw data and assembled sequence data have been deposited at the DDBJ database under the following annotations:
Submission id- DRA009076;
BioProject id- PRJDB8685;
BioSample id- SAMD00177989;
Experiment- DRX185163-DRX185191;

Run: DRR194711-DRR194739.

Assembled O. pumila genome assembly has been deposited at the DDBJ database (accession ids- BLIW01000001- BLIW01000013).
Plant material used in this study including hairy roots are maintained at Chiba University, Japan, and will be available for experimental purposes, subject to a material transfer agreement. All sequence datasets, assembled genome, transcriptome datasets, genome browser, annotation, and KEGG mapping results are available through a dedicated server to perform comparative genome analysis (http://pumila.kazusa.or.jp/). All analysis results, including transcriptome analysis, metabolome analysis, identified metabolic gene-clusters, orthogene information, and results from comparative genome analysis are available in the form of supplementary tables and datasets.

Field-specific reporting

Please select the one below that is the best fit for your research. If you are not sure, read the appropriate sections before making your selection.

☒ Life sciences ☐ Behavioural & social sciences ☐ Ecological, evolutionary & environmental sciences

For a reference copy of the document with all sections, see nature.com/documents/nr-reporting-summary-flat.pdf

Life sciences study design

All studies must disclose on these points even when the disclosure is negative.

Sample size	For whole genome sequencing, we used young leaves of Ophiorrhiza pumila, and extracted genomic DNA for different sequencing platforms. For transcriptome profiling, we used five tissues, and also used previously published RNA-seq datasets for hairy roots and cell suspension culture of Ophiorrhiza pumila. For metabolite profiling, same tissues were selected. The choice of tissues/samples selected for this study were based on our previous knowledge on camptothecin accumulation across tissues of O. pumila. We identified and selected tissues based on different levels of camptothecin accumulation, the key medicinal compound that we were exploring in O. pumila. The objective was the include tissues that are high (hairy root, root and inter-node) or low accumulation (leaf) of camptothecin for the analysis, and to capture key features associated with MIAs biosynthesis. For complete nitrogen labeling, we used second generation hairy roots maintained at growth media as control sample, and second generation hairy roots maintained at growth media replaced with N15 labeled chemical constituents as completely N15-labeled samples.
Data exclusions	We only excluded sequences that were of low quality for genome assembly.
Replication	For metabolome analysis for multiple tissues of O. pumila, we used 5 biological replicates per each tissue used for metabolite profiling. For complete nitrogen label metabolome analysis, we used five replicates for each condition. All attempts at replication were successful for metabolome analysis as suggested by the unsupervised Principle component analysis. For all FISH experiments, we performed experiment twice, and for each experiment more than 10 cells/slides were visualized to confirm the signal from each of the probes analyzed. For all sequencing analysis and sampling, no replication was performed that was not documented in the paper.
Randomization	For metabolite analysis, plants from each of the experimental groups were selected randomly, and extracted metabolite samples for profiling were randomized before data acquisition. Samples were assigned to accessions, and were randomized before injection to the mass-spectrometer. All biomolecule extraction, including metabolites and RNAs for all samples were performed on the same day to avoid any batch effect, and experiments were performed by the same individual. Plants grown for transcriptome, Hi-C, and shotgun sequencing were randomly selected.
Blinding	The team that worked for sample preparation and the team that performed data acquisition for all analysis (Metabolome, transcriptome, genome sequencing) worked independently, and team performing profiling were provided with the accession numbers for the samples. The complete information related to samples, treatments, tissues types and replications were known to the team that performed analysis and data interpretation. For sequencing, we relied upon service providers or collaborators, while metabolite profiling was performed at CSRS, RIKEN.

Reporting for specific materials, systems and methods

We require information from authors about some types of materials, experimental systems and methods used in many studies. Here, indicate whether each material, system or method listed is relevant to your study. If you are not sure if a list item applies to your research, read the appropriate section before selecting a response.

Materials & experimental systems

n/a	Involved in the study
☒	☐ Antibodies
☒	☐ Eukaryotic cell lines
☒	☐ Palaeontology
☒	☐ Animals and other organisms
☒	☐ Human research participants
☒	☐ Clinical data

Methods

n/a	Involved in the study
☒	☐ ChIP-seq
☐	☒ Flow cytometry
☒	☐ MRI-based neuroimaging

Flow Cytometry

Plots

Confirm that:

☒ The axis labels state the marker and fluorochrome used (e.g. CD4-FITC).

☒ The axis scales are clearly visible. Include numbers along axes only for bottom left plot of group (a 'group' is an analysis of identical markers).

☒ All plots are contour plots with outliers or pseudocolor plots.

☒ A numerical value for number of cells or percentage (with statistics) is provided.

Methodology

Sample preparation	Young leaves of O. pumila and A. thaliana were cut using a razor blade in the ice-cold 2-amino-2-(hydroxymethyl)-1,3 propanediol (TRIS)-MgCl2 buffer (0.2 M TRIS-HCl, 4 mM MgCl2, 0.5% Triton-X 100, pH 7.5) including propidium iodide (50 µg ml-1) and ribonuclease (50 µg ml-1), and incubated for 5 minutes. The relative DNA content of isolated nuclei was analyzed using a flow cytometer.
Instrument	FACSCalibur system (Becton Dickinson, New Jersey, USA)
Software	BD FACS DIVA software
Cell population abundance	No post-fractions were collected.
Gating strategy	The preliminary FSC/SSC gates of the starting cell population were not used.

☐ Tick this box to confirm that a figure exemplifying the gating strategy is provided in the Supplementary Information.
