# Supplementary material for: Chromosome-level genome assembly of Ophiorrhiza pumila reveals the evolution of camptothecin biosynthesis
Source: Nat Commun. 2021 Jan 15;12:405. doi: 10.1038/s41467-020-20508-2 (PMC7810986; doi:10.1038/s41467-020-20508-2)
Supplement: Supplementary file 4 — Description of Additional Supplementary Files [file 41467_2020_20508_MOESM4_ESM.pdf]

Description of Additional Supplementary Files

Supplementary Data 1 Raw sequencing reads from different next-generation sequencing platforms used to derive *de novo* genome assembly.

Supplementary Data 2 Parameters optimization and resulting assembly statistics for contig-level genome assembly using Canu (Selected assemblies are colored as orange).

Supplementary Data 3 Parameters optimization and resulting assembly statistics for contig-level genome assembly using Falcon-unzip assembler (Selected assemblies are colored as orange).

Supplementary Data 4 *Ophiorrhiza pumila* phased genome assembly statistics at different stages and combinations of scaffolding.

Supplementary Data 5 List of primers used to perform Fluorescence in situ hybridization (FISH) analysis.

Supplementary Data 6 Assembly statistics and metadata for published and publicly available chromosome scale plant genomes used to compare with *Ophiorrhiza pumila* reference genome assembly.

Supplementary Data 7 *Ophiorrhiza pumila* genes annotated as transcription factors.

Supplementary Data 8 Prediction and annotation of non- coding RNAs in the *Ophiorrhiza pumila* reference genome.

Supplementary Data 9 List of tRNA predicted in *Ophiorrhiza pumila* genome using tRNAScan tool.

Supplementary Data 10 Summary of non-coding RNAs predicted in the *Ophiorrhiza pumila* reference genome.

Supplementary Data 11 Predicted telomere regions and associated repeats of *Ophiorrhiza pumila* genome assembly.

Supplementary Data 12 Transposable elements predicted and compared for eight plant genomes.

Supplementary Data 13 Composition of growth media used for ^{15}N stable isotope labeling of *Ophiorrhiza pumila* hairy root metabolome.

Supplementary Data 14 Nitrogen-containing metabolites identified in *Ophiorrhiza pumila* metabolome with assigned chemical annotation.

Supplementary Data 15 Accumulation levels of metabolites identified in this study across six tissues of *Ophiorrhiza pumila.*

Supplementary Data 16 Manually curated database for functionally characterized enzymes associated with monoterpene indole alkaloids biosynthesis used for homology-based annotation *Ophiorrhiza pumila* gene models.

Supplementary Data 17 Annotation of *Ophiorrhiza pumila* gene models using Blast search based on monoterpene indole alkaloids biosynthesis enzyme database curated in this study.

Supplementary Data 18 *Ophiorrhiza pumila* genes assigned to monoterpene indole alkaloids (MIAs) biosynthesis pathway based on sequence homology with functionally characterized genes and used as criteria to select MIA gene-cluster.

Supplementary Data 19 Metabolite-Genes relationships based on Pearson correlation coefficient cutoff as 0.7 and *p*-value cutoff as 0.05.

Supplementary Data 20 OrthoFinder based gene classification using *Ophiorrhiza pumila* and 12 other plant genomes.

Supplementary Data 21 Posterior rate for gain, loss, expansion, and contraction calculated for the gene families.

Supplementary Data 22 Synonymous substitution rate (Ks)-median for orthogroups assigned to genes associated with secoiridoid and MIA biosynthesis pathways.

Supplementary Data 23 Metabolite gene-clusters with associated biochemical reactions.

Supplementary Data 24 Metabolite gene-clusters identified in the *Ophiorrhiza pumila* genome.

Supplementary Data 25 Synonymous substitution rate (Ks) for gene pairs associated with syntenic relationship between *Ophiorrhiza pumila* and four plant species, namely, *Coffea canephora, Catharanthus roseus, Gelsemium sempervirens,* and *Camptotheca acuminata.*

Supplementary Data 26 Synonymous substitution rate (Ks) for genes assigned to *Ophiorrhiza pumila* metabolite gene-clusters and associated with syntenic relationship between *O. pumila* and three other plant species, namely, *Catharanthus roseus, Gelsemium sempervirens,* and *Camptotheca acuminata.*
