# Supplementary material for: Chromosome-level genome assembly of Ophiorrhiza pumila reveals the evolution of camptothecin biosynthesis
Source: Nat Commun. 2021 Jan 15;12:405. doi: 10.1038/s41467-020-20508-2 (PMC7810986; doi:10.1038/s41467-020-20508-2)
Supplement: Supplementary file 2 — Peer Review File [file 41467_2020_20508_MOESM2_ESM.pdf]

Reviewer #1 (Remarks to the Author):

This manuscript reports an enormous amount of works that allowed the authors to establish a new plant genome with assembly parameters of very high quality and with experimental validations by chromosome FISH, to depict the genome characteristics in detail, to comprehensively describe the metabolic diversity of the plant as well as a gene-to-metabolite network, and to propose important hypothesis on the evolution of genes involved in the biosynthesis of one of most diversified class of secondary metabolites, monoterpene indole alkaloids (MIAs). Plants producing MIAs provide several pharmacologically active molecules and anti-cancer drugs, such as camptothecin, vinblastine, vincristine. Deciphering the genetic and evolutionary mechanisms at the origin of the unique metabolic diversity of land plants is one of the major challenge in plant biology, and this work provides important resources towards this quest.

Major claims of the paper and critical assessment:

A near complete genome of the camptothecin producing medicinal plant Ophiorrhiza pumila, with very few gaps : 21 remaining assembly gaps over 11 chromosomes ! Most plant genome assemblies have from several hundreds to several thousands gaps. Although O. pumila genome is a typical plant genome with relatively high level of repetitive sequence, the authors combined four complementary next-generation sequencing technologies with an ordered multi-scaffolding approach and experimental validation of each scaffold at the assembly gaps by chromosome FISH to achieve a near complete reference plant genome assembly of O. pumila. In addition to the reference genome, two haplotype-resolved genome assemblies of O. pumila were generated with assembly parameters of very high quality, although with slightly more gaps than the reference genome. This report is of high interest for anyone in the field of plant genomic since it is one of the best plant genome assembly described (as shown in Supplementary figure 1 that compares the status of several plant genome assemblies). One important lesson from this work, based on detection of orientational error after bioinformatics-based assembly of O. pumila genome, is the needs for experimental validation of scaffolding models. That other plant genomes might needs similar validations was shown by the authors. The observation of unusual synteny relationships between published Coffea canephora genome and O. pumila genome, prompt the author to verify by FISH the coffee chromosome inconsistent segments, and substantiate a misassembly in the chromosome 2 of the coffee genome. Therefore, O. pumila genome assembly will likely provide a resource to improve plant genome assembly and a model to understand the evolution of plant genome.

Another major claim of this work is a comprehensive description of nitrogen-containing metabolites in O. pumila. The nitrogen-oriented metabolome was achieved through an original methodology, previously reported by the group (Nature methods 2019, Analytical Chemistry 2020). It includes complete 15N stable-isotope labelling and chemoinformatics approach. 273 nitrogen-containing metabolites were assigned, mostly to indole alkaloids, MIAs, and carboline moieties containing metabolites. Tissue specific accumulation patterns were also described, and in combination with a transcriptomic profiling, a gene-to-metabolite network associated to MIA biosynthesis was identified that provide candidate gene for identification of the biosynthetic pathway molecular determinants. Candidate gene for biosynthesis of the central precursor for MIAs, strictosidine, were convincingly identified, based on gene homology to model plant species for MIA biosynthesis, like Catharanthus roseus, however recognition of downstream genes towards O. pumila specific pathway branch will require further works.

Most originally, interesting hypothesis for evolution of MIA biosynthesis in plants were elaborated. Ks plots analysis over selected plant genomes (within asterids) including three MIA-producing plant provided basis for characterization of whole genome evolution. A recent whole genome duplication was detected in the camptothecin –producing Camptotheca acuminata (Cornales), but not in O. pumila (Rubiaceae, Gentianales) neither in C. roseus (Apocynaceae, Gentianales). Previous work showed that MIA biosynthesis had acquired a different path in Cornales than in Gentianales as evidenced by the

production of strictosidinic acid derivative in C. acuminata (Cornales) instead of strictosidine in Gentianales. This work now point out to specific evolution of MIA in Cornales with respect to lack of Strictosidine synthase orthogroup and presence of alternative genes to secologanin synthase. Interestingly analysis of evolution of gene families involved in MIA biosynthesis showed faster evolution in MIA producing plants than in non-MIA producing plants, except, remarkably for tryptophan decarboxylase, an enzyme with potential broader functions in plant metabolism. Among those faster evolving gene families, SLS and STR were claimed to show positive selection.

Finally, the authors claims a role for secondary metabolite gene clusters in evolution of MIA biosynthesis. Historically, metabolic gene clusters were considered a characteristic of prokaryotes. Most genes of prokaryotic genomes are organized in clusters of coregulated genes with related functions. However, an increasing number of genes associated to plant secondary metabolic pathways have been reported in gene cluster over the year. The iconic examples in plant secondary metabolism is the 10 gene cluster encoding all enzymes specific to noscapine pathway in opium poppy (Science [2012] 336, 1704), but smaller gene clusters, with a minimum of three and usually more genes, were also identified. The genetic process at the origin of metabolic gene clusters are not clearly understood but do not depends strictly on the widespread process of local gene duplication and divergence producing tandem array of homologous genes. Well-characterized metabolic gene clusters in plants involve a number of functionally related genes (e.g. involved in a common metabolic pathway), and most of theses genes are non-homologous (The Plant Journal [2011] 66, 66-79). In my point of view, cluster of genes simply based on potential function in secondary metabolism do not provide a strong evidence for metabolic gene cluster. Evidence for functional link shared by a significant number of genes in the cluster should be search for. Some gene clusters described in this work appears to show functional link, for instance cluster C1541. However, a number of gene cluster may also not have the same level of functional signification. In my point of view, it would be important to further characterize the signification of gene clusters highlighted in O. pumila.

Detailed comments.
Major revisions :

-Critical review of the manuscript on the gene clusters: One of the criteria to identify functional gene cluster is the correlation of expression of the gene members. With respect to O. pumila candidate gene clusters, does the clustered genes show co-expression? PhytoClust (Nucleic Acids Res. 2017, 45:7049–63) provide analysis tool for co-expression analysis. In addition, do they belong to gene-to-metabolite network described in the supplementary figure 27?

-page 20, line 16 : “The entire secoiridoid biosynthetic pathways and MIAs biosynthesis-associated genes from the Ophiorrhiza genome were members of gene cluster”. What is the signification of this observation if the pathway associated genes are spreads among several different clusters ? From figure S26, I could count 40 different gene clusters (C1318, C1320, C1321, C1327, C1385, C1401, C1418, C1423, C1444, C1445, C1453, C1454, C1493, C1497 ,C1501, C1504, C1527, C1532, C1537, C1538, C1572, C1592, C1624, C1635, C1643, C1746, C1747, C1748, C1749, C1752, C1810, C1824, C1914, C1953, C1381, C1541, C1559, C1565, C1684, C1693; why is this not in agreement with “33 gene clusters” in page 20, line 10) , with 6 of them (C1381, C1541, C1559, C1565, C1684, C1693) having two different OG class genes and none more than two. Some gene cluster have additional OG not shown in figure S26, that may add to this counting but if they are not shown, does that mean they are not expressed and therefore unlikely to play a function in MIA biosynthesis ?

On the other hand, did the authors considered the possibility of metabolic gene clustering at a supra-chromosomal level in the chromatin ? Does data from Hi-C contact map might be used to identify such potential gene clustering ?

Minor revisions :

Figures
-Although most figures in this manuscript are of very high quality, some data cannot be easily interpreted by non-specialist without some more information in legend. I have pinpoint most of these below.

Figure 1b : centromere positions are shown by a dotted lines which point to the pachytene chromosome picture but also cross the assembled chromosome double line at positions that do not seem to correspond to centromere positions. For instance, I presumed gap 1 in chromosome 1 is in the centromeric region. Gap 1 is far away from position of assembled chromosome crossed by the dotted line.

Figure 3e, the signification of the arrows are not mentioned in the legend.

Figure 4. I do not understand the rationale for some of the metabolite ordering. For instance, deoxyloganic acid and loganin are grouped away from secologanin.

Figure 6 title should be reconsider with respect to my comment on convergent evolution below. Figure 6a does the legend for colour shading is related to the shading at the top right of the panel ? This top right part of the figure should be better explained in the legend. Figure 6b and d, please provide more information about busted analysis in legend. Figure 6e, what is the signification of red highlighted orthogene ?

Supplementary figure 1 should also highlight the status of other genome assembly from MIA-producing species. In addition, the authors should consider the possibility to include throughout their work some comparisons with the genome of the Apocynaceae Rhazya stricta published in 2016 .

Supplementary Fig. 2 : please provide more information on colour bars signification. In addition to green, what means the light blue (turquoise) and dark blue bar, as well as the yellow colour and alignment shading signification. Since green bars are multicolour, it is not clear what part is from PacBio reads. Does the bar labeled Canu_contig represents PacBio reads ?

Supplementary Fig. 6. Chromosome 3 was not identified by FISH probe (no red mark on ideogram of Chr 3) according to Figure S6c. Is that correct ?

Supplementary Fig. 7. I do not understand what is shown in this figure. Which line is the reference genome, where are the wedges. Is the level of resolution sufficient to view the wedges ?

Supplementary figure 8: what is the signification of the green pixels ?

Supplementary figure 15. what are the specific legends for figure 15 a to f ?

Supplementary figure 17. Indicate in legend the meaning of numbers in the phylogenetic tree (divergence time and branch length in Mya ago ?). Edit species name typos : sempervirens and benthamiana

Supplementary figure 26. The signification of the vertical blue lines is not obvious, explain in legend or use a more recognisable graphic legend.

Supplementary figure 30. This figure shows 4 genes from chr11 and 2 from chr5 for LAMT OG0000252 and 5 genes in OG0014261. However the figure 6A only show one gene (if I understand properly the colour shading) for LAMT OG0000252. Is this correct in figure 6A. Double check for potential similar mistakes.

Supplementary figure 31. “Results suggested evolution of STR as key event that preceded with evolution of genes associated with MIA biosynthesis.” It may not be obvious for non specialist how this conclusion is drawn from this figure data ? Explain more clearly in the text. Or provide additional hints in the figure.

Supplementary figure 32. Some MIA gene clusters (e.g. C1321, C1327) present on Chr1 are not shown on figure 32a. Please show and check for other missing MIA gene clusters on the other chromosomes. Please add Chr 3, 9 and 10 with their MIA gene clusters.

Content and ideas :

-Throughout the manuscript, I suggest to provide additional informations for non-specialist to better understand the importance of the achievements in this report. Since this work has the potential to attract a broad audience, more guidance should be provided to the reader to better understand the methodological approach and how to interpret the results. For instance, a few words on the characteristics and advantage of some of the newer NGS technology (Bionano, HiC) and assembly methodologies would help. How to compare Ks value and to interpret Ks plots may not be simple for non-specialist. What is the interest of using 15N labelling for metabolic analysis?

-page 4, line 22: “A combination of the comparative genomics approach revealed the role of strictosidine biogenesis towards orchestrated evolution of down-stream enzymes of MIA biosynthesis pathways”. This strong statement claims major evidence for orchestrated evolution. What data show this? Unless further evidences are provided, I suggest rephrasing with wordings that are more careful.

-page 19, line 5: “suggesting convergent evolution of”. Convergent evolution imply independent evolution in different species of a character that was not present in their last common ancestor. Can you rule out that SLS and STR were not primitive characters in the MIA-producing plants ?

-page 20, line 8: “STR lost within the coffee genome at gene cluster may have limited the opportunity to direct evolution towards MIA biosynthesis, which also explains higher Ks-median for enzymes associated with MIA biosynthesis”. Can the authors exclude an alternative interpretation. MIA biosynthesis had evolved in Rubiaceae before divergence of Coffee. After Coffee divergence, lost of STR stopped positive selection on these genes.

-page 22, line 2. I disagree with the first part of the sentence. The pathway for catharanthine and vindoline are fully identified in C. roseus and may be one or two steps are missing to go from these MIAs to vinblastine. However, I agree that for camptothecin pathway elucidation and study of MIA pathway evolution, O. pumila genome will be an invaluable tool.

Word choice, grammar, typos, etc.
Page 6, line 7: “The entire genome consist of just…” this sentence is supported by figure 1B. Please refer to.

Page 10, line 17: “Parallel evolution of MIA biosynthesis in plants” this subtitle precedes a long description of O. pumila genome content, the authors should consider adding a dedicated subtitle for this part and shifting this one below.

Page 13, line 10: “secondary metabolite gene clusters…. Reference 2, 31 , 33.” Gene cluster in some part of this manuscript either refer to hierarchical gene cluster of expression (Suppl. figure 23) or to gene cluster on chromosome. It is not clear what type of cluster is considered here since for instance

reference 33 describes hierarchical gene cluster of expression. Please use an alternative expression for gene cluster when dealing with expression cluster to avoid confusion. Reference 31 does not seem to refer to gene clusters…Some comprehensive review on metabolite gene clusters should be cited.

Page 23, line 19. F.K. is not in the author list, and R.K. is not in author contributions. Does F.K. should be R.K. ?

Page 31, line 12. Correct the number of molecules. Line 15 : bp. Line 21 : 42X in table S1. What is the correct fold ?

Page 51, line 15 : acuminata (while I was writing this, my text editor did an improper correction into acuminate !), line 18 : acuminata.

Page 58, line 8 : supplementary. Line 10 : again here, gene cluster refer to a different definition than with metabolic gene cluster elsewhere in the paper. I suggest using a different wording to avoid confusion.

Reviewed by Pr Benoit St-Pierre

Reviewer #2 (Remarks to the Author):

The authors have analysed and annotated the genome of Ophiorrhiza pumila,a plant which produces the antitumor alkaloid camptothecin. A special emphasis was laid on the genes involved in camptothecin biosynthesis as compared to the biosynthesis of othe monoterpene alkaloids.
This is a very comprehensive analysis covering many areas and topics. I really appreciate that the authors publish 1 big paper instead of several smal ones.
The methodoly is excellent, the bioinformatics adequate and the ms is well written. Figures are of good quality (except the foto of the plant, which looks a bit out of focus in my copy).
There are afew typos in species names, e.g. not Coffee but Coffea.
The authors discuss the origin of the genes of alkaloid formation and have interesting conclusion.
I published a review in the pregenomic time, which might still be of interest

Wink, M.: Evolution of secondary metabolites from an ecological and molecular phylogenetic perspec-tive. Phytochemistry 64, 3-19, 2003
Wink, M. F. Botschen, C. Gosmann, H. Schäfer and P. G. Waterman: Chemotaxonomy seen from a phylogenetic perspective and evolution of secondary metabolism. In Wink, M. (Ed.); Biochemistry of plant secondary metabolism, Blackwell, Annual Plant Reviews Vol. 40, 2nd ed., 2010

Reviewer #3 (Remarks to the Author):

The authors of the article "Multi-scaffolding driven chromosome-level Ophiorrhiza genome revealed gene cluster centered evolution of camptothecin biosynthesis" provide a very accurately assembled de novo genome of a very interesting model plant species. The applied sequencing and assembling strategy are thorough and very well and detailed described. However, the article is weak when it comes to monoterpene indole alkaloid (MIA) biosynthesis part. It is indicative, that neither a single chemical structure of an MIA nor the current knowledge of their biosynthesis is illustrated in the introduction. In the results part, the authors introduce the names of biosynthetic enzymes, which is way too late.
It is also not clear in the introduction, what the authors consider as "camptothecin biosynthesis". Is it the biosynthesis beginning with the universal precursor of MIAs, strictosidine – or are earlier steps leading to strictosidine included. If so, the current state of knowledge should be given in the introduction. This will also help the reader to follow the authors strategy to identify MIA biosynthetic

genes, especially, as in the methods part, the authors state, that 94 genes (suppl. table 16) that have been functionally characterized to be associated with MIA biosynthesis, were manually curated in their dataset. T

Furthermore, MIAs are found in many diverse species from different plant families. As the authors state that they studied the evolution of MIAs, they should propose and discuss some evolutionary theories – e.g. did MIAs evolve in parallel in the different plant families and how is the organization in gene clusters involved? Further – as MIAs are secondary metabolites, they should also discuss the evolutionary pattern of other secondary metabolites in the discussion part, to put their own research in a broader frame. I think this would strongly improve the manuscript which is at this point, a very detailed explanation of a de novo genome sequencing.

Another very general remark: the manuscript is way too long and it could be easily shortened if the authors would strictly describe the methods and exclude any results from the methods-chapter of their manuscript. They not only repeatedly present results in the methods part (which introduces a lot of redundancy to their manuscript), they also discuss their results in the results part – my advice: either have a combined results & discussion part, or really separate them.

I believe the data generated in this work is very valuable and interesting to the scientific community, but the writing of the manuscript has to be strongly improved.

Some specific major and minor comments:

Check Latin names of the species, sometimes “Coffee canephora” is written.

Page 3:

Line 4: vincristine is an original plant MIA, produced by Catharanthus roseus. The text suggest that it is derived from plant origin.

Line 13: when O. pumila can serve as a toolkit to understand MIAs (plural) biosynthesis – how many different MIAs can be detected in O. pumila?

Line 22: its MIA biosynthesis, and – does Coffea canephora produce the universal MIA precursor strictosidine? Or is this pathway completely absent in C. canephora?

Page 5:

Line 9: as the authors mention that polyploidy makes de novo genome sequencing challenging –what is the ploidy status of O. pumila?

Page 10:

The title “Parallel evolution of MIA biosynthesis”: check the title, it does not fit -- in the first part of this chapter, repeats are described, and gene models, parameters for the quality of the genome – but nothing is said about the MIA biosynthesis.

Line 22: it was already described in detail, that chromosome 2 had to be rearranged….

Page 12:

Line 2: to test for a "recent" WGD, only paralogs of O. pumila are of interest, in my understanding. Orthologs give information on speciation. Linked to this – in Fig. 3e, two arrows are shown, the one indicating the newly identified WGD in C. acuminata, and the other??

Line 6: only here MIA biosynthesis “starts” – but the things that are described here, should be stated in the introduction and citations should be included. E.g. “whole genome duplications and transposable elements are regarded as key mechanisms for the evolution of novel features in plants” – first, I miss the citation, and second, this statement is definitely no result…

Line 11: Why does the differential repeat profiles and the independent WGD in C. acuminate suggest an independent evolution of MIA biosynthesis? Where is the connection between repeat profiles and MIA biosynthesis? A WGD itself also does not per se effect a biosynthetic trait, if the trait was present before the WGD, it will be present thereafter. o test evolutionary scenarios, a trait has to be linked to the phylogeny of the species in which this trait occurs. One can do a character state reconstruction, for example. In case of MIAs, they occur in distantly related species and either evolved independently, or evolved very early and were subsequently lost repeatedly.

Line 17: What does the author mean by the term “active evolution”? And – how does this title relate to the chapter?

Line 21: the authors state, that 13C based metabolomes exist for 12 plant species, on the next page (line 3) the authors mention “metabolome space for previously analyzed 11 plant species”. 12 or

11??!
Figure 4: There is a legend, that assigns a color code to specific metabolite classes (Indole, Anthraquinones…), but there is also a color code in the circle plot – specific for species. This makes no sense to me. How can a slice of the circle plot represent a species? It should be compound, no? Also, in the zoom in – Phenylalanine and Leucine – shouldn't these amino acids be present in the metabolome of all species? If I interpret the figure correct, there is no phenylalanine detectable in the metabolome of O. pumila and A. thaliana. And – though intuitive – but the color code of the heat map is missing. Suppl. Fig. 22 is also a heat map, correct? Camptothecin is only present in low concentrations in the hairy roots according to Suppl. Fig. 22. This is in conflict with cited literature.
Page 13:
Line 12: "This suggests the possibility of conserved gene families…." – Be aware, that the species named – C. roseus, G. sempervirens and C. acuminate are not closely related. Compared to other specialized metabolites and their occurrences, an independent evolution is possible. To answer this question, a sampling of species that fill the gaps in the phylogeny between C. roseus, G. sempervirens and C. acuminate would be necessary.
Line 20: wording - one does not "need" co-expression analysis to identify homologs of MIA biosynthetic genes, but of course is nice to see that these homologs are expressed in the tissues, where MIAs have been found.
Page 14:
Line 21: What was the rationale behind the analyses of all orthogroups concerning their expansion/loss/gain? It is not MIA biosynthesis related.
Page 17:
Line 10: I don't agree with the classification of TDC in two distinct groups – present in MIA producing plants and present in non-MIA producing plants. Fig. 6 b, same is true for STR, Fig. 6c. Furthermore, Fig. 6a: What are the red arrows indicating? It is not explained in the figure legend.
Page 19:
Lane 15: A cluster that includes at least one orthogroup specific to MIA-producing plants is not a cluster. A minimum of two orthogroups/gene/units can form a cluster. A single orthogroup can cluster in a synteny bloc with other genes, but then its not a biosynthetic cluster. In general, the current state of knowledge about the organization of MIA biosynthetic genes in clusters is not discussed.
Page 20:
Line 3: what evidence other than the absence of the STR gene supports the conclusion that Coffee lost the gene?
Page 30:
Line 4: I am not familiar with the term "aseptic plant"

I don't comment in detail the methods part. It includes, as mentioned above, results and needs thorough restructuring.

Reviewer #4 (Remarks to the Author):

I believe the authors had generated a high-quality assembly of Ophiorrhiza pumila by integrating multiple datasets produced by different platforms using advanced technologies. The continuity of the assembly was also experimentally verified and resorted according to the evidence of FISH. I think there are only minor issues should be addressed regarding the part of assembly.
(1) Page 7 Line 7. The contig N50 of Camptotheca acuminata is ~1.47 Mb. It seems the authors cited an earlier version of the assembly (Zhao et al., 2017) but in fact the assembly had also been improved to a higher level. Given the authors are describing their new strategy of genome assembly, why they compared their results only to anti-cancer MIA producing plant species? Is there any special difficulty in assembling the genome of this group? Otherwise, why not compare to the others?
(2) The authors pointed out the challenges in plant genome assembling, which could be caused by genome heterozygosity, polyploidy, and repetitive sequences. Does the "multi-tiered scaffolding

strategy" also work good in complex genomes? Or this strategy works for O. pumila only because it is a simple genome? It is no doubt that the assembly presented in this work is of high-quality, but whether the strategy is robust to other genomes, particularly complex genomes, such as polyploidy species, needs more tests. I suggest weaken the statement of this strategy as a better method for genome assembling unless it has been thoroughly tested.

Reviewer #1

This manuscript reports an enormous amount of works that allowed the authors to establish a new plant genome with assembly parameters of very high quality and with experimental validations by chromosome FISH, to depict the genome characteristics in detail, to comprehensively describe the metabolic diversity of the plant as well as a gene-to-metabolite network, and to propose important hypothesis on the evolution of genes involved in the biosynthesis of one of most diversified class of secondary metabolites, monoterpene indole alkaloids (MIAs). Plants producing MIAs provide several pharmacologically active molecules and anti-cancer drugs, such as camptothecin, vinblastine, vincristine. Deciphering the genetic and evolutionary mechanisms at the origin of the unique metabolic diversity of land plants is one of the major challenge in plant biology, and this work provides important resources towards this quest.

Major claims of the paper and critical assessment:

A near complete genome of the camptothecin producing medicinal plant *Ophiorrhiza pumila*, with very few gaps : 21 remaining assembly gaps over 11 chromosomes ! Most plant genome assemblies have from several hundreds to several thousands gaps. Although *O. pumila* genome is a typical plant genome with relatively high level of repetitive sequence, the authors combined four complementary next-generation sequencing technologies with an ordered multi-scaffolding approach and experimental validation of each scaffold at the assembly gaps by chromosome FISH to achieve a near complete reference plant genome assembly of *O. pumila*. In addition to the reference genome, two haplotype-resolved genome assemblies of *O. pumila* were generated with assembly parameters of very high quality, although with slightly more gaps than the reference genome. This report is of high interest for anyone in the field of plant genomic since it is one of the best plant genome assembly described (as shown in Supplementary figure 1 that compares the status of several plant genome assemblies). One important lesson from this work, based on detection of orientational error after bioinformatics-based assembly of *O. pumila* genome, is the needs for experimental validation of scaffolding models. That other plant genomes might needs similar validations was shown by the authors. The observation of unusual synteny relationships between published Coffea canephora genome and *O. pumila* genome, prompt the author to verify by FISH the coffee chromosome inconsistent segments, and substantiate a misassembly in the chromosome 2 of the coffee genome. Therefore, *O. pumila* genome assembly will likely provide a resource to improve plant

genome assembly and a model to understand the evolution of plant genome.

Another major claim of this work is a comprehensive description of nitrogen-containing metabolites in *O. pumila*. The nitrogen-oriented metabolome was achieved through an original methodology, previously reported by the group (Nature methods 2019, Analytical Chemistry 2020). It includes complete 15N stable-isotope labelling and chemoinformatics approach. 273 nitrogen-containing metabolites were assigned, mostly to indole alkaloids, MIAs, and carboline moieties containing metabolites. Tissue specific accumulation patterns were also described, and in combination with a transcriptomic profiling, a gene-to-metabolite network associated to MIA biosynthesis was identified that provide candidate gene for identification of the biosynthetic pathway molecular determinants. Candidate gene for biosynthesis of the central precursor for MIAs, strictosidine, were convincingly identified, based on gene homology to model plant species for MIA biosynthesis, like *Catharanthus roseus*, however recognition of downstream genes towards *O. pumila* specific pathway branch will require further works.

Most originally, interesting hypothesis for evolution of MIA biosynthesis in plants were elaborated. Ks plots analysis over selected plant genomes (within asterids) including three MIA-producing plant provided basis for characterization of whole genome evolution. A recent whole genome duplication was detected in the camptothecin -producing *Camptotheca acuminata* (Cornales), but not in *O. pumila* (Rubiaceae, Gentianales) neither in *C. roseus* (Apocynaceae, Gentianales). Previous work showed that MIA biosynthesis had acquired a different path in Cornales than in Gentianales as evidenced by the production of strictosidinic acid derivative in *C. acuminata* (Cornales) instead of strictosidine in Gentianales. This work now point out to specific evolution of MIA in Cornales with respect to lack of Strictosidine synthase orthogroup and presence of alternative genes to secologanin synthase.
Interestingly analysis of evolution of gene families involved in MIA biosynthesis showed faster evolution in MIA producing plants than in non-MIA producing plants, except, remarkably for tryptophan decarboxylase, an enzyme with potential broader functions in plant metabolism. Among those faster evolving gene families, SLS and STR were claimed to show positive selection.

Finally, the authors claims a role for secondary metabolite gene-clusters in evolution of MIA biosynthesis. Historically, metabolic gene-clusters were considered a characteristic of prokaryotes. Most genes of prokaryotic genomes are organized in clusters of coregulated genes with related functions. However, an increasing number of genes associated to plant secondary metabolic pathways have been reported in gene-cluster over the year. The iconic examples in plant secondary metabolism is the 10

gene-cluster encoding all enzymes specific to noscapine pathway in opium poppy (Science [2012] 336, 1704), but smaller gene-clusters, with a minimum of three and usually more genes, were also identified. The genetic process at the origin of metabolic gene-clusters are not clearly understood but do not depends strictly on the widespread process of local gene duplication and divergence producing tandem array of homologous genes. Well-characterized metabolic gene-clusters in plants involve a number of functionally related genes (e.g. involved in a common metabolic pathway), and most of theses genes are non-homologous (The Plant Journal [2011] 66, 66-79).

In my point of view, cluster of genes simply based on potential function in secondary metabolism do not provide a strong evidence for metabolic gene-cluster. Evidence for functional link shared by a significant number of genes in the cluster should be search for. Some gene-clusters described in this work appears to show functional link, for instance cluster C1541. However, a number of gene-cluster may also not have the same level of functional signification. In my point of view, it would be important to further characterize the signification of gene-clusters highlighted in *O. pumila*.

Author's Response- We do agree with the reviewer's opinion. While gene-clusters based on potential function in secondary metabolism is not enough to be regarded as strong evidence for metabolic gene-cluster, if that gene-cluster is consistently present and conserved across distant plant species producing a similar class of metabolites, then the gene-cluster gets a higher score of confidence to be associated with biosynthesis pathways. We used the same rationale to showcase the importance of C1541, which was conserved across all strictosidine derived MIA producing plants. The 33 MIA gene-clusters reported in this study were selected based on an unbiased approach, firstly by identifying secondary metabolic gene-cluster using PlantClusterFinder software, and next by using high-confidence homologs of functionally characterized MIA genes (Supplementary Table 18) as selection criteria, resulting in assigning 33 of 358 gene-clusters as MIA gene-clusters. Our results showed that 20 of 33 MIA gene-clusters were highly collinear across MIA producing plants. Further, we also observed that secoiridoid and MIA biosynthesis pathways were coexpressed and were represented by 29 of the 33 MIA gene-clusters. Among these, we identified C1541, which, when we explored for collinearity, identified STR as an important enzyme towards the evolution of MIA biosynthesis. In these ways, we have reported several features of MIA gene-clusters to assign functional significance. While the ability to identify conserved gene-clusters across plant species is subjected to the availability of high-quality genome assemblies, in this study, we could show significant collinearity between plant species across gene-clusters identified in the *Ophiorrhiza* genome. We have now described this aspect in the results and discussion section. One may use coexpression coefficients within a gene-cluster to assign a sense of the significance of gene-cluster, which we have now included as updated Supplementary Table 24. As coexpression within a given gene-cluster is not

an essential feature for plants as reported by numerous studies, we do not favor a strict condition-based scoring just based on coexpression analysis. In our opinion, coexpression and collinearity are the key features that provide significance to a gene-cluster identified based on the presence of potential functional genes, which we have proposed and provided in this study.

We also need to mention here that number of tissues used for expression and metabolite profiling in this study (six tissues for metabolome, seven tissues for expression analysis) is not comprehensive enough to rely too much on coexpression and correlation scores of biomolecules . For a comprehensive analysis, it would be ideal to have *O. pumila* plants (or hairy roots), being subjected to multiple biological and/or abiotic conditions to simulate or activate diverse metabolic processes, and to use them to perform coexpression and gene-to-metabolome association analysis. These big datasets could then provide sufficient statistical power to further attach more significance to an identified MIA gene-cluster. The objective of this study was to establish an excellent genome and metabolome resource to understand camptothecin biosynthesis, and to use it for comparative genome analysis to understand key events related to the evolution of MIA biosynthesis. Future studies and more omics datasets for *O. pumila* now as we have an excellent system, similar to what we have now for *Catharanthus roseus*, will enable us to further filter out and exclude some of the possible false positive gene-clusters and identify the more significant functional MIA gene-clusters.

Detailed comments.
Major revisions :

-Critical review of the manuscript on the gene-clusters: One of the criteria to identify functional gene-cluster is the correlation of expression of the gene members. With respect to *O. pumila* candidate gene-clusters, does the clustered genes show co-expression? PhytoClust (Nucleic Acids Res. 2017, 45:7049-63) provide analysis tool for co-expression analysis. In addition, do they belong to gene-to-metabolite network described in the supplementary figure 27?

Author's Response- While coexpression is indeed one of the criteria to identify gene-clusters in the microbial genome, the same may not necessarily be applicable in the case of plant genomes. Previously, Wisecaver et al. reported a lack of coexpression between identified and experimentally validated gene-clusters in the Arabidopsis, as observed in several other studies[1,2,3]. While coexpression for genes within a given gene-cluster does provide an interesting possibility of being functional and associated to the same biosynthesis pathways, it is not essential for most of the cases[2]. A recent study reported spatial conformations of gene-clusters within chromatin structure as a factor that

constrain coexpression within clusters of nonhomologous eukaryotic genes and suggest that gene-clustering in the one-dimensional chromosome is accompanied by compartmentalization of the 3D chromosome[3].

To check if this is also the case for the *Ophiorrhiza pumila* genome, we performed Pearson correlation coefficient (PCC) based coexpression analysis for genes assigned to a given gene-cluster **(updated Supplementary Table S24)**. Distribution for mean and median PCC values of 358 gene-clusters and for 33 MIA gene-clusters showed 75 percentile of distribution being between the range of 0-0.2, suggesting not a strong coexpression trend between the member genes of a given gene-cluster **(Figure 1 for the reviewer)**. Noticeably, the lower 25 percentile of mean and median PCC values of MIA gene-clusters were slightly higher. We could identify gene-clusters such as C1394, C1620, C1708, C1709, C1909, C1925, and C1959, which included 7-18 gene members and were coexpressed **(updated Supplementary Table S24)**. Among MIA gene-clusters, C1385, and C1749 were coexpressed. Although our results showed low co-expression values taken all genes together within a gene-cluster, we observed 262 gene-clusters with at least a pair of genes with PCC value over 0.7. Our results thus suggest a limited scope of coexpression among genes within gene-clusters. If we exclude genes with zero or low expression, then overall PCC values for gene-clusters significantly increases. This is consistent with previously reported gene-clusters in plants[2].

We understand the rationale for this comment from the reviewer. It is a very common expectation for considering gene-clusters with high co-expression as important for further functional characterization. Following reviewer's comments, we have now modified **Supplementary Table 24** and have included mean, median, maximum, and minimum PCC values for individual gene-clusters of *O. pumila*. We believe that including coexpression values in Supplementary Table 24 provides important information for our target readers to look for coexpressed gene-clusters. We also have collinearity information for gene-clusters in Supplementary Table 25. Gene-cluster information together with coexpression analysis, and collinearity with MIA producing plants do support our identified 33 MIA gene-clusters.

We thank the reviewer for the advice and this comment. We have added the following sentences to offer more details related to coexpression of gene-clusters in the manuscript **(Page 19, Line 12)**-“ In total, we identified 358 metabolic gene-clusters in the O. *pumila* genome, representing 3,551 gene models across 11 chromosomes (Supplementary Table 23). Expression analysis showed 24 gene-clusters including 954 genes with no expression across O. *pumila* tissues used for transcriptome profiling (Supplementary Table 24). Coexpression analysis for a given gene-cluster showed a low Pearson correlation coefficient (PCC) value, with the median PCC value for 3/4th of the gene-clusters being less than 0.3 (Supplementary Table 24). We identified metabolic

gene-clusters such as C1394, C1620, C1708, C1709, C1909, C1925, and C1959, which included 7-18 gene members and were highly coexpressed. Using the presence of at least one orthogene family associated with MIAs biosynthesis pathways from O. *pumila* gene models in the identified gene-clusters as the selection criteria, we assigned 33 gene-clusters as putative MIAs gene-clusters (Supplementary Table 18, 23-24). While MIAs biosynthesis associated genes were highly coexpressed, MIA gene-clusters showed low coexpression values among member genes. Among MIA gene-clusters, C1385 and C1749 were highly co-expressed. Although our results showed low coexpression within a gene-cluster, we observed 262 gene-clusters with at least a pair of genes having PCC values over 0.7. The fact that several of these gene-clusters included genes with no expression was one of the reasons for the low PCC score within a gene-cluster. The behavior of identified gene-clusters and associated coexpression values were similar to previously reported trends in other plant genomes[49, 50], suggesting a lack of wide-spread coexpression among member genes of associated gene-clusters in plants."

We have also provided expanded our discussion to put forward the coexpression aspect of MIA gene-clusters, and its biological interpretations (Page 24, Line 6 onwards).

About gene-to-metabolite network, yes, several of the included genes in the network were also assigned to gene-clusters **(Supplementary Table 19, and 24)**. We previously had following sentence to describe this **(In reference to first submitted version: Page 20, line 13)**, "These gene-clusters represent the pan-genome for MIAs biosynthesis and includes several functionally characterized genes as well as potential genes involved in the MIAs biosynthesis, which also showed a high correlation with nitrogen-containing metabolites identified in *Ophiorrhiza* metabolome."

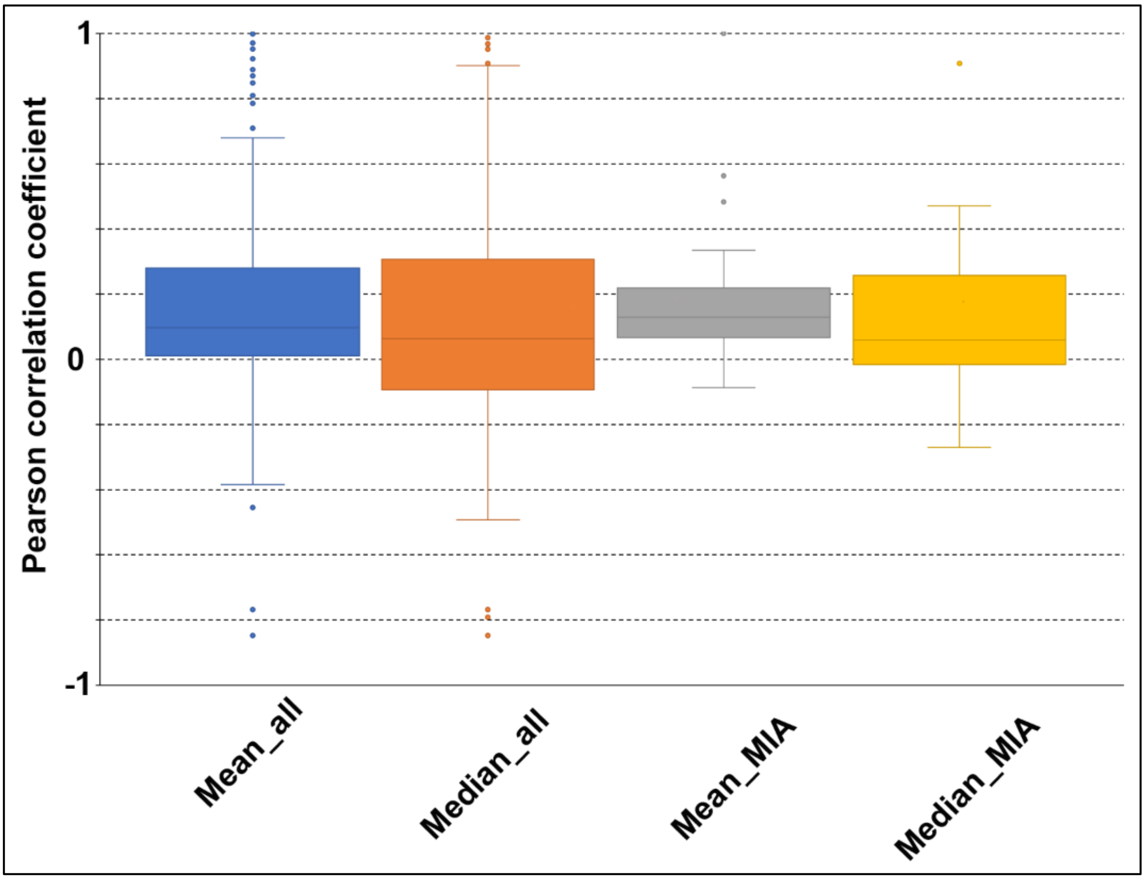

Figure 1 for reviewers: Box-and-whisker plot depicting distribution of mean and median Pearson's correlation coefficients (PCC) based on the expression of genes assigned to all identified gene-clusters and MIA gene-clusters.

Response Reference-

1. Jennifer H. Wisecaver, Alexander T. Borowsky, Vered Tzin, Georg Jander, Daniel J. Kliebenstein, Antonis Rokas. A Global Coexpression Network Approach for Connecting Genes to Specialized Metabolic Pathways in Plants. The Plant Cell May 2017, 29 (5) 944-959; DOI: 10.1105/tpc.17.00009
2. Satria A. Kautsar, Hernando G. Suarez Duran, Kai Blin, Anne Osbourn, Marnix H. Medema, plantiSMASH: automated identification, annotation and expression analysis of plant biosynthetic gene-clusters, Nucleic Acids Research, Volume 45, Issue W1, 3 July 2017, Pages W55–W63, https://doi.org/10.1093/nar/gkx305
3. Hans-Wilhelm Nützmann, Daniel Doerr, América Ramírez-Colmenero, Jesús Emiliano Sotelo-Fonseca, Eva Wegel, Marco Di Stefano, Steven W. Wingett, Peter Fraser, Laurence Hurst, Selene L. Fernandez-Valverde, and

Anne Osbourn. Active and repressed biosynthetic gene-clusters have spatially distinct chromosome states. PNAS June 16, 2020 117 (24) 13800-13809

-page 20, line 16 : "The entire secoiridoid biosynthetic pathways and MIAs biosynthesis-associated genes from the *Ophiorrhiza* genome were members of gene-cluster". What is the signification of this observation if the pathway associated genes are spreads among several different clusters ? From figure S26, I could count 40 different gene-clusters (C1318, C1320, C1321, C1327, C1385, C1401, C1418, C1423, C1444, C1445, C1453, C1454, C1493, C1497 ,C1501, C1504, C1527, C1532, C1537, C1538, C1572, C1592, C1624, C1635, C1643, C1746, C1747, C1748, C1749, C1752, C1810, C1824, C1914, C1953, C1381, C1541, C1559, C1565, C1684, C1693; why is this not in agreement with "33 gene-clusters" in page 20, line 10) , with 6 of them (C1381, C1541, C1559, C1565, C1684, C1693) having two different OG class genes and none more than two. Some gene-cluster have additional OG not shown in figure S26, that may add to this counting but if they are not shown, does that mean they are not expressed and therefore unlikely to play a function in MIA biosynthesis ?

Authors Response- In *Ophiorrhiza pumila*, we adopted a coexpression analysis strategy and identified candidate genes associated with secoiridoid biosynthetic pathways and MIAs biosynthesis. The statement, "The entire secoiridoid biosynthetic pathways and MIAs biosynthesis-associated genes from the *Ophiorrhiza* genome were members of gene-cluster", attempts to highlight the importance of gene-clusters for the discovery of potential candidate genes involved in the biosynthesis pathways. In the same section, we used following sentence to bring this point forward **(In reference to the first submitted version: Page 20, line 23)**, “Synteny between *O. pumila* and *C. roseus* or *G. sempervirens* genomes centered around gene- clusters were statistically significant based on Fisher’s exact test (p-value < 0.05), suggesting gene-clusters as the critical genomic regions for evolution and expansion of specialized metabolites (Supplementary Table 26).”

Unlike microbes, plant metabolic gene-clusters are scattered throughout the genome, as also the case for Arabidopsis and other plant species. Previously, Wisecaver et al. reported the absence of significant coexpression within a given gene-cluster in Arabidopsis, and so for several other plant species as described above in our previous response[1,2]. Indeed, expression analysis showed entire seco-iridoids and MIA-biosynthesis genes being highly coexpressed, all enzymes were also assigned to different gene-clusters. Instead of coexpression within individual gene-clusters, we identified coexpressed genes being associated with gene-clusters. MIA biosynthesis pathway is not present within a single gene-cluster, but instead spread across 33 MIA-gene-clusters, 29 of which were represented by highly coexpressed genes in Seco-iridoids and MIA biosynthesis pathways. Therefore, our statement suggests that the

member genes of gene-clusters represented in figure S26 could be potential candidate genes involved directly or indirectly towards the biosynthesis of MIAs.

To further emphasize above mentioned point, we have made some adjustments in terms of the ways we have expressed our results and rephrased the section (Please find the changes in the manuscript under track mode). We have also added following sentences in discussion section of the manuscript-

(Revised Manuscript: Page 24, Line 6 onwards) "As several functional metabolic gene-clusters identified in plant genome, identifying and analyzing gene-clusters seems to be a promising mean to identify candidates genes involved in the biosynthesis of specialized metabolites[55]. Previously, Wisecaver et al., using a coexpression network approach to understand specialized metabolites biosynthesis in Arabidopsis, reported a lack of coexpression associated with metabolic gene-clusters[49]. Similarly, several studies have also reported selective nature of coexpression for genes from a metabolic gene-cluster[39, 55, 56, 57]. In the *Ophiorrhiza* genome, we also observed a lack of coexpression trends within member genes of a given gene-cluster. However, we identified highly coexpressed genes associated with secoiridoids and MIAs biosynthesis assigned to 28 of 33 MIA gene-clusters reported in this study (Supplementary Fig. 27). Association of coexpressed genes assigned to secoiridoids and MIAs biosynthesis pathways to a gene-cluster was statistically significant based on Fisher Exact test. Further, 20 of the 33 MIA gene-clusters of the *Ophiorrhiza* genome were collinear across other MIA producing plants (Supplementary Fig. 32, and Supplementary Table 25). The scattered nature of metabolic gene-clusters seems prevalent across plant genomes, as observed in the case of MIA gene-clusters as well as previously reported secondary metabolic gene-clusters in other plant species[55, 58]. With the complexities associated with the gene expression regulation in plants, it is only rational that gene's physical proximities may not be enough to facilitate coexpression among genes within a gene-cluster[57]. On the other hand, gene-clusters do represent genome segments that serve as the hot-spots for retaining and evolving specialized metabolites biosynthesis. Benzylisoquinoline alkaloid (BIA) biosynthesis is one of the best-known specialized metabolites with enzymes forming gene-cluster within opium poppy genome. Nevertheless, the nature of gene-clustering was reported to be of heterogeneous nature with thebaine and noscapine pathways being highly clustered while morphine and sanguinarine pathways being scattered[56]. These suggest the possibility of the active evolution of genomic architecture through a combination of natural and artificial selection for specialized metabolites biosynthesis through gene-clusters. The gene-clusters, therefore, could be regarded as blocks of secondary metabolite modules, where mixes and matches of these modules result in a new chemotype, which may offer unique phenotypes for being positively selected. In the process of evolution, plants could lose some members of these modules or the entire module itself, and thus, would also lose the ability to evolve further or perfect the specific phenotype. On the other hand, plant species that could retain the

specific module could then derive the evolution of a unique phenotype towards perfection based on ecological challenges offered during the progression. Genome restructuring and dynamics, which is one of the key mechanisms towards evolution and speciation, at the gene-clusters does seem to provide an opportunity to evolve diverge chemotypes across plant species. In this study, we identified gene-cluster C1541 playing precisely this role for strictosidine-derived MIAs producing plants. This implies a selection pressure, favoring the clustering of genes involved in the biosynthesis of specialized metabolites, which could be the way forward to identify genes involved in the biosynthesis of common metabolite classes."

(Response to the Second half of the comment)

We apologize for the confusion in the number of MIA gene-cluster numbers. In total, we identified 358 gene-clusters, and using 216 high-confidence MIA gene sets identified based on sequence similarity and CD-HIT-based enzyme classification approach **(Supplementary Table 18)**, we identified and assigned 33 of these gene-clusters as MIA-gene-clusters. Out of these 33 gene-clusters, 29 were represented by member genes in the co-expressed enzymes associated with seco-iridoids and MIA biosynthesis pathways. Thus, 40 gene-clusters that the reviewer counted includes 29 MIA gene-clusters, while the rest were not identified as MIA gene-clusters as they did not meet the set criteria as explained in the method section. The criteria for defining the MIA gene-cluster are explained in the Method section (Page 60, line no 5 onwards). In order to specifically show MIA-gene-clusters to avoid this confusion we have excluded gene-cluster ids from our updated Supplementary Fig. 26 that are not assigned as MIA gene-cluster in our analysis. Nevertheless, genes and assigned gene-cluster ids are provided through Supplementary Table 24, and therefore, one if interested to look for a specific gene, they could always extract information regarding the associated gene-cluster. We are grateful to the reviewer to highlight this and for the help to improve Supplementary Fig. 26. We have also made changes in the figure legends and manuscript text (mentioned above) to avoid confusion.

The gene-clusters are expected to have at least two different OG (orthogene families). Typically, a gene-cluster is defined as a genomic segment with at least one gene encoding a so-called 'signature enzyme,' i.e., an enzyme that catalyzes the first committed step of the biosynthetic pathway and synthesizes the scaffold of the following specialized metabolites[2,3,4]. The remaining genes encode subsequent 'tailoring enzymes' which modify the scaffold to form the desired end-product. Therefore, for a gene-cluster, a minimum requirement, along with the genomic segment's length, is at least two genes encoding two different biochemical reactions and hence belong to different orthogene families (OGs). Therefore, all gene-clusters reported in this study have two or more OG class genes (Supplementary Table S20). We also realized that in Supplementary Fig. 26 (Previous version), we did not show the assigned OG to the gene names. We have now modified the figure and are thankful to the reviewer to bring our attention on this aspect.

The genes with no assigned orthogene groups were regarded as outgrouped genes based on OrthoFinder analysis, and hence, no orthogroup name is provided, it has nothing to do with gene expression.

Response Reference-

1. Jennifer H. Wisecaver, Alexander T. Borowsky, Vered Tzin, Georg Jander, Daniel J. Kliebenstein, Antonis Rokas. A Global Coexpression Network Approach for Connecting Genes to Specialized Metabolic Pathways in Plants. The Plant Cell May 2017, 29 (5) 944-959; DOI: 10.1105/tpc.17.00009
2. Satria A. Kautsar, Hernando G. Suarez Duran, Kai Blin, Anne Osbourn, Marnix H. Medema, plantiSMASH: automated identification, annotation and expression analysis of plant biosynthetic gene-clusters, Nucleic Acids Research, Volume 45, Issue W1, 3 July 2017, Pages W55–W63, https://doi.org/10.1093/nar/gkx305
3. Pascal Schläpfer, Peifen Zhang, Chuan Wang, Taehyong Kim, Michael Banf, Lee Chae, Kate Dreher, Arvind K. Chavali, Ricardo Nilo-Poyanco, Thomas Bernard, Daniel Kahn, Seung Y. Rhee. Genome-Wide Prediction of Metabolic Enzymes, Pathways, and Gene-clusters in Plants. Plant Physiology Apr 2017, 173 (4) 2041-2059; DOI: 10.1104/pp.16.01942
4. Hans-Wilhelm Nützmann, Anne Osbourn. Gene-clustering in plant specialized metabolism. Current Opinion in Biotechnology, Volume 26, April 2014, Pages 91-99

On the other hand, did the authors considered the possibility of metabolic gene-clustering at a supra-chromosomal level in the chromatin ? Does data from Hi-C contact map might be used to identify such potential gene-clustering ?

Authors Response- Analyzing metabolic gene-clustering at a supra-chromosomal level in the chromatin is certainly an interesting idea. However, using a single replicate of Hi-C experiment data generated in this study is not enough to draw any conclusion. To achieve a statistical significance, we will need to have few replicates and ideally, at least two conditions, to capture the dynamics of metabolic-gene-clusters. Indeed, treating hairy roots of *Ophiorrhiza pumila* with elicitors (such as jasmonic acid) and Hi-C based analysis between treated and control samples would provide more strong evidence as if a metabolic gene-cluster gets activated in the presence or in response to the elicitor. Since this study's objective was to achieve a high-quality resource to enable asking precisely this level of high resolution biologically relevant questions, we consider this out of scope from this study. We feel that this study would provide the quality of genome

assembly that will inspire many such investigations at the chromatin levels, as suggested by the reviewer.

Minor revisions :

Figures
-Although most figures in this manuscript are of very high quality, some data cannot be easily interpreted by non-specialist without some more information in legend. I have pinpoint most of these below.

Figure 1b : centromere positions are shown by a dotted lines which point to the pachytene chromosome picture but also cross the assembled chromosome double line at positions that do not seem to correspond to centromere positions. For instance, I presumed gap 1 in chromosome 1 is in the centromeric region. Gap 1 is far away from position of assembled chromosome crossed by the dotted line.

Author's Response- Thank you for pointing this out. Indeed, the centromere positions correspond to the pachytene chromosome pictures. We do not have evidence in this study to say that the assembly gaps correspond to the centromere or a wider centromeric region, which also are highly repetitive genomic regions. In order to correct this and avoid any confusion, we have now modified Figure 1, and replaced the dotted line with a triangle at the pachytene chromosome picture, suggesting it as the putative centromere. It is not possible to really scale chromosome size with the pachytene chromosome image, and therefore, now our figure addresses this issue. We have also made appropriate changes in the figure legends.

Figure 3e, the signification of the arrows are not mentioned in the legend.

Author's Response- Thank you so much for highlighting this. We have now added following sentence to the legend, "The arrow highlights the sign of recent whole genome duplication identified in *C. acuminata* genome". As no whole genome duplication was detected in *O. pumila*, we have now removed the arrow for its *Ks*-peak.

Figure 4. I do not understand the rationale for some of the metabolite ordering. For instance, deoxyloganic acid and loganin are grouped away from secologanin.

Author's Response- We have not adopted any rationale or format for the order of representation of metabolites in the Supplementary fig. 22 (now, it is Supplementary Fig. 23), and Figure 4. The objective for Figure 4 was to represent identified and confirmed metabolites of *Ophiorrhiza pumila* in relationships with other plant metabolomes.

Moreover, using Supplementary Fig. 22 (now, it is Supplementary Fig. 23), we showed the accumulation of specialized metabolites identified and its accumulation across different tissues of *O. pumila*. In many cases, we could propose putative chemical formula based on isotope labeling but arranging them through the logic of association was not possible **(Supplementary Table 14, and 15)**. Hence, we opted not to take this into consideration while reporting our results and the corresponding figure.

Figure 6 title should be reconsider with respect to my comment on convergent evolution below. Figure 6a does the legend for colour shading is related to the shading at the top right of the panel ? This top right part of the figure should be better explained in the legend. Figure 6b and d, please provide more information about busted analysis in legend. Figure 6e, what is the signification of red highlighted orthogene ?

Author's Response- Thank you for your comment. Following your comment, we have opted to change the figure title, which reads now as, "**Emergence of strictosidine synthase (STR), the starting point for the evolution of monoterpene indole-alkaloid biosynthesis in plants**"

For figure legend, we are grateful to the reviewer for pointing out specific details that were missing or needed better explanation. We have now modified legend for Figure 6, and have provided all details as pointed out by the reviewer.

Supplementary figure 1 should also highlight the status of other genome assembly from MIA-producing species. In addition, the authors should consider the possibility to include throughout their work some comparisons with the genome of the Apocynaceae Rhazya stricta published in 2016 .

Author's Response- Thank you for the suggestion. Initially, for figure S1 (now Supplementary Fig. 2), we decided to keep only those plant genomes that were at the chromosomal levels, and therefore, since none of the MIA producing plant genomes published to date is of chromosome level, these got excluded. We have now modified Supplementary Fig. 1 (now Supplementary Fig. 2) according to your suggestion and have included all published MIA-producing species genome statistics. We have highlighted MIA producing plants using a "red circle", and have modified the figure legends accordingly.

About the second comments, we initially intended to include Apocynaceae *Rhazya stricta* published in 2016 for the comparative genomics analysis in this report, however, we were not able to access the annotation file for the published genome. We attempted using the resource link from the authors, http://rhacyc.icmb.utexas.edu:1555/, but the

resource is not available/accessible. We attempted contacting the corresponding author of this study, but we received no response.

Next, we looked for the deposited assembly at NCBI Genome database, and although we could obtain whole genome fasta file (representing 979 super-scaffolds) and protein fasta files, we could not obtain cds, transcript and annotation .gff files.

In the absence of annotation file, we could not perform synteny analysis and several other comparisons which we were able to do with rest of the MIA producing plants.

Below are the links for the publicly available databases for *R. stricta* genome.

https://www.ncbi.nlm.nih.gov/genome/?term=Rhazya+stricta

https://www.ncbi.nlm.nih.gov/assembly/GCA_001752375.1/#/st

https://www.ncbi.nlm.nih.gov/Traces/wgs/MEJB01?display=contigs&page=1

Following reviewers' comment, we attempted predicting *R. stricta* gene-models with the publicly available genome fasta file. We performed a rough gene-prediction and used Augustus tool with Arabidopsis gene-models as training set. The number of the predicted gene models were 35450, and the BUSCO completeness was 80.4%. Our results using Braker2 based approach using RNA-seq based evidences resulted in 27,423 genes models, and with the BUSCO based analysis showed genome completeness of just 72.3%. The exact phrase from the *R. stricta* manuscript describing its predicted gene models is, "The transcriptome-guided annotation of the R. stricta genome identified 21,164 protein-coding genes (Supplementary Table S5), 60% of which were assigned UniProt identifiers. Comparison to a set of core eukaryotic sequences (CEGMA) demonstrated that the R. stricta assembly captured 98% of the expected genes.".

Our methods for predicting gene models are not the same as reported for *R. stricta* genome study, and hence, difference between number of the predicted gene models are not surprising. Nevertheless, the genome completeness analysis based on BUSCO or CEGMA is quite off, and hence, we are not sure if we could use our predicted gene models for rest of the comparison. Since the datasets were not available, we opted to exclude *Rhazya stricta* from our analysis for the reasons as explained above.

In this study, we have included two Apocynaceae species, namely *C. roseus* and *G. sempervirens* for the comparative genome analysis. Further, focus of this study is to understand evolution of Camptothecin biosynthesis using a high-quality omics resources generated through this study. Therefore, despite not being able to include *R. stricta* genome features for the comparative analysis, we believe that we were still successful in achieving the primary objective of our study.

Based on the reviewer's suggestion and available resources that we could access, we performed repeat analysis using *Rhazya stricta* genome, and the repeats classification is now included in the **Supplementary Table S12**. We have also modified text in the manuscript to provide comparison of *Rhazya stricta* genome with *O. pumila* based on available datasets.

(Page 12, Line 1), "A comparison of TEs across other plant species showed Gypsy-LTR as the dominant repeat class in *Ophiorrhiza*, *R. stricta*, *C. roseus*, *C. canephora*, *Nicotiana benthamiana*[26], and *Solanum lycopersicum*[27], while *C. acuminata* genome was dominated by Copia-LTR repeat type".

Supplementary Fig. 2 : please provide more information on colour bars signification. In addition to green, what means the light blue (turquoise) and dark blue bar, as well as the yellow colour and alignment shading signification. Since green bars are multicolour, it is not clear what part is from PacBio reads. Does the bar labeled Canu_contig represents PacBio reads ?

Author's Response- Thank you so much. This is a alignment plot from Bionano access software (https://bionanogenomics.com/support-page/bionano-access-software/). In Supplementary Fig. 2, Green bars represents the NGS assembly (PacBio assembly in this case), and regions which has conflicts, matches, or no support from Bionano reads are shown as red, dark blue and yellow lines, respectively. Since in few cases, the contigs are too big, the number of yellow lines for examples exceeds and hence appears to be painted as yellow. We have now modified our figure legend and have provided these informations to it.

Supplementary Fig. 6. Chromosome 3 was not identified by FISH probe (no red mark on ideogram of Chr 3) according to Figure S6c. Is that correct ?

Author's Response- Yes, that is correct. While we used all 11 chromosomes probes in our experiment, the probes used for chromosome 3 showed us a very weak signal, and we were not able to detect among the FISH probes for rest of the ten chromosomes. Despite several of our attempts, we were not able to get chromosome 3 labeled in the presence of all probes from different chromosomes simultaneously. There could be several reasons, for example, weak interaction or interference of other probes, among others. Nevertheless, we could manage to label 10 out of 11 chromosomes in Supplementary Fig. 6. We could also confirm and validate chromosome 3 individually, as shown in Figure 1B.

Supplementary Fig. 7. I do not understand what is shown in this figure. Which line is the reference genome, where are the wedges. Is the level of resolution sufficient to view the wedges ?

Author's Response- The Supplementary Fig. 7 (now, it is Supplementary Fig. 8) aims to provide evidence for the accurate genome assembly as supported by Bionano datasets. This is, in a way, the final genome assembly validation step using orthogonal NGS technologies (as we showed through Hi-C and Bionano based mapping). The difference between this figure and Supplementary Fig. 2 (now, it is Supplementary Fig. 3) is that while in Supplementary Fig. 2, we were looking for conflicts within contigs (Pre-scaffolding stage), in this figure, we were looking for any genome assembly region where we had conflicts or no support from Bionano datasets (Post-scaffolding stage). As shown in this figure, the entire genome assembly was supported by Bionano data. Similar to the figure legend of Supplementary Fig 2, the green bar is our final genome assembly (after polishing through Illumina reads) while light blue color represents Bionano assembly, and as can be seen, the entire width of individual chromosomes has been supported. The green and red shading across alignments between *O. pumila* final genome and Bionano assemblies represent insertions and deletions, respectively. The wedges, which actually are the shades, represents the region of structural variants, which is represented in the Supplementary Fig. 7

Supplementary figure 8 (now, it is Supplementary Fig. 9): what is the signification of the green pixels ?

Author's Response- The green pixels represents local interactions for the aligned contigs (based on Hi-C reads mapping to the *O. pumila* phase genome assembly) while blue pixels represents assigned chromosomes. The green pixels are the default view from Juicebox software (https://github.com/aidenlab/Juicebox) that we used to validate our Hi-C based scaffolded assembly. As not providing any specific information that we want to discuss, we have redrawn the plot and excluded green pixel in the modified version of this figure. No changes were made to the figure legend.

Supplementary figure 15. what are the specific legends for figure 15 a to f ?

Author's Response- Thank you so much for the comment. We have now provided details for Supplementary Fig. 15 a to f (now Supplementary Fig. 16).

Supplementary figure 17. Indicate in legend the meaning of numbers in the phylogenetic

tree (divergence time and branch length in Mya ago ?). Edit species name typos : sempervirens and benthamiana

Author's Response- Thank you. We have modified figure legend and have provided meaning of the numbers in the phylogenetic tree, (Yes, these are divergence time and branch length in Mya ago). We have also corrected the species name typos. Thank you for the correction and your careful observations.

Supplementary figure 26. The signification of the vertical blue lines is not obvious, explain in legend or use a more recognisable graphic legend.

Author's Response- Thank you so much. The blue vertical lines were representing grouping of genes assigned to the same gene-cluster. We have now modified our figure to make vertical blue lines self-explanatory. We have also modified our figure legend to reflect the changes.

Supplementary figure 30. This figure shows 4 genes from chr11 and 2 from chr5 for LAMT OG0000252 and 5 genes in OG0014261. However the figure 6A only show one gene (if I understand properly the colour shading) for LAMT OG0000252. Is this correct in figure 6A. Double check for potential similar mistakes.

Author's Response- Thank you for highlighting this point, as we identified an error in our color-bar legend. The legend for the color bar, reading "Number of genes", is a mistake. In order to normalize gene counts assigned to a given orthogene group across different plant species, we divided the number of genes assigned from a specific plant species by the total number of genes assigned for a given orthogene family, which was used as "Normalized gene counts" for the heatmap. This is the reason why the color codes range from 0 to 1. For example, OG0014261 in total included five genes (Supplementary Fig. 30), and all five genes are from *O. pumila*. Therefore, the normalized value for OG0014261 becomes 1, which is why it is colored max intensity (Fig. 6a). The same goes for all the other orthogenes. We have now modified the legend in the figure which says, "Normalized gene counts", and has added method for normalization in the figure legends. We have checked all our figures and have ensured no such errors.

Supplementary figure 31. "Results suggested evolution of STR as key event that preceded with evolution of genes associated with MIA biosynthesis." It may not be obvious for non specialist how this conclusion is drawn from this figure data ? Explain more clearly in the text. Or provide additional hints in the figure.

Author's Response- We appreciate your point. We have now added following sentence to clarify our results in the figure legend,

“**Supplementary Fig. 31. Evolution of secoiridoid and MIA biosynthesis associated orthogene families in camptothecin producing plants and coffee genome.** Median for synonymous substitutions per synonymous sites (*Ks*) were calculated using genes assigned to a given orthogene family for a plant species as described in the method section. Small *Ks*-median values for several key enzymes associated with MIA biosynthesis in *O. pumila* and *C. acuminata*, while high *Ks*-median values for genes from coffee genome suggests faster and active evolution of the functional genes in the camptothecin producing plants post established functional orthogene of STR enzymes. Results suggested evolution of STR as key event that preceded with evolution of genes associated with MIA biosynthesis, a factor that was missing for coffee genome.”

We have further added following sentences to explain our results, (Page 18, Line 20),

“For camptothecin producing plants, Ks-median for genes associated with MIA biosynthesis were significantly smaller for O. *pumila* and *C. acuminata*, while higher for coffee genome, which shares otherwise high genome collinearity and sequence similarity with *Ophiorrhiza* genome (Supplementary Fig. 31). Higher Ks-median for the MIAs associated orthogene families in the coffee genome suggests ancient origin for the genes that otherwise would have been actively evolving as suggested by smaller Ks-median value.”

Higher *Ks*-median for the MIA associated orthogene families in the coffee genome suggests ancient origin for the genes that otherwise have been actively evolving in the camptothecin producing plants as suggested by smaller *Ks*-median value **(Supplementary Fig. 31)**.”

This sentence is followed by our further explanation (in the same paragraph) on importance of STR in the evolution of MIA biosynthesis [(Page 18, Line 1) to (Page 18, Line10)].

Supplementary figure 32. Some MIA gene-clusters (e.g. C1321, C1327) present on Chr1 are not shown on figure 32a. Please show and check for other missing MIA gene-clusters on the other chromosomes. Please add Chr 3, 9 and 10 with their MIA gene-clusters.

Author’s Response- As we explained in our response to your comments on MIA gene-clusters, “From figure S26, I could count 40 different gene-clusters (C1318, C1320, C1321, C1327, C1385, C1401, C1418, C1423, C1444, C1445, C1453, C1454, C1493, C1497 ,C1501, C1504, C1527, C1532, C1537, C1538, C1572, C1592, C1624, C1635, C1643, C1746, C1747, C1748, C1749, C1752, C1810, C1824, C1914, C1953, C1381, C1541, C1559, C1565, C1684, C1693; why is this not in agreement with "33 gene-clusters" in page 20, line 10) , with 6 of them (C1381, C1541, C1559, C1565, C1684, C1693)”, we opted stringent criteria to define a gene-cluster as MIA gene-cluster. In our

response, we described our approach of using high sequence similarity and clustering of *O. pumila* genes with functionally characterized genes associated with MIA biosynthesis to identify high-confidence *O. pumila* MIA genes (Supplementary Table S18), and presence of at least one within a gene-cluster were used as criteria to annotate it as a MIA gene-cluster. In this way, we identified 33 gene-clusters, namely C0000, C1318, C1320, C1381, C1385, C1401, C1418, C1419, C1423, C1493, C1497, C1501, C1504, C1527, C1532, C1537, C1538, C1541, C1559, C1565, C1592, C1624, C1651, C1652, C1684, C1685, C1693, C1746, C1747, C1748, C1749, C1914, and C1953. These MIA gene-clusters were identified on chr2 (as Figure 7) and chr1, chr4, chr5, chr6, chr7, chr8, and chr11 (rest are shown in Supplementary figure 32), while none were identified on chr3, chr9 and chr10. Therefore, all MIA gene-clusters selected based on our described approach are shown in respective figures.

Content and ideas :

-Throughout the manuscript, I suggest to provide additional informations for non-specialist to better understand the importance of the achievements in this report. Since this work has the potential to attract a broad audience, more guidance should be provided to the reader to better understand the methodological approach and how to interpret the results. For instance, a few words on the characteristics and advantage of some of the newer NGS technology (Bionano, HiC) and assembly methodologies would help. How to compare Ks value and to interpret Ks plots may not be simple for non-specialist. What is the interest of using 15N labelling for metabolic analysis?

Author's Response- Thank you for the suggestion. We do share the views with the reviewer, but we are also restricted by the journal's word limits. With the volume of datasets and results that we have to describe in this study, we were limited in terms of how much details we could go in. We do agree that several aspects of this study would be of interest to a broad audience, and therefore, we have now briefly added sentences with appropriate references (and recent review articles) to provide additional information as well as to bring readers to the comprehensive overview of the topic in hand. These are the changes we have made in the manuscript (based on the three major points that reviewer advised for)-

1. **Characteristics and advantage of some of the newer NGS technology (Bionano, HiC) and assembly methodologies**

We have now added followings, (Page 5, Line 22)-" Next-generation sequencing technologies such as Bionano optical maps and Hi-C library sequencing-based approaches provide valuable orthogonal evidence to validate and improve reference genomes and for deriving chromosome level genome assembly".

2. **Compare Ks value and to interpret Ks plots**

We have now added following sentences (Page 18, Line 1)- "WGDs and small-scale genome duplications (SSDs) are the major source of evolutionary novelties and provide gene-pools to evolve new or specialized functions, which also play an important role in speciation[44, 45, 46]. Theoretical models for the evolutionary trajectories of duplicated genes propose that, in most cases, one copy of the duplicated gene retains the original function while another copy neutrally evolves without any selective constraints, thus resulting in either becoming inactive due to the accumulation of deleterious mutations or even deletion47. In certain cases, a small fraction of duplicates is also retained post gain-of-function mutations through positive selection forces[46]. The native gene-pools undergoes through a rapid rate of mutations, thus a lower Ks value compared to the ancestral gene-pools, resulting in the emergence of a new enzyme with a novel function."

3. **15N labelling for metabolic analysis**

We have now added following sentences (Page 12, Line 11), "Stable isotope labeling, coupled with high-resolution mass-spectrometry, offers a powerful approach to assign the number of atoms and chemical information to the detected metabolites. It increases the confidence in molecular formula determination for an identified metabolite feature by eliminating false positives while taking into account the elemental compositions[7, 40, 41]."

In all the above three explanations, we have provided relevant research and review articles to also provide further resources for interested readers.

-page 4, line 22: "A combination of the comparative genomics approach revealed the role of strictosidine biogenesis towards orchestrated evolution of down-stream enzymes of
MIA biosynthesis pathways". This strong statement claims major evidence for orchestrated evolution. What data show this? Unless further evidences are provided, I suggest rephrasing with wordings that are more careful.

Author's Response- We have now modified the statement as advised by the reviewer, and it reads as (Page 5, Line 10), "A combination of comparative genomics approaches suggested emergence of strictosidine synthase as a key event towards the evolution of strictosidine derived MIAs biosynthesis in plants."

Throughout this study, we have shown consistently how emergence and retaining STR was key towards the evolution of MIAs biosynthesis, as shown in Fig. 6a,d-f, Supplementary Fig. 31. By rephrasing as mentioned above, we tried to tone-down the statement, and we believe that the above statement is supported by our results in this study.

-page 19, line 5: "suggesting convergent evolution of". Convergent evolution imply independent evolution in different species of a character that was not present in their last common ancestor. Can you rule out that SLS and STR were not primitive characters in the MIA-producing plants ?

Author's Response- The rationale for us using the convergent evolution term for MIA biosynthesis(camptothecin) in this study are as follows-

1. We have discussed about the parallel route of MIA biosynthesis (camptothecin), one which adopts via strictosidine biosynthesis and catalyzed by STR, and another one is derived by strictosidinic acid (in *C. acuminata*).
2. MIA biosynthesis is highly restricted to Gentianales; exception includes camptothecin biosynthesis, which is also produced in plants from Cornales order, such as *C. acuminata*. These plants are distant, yet eventually resulted in the same chemotype.
3. For strictosidine derived MIA biosynthesis, evolution and specialization of functional STR was the key event. Evolution of downstream enzymes showed recent evolution (and smaller Ks-median value) post-emergence of the STR enzyme. On the other hand, *C. acuminata* showed no specialized STR, but rather evolved bifunctional SLS enzymes, which catalyzed the biosynthesis of loganic acid and secologanic acid, the key precursors to derive strictosidinic acid to subsequently synthesize camptothecin and other MIAs. Compared to STR (for strictosidine derived MIA producing plants), bifunctional enzyme SLS showed smaller Ks-median value, suggesting the neofunctionalization of enzymes involved in the secologanin biosynthesis. We also showed that the whole genome duplication followed established bifunctional enzyme SLS, suggesting that the strictosidinic acid synthesis played a key role in the evolution of subsequent MIAs biosynthesis in *C. acuminata*.
4. Genome duplications and transposable elements are key evolutionary forces that lead to evolving new phenotypes while perfecting biological processes to meet ecological challenges. We showed that compared to strictosidine derived MIA producing plants, *C. acuminata* showed a recent whole-genome duplication, and the dominant LTR repeats were completely different between *C. acuminata* and rest of the MIA producing plants. Our results showed different trajectories of genome evolution dynamics, which converged to a similar chemo-type.

Our results clearly showed two directions of evolutions, resulting in the synthesis of the same chemotype, camptothecin. We believe that the circumstantial evidence is enough to propose the hypothesis of a possible convergent evolution of MIAs in plants. Including more plant genomes, specifically ones that are close and fill the phylogenetic gap

between plants from Gentianales and Cornales (including plants that do not produce MIAs), will be required to provide strong evidence to our hypothesis.

We have removed the phrased "suggesting convergent evolution of" from the sentence as we felt we need to explain it further to propose this hypothesis (Page 19, Line 4). We have now expanded our discussion and have proposed our hypothesis of convergent evolution of MIA biosynthesis based on our results (Page 22, Line 14)

-page 20, line 8: "STR lost within the coffee genome at gene-cluster may have limited the opportunity to direct evolution towards MIA biosynthesis, which also explains higher Ks-median for enzymes associated with MIA biosynthesis". Can the authors exclude an alternative interpretation. MIA biosynthesis had evolved in Rubiaceae before divergence of Coffee. After Coffee divergence, lost of STR stopped positive selection on these genes.

Author's Response- By the statement, "STR lost within the coffee genome at gene-cluster may have limited the opportunity to direct evolution towards MIA biosynthesis, which also explains higher Ks-median for enzymes associated with MIA biosynthesis", we actually meant loss of positive selection or evolution of existing enzymes in the coffee to produce MIA biosynthesis.

If functional STR was present in the coffee genome, we would also expect synthesis of strictosidine in at least some cultivars or species from the Coffea genus. We performed a comprehensive literature search and could not identify any report of detecting strictosidine in any species from the Coffea genus. We also explored identified metabolites ions reported by Souard F, et al. (Metabolomics fingerprint of coffee species determined by untargeted-profiling study using LC-HRMS. Food chemistry 245, 603-612 (2018)), and could not identify strictosidine. This gives a strong indication that most coffee plants, or at least those used for metabolite profiling by Souard F, et al. should not have any functional STR gene. As reviewer mentioned, that one possibility is that after the loss STR, Coffea genus species did not pursue active specialization of enzymes for MIA biosynthesis as did *Ophiorrhiza pumila*, and that may explain high Ks-median value. This statement by itself means that the loss of STR resulted in the loss of the opportunity to evolve specialization for MIA biosynthesis. While we have no evidence to say that if we introduce STR in Coffee, it will start producing some of the intermediates of MIAs, nevertheless, the similarity of coffee genome with that of *Ophiorrhiza* and presence of homologs do make us believe that it will be the case, also based on our previous studies reported in Shimizu et al. Genome purification or expulsion of genes are representatives of genome dynamics, but expecting the entire biosynthesis pathway being expelled based on loss of STR seems very improbable if not impossible. As we just said above and reviewer mentioned, that coffee genome may still have those genes but were not positively selected. This by itself means that these enzymes then form the

part of native enzyme pools that serve to bring chemo-diversity for offering positive selection force, which implies that these genes lost the opportunity to actively evolve.

For us, stopping positive selection itself means loss of the opportunity to evolve specialization from the active pool of enzymes present in the Coffee genome. In that sense, our statement still holds the ground in either of the possibility. Further, by evolving, we do not mean getting a new enzyme, but rather specialization of existing enzymes. Our results showed a highly collinear genome of *Ophiorrhiza* and coffee genome. We could identify genes from the coffee genome in orthogene families representing all key enzymes associated with MIA biosynthesis (Supplementary Fig. 31). As shown in Supplementary Fig. 31 and described in our result section, the key difference between the coffee genome and camptothecin producing plants were no representation of coffee genes in OG0013616 (Orthogene family representing SLS, including genes that were functionally characterized to produce strictosidinic acid) and OG0015245 (Orthogene family for functional STR). With this difference, we showed that all major orthogene families evolved faster in the camptothecin producing plants (as well as in the MIA producing plants; Fig. 6e) but not in the case of the coffee genome, suggesting a dormant state (in terms of evolution/neofunctionalization) for these enzymes.

Specialization of existing enzymes through active evolution in the presence of strictosidine or strictosidine intermediates seems a most likely scenario based on the Ks-median values we observed between camptothecin producing plants and coffee genome. Shimizu et al., in a recent study, showed that expression of a foreign gene, La-L/ODC (Lupinus angustifolius L/ODC), catalyzing the conversion of primary metabolism towards quinolizidine alkaloid, in Arabidopsis result in the emergence of several new metabo-intermediates dues to enzyme co-opting phenomenon. In this study, the authors proposed the emergence of L/ODC was essential to allow native enzymes to catalyze cadaverine intermediates, resulting in the expansion of chemo-diversity, which eventually got perfected and specialized through evolutionary forces. Our results suggest the same pattern for MIAs biosynthesis. It is difficult to say if coffee had completely evolved MIA biosynthesis pathways. However, based on our results shown in Supplementary Fig. 31 and Fig. 6e, the rate of synonymous substitution (Ks) were high for coffee for all enzymes assigned to MIA orthogene families, suggesting no major changes or evolutional progress for these set of genes.

Response reference-

Shimizu, Y., Rai, A., Okawa, Y., Tomatsu, H., Sato, M., Kera, K., Suzuki, H., Saito, K. and Yamazaki, M. (2019), Metabolic diversification of nitrogen‐containing metabolites

by the expression of a heterologous lysine decarboxylase gene in Arabidopsis. Plant J, 100: 505-521. doi:10.1111/tpj.14454

-page 22, line 2. I disagree with the first part of the sentence. The pathway for catharanthine and vindoline are fully identified in *C. roseus* and may be one or two steps are missing to go from these MIAs to vinblastine. However, I agree that for camptothecin pathway elucidation and study of MIA pathway evolution, *O. pumila* genome will be an invaluable tool.

Author's Response- We have now rephrased this sentence, which now reads as,

"As the biosynthetic pathways of anti-cancer MIA camptothecin are not known, an accurate and very high-quality genome assembly and metabolome resources of *Ophiorrhiza* is valuable …."

Word choice, grammar, typos, etc.

Page 6, line 7: "The entire genome consist of just." this sentence is supported by figure 1B. Please refer to.

Author's Response- Thank you so much. We have now added the reference of Fig. 1b.

Page 10, line 17: "Parallel evolution of MIA biosynthesis in plants" this subtitle precedes a long description of *O. pumila* genome content, the authors should consider adding a dedicated subtitle for this part and shifting this one below.

Author's Response- Thank you so much. We have now changed the title, which now reads as, "Contrasting genomic features indicate convergent evolution of MIA biosynthesis". The initial description of gene-models predicted for *O. pumila* is important to keep our readers informed for the subsequent results and interpretation, and therefore, we wish to keep the present flow of results. Also, the new result section does matches with the content of this part.

Page 13, line 10: "secondary metabolite gene-clusters.. Reference 2, 31 , 33." Gene-cluster in some part of this manuscript either refer to hierarchical gene-cluster of expression (Suppl. figure 23) or to gene-cluster on chromosome. It is not clear what type of cluster is considered here since for instance reference 33 describes hierarchical gene-cluster of expression. Please use an alternative expression for gene-cluster when dealing with expression cluster to avoid confusion. Reference 31 does not seem to refer to gene-clusters. Some comprehensive review on metabolite gene-clusters should be cited.

Author's Response- Thank you so much. We have now adopted "gene-cluster" to refer to metabolic gene-clusters and differentiate it with "gene-clusters" identified based on expression and hierarchical clustering. About reference 31, it was to indicate that *O. pumila* and *C. roseus* (reference 31 reported identified MIAs in *C. roseus*) have shared metabolites, and hence conserved gene-clusters could be interesting to look for. We have now added a more appropriate reference for this sentence. We have also added more explanation to our discussion section to provide our interpretation of identified MIA gene-clusters with respect to previously published gene-clusters in other plants.

Page 23, line 19. F.K. is not in the author list, and R.F. is not in author contributions. Does F.K. should be R.K. ?

Author's Response- Thank you. You are correct, F.K. should be R.F. We have made the changes accordingly.

Page 31, line 12. Correct the number of molecules. Line 15 : bp. Line 21 : 42X in table S1. What is the correct fold ?

Author's Response- Thank you so much. We have made these changes. The correct coverage is 42X as mentioned in the Supplementary Table 1, and we have made appropriate corrections in the manuscript text as well.

Page 51, line 15 : acuminata (while I was writing this, my text editor did an improper correction into acuminate !), line 18 : acuminata.

Author's Response- Thank you so much. We have made the correction. We also wanted to say that we are so impressed with the reviewer's keen observations throughout his/her comments. Indeed, this mistake was due to text editor as happened to the reviewer. Our sincere gratitude to the reviewer.

Page 58, line 8 : supplementary. Line 10 : again here, gene-cluster refer to a different definition than with metabolic gene-cluster elsewhere in the paper. I suggest using a different wording to avoid confusion.

Author's Response- Thank you. We have now addressed this point as explained above.

Reviewer #2

Reviewed by Pr. Benoit St-Pierre
****

The authors have analysed and annotated the genome of *Ophiorrhiza pumila*,a plant which produces the antitumor alkaloid camptothecin. A special emphasis was laid on the genes involved in camptothecin biosynthesis as compared to the biosynthesis of othe monoterpene alkaloids.
This is a very comprehensive analysis covering many areas and topics. I really appreciate that the authors publish 1 big paper instead of several smal ones.

Author's Response- Thank you so much for your kind words. We completely agree with you about the importance of sharing a complete story as a single paper then several small ones.

The methodoly is excellent, the bioinformatics adequate and the ms is well written. Figures are of good quality (except the foto of the plant, which looks a bit out of focus in my copy).

There are afew typos in species names, e.g. not Coffee but Coffea.

Author's Response- Thank you for pointing this out. We have now corrected this in the manuscript. We have also checked the entire manuscript to avoid any similar errors.

The authors discuss the origin of the genes of alkaloid formation and have interesting conclusion.
I published a review in the pregenomic time, which might still be of interest

Wink, M.: Evolution of secondary metabolites from an ecological and molecular phylogenetic perspec-tive. Phytochemistry 64, 3-19, 2003
Wink, M. F. Botschen, C. Gosmann, H. Schäfer and P. G. Waterman: Chemotaxonomy seen from a phylogenetic perspective and evolution of secondary metabolism. In Wink, M. (Ed.); Biochemistry of plant secondary metabolism, Blackwell, Annual Plant Reviews Vol. 40, 2nd ed., 2010

Author's Response- Thank you so much. We agree that there are so many interesting results and reviews that we also wish to cite, for example, the first suggested review here. We are well aware of several of your articles on MIA biosynthesis, many predicted results

that we have reported in this study. One of the review article, which said exactly what we discovered was, “The cell and developmental biology of alkaloid biosynthesis. VincenzoDe Luca, Benoit St Pierre. Trends in Plant Sciences,Volume 5, Issue 4, 1 April 2000, Pages 168-173”, which in one sentence summary says, “The cell and developmental biology of alkaloid biosynthesis, which is remarkably complex, evolved in part by recruiting pre-existing enzymes to perform new functions”. This is exactly what we have observed and proposed through our analysis, and hence, we have now cited this article as well along with Wink et al paper from Phytochemistry. We also offer our apologies to not being able to include few more references here due to limitations on number of papers we could cite.

****

Reviewer #3

The authors of the article "Multi-scaffolding driven chromosome-level *Ophiorrhiza* genome revealed gene-cluster centered evolution of camptothecin biosynthesis" provide a very accurately assembled *de novo* genome of a very interesting model plant species. The applied sequencing and assembling strategy are thorough and very well and detailed described.

However, the article is weak when it comes to monoterpene indole alkaloid (MIA) biosynthesis part. It is indicative, that neither a single chemical structure of an MIA nor the current knowledge of their biosynthesis is illustrated in the introduction. In the results part, the authors introduce the names of biosynthetic enzymes, which is way too late.

It is also not clear in the introduction, what the authors consider as "camptothecin biosynthesis". Is it the biosynthesis beginning with the universal precursor of MIAs, strictosidine - or are earlier steps leading to strictosidine included. If so, the current state of knowledge should be given in the introduction. This will also help the reader to follow the authors strategy to identify MIA biosynthetic genes, especially, as in the methods part, the authors state, that 94 genes (suppl. table 16) that have been functionally characterized to be associated with MIA biosynthesis, were manually curated in their dataset.

Author's Response- Thank you. In our article, we aimed to focus on genome assembly strategy, experimental validation as essential component for next generation plant genomes, and evolution of secondary metabolism as three main research achievements and outcomes from this study. While we briefly described MIAs as derived from strictosidine and its vast diversity, we purposely left introducing MIAs biosynthesis pathways in detail as we dedicated more than half of this manuscript on MIA intermediates, biosynthesis pathways, gene-clusters and evolution of MIA biosynthesis pathways. We understand and agree with reviewers comment, and have slightly modified Introduction and added following sentences,

(Page 3., Line 6)," MIAs are natural products, derived from (S)-strictosidine, with monoterpene moiety derived from secologanin, an iridoid class of monoterpenes, and the indole moiety from tryptamine, a decarboxylation production from the amino acid tryptophan (Supplementary Fig. 1)".

(Page 3., Line 11)," Most of our current understanding of MIAs biosynthesis is restricted to vinca alkaloids derived from *Catharanthus roseus*. *C. roseus* has mainly been credited

for the elucidation of key biochemical steps, including secoiridoids biosynthetic pathways and subsequent modification of strictosidine towards MIAs biosynthesis due to the advantages such as the sequenced genome, comprehensive omics resources, and most importantly, the availability of an excellent experimental system to perform functional characterization of target enzymes[4, 5, 6, 7, 8, 9]. Camptothecin, another strictosidine derived and one of the most potent anti-cancer MIA, is the precursor for the commercial synthesis of topotecan and irinotecan, while several of its derivatives are under different stages of clinical trials[10, 11]. Camptothecin biosynthetic pathway and regulatory mechanism of production remain unknown despite being one of the most promising plant-derived anti-tumor drugs (Supplementary Fig. 1)[11]."

In the above-mentioned sentences, we have provided relevant references that offer current knowledge of MIAs biosynthesis.

We have also renamed our Supplementary Fig. 24 as Supplementary Fig. 1, which represents our current knowledge of MIAs biosynthesis pathways. In the new Supplementary Fig. 1, we have shaded what we mean or consider as "camptothecin biosynthetic pathway" (shaded as yellow).

We will like to thank the reviewer for this advice as we certainly feel some rephrasing of our introduction and bringing forward MIAs biosynthesis pathway (now Supplementary Fig. 1) allow a better flow of logic as far as MIAs biosynthesis and evolution part is concern.

Furthermore, MIAs are found in many diverse species from different plant families. As the authors state that they studied the evolution of MIAs, they should propose and discuss some evolutionary theories - e.g. did MIAs evolve in parallel in the different plant families and how is the organization in gene-clusters involved?

Author's Response- Thank you so much for your suggestion. We have restructured our discussion, and have offered our hypothesis based on the presented datasets,

(Page 22, Line 14 onwards) ,

"Among Gentianales, the emergence of STR for the synthesis of strictosidine was an important innovation to promote the evolution of MIAs biosynthesis, which occurred post-whole-genome triplication of core eudicot genomes (Fig. 6e, and Supplementary Fig. 31). While STR-like enzyme homologs were identified and assigned across plant species from different lineages (Supplementary Fig. 29a), functional STR (OG0015245) were specifically identified in the strictosidine derived MIAs producing plants (Fig. 6a, and Supplementary Fig. 29b). *C. roseus* and *G. sempervirens*, which diverged about 68 Mya from *Ophiorrhiza*, represented a single-copy gene for STR, while *Ophiorrhiza* genome

included two genes for the STR functional orthogene resulting from the tandem duplication. One of the exceptions to the otherwise highly restricted MIAs biosynthesis in Gentianales is camptothecin, which was first identified in *C. acuminata* of Cornales. Similar to other plant genomes analyzed in this study, *C. acuminata* lost the functional STR enzyme, and does not synthesize strictosidine. Instead, *C. acuminata* synthesizes strictosidinic acid, which derives the biosynthesis of MIAs including camptothecin[42]. For *C. acuminata*, the emergence of bi-functional SLS (OG0013616) was important towards the biosynthesis of strictosidinic acid, which incidentally also showed the fastest rate of substitution among all MIA producing plants (Fig. 6e, and Supplementary Fig. 31)[43]. With WGD peak for *C. acuminata* detected at Ks-peak 0.469, and Ks-median for SLS (OG0013616) being 0.75, our results suggest an earlier emergence of key metabolite intermediates pre-WGD in *C. acuminata*, which then served as a catalyst that allowed expansion and evolution of MIAs biosynthesis post-WGD (Supplementary Fig. 31). Synteny analysis between coffee and *Ophiorrhiza* genome showed significant genome-collinearity, yet the key enzymes lost in coffee genome included functional STR orthogene family. Coffee and *Ophiorrhiza* genome diverged at around 47 Mya, suggesting while STR enzyme evolved through SSDs in *Ophiorrhiza*, coffee genome instead lost the functional enzyme for strictosidine synthesis. Comprehensive metabolite profiling for several species from the Coffea genus could not detect strictosidine, including wild coffee species, suggesting the possibility of STR being lost across different species from Coffea genus[54]. Our study proposes retaining STR post-whole-genome triplication in core eudicots as the critical event that allowed selected plant species to evolve MIAs and its chemo-diversity (Fig. 6d-f, Supplementary Fig. 31)."

Further - as MIAs are secondary metabolites, they should also discuss the evolutionary pattern of other secondary metabolites in the discussion part, to put their own research in a broader frame. I think this would strongly improve the manuscript which is at this point, a very detailed explanation of a *de novo* genome sequencing.

Author's Response- Thank you so much for your suggestion. Keeping the core theme of secondary metabolites biosynthesis and role of gene-cluster to facilitate evolution, we have now modified our discussion and have added sentences,

(Page 24, Line 6)

"As several functional metabolic gene-clusters identified in plant genome, identifying and analyzing gene-clusters seems to be a promising mean to identify candidates genes involved in the biosynthesis of specialized metabolites[55]. Previously, Wisecaver et al., using a coexpression network approach to understand specialized metabolites biosynthesis in Arabidopsis, reported a lack of coexpression associated with metabolic gene-clusters[49]. Similarly, several studies have also reported selective nature of coexpression for genes from a metabolic gene-cluster[39, 55, 56, 57]. In the *Ophiorrhiza*

genome, we also observed a lack of coexpression trends within member genes of a given gene-cluster. However, we identified highly coexpressed genes associated with secoiridoids and MIAs biosynthesis assigned to 29 of 33 MIA gene-clusters reported in this study (Supplementary Fig. 27). Association of coexpressed genes assigned to secoiridoids and MIAs biosynthesis pathways to a gene-cluster was statistically significant based on Fisher Exact test. Further, 20 of the 33 MIA gene-clusters of the *Ophiorrhiza* genome were collinear across other MIA producing plants (Supplementary Fig. 32, and Supplementary Table 25). The scattered nature of metabolic gene-clusters seems prevalent across plant genomes, as observed in the case of MIA gene-clusters as well as previously reported secondary metabolic gene-clusters in other plant species[55, 58]. With the complexities associated with the gene expression regulation in plants, it is only rational that gene's physical proximities may not be enough to facilitate coexpression among genes within a gene-cluster[57]. On the other hand, gene-clusters do represent genome segments that serve as the hot-spots for retaining and evolving specialized metabolites biosynthesis. Benzylisoquinoline alkaloid (BIA) biosynthesis is one of the best-known specialized metabolites with enzymes forming gene-cluster within opium poppy genome. Nevertheless, the nature of gene-clustering was reported to be of heterogeneous nature with thebaine and noscapine pathways being highly clustered while morphine and sanguinarine pathways being scattered[56]. These suggest the possibility of the active evolution of genomic architecture through a combination of natural and artificial selection for specialized metabolites biosynthesis through gene-clusters. The gene-clusters, therefore, could be regarded as blocks of secondary metabolite modules, where mixes and matches of these modules result in a new chemotype, which may offer unique phenotypes for being positively selected. In the process of evolution, plants could lose some members of these modules or the entire module itself, and thus, would also lose the ability to evolve further or perfect the specific phenotype. On the other hand, plant species that could retain the specific module could then derive the evolution of a unique phenotype towards perfection based on ecological challenges offered during the progression. Genome restructuring and dynamics, which is one of the key mechanisms towards evolution and speciation, at the gene-clusters does seem to provide an opportunity to evolve diverge chemotypes across plant species. In this study, we identified gene-cluster C1541 playing precisely this role for strictosidine-derived MIAs producing plants. This implies a selection pressure, favoring the clustering of genes involved in the biosynthesis of specialized metabolites, which could be the way forward to identify genes involved in the biosynthesis of common metabolite classes. One possible explanation for the positive selection of gene-clusters is the reduced rate of recombination between genes involved in the local adaptation[55, 59]. A recent study reported the deletion of the entire noscapine biosynthesis pathway in five cultivars of opium poppy lacking noscapine while deletion of tandemly duplicated cluster of T6ODM genes from morphine pathways were identified as associated with cultivars lacking

morphine and codeine[56]. Positive selection of gene-clusters does have a possible role in providing chemotypes that may have implications for ecological/local success for a species or cultivar for successful propagation, as was reported for opium poppy[56]. Conserved nature and collinearity of metabolic gene-clusters of *Ophiorrhiza* genome across MIA producing plant species suggest a potential mean to select genes for functional studies. What role gene-cluster could play at the divergence of species is not clear, and more high-quality genomes of plants producing specialized metabolites are needed for comprehensive comparative genome analysis to understand evolutionary principles that allowed a wide distribution of metabolic gene-clusters across diverse plant species."

Another very general remark: the manuscript is way too long and it could be easily shortened if the authors would strictly describe the methods and exclude any results from the methods-chapter of their manuscript. They not only repeatedly present results in the methods part (which introduces a lot of redundancy to their manuscript), they also discuss their results in the results part - my advice: either have a combined results & discussion part, or really separate them.

Author's Response- Thank you so much. We have now modified our method section quite extensively and have removed any redundant description of results or information which is not required to emphasize major outcome of this study. We have also separated results and discussion part and removed any redundancies. Further, we have expanded our discussion part based on reviewers' comments. All the changes can be inspected through text in the tracking mode file.

I believe the data generated in this work is very valuable and interesting to the scientific community, but the writing of the manuscript has to be strongly improved.

Some specific major and minor comments:

Check Latin names of the species, sometimes "Coffee canephora" is written.

Author's Response- Thank you so much. We have now checked the entire manuscript and have verified and corrected places where it was applicable.

Page 3:
Line 4: vincristine is an original plant MIA, produced by *Catharanthus roseus*. The text suggest that it is derived from plant origin.

Author's Response- Thank you. We have now slightly rephased the sentence, which now reads as (Page 3, Line 3), "Among 30 categorized essential anti-cancer drugs by World Health Organization in 2015, several molecules, including topotecan, irinotecan, vincristine, and vinorelbine, are plant extracted or derived from plant origin monoterpene indole alkaloids (MIAs), such as camptothecin, and catharanthine."

Line 13: when *O. pumila* can serve as a toolkit to understand MIAs (plural) biosynthesis - how many different MIAs can be detected in *O. pumila*?

Author's Response- The purpose of this sentence, "*O. pumila* hairy roots have shown to accumulate high levels of camptothecin and serve as an experimental toolkit to understand MIAs biosynthesis for over a decade", was to say that *O. pumila* hairy roots provide an excellent system to explore the biosynthesis and regulation of camptothecin. Before this study, metabolites such as camptothecin, pumiloside, deoxypumiloside, strictosidine, and strictosamide were among reported MIAs in *Ophiorrhiza pumila*, while several biosynthesis genes were reported using *O. pumila* hairy roots. In this study, we chemically assigned 40 metabolites as MIAs, 14 metabolites as Indole alkaloids, and over 100 metabolites, which were not chemically assigned but identified as containing 2 nitrogen atoms and are potential alkaloids **(Supplementary Table 15)**. We have described details and discussed accumulation pattern for identified MIAs across multiple tissues of *O. pumila* **(Supplementary Fig. 23)**. The sole objective for complete nitrogen labeling and metabolome analysis was to identify diverse MIAs synthesized in *O. pumila*. We have cited articles in the introduction that provide details about known MIAs detected in *O. pumila* prior to this study, while the identified and expanded metabolome information is discussed in the result section.

Line 22: its MIA biosynthesis, and - does Coffea canephora produce the universal MIA precursor strictosidine? Or is this pathway completely absent in *C. canephora*?

Author's Response- Thank you so much. We have now corrected this in the manuscript.

We performed extensive literature review and looked for publications reporting targeted/untargeted metabolite profiling for *C. canephora* or other coffee species. Despite best of our effort, we could not find any description of reported strictosidine identification to any coffee species. We also explored identified metabolites ions reported by Souard F, et al. (Metabolomics fingerprint of coffee species determined by untargeted-profiling study using LC-HRMS. Food chemistry 245, 603-612 (2018)), and could not identify strictosidine. Therefore, we would like to believe that this pathway is completely absent in *C. canephora*. We have used our search results in our discussion section as well to propose our hypothesis of MIAs evolution in Plants.

Page 5:

Line 9: as the authors mention that polyploidy makes *de novo* genome sequencing challenging -what is the ploidy status of *O. pumila*?

Author's Response- *O. pumila* is a diploid genome (Supplementary Fig. 4a,b). Also, in our phased genome assemblies, we have haplotig1 and haplotig2, representing diploid state of this genome (Supplementary Fig. 5, 9). We have cited these figures when we reported number of chromosomes and phasing data.

Page 10:

The title "Parallel evolution of MIA biosynthesis": check the title, it does not fit -- in the first part of this chapter, repeats are described, and gene models, parameters for the quality of the genome - but nothing is said about the MIA biosynthesis.

Author's Response- Thank you. In this section, we showed differential genome evolution mechanism for MIA producing plants leading to same chemotype. Although we certainly cannot say parallel evolution, we can still use our results as indicative of potential convergent evolution towards MIA biosynthesis.

We have now modified our title, and it reads as, "Contrasting genomic features indicate convergent evolution of MIA biosynthesis".

We have also added following statement to conclude our section (Page 11, Line 19), "MIA biosynthesis is known to be remarkably restricted to Gentianales, such as Rubiaceae[24]. The exception being MIA quinolone derivatives, e.g., camptothecin, which is synthesized by the Rubiaceae members such as *Ophiorrhiza* as well as by *C. acuminata* of Cornales. Whole-genome duplications and transposable elements are regarded as key mechanisms for evolving novel features in plants[36, 37, 38, 39]. The differential repeat profiles across O. *pumila*, *C. roseus*, R. stricta, and *C. acuminata* genomes, and whole-genome duplication in *C. acuminata* suggest different trajectories of acting evolutionary forces, yet resulting in similar chemotype across MIA producing plants from Gentianales and Cornales orders. These results thus raise the possibility of either a convergent evolution of MIA biosynthesis in otherwise distant plant species, or an ancient origin of MIA biosynthesis, which subsequently lost repeatedly across plant species while retained by the producing plants."

Line 22: it was already described in detail, that chromosome 2 had to be rearranged.

Author's Response- The exact phrased used here was, "Predicted gene-models distribution along respective chromosomes was in a V-shaped valley form with low gene-density near the centromere for all 11 chromosomes, including chromosome 2, which was corrected based on FISH-based evidence (Fig. 3b)." Through this sentence, we wanted to re-emphasize the importance of experimental validation, as if not corrected, chromosome 2 would have completely difference gene-model distribution plot compared to rest of the chromosome.

We have now rephased this sentence to state this point, and now it reads as (Page 10, Line 11), "Predicted gene-models distribution along respective chromosomes was in a V-shaped valley form with low gene-density near the centromere for all 11 chromosomes, which would have been completely different for chromosome 2 if not corrected based on FISH evidences (Fig. 3b)."

Page 12:

Line 2: to test for a "recent" WGD, only paralogs of *O. pumila* are of interest, in my understanding. Orthologs give information on speciation. Linked to this - in Fig. 3e, two arrows are shown, the one indicating the newly identified WGD in *C. acuminata*, and the other??

Author's Response- Thank you. We have now slightly modified our Fig. 3e, and have retained one arrow that shows the emergence of peak representing recent whole genome duplication. We have rephased the figure legend to indicate this point. Since no WGD peaks were detected for O. *pumila*, we opted to exclude second arrow from the figure.

Line 6: only here MIA biosynthesis "starts" - but the things that are described here, should be stated in the introduction and citations should be included. E.g. "whole genome duplications and transposable elements are regarded as key mechanisms for the evolution of novel features in plants" - first, I miss the citation, and second, this statement is definitely no result.

Author's Response- We have now modified Introduction as advised by the reviewer.

About the phrase, (Page 12, line 6) "MIA biosynthesis is remarkably restricted to Gentianales, including Rubiaceae. The exception being MIA quinolone.....", we will like to keep the content the way it is. While we do agree that this phrase could become part of the introduction, we feel that this complements our results more appropriately.

About the sentence, "whole genome duplications and transposable elements are regarded as key mechanisms for the evolution of novel features in plants", indeed, this

is not a result but rather a statement to interpret our result. We have now added the relevant refence to the sentences that reviewer asked us. We have further elaborated on this point as explained in another comment from the reviewer described above.

Line 11: Why does the differential repeat profiles and the independent WGD in C. acuminate suggest an independent evolution of MIA biosynthesis? Where is the connection between repeat profiles and MIA biosynthesis? A WGD itself also does not per se effect a biosynthetic trait, if the trait was present before the WGD, it will be present thereafter. o test evolutionary scenarios, a trait has to be linked to the phylogeny of the species in which this trait occurs. One can do a character state reconstruction, for example. In case of MIAs, they occur in distantly related species and either evolved independently, or evolved very early and were subsequently lost repeatedly.

Author's Response- Indeed, MIA biosynthesis occur in distantly related species. WGD and transposable elements are indispensable evolutionary mechanisms to develop a novel phenotype/chemotype/feature, which eventually could offer force for positive selection. When distant plant species show contrasting modes of genome evolution yet result in achieving similar chemotype, one could argue the possibility of a potential convergent evolution or an ancient origin for MIA biosynthesis pathways.

One of the reasons for our hypothesis is due to the alternate biosynthesis pathway among camptothecin producing plants (strictosidine derived and catalyzed by STR, and strictosidinic acid derived and catalyzed by bifunctional enzyme SLS). The bifunctionality of SLS evolved only for *C. acuminata*, while have not been reported for strictosidine derived plants, which relies upon evolution of STR and strictosidine as the core intermediate. We have discussed these points in our manuscript. In the subsequent sections, we do provide our interpretation to propose our hypothesis of independent evolution of MIA biosynthesis. The above sentence was used to propose a hypothesis, which we pursue throughout our report, and discuss it extensively.

We have now modified our sentence, and have added following statement to clearly mention this,

(Page 12, Line 5), "These results thus raise the possibility of either a convergent evolution of MIA biosynthesis in otherwise distant plant species, or an ancient origin of MIA biosynthesis, which subsequently lost repeatedly across plant species while retained by the producing plants."

Line 17: What does the author mean by the term "active evolution"? And - how does this title relate to the chapter?

Author's Response- By active evolution, we referred to the enzyme families that evolved in *O. pumila*. Our metabolome results showed diverse nitrogen containing metabolites, which would require enzymes to catalyze their biosynthesis. Our analysis showed coexpressed genes from MIA biosynthesis pathways, and evolution of enzyme families specific to MIA producing plants as well as known to be involved in the specialized metabolites biosynthesis. We understand that "active evolution" gives a sense of dynamic state, which might be misleading when we are describing static state of evolution. Therefore, we have now modified our section heading, which now reads as,

"Diverse indole alkaloids corroborates with enzyme families evolved in *Ophiorrhiza* genome"

Line 21: the authors state, that 13C based metabolomes exist for 12 plant species, on the next page (line 3) the authors mention "metabolome space for previously analyzed 11 plant species". 12 or 11??!

Author's Response- Indeed, metabolome resource was generated for 12 plant species, *Ophiorrhiza pumila* being one of the 12 plant species used for metabolome analysis. Here, by saying "metabolome space for previously analyzed 11 plant species" in the next page, we meant while comparing *O. pumila* metabolome data with rest of the 11 plant species for which metabolome datasets were generated. We have now slightly rephased this sentence to avoid any confusion, and it reads now as,

"Compared to metabolome space for rest of the previously analyzed 11 plant species, *Ophiorrhiza* metabolome showed distinct and diverse nitrogen-containing metabolites including MIAs"

Figure 4: There is a legend, that assigns a color code to specific metabolite classes (Indole, Anthraquinones.), but there is also a color code in the circle plot - specific for species. This makes no sense to me. How can a slice of the circle plot represent a species? It should be compound, no? Also, in the zoom in - Phenylalanine and Leucine - shouldn't these amino acids be present in the metabolome of all species? If I interpret the figure correct, there is no phenylalanine detectable in the metabolome of *O. pumila* and A. thaliana. And - though intuitive - but the color code of the heat map is missing. Suppl. Fig. 22 is also a heat map, correct? Camptothecin is only present in low concentrations in the hairy roots according to Suppl. Fig. 22. This is in conflict with cited literature.

Author's Response- Thank you for giving us a chance to clarify our Figure 4. The figure represents accumulation trend for metabolites identified using complete ^{13}C labeling for 12 plant species (*Ophiorrhiza* being one of the 12 plant species) and newly acquired complete nitrogen labeling datasets for *Ophiorrhiza* metabolome. All samples were

treated exactly the same way, and metabolome profiling, fragmentation, and analysis were performed following the same pipeline as described in the method section and reported by Tsugawa et al. 2019 (Nature Methods volume 16, pages295–298. 2019).

The objective for this figure is to provide two main information's- (i) Relative accumulation of metabolites identified across 12 plant species, and (ii) Linkage between species based on daughter ions based metabolite-ontology classification. The links between two boxes are based on daughter ions based metabo-ontology as described in Tsugawa et al. 2019. For the circus plot, these are the steps that we followed-

1. We first filtered metabolites based on intensity irrespective of plant species (log10 intensity > 3.9). In total, we obtained 424 metabolites across 12 plant species, including 91 metabolites for *O. pumila*.
2. We next assigned any metabolite identified in *Ophiorrhiza pumila* to it's category, as we wanted to represent accumulation of identified metabolites with respect to other plant species.
3. For the metabolites that were not identified in *Ophiorrhiza pumila*, we assigned it to that plant category which showed highest accumulation among rest of the plant species.
4. We next plotted its accumulation across all 12-plant species as the heatmap.

A circus plot plant category does not mean that the metabolite is specific to it, but rather have the highest intensity among all compared plant species (the exception here is *O. pumila*, as we opted to include all identified metabolites, even if the levels were not highest in it), and then the heatmap showed its levels across other species. As can be seen from this plot, metabolites been highly accumulated in *Ophiorrhiza* are specific to it, and belongs to MIA and other specialized metabolite classes. Similarly, two licorice plants, *G. uralensis* and *G. glabra*, showed very similar types and accumulation of metabolites, which is what we expected as both plants are known to have similar chemotypes. Probably the circus plot classification/slice gives an impression that the metabolites belong a certain plant (which is what we normally expect in genome circus plot, where each slice represents a species while connections are based on relationships such as synteny or homology or so on). We have now modified our figure legend to clarify this in order to avoid any confusion as follows-

"Fig. 4. Metabolites of *Ophiorrhiza pumila*, assigned using ^{13}C and ^{15}N stable isotope labeling, compared with metabo-space of 11 plant species. The connections between metabolite features are based on metabolite network relationships defined by a correlation coefficient greater than 0.85. Highly accumulated metabolites across 12 plant species and their relationships in the form of metabo-ontology and scaled accumulation levels as a heat-map are shown here. Metabolites were filtered (log10 intensity > 3.9) and assigned to the *O. pumila* category. If a metabolite were not detected in *O. pumila*,

then the metabolite was assigned to the plant category with the highest accumulation compared to the rest of the plant species. * indicates chemically assigned metabolites based on pure standards or MS/MS analysis using public databases."

About Phenylalanine and Leucine, these metabolites were detected in both Arabidopsis and *Ophiorrhiza* as in the rest of the plant species. The metabolite levels were scaled, and therefore, were at lowest level for these two plants when compared to rest of the species. We have these metabolites mentioned with its accumulation across different tissues shown in Supplementary Table 15 (row no 18 and 20).

We have now provided all datasets, metabo-ontology based linkages and the rscript used to create this plot through GitHub (https://github.com/amit4mchiba/Circos-plot-for-12-plant-metabolome-analysis), and have provided information in the method section as well.

Thanks for bringing our notice about the color code. We have now corrected this in our updated figure.

About camptothecin, the results are not in conflict of the cited literature. The study that we cited showed high accumulation of camptothecin in the hairy roots, while it was not detected in the cell suspension culture (CSC) of *O. pumila*. Therefore, in our citated reference, authors used these two conditions to identify differentially expressed genes. In this study, we identified levels of metabolites across five tissues of *O. pumila* together with it's hairy root. Indeed, level of camptothecin were relatively low in hairy roots when compared to rest of the tissues. At the same time, we need to mention here that almost all intermediates of camptothecin biosynthesis pathways were highly accumulated in the root and the hairy root of *O. pumila*, which is believed as the tissues of active biosynthesis (Supplementary Figure 23). Our hypothesis is that once camptothecin is formed, it is transported to different tissues of the plant, but the site of biosynthesis is still localized. We were expecting camptothecin level just localized to the roots of *O. pumila*, but as shown, its relatively at the same levels across all tissues we analyzed in this study.

Page 13:
Line 12: "This suggests the possibility of conserved gene families.." - Be aware, that the species named - *C. roseus*, *G. sempervirens* and C. acuminate are not closely related. Compared to other specialized metabolites and their occurrences, an independent evolution is possible. To answer this question, a sampling of species that fill the gaps in the phylogeny between *C. roseus*, *G. sempervirens* and C. acuminate would be necessary.

Author's Response- Indeed, the plants named here are from distant families, particularly for camptothecin producing plants. We have in our responses mentioned that MIAs biosynthesis is highly restricted to Gentianales with the exception of camptothecin

producing plants. Nevertheless, it's also true that strictosidine or strictosidinic acid (in case of *C. acuminata*) serves as the universal precursor for all MIAs produced by plants. While we will certainly expect unique genes and gene-families that may result in specific MIAs identified or accumulated specifically in one of these plants, the fact that all MIAs share same origin for synthesis, expectation for conserved gene-families are not unreasonable as these plants share several MIAs and MIA-intermediates. In this study, we did included plants from Solanales, Asterales order along with plants from relatively broader lineages together with MIA producing plants from Gentianales and Cornales, and were able to report conserved and MIAs specific gene families. We do agree that a sampling of species, especially those from Gentianales but not a MIA producing species would be ideal to get a more accurate statement. We think that this could be something that could be explored in the future studies. Since the statement itself is indicative (or suggestive) and not affirmative, we have not rephrased the sentence.

Line 20: wording - one does not "need" co-expression analysis to identify homologs of MIA biosynthetic genes, but of course is nice to see that these homologs are expressed in the tissues, where MIAs have been found.

Author's Response- Agreed. We have slightly modified our sentence to avoid any misrepresentation of our statement, which now reads as,

"Secoiridoid biosynthesis genes were highly coexpressed with homologs of MIA biosynthesis-associated genes, including 10-hydroxycamptothecin O-methyltransferase, O-acetylstemmadenine oxidase (ASO/PAS), polyneuridine-aldehyde esterase (PNAE), perakine reductase (PR), rankinidine/humantenine-11-hydroxylase 3 (RH11H), sarpagan bridge enzyme (SBE), strictosidine beta-D-glucosidase (SGD), tabersonine-19-hydroxy-O-acetyltransferase (T19AT), tabersonine 3-oxygenase, and tetrahydroalstonine synthase (THAS) (Supplementary Fig. 25-27)."

Page 14:

Line 21: What was the rationale behind the analyses of all orthogroups concerning their expansion/loss/gain? It is not MIA biosynthesis related.

Author's Response- Our approach throughout this study was to first identify an overview of changes that occurred in *Ophiorrhiza pumila* genome with respect to plants from broader lineages, and then narrow-down our analysis to include MIA specific genes. We have talked about MIA specific gene expansion (Fig. 6a) and have also shown that several of the genes associated with MIA biosynthesis were specifically expanded to the producing plants. Including all orthogroups also allowed us to show that orthogene families present across all plant species analyzed in this study and those specific to MIA producing plants, are homologs of the same genes (For example STR, OG0015245 (MIA specific) and OG0000148 (present across all plants)). Also, our study's hypothesis is that

STR evolution promoted directed evolution of MIAs, which mostly used native pool of enzymes and evolved it to specialized functions. Therefore, including all orthogroups allowed us to have a background/reference with which we could compare and evaluate expansion/gain specific to MIAs producing species.

Page 17:

Line 10: I don't agree with the classification of TDC in two distinct groups - present in MIA producing plants and present in non-MIA producing plants. Fig. 6 b, same is true for STR, Fig. 6c. Furthermore, Fig. 6a: What are the red arrows indicating? It is not explained in the figure legend.

Author's Response- Thank you for your comment. It was an honest error from our end to include TDC in the sentence while we just meant SLS and STR, and we have now corrected this. For SLS and STR enzymes in Fig. 6b and Fig. 6d, the groups are not our classification, but rather based on orthogene analysis. In the case of SLS (Fig. 6b), "A" refers to orthogene OG0002438, representing diverse plant species, while "B" refers to orthogene OG0013616, which includes all functionally characterized SLS enzymes and specific to the MIA producing plants. In the case of STR (Fig. 6d), "D" refers to orthogene OG0000148, representing genes from diverse plant species, while "E" refers to genes specific to strictosidine derived MIA producing plants including all three functionally characterized genes from *O. pumila*, *C. roseus* and *G. sempervirens*. Therefore, we used the term distinct groups, present to MIA producing plants and those also present in non-MIA producing plants.

The arrow indicates orthogene families specifically gained or expanded in the MIA producing plants and key enzymes from MIA biosynthesis pathways. The orthogenes indicated through red arrows are also the orthogenes that we have highlighted in the Fig. 6e. We have now modified our legend and have added this information.

Page 19:

Lane 15: A cluster that includes at least one orthogroup specific to MIA-producing plants is not a cluster. A minimum of two orthogroups/gene/units can form a cluster. A single orthogroup can cluster in a synteny bloc with other genes, but then its not a biosynthetic cluster. In general, the current state of knowledge about the organization of MIA biosynthetic genes in clusters is not discussed.

Author's Response- Indeed. That was the criteria to define a metabolic gene-cluster in the *Ophiorrhiza* genome. Once we identified our 358 gene-clusters, we then looked within those gene-clusters that may have at least one of the MIA-associated orthogroups. As shown in Fig. 7 and Supplementary Fig. 32, all the 33 MIA gene-clusters reported in

this study have two or more orthogene families, including orthogenes associated with MIA biosynthesis.

We opted not to discuss in much detail about the previously reported MIA gene-clusters as those were based on non-chromosomal and fragmented genome assemblies, and there is little known about the MIA gene-clusters prior to this study. *C. roseus* and *G. sempervirens* genome publication reported STR-TDC gene-cluster, which we showed as conserved gene-cluster C1541. The reported gene-clusters were also very short, represented by just two or three genes in most of the cases. Our study showed the advantage of a high-quality genome assembly and the use of genome collinearity to identify gene-clusters for further characterization.

We have now included the following sentence to briefly state the previously reported MIA gene-cluster. (Page 20. Line 16), "Previously, *C. roseus* and *G. sempervirens* genome analysis also reported presence of STR-TDC pair and reported as identified gene-clusters in these species".

Page 20:

Line 3: what evidence other than the absence of the STR gene supports the conclusion that Coffee lost the gene?

Author's Response- We have several circumstantial evidences to support our conclusion. Firstly, we identified absence of STR gene at gene-cluster C1541 of *Ophiorrhiza pumila*, which otherwise was collinear and represented all the other enzymes such as TDC that were also present in *Ophiorrhiza* genome **(Fig. 6f)**. Second, we did literature search and looked for all publications reporting the metabolome of coffee and mined the raw metabolome datasets. We could not find any evidence of synthesis of strictosidine (previously reported or present in the metabolome datasets) in coffee. This suggest absence of enzyme catalyzing the synthesis of strictosidine in the plants. We also showed through Supplementary Fig. 31 as orthogenes associated with functional MIA biosynthesis enzymes showed higher Ks-median value in the coffee genome. This suggest dormant state of enzyme evolution, which otherwise evolved in MIA producing plants. We proposed that the absence of STR enzyme, and hence strictosidine, resulted in the loss of early metabolite precursors that could have resulted in evolution of MIA biosynthesis through neofunctionalization. These observations support our conclusion.

Page 30:

Line 4: I am not familiar with the term "aseptic plant"

Author's Response- Thank you. Here, we meant plant growing under aseptic conditions. We have modified this in the method section.

I don't comment in detail the methods part. It includes, as mentioned above, results and needs thorough restructuring.

Author's Response- Thank you. We have tried our best to exclude any of the results part from the method section and have attempted to concise results, discussion, and method sections. All changes can be reviewed by the manuscript under track mode.

Reviewer #4

I believe the authors had generated a high-quality assembly of *Ophiorrhiza pumila* by integrating multiple datasets produced by different platforms using advanced technologies. The continuity of the assembly was also experimentally verified and resorted according to the evidence of FISH. I think there are only minor issues should be addressed regarding the part of assembly.

(1) Page 7 Line 7. The contig N50 of *Camptotheca acuminata* is ~1.47 Mb. It seems the authors cited an earlier version of the assembly (Zhao et al., 2017) but in fact the assembly had also been improved to a higher level.

Author's Response- Thank you so much. Indeed, the genome assembly for *C. acuminata* that we described and used for comparative genome analysis were from Zhao et al., 2017, version 2. The version of *C. acuminata* genome that reviewer mentioned here is not publicly available in best of our knowledge. We know one on-going study that has attempted to improve *C. acuminata* genome assembly, and probably reviewer is referring to that, but we have no access to that assembly at this moment.

Given the authors are describing their new strategy of genome assembly, why they compared their results only to anti-cancer MIA producing plant species? Is there any special difficulty in assembling the genome of this group? Otherwise, why not compare to the others?

Author's Response- The objective for comparison of *O. pumila* genome with other MIA producing plants were to understand convergent evolution of MIA biosynthesis. We have compared *O. pumila* with other plant genomes as shown in now Supplementary Figure 2. Through this result, we could show current status of published genome assemblies, and in that comparison, assembly stats for *O. pumila*. We have explained in our results and discussion section about the widespread gaps and unassigned contigs to over 95% of all chromosome level plant genome assemblies, and we showed that we were able to overcome these difficulties and generate an excellent quality of reference genome.

We have also discussed quite extensively about our comparison with Coffee genome, and other non-MIA producing genomes such as tomato genome, grapes genome and ancient eudicot genome (AEK) throughout this manuscript **(Fig. 3a,e-d, Supplementary Fig. 10-12,17-20)**. We mentioned only about MIA plants in the result section to highlight the huge difference *Ophiorrhiza* genome could make for researchers working on MIA producing plants. Since the reason why we opted to choose *Ophiorrhiza pumila* for genome project was because of its ability to produce MIAs, and camptothecin, it serves as an excellent model for functional characterization of target enzymes, and well placed to explore evolution of MIAs biosynthesis, we felt the need to emphasize it. We have

stated in the manuscript that O. *pumila* genome is one of the best plant genome assembled ever, and certainly the best medicinal plant genome assembled ever.

In terms of repeat content, ploidy level, and genome sizes, MIA producing plants including *O. pumila* faces similar challenges as any plant genome assembly, and therefore, the method described in this study is applicable to any plant genome.

(2) The authors pointed out the challenges in plant genome assembling, which could be caused by genome heterozygosity, polyploidy, and repetitive sequences. Does the "multi-tiered scaffolding strategy" also work good in complex genomes? Or this strategy works for *O. pumila* only because it is a simple genome? It is no doubt that the assembly presented in this work is of high-quality, but whether the strategy is robust to other genomes, particularly complex genomes, such as polyploidy species, needs more tests. I suggest weakening the statement of this strategy as a better method for genome assembling unless it has been thoroughly tested.

Author's Response- The assembly pipeline used in this study is based on universal next generation sequencing technologies, and all algorithms used are applicable to all kinds of organisms irrespective of heterozygosity, polyploidy, and repetitive sequences. The reason why our approach was so successful and applicable to complex genomes are for as following -

1. The general approach for majority of plant genome assemblies is to achieve a pseudo-molecule, and therefore, there are no objective investigation of contig level assembly as most studies blindly believe the assembler (and since its *de novo* genome, unless the genome is previously sequenced, no way to find the correctness). In our strategy, we showed the importance of assembly validation at each step of assembly process, and even after assembly was completed with a very high contig N50, we performed FISH based validation and could still show the possible error. If genome assembly has errors (unidentified) at the contig stage, the assembly scaffolding is bound to carry the error forward, which will eventually impact the assembly contiguity.
2. One of the key messages that form our approach is the assembly validation step, and to included experimental validation of genome assembly as essential component of plant genome assembly pipeline.
3. Our approach emphasized on parameter optimization for each stage of assembly. For *O. pumila* genome, we showed that from default parameters to different assembler parameters we tested for Canu and Falcon-unzip made a big difference in terms of assembly contiguity we achieved at the end. The parameters used in this study will certainly not be applicable to all plant species,

but rather one will need to optimize parameters based on genome characteristics such as heterozygosity, polyploidy and so on.

4. While multi-scaffolding genomes are not uncommon, but the impact of order of scaffolding on final genome assembly quality have not been taken into consideration. Previously, for the goat genome, authors used multi-scaffolding approach and showed a significant improvement in genome assembly (https://www.nature.com/articles/ng.3802). In this study, we showed all combinations of assembly scaffolding using Bionano and Hi-C based methods and explained our rationale as how the scaffolding technology resolution allows to finetune the assembly when used a certain order.

Our described approach relies on (i) Parameter optimization, (ii) Assembly validation at each stage, and (iii) Multi-scaffolding, with the order of scaffolding does influences the final assembly outcome. The method used are universal and will be applicable to all plant species. We have specifically mentioned about these points in our results and discussion sections. Our observations and the approach described in this study provides a general to-do principle for any plant genome assembly project, and therefore, applicable to all plants with potential to improve assembly quality.

We do understand the point that reviewer wants to make through this comment, and therefore, we have now removed any word like "devised" or any strong word to tone-down the statement. We have also used the term, "in this study", to avoid any hint of generality of this approach since not tested on many plant genomes. We have also added following sentences in the discussion to comply with reviewer's suggestion.

(Page 27, line 18), "While its not possible to say if the same order of scaffolding technologies offers as significant improvement as we observed for *Ophiorrhiza* genome, our result certainly showed the importance of assembly validation at each stage of assembly. A stepwise scaffolding and error correction refine assembly at each stage, and therefore, assists in achieving high assembly contiguity. We also showed that despite achieving high contiguity and fewer assembly gaps, genome assemblies remain prone towards orientational errors at the assembly gaps, which needs to be addressed for all plant genomes."

Reviewer #1 (Remarks to the Author):

In this revised version, the authors have performed a careful revision of the manuscript taking in consideration most of my comments. I would like to thanks them for the detailed explanations provided for rebuttal.

1) However, on metabolic gene cluster (MGC) I am not completely convinced by a few points in their refutation. This said I consider the revisions provided are very useful to improve the signification of their data on MGCs. I disagree about the importance of coexpression within gene belonging to a MGC. In my point of view, this is a critical point. I understand this is a matter of debate in the literature, that other authors may have a different point of view of mine although a number also agree with it. So I would like to raise my last comments on this point and leave it up to the authors to consider it or not.
With respect to my comments on metabolic gene clusters, the authors have provided new data that will help to assess the significance of the proposed metabolic gene clusters, like the coexpression data in Supp table 24 and many other improvements. That's great. But on page 24 and line 6 onward, I do not agree with the following sentence: "Previously, Wisecaver et al., using a coexpression network approach to understand specialized metabolites biosynthesis in Arabidopsis, reported a lack of coexpression associated with metabolic gene-clusters." In my understanding, Wisecaver et al. rather consider that a lack of coexpression whithin some metabolic gene cluster is an indication of false positives and they report that most functionally characterized gene cluster are coexpressed. Wisecaver et al. wrote "in contrast, bioinformatically predicted biosynthetic gene clusters (BGCs) (i.e., those lacking an associated metabolite) were no more coexpressed than the null distribution for neighboring genes. These results suggest that most predicted plant BGCs are not genuine SM pathways and argue that BGCs are not a hallmark of plant specialized metabolism".
In addition, this is what Wisecaver et al. are indicating in the peer review report available online: "In the last few months, several papers have been released online that argue for the existence of thousands of BGCs in myriad plant genomes based solely on gene location information. See Schlapfer et al. 2017 Plant Physiology; this paper is now cited in our introduction. See also two preprint articles: Kautsar et al. http://biorxiv.org/content/early/2017/02/17/083535 and Toepfer et al. http://biorxiv.org/content/early/2016/10/07/079343. Based on our co-expression analysis, we believe focusing on BGCs for identification of SM pathways in plants is a siren song: the analysis is relatively quick and straightforward but because it is based on unvalidated assumptions (that plant SM pathways are typically clustered), it yields mostly false positives."
I am not claiming this is the case for most of metabolic gene clusters reported in this paper, since you also have for some gene collinearity data and some coexpression data but you should reconsider your interpretation of Wisecaver results.

2) About my comments on convergent evolution of MIA : Thanks for these clarifications. I realize that I initially misunderstood your claims about convergent evolution in the original version of the manuscript. I did not realize it was concerning camptothecin biosynthesis specifically. Of course, I agree that the two roads for camptothecin biosynthesis in Cornales versus Gentianales are evidence of convergent evolution. The discussion on page 22 and downward now provide more detailed argumentation on this point.

3) Clarifications and new data on MIA evolution in Rubiaceae are great and helpful.
4) The new description on the supplementary Fig. 31 are also very helpful.
5) I will not further comments on many other modifications provided by the authors that I agree with and many thanks again for the detailed explanations provided.
6) According to the journal instructions on the reviewing process, I added my name at the end of the 'comments for authors'. This was apparently misientified as the beginning of reviewer 2 comments. I(Pr. Benoit St-Pierre) am reviewer #1.

Reviewer #2 (Remarks to the Author):

The authors have paid great attention to the recommendations of the reviewers.
For my part, I am happy and would suggest to accept the ms.

Reviewer #3 (Remarks to the Author):

Dear Editors, dear authors,

The revised article "Multi-scaffolding driven chromosome-level Ophiorrhiza genome revealed gene - cluster centered evolution of camptothecin biosynthesis" was improved in many ways. Also, the authors gave a very detailed answer to the questions raised in the first round of the review process. Still, my impression is the following: the results produced by the authors are very interesting, but the article in its present form does not present the results in clear way and the article is very wearisome to read – especially for a non-specialist reader. To illustrate this: I really like to involve and discuss actual research papers in the lessons that I teach at the university. The paper in its presented form – I am really sure that the students, even the graduated ones - will have a very hard time to interpret the data and to get the message. My strong advice to the authors: If you want to address a broad audience, I really would suggest a thorough revision of the manuscript which in my opinion needs a clearer structure, a clearer message. The readers are in my opinion overwhelmed by the data you provide but this is not helpful if you want that the reader really understands your results/work.

Some precise comments: Concerning the parallel or convergent evolution - I really miss a phylogenetic tree of the species that produce MIAs – the basis for formulating evolutionary hypothesis. And a basis for comparative analyses. I am sure that not all readers have an idea of the evolutionary relationships of Apocynaceae, Rubiaceae, …… A little phylogenetic tree would nicely illustrate the evolutionary background of the species that produce MIAs. Especially in the light of the chapter "Contrasting genomic features indicate convergent evolution of MIA biosynthesis" such a tree would be very illustrative. And talking about this chapter: It still describes many interesting genomic features of the O. pumila genome (gene models, tRNAs,….), and other interesting observations like divergence times, WGD events. No doubt, all interesting observations, but I don't see any causal connection to MIA biosynthesis and how the authors can conclude from the results described in this chapter, that MIA biosynthesis has evolved convergently. The differential repeat profiles across O. pumila, C. roseus…. just proof that the repeats experienced different evolutionary forces but he MIA biosynthetic genes are possible under purifying selection and thus remain in the genome/species – unaffected by the evolutionary fate of the repeats.
A few other things:
"Diverse indole alkaloids corroborates with enzyme families evolved in Ophiorrhiza genome" I don't get the message of the title.
Fig. 4: I had some questions concerning Figure 4 concerning the color codes/legends, which are more than misleading in my opinion. It is of course nice that the authors kindly clarified Figure 4 to me, but the fact that the figure seems to be unintuitive did not really motivate the authors to improve the figure. The color code in the legend is still unclear. Some colors like brown are used in the circle plot, but are not given in the legend.
In Tsugawa et al. 2019 (Nature Methods volume 16, pages295–298. 2019) – the circle blot in Fig. 2 is nicely explained. But in the submitted paper, it is misleading. I try to be more specific:

In the legend, the green color indicates "Indole", in the circle plot – the green color seems to indicate "Arabidopsis thaliana". If you want that the reader gets your message – indole = highest intensity in Arabidopsis - then please adjust your figure. The lines/rows in the circle represent the species, and the "columns" represent the compounds.
And also, please be aware – in the legend, the colors indicating organic acids and terpene glycoside

are hard to distinguish.

To summarize: Very interesting results were produced, but the writing needs more structure and focus.

Reviewer #4 (Remarks to the Author):

All my conerns have been well addressed.

REVIEWERS' COMMENTS

Reviewer #1 (Remarks to the Author):

In this revised version, the authors have performed a careful revision of the manuscript taking in consideration most of my comments. I would like to thanks them for the detailed explanations provided for rebuttal.

1) However, on metabolic gene cluster (MGC) I am not completely convinced by a few points in their refutation. This said I consider the revisions provided are very useful to improve the signification of their data on MGCs. I disagree about the importance of coexpression within gene belonging to a MGC. In my point of view, this is a critical point. I understand this is a matter of debate in the literature, that other authors may have a different point of view of mine although a number also agree with it. So I would like to raise my last comments on this point and leave it up to the authors to consider it or not.

With respect to my comments on metabolic gene clusters, the authors have provided new data that will help to assess the significance of the proposed metabolic gene clusters, like the coexpression data in Supp table 24 and many other improvements. That’s great. But on page 24 and line 6 onward, I do not agree with the following sentence: “Previously, Wisecaver et al., using a coexpression network approach to understand specialized metabolites biosynthesis in Arabidopsis, reported a lack of coexpression associated with metabolic gene-clusters.” In my understanding, Wisecaver et al. rather consider that a lack of coexpression whithin some metabolic gene cluster is an indication of false positives and they report that most functionally characterized gene cluster are coexpressed. Wisecaver et al. wrote "in contrast, bioinformatically predicted biosynthetic gene clusters (BGCs) (i.e., those lacking an associated metabolite) were no more coexpressed than the null distribution for neighboring genes. These results suggest that most predicted plant BGCs are not genuine SM pathways and argue that BGCs are not a hallmark of plant specialized metabolism".

In addition, this is what Wisecaver et al. are indicating in the peer review report available online: “In the last few months, several papers have been released online that argue for the existence of thousands of BGCs in myriad plant genomes based solely on gene location information. See Schlapfer et al. 2017 Plant Physiology; this paper is now cited in our introduction. See also two preprint articles: Kautsar et al. http://biorxiv.org/content/early/2017/02/17/083535 and Toepfer et al. http://biorxiv.org/content/early/2016/10/07/079343. Based on our coexpression analysis,

we believe focusing on BGCs for identification of SM pathways in plants is a siren song: the analysis is relatively quick and straightforward but because it is based on unvalidated assumptions (that plant SM pathways are typically clustered), it yields mostly false positives.”
I am not claiming this is the case for most of metabolic gene clusters reported in this paper, since you also have for some gene collinearity data and some coexpression data but you should reconsider your interpretation of Wisecaver results.
Author’s Response- Thank you so much. We are in complete agreement with the reviewer. We do agree that bioinformatics based gene-cluster discovery is going to include several false positives. In that sense, Wisecaver et al., in their discussions, emphasized that the prominent signature for enzymes to be associated with specialized enzymes is being coexpressed. Nevertheless, the authors also highlighted that proximity of genes associated with secondary metabolic pathways along chromosomes was statistically significant in Arabidopsis.

We believe that it is very early to comment if metabolic gene-clusters need to be coexpressed or collinear with plant species producing similar secondary metabolism. As the number of high-quality plant genome assemblies has only recently started emerging, we hope that secondary metabolic gene-clusters' features will be further clarified soon. Our results showed collinearity as key and statistically significant for proposed MIA gene-clusters. Comparative genome analysis of plants producing similar metabolite classes would provide clues as this feature is conserved across plant species.

Following the reviewer's comment and suggestion, we have now modified the discussion section. It now reads as-
“Wisecaver et al., noting that the physical proximity of genes associated with metabolic pathways is statistically significant in Arabidopsis, suggested gene coexpression as a key feature for identifying enzymes associated with known specialized metabolic pathways irrespective of the location of their genes in the genomes49.”

Using this change, we tried to convey that the association of coexpression and gene proximity with secondary metabolite biosynthesis pathways is not clear yet. More research will be required to address this question.

2) About my comments on convergent evolution of MIA : Thanks for these clarifications. I realize that I initially misunderstood your claims about convergent evolution in the original version of the manuscript. I did not realize it was concerning camptothecin biosynthesis specifically. Of course, I agree that the two roads for camptothecin biosynthesis in Cornales versus Gentianales are evidence of convergent evolution. The discussion on page 22 and downward now provide more detailed argumentation on this point.
Author’s Response- Thank you so much.

3) Clarifications and new data on MIA evolution in Rubiaceae are great and helpful.
Author's Response- Thank you so much.

4) The new description on the supplementary Fig. 31 are also very helpful.
Author's Response- Thank you so much.

5) I will not further comments on many other modifications provided by the authors that I agree with and many thanks again for the detailed explanations provided.
Author's Response- Thank you so much for helping us to improve the manuscript significantly. We are immensely grateful for your comments on gene-cluster, which we were able to address by adding discussion points and data, which certainly have to improve the current version's content.

6) According to the journal instructions on the reviewing process, I added my name at the end of the 'comments for authors'. This was apparently misientified as the beginning of reviewer 2 comments. I(Pr. Benoit St-Pierre) am reviewer #1.
Author's Response- Thank you so much.

Reviewer #2 (Remarks to the Author):

The authors have paid great attention to the recommendations of the reviewers.
For my part, I am happy and would suggest to accept the ms.
Author's Response- Thank you so much.

Reviewer #3 (Remarks to the Author):

Dear Editors, dear authors,
The revised article "Multi-scaffolding driven chromosome-level Ophiorrhiza genome revealed gene -cluster centered evolution of camptothecin biosynthesis" was improved in many ways. Also, the authors gave a very detailed answer to the questions raised in the first round of the review process.
Still, my impression is the following: the results produced by the authors are very interesting, but the article in its present form does not present the results in clear way and the article is very wearisome to read – especially for a non-specialist reader. To illustrate this: I really like to involve and discuss actual research papers in the lessons that I teach at the university. The paper in its presented form – I am really sure that the students, even the graduated ones - will have a very hard time to interpret the data and to get the message. My strong advice to the authors: If you want to address a broad audience, I really would suggest a thorough revision of the manuscript which in my opinion needs a clearer structure, a clearer message. The readers are in my opinion overwhelmed by the data you provide but this is not helpful if you want that the reader really understands your results/work.

Author's Response- Thank you so much. We admit that the data that we attempted to present in this study were too diverse with independent scope of discussion, while we tried to focus on the converging aspect of the comparative genomics and multi-omics analysis. In order to strike a balance between describing individual analysis as well as the associations while keeping the formatting restrictions of Nature communication, we asked help from professional editors and non-specialist to improve writing of this manuscript. We got our manuscript edited for flow of content, English and ease of reading by opting services from Springer Nature Author Services (https://authorservices.springernature.com/). We further requested two of our colleagues to critically review the manuscript for flow and content, and we modified the manuscript accordingly.
All changes can be viewed by going through "Manuscript under track mode". We have modified acknowledgement section to thank our colleagues for their input/suggestions. The scientific content of the manuscript even after all changes remains the same. We believed that after these changes, the latest version of manuscript has significantly improved, and we will like to thank reviewer for this critical comment.

Some precise comments: Concerning the parallel or convergent evolution - I really miss a phylogenetic tree of the species that produce MIAs – the basis for formulating evolutionary hypothesis. And a basis for comparative analyses. I am sure that not all readers have an idea of the evolutionary relationships of Apocynaceae, Rubiaceae, …… A little phylogenetic tree would nicely illustrate the evolutionary background of the species that produce MIAs. Especially in the light of the chapter "Contrasting genomic features indicate convergent evolution of MIA biosynthesis" such a tree would be very illustrative.
Author's Response- Thank you so much for your comment. We have actually cited the article, "The nuclear genome of Rhazya stricta and the evolution of alkaloid diversity in a medically relevant clade of Apocynaceae", which does offer a little phylogenetic tree doing exactly what the reviewer suggested here. We assumed that our results, and by citing this article, we will be able to bring our point on "evolutionary relationships of Apocynaceae, Rubiaceae" in this chapter. Now, following the reviewer's suggestion, we, instead of creating a new phylogenetic tree, have now included symbols representing plants belonging to Apocynaceae or Rubiaceae family in Supplementary Fig. 18, and have modified the legend accordingly. The changes now address the concern that the reviewer expressed above.

And talking about this chapter: It still describes many interesting genomic features of the O. pumila genome (gene models, tRNAs,….), and other interesting observations like divergence times, WGD events. No doubt, all interesting observations, but I don't see any causal connection to MIA biosynthesis and how the authors can conclude from the results described in this chapter, that MIA biosynthesis has evolved convergently. The differential repeat profiles across O. pumila, C. roseus…. just proof that the repeats experienced different evolutionary forces but he MIA biosynthetic genes are possible

under purifying selection and thus remain in the genome/species – unaffected by the evolutionary fate of the repeats.

Author's Response- Thank you for your comments. Our results described in this section attempts to suggest parallel evolution of camptothecin biosynthesis, one of the most potent anti-cancer MIA. Indeed, the possibility of convergent or purifying selection can not be ascertained based on repeat contents and WGD analysis. However, in this chapter, as well as in the subsequent chapters and discussion, we have imphasized upon the two roads for camptothecin biosynthesis in Cornales versus Gentianales together with differential repeat content, WGD, evolution of species enzymes among other evidences to propose convergent evolution.

We feel that this section heading is appropriate as it offers a hypothesis, which we try to elaborate on further with evidences from comparative genomics, multi-omics analysis, gene-cluster analysis, and conserved synteny across producing plants. We end this section with following paragraph-

"MIA biosynthesis is known to be remarkably restricted to Gentianales, such as in Rubiaceae[24]. The exceptions are MIA quinolone derivatives, e.g., camptothecin, which is synthesized by Rubiaceae members such as *Ophiorrhiza* as well as by C. acuminata in the Cornales. Whole-genome duplications and transposable element movement are regarded as key mechanisms for evolving novel features in plants[36,37,38,39]. The differential repeat profiles across the *O. pumila*, *C. roseus*, *R. stricta*, and *C. acuminata* genomes and whole-genome duplication in *C. acuminata* suggest different trajectories of acting evolutionary forces, yet resulting in similar chemotypes across MIA-producing plants from the Gentianales and Cornales orders. These results raise the possibility of either a convergent evolution of MIA biosynthesis in otherwise distant plant species or an ancient origin of MIA biosynthesis, which is subsequently lost repeatedly across plant species while retained by the producing plants."

In this way, we tried to avoid any wild specultations while proposing a hypothesis, which we pursued in rest of the sections and in the discussion of the manuscript.

A few other things:

"Diverse indole alkaloids corroborates with enzyme families evolved in Ophiorrhiza genome" I don't get the message of the title.

Author's Response- The purpose of this section heading is to show a directed evolution of enzyme families that could bring the MIA diversity in the producing plants. We started this section by reporting diverse and conserved MIA across producing plants including several metabolites reported for *O. pumila* using isotope labeling experiment. Using comparative genomics, and gene-family gain/loss/expension/contraction analysis, we tried to show that producing plants evolved enzymes that were specific to the metabolite classes being produced. We feel that this section heading, taking into

consideration the entire content of this section, fits well, and therefore, we will like to keep it this way.

Fig. 4: I had some questions concerning Figure 4 concerning the color codes/legends, which are more than misleading in my opinion. It is of course nice that the authors kindly clarified Figure 4 to me, but the fact that the figure seems to be unintuitive did not really motivate the authors to improve the figure. The color code in the legend is still unclear. Some colors like brown are used in the circle plot, but are not given in the legend.
In Tsugawa et al. 2019 (Nature Methods volume 16, pages295–298. 2019) – the circle blot in Fig. 2 is nicely explained. But in the submitted paper, it is misleading. I try to be more specific: In the legend, the green color indicates “Indole”, in the circle plot – the green color seems to indicate “Arabidopsis thaliana”. If you want that the reader gets your message – indole = highest intensity in Arabidopsis - then please adjust your figure. The lines/rows in the circle represent the species, and the “columns” represent the compounds.
And also, please be aware – in the legend, the colors indicating organic acids and terpene glycoside are hard to distinguish.
Author’s Response- Thank you so much. We have now modified the figure exactly the way you suggested, and have also provided more details in order to make it more intuitive for better understanding. We have now changed the color for legends to make it look distinct, legend provide more details in terms of species and the links between metabolites across plant species. We appreciate your frank comments on this figure, and your specific suggestions, which allowed us to improve Fig. 4.

To summarize: Very interesting results were produced, but the writing needs more structure and focus.
Author’s Response- Thank you so much. As described above, we have now improved this manuscript in terms of structure and focus.

Reviewer #4 (Remarks to the Author):

All my conerns have been well addressed.
Author’s Response- Thank you so much.
